# Highly enantioselective synthesis of both tetrahydroquinoxalines and dihydroquinoxalinones *via* Rh–thiourea catalyzed asymmetric hydrogenation†

**DOI:** 10.1039/d3sc00803g

**Published:** 2023-07-06

**Authors:** Ana Xu, Chaoyi Li, Junrong Huang, Heng Pang, Chengyao Zhao, Lijuan Song, Hengzhi You, Xumu Zhang, Fen-Er Chen

**Affiliations:** a School of Science, Harbin Institute of Technology (Shenzhen) Taoyuan Street, Nanshan District Shenzhen 518055 China songlijuan@hit.edu.cn youhengzhi@hit.edu.cn rfchen@fudan.edu.cn; b Green Pharmaceutical Engineering Research Center, Harbin Institute of Technology (Shenzhen) Taoyuan Street, Nanshan District Shenzhen 518055 China; c Department of Chemistry, Shenzhen Grubbs Institute, Southern University of Science and Technology Shenzhen 518055 China; d Engineering Center of Catalysis and Synthesis for Chiral Molecules, Department of Chemistry, Fudan University Shanghai 200433 China

## Abstract

Chiral tetrahydroquinoxalines and dihydroquinoxalinones represent the core structure of many bioactive molecules. Herein, a simple and efficient Rh–thiourea-catalyzed asymmetric hydrogenation for enantiopure tetrahydroquinoxalines and dihydroquinoxalinones was developed under 1 MPa H_2_ pressure at room temperature. The reaction was magnified to the gram scale furnishing the desired products with undamaged yield and enantioselectivity. Application of this methodology was also conducted successfully under continuous flow conditions. In addition, ^1^H NMR experiments revealed that the introduction of a strong Brønsted acid, HCl, not only activated the substrate but also established anion binding between the substrate and the ligand. More importantly, the chloride ion facilitated heterolytic cleavage of dihydrogen to regenerate the active dihydride species and HCl, which was computed to be the rate-determining step. Further deuterium labeling experiments and density functional theory (DFT) calculations demonstrated that this reaction underwent a plausible outer-sphere mechanism in this new catalytic transformation.

## Abstract 2

Highly efficient asymmetric hydrogenation of quinoxalines and quinoxalinones *via* Rh–thiourea complex was disclosed under batch and flow. Mechanism studies demonstrated the importance of HCl and a plausible outer-sphere mechanism during the process.

## Graphical abstract

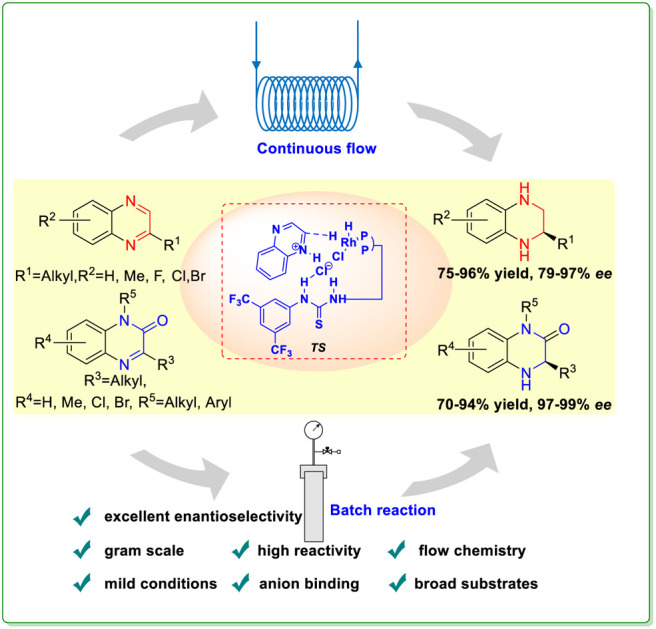

## Introduction

**Fig. 1:**
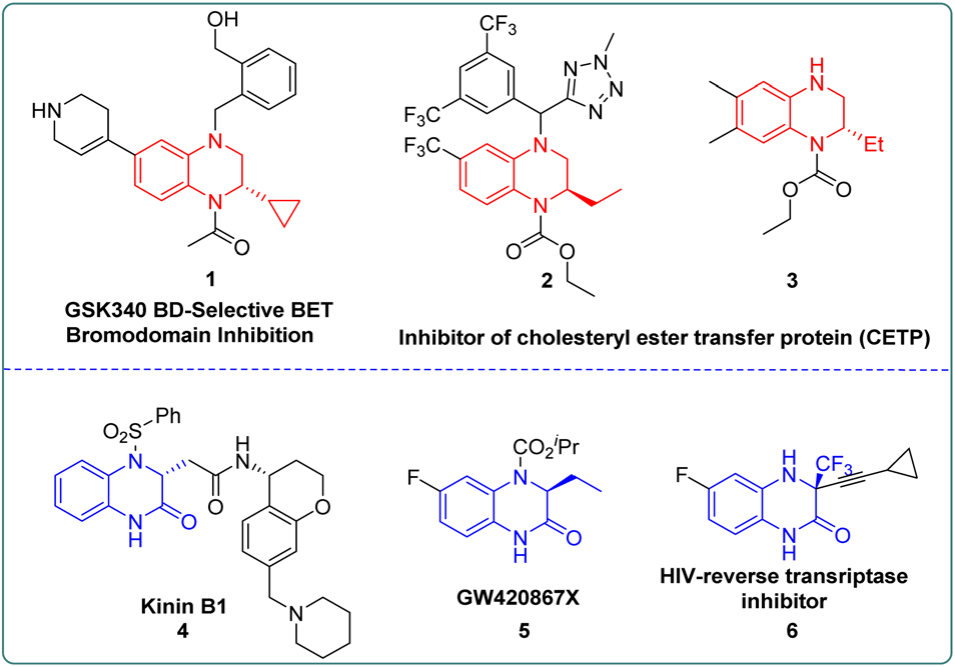
Bioactive molecules with chiral THQs and DHQs.

Chiral 1,2,3,4-tetrahydroquinoxaline (THQ) and 3,4-dihydroquinoxalinone (DHQ) ring units are crucial motifs of many bioactive molecules.^1^ Numerous optically pure THQs or DHQs have been developed as various pharmaceuticals or inhibitors. As shown in Fig. 1, the THQ fragment is an indispensable structural unit for cholesterol ester transfer protein (CETP) inhibitors in the treatment of atherosclerosis and obesity.^2^ In addition, kinin B1 can treat inflammation and pain caused by septicemia, and GW420867X is a non-nucleoside HIV-1 reverse transcriptase inhibitor, which both embed the chiral DHQs as crucial motifs.^3^ Therefore, developing efficient and concise methods to synthesize these compounds has attracted the attention of many chemistry researchers.^4^ Pioneering catalytic synthesis methods were established including intermolecular or intramolecular reaction with chiral substrates,^5,6^ asymmetric transfer hydrogenation^7^ and asymmetric hydrogenation.^8^ Among these, using chiral substrates to prepare enantiopure THQs or DHQs usually required multiple reaction steps and the introduction of the corresponding optically pure amino alcohols or amino acids in advance.^9^ On the other hand, asymmetric transfer hydrogenation which could simplify the reaction process, normally generated stoichiometric amounts of waste with the hydride source, leading to the reduction of the overall efficiency.^7*a*,10^ By contrast, asymmetric hydrogenation exhibited many advantages such as atom economy, selectivity, functional group tolerance and product expandability, which received wide attention.^11^

**Scheme 1:**
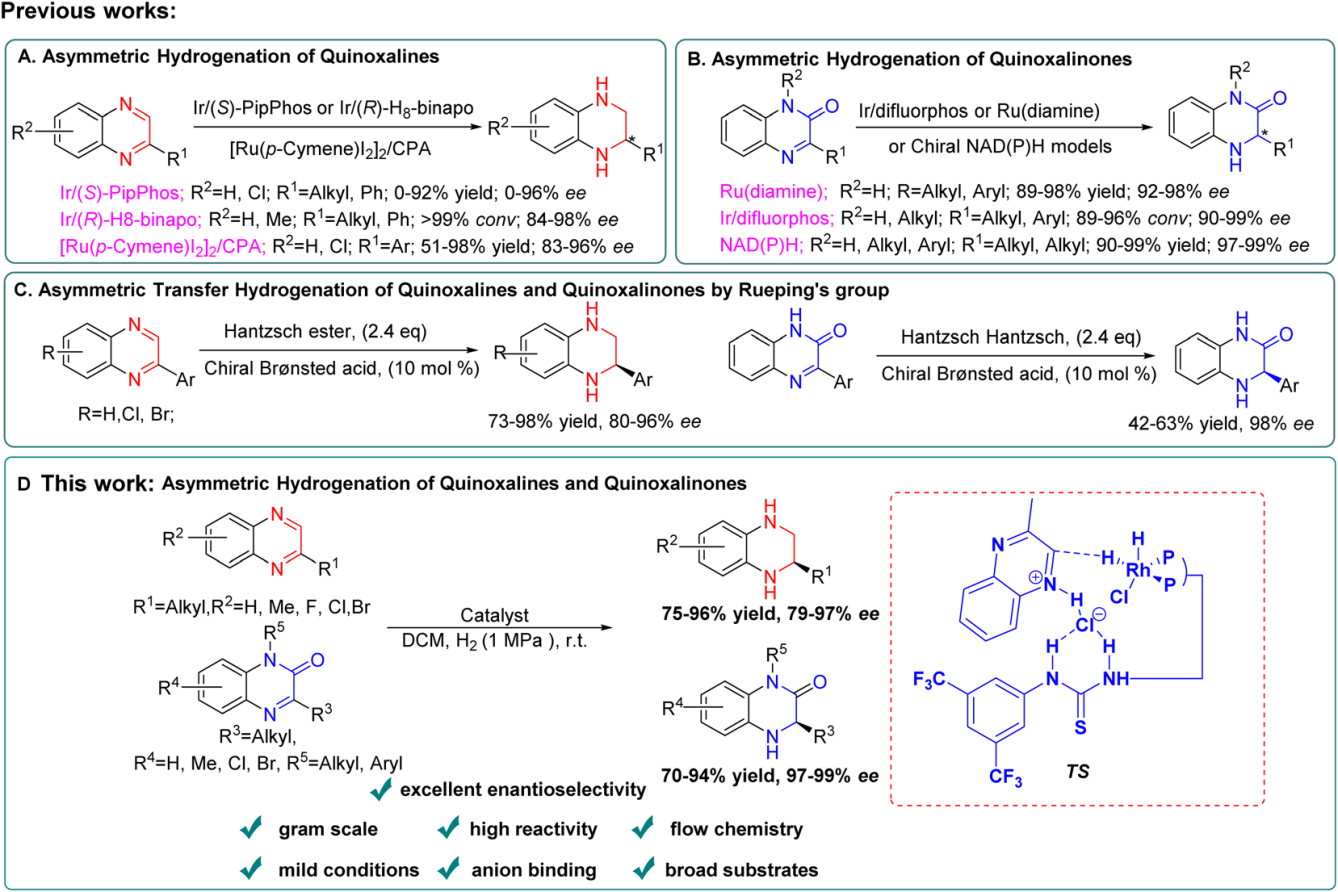
Asymmetric synthesis of chiral THQs and DHQs.

For quinoxaline derivatives, Murata's group reported pioneering work on asymmetric hydrogenation using Rh-Diop in 1987, even though the desired product was only isolated in 3% ee.^12^ Subsequently, other noble metals, such as Ir or Ru were explored with different bidentate ligands for asymmetric hydrogenation of quinoxaline derivatives with improved enantioselectivity (up to 90–90% ee).^13^ Until 2009, the more general and efficient catalytic systems were developed employing monodentate phosphoramidite ligand (*S*)-PipPhos, (*R*)-H_8_-binapo and chiral phosphoric acid (CPA) separately (Scheme 1A).^14^ For quinoxalinone derivatives, milestone studies were reported by Vidal-Ferran (Ir(P-OP)),^8*e*^ Zhou (chiral NAD(P)H models)^8*d*^ and Fan (Ru-diamine),^3*b*^ respectively (Scheme 1B).

However, in these cases, limitations such as the use of toxic solvents,^14*c*^ and the required high pressure^3*b*^ and low temperature^14*b*^ still existed making their broad industrial applications remain a challenge. In addition, these catalytic systems couldn't be simultaneously suitable for both asymmetric hydrogenation of quinoxalines and quinoxalinones. In 2010, Rueping's group reported the first general and efficient asymmetric transfer hydrogenation for quinoxalines and quinoxalinones using a Hantzsch ester and chiral Brønsted acid system, and the desired products were obtained with good to excellent enantioselectivities (Scheme 1C).^7*a*^ But their substrate scope is still limited to aromatic substituents and it's not suitable for industrial applications due to the use of stoichiometric amounts of Hantzsch ester.

Very recently, Zhang's group successfully developed a combination of Brønsted acid and thiourea-diphosphone-transition metal catalyst systems for asymmetric hydrogenation of imines,^15^ benzoxazinones,^16^ nitroalkenes,^17^ and quinolones^18^ to prepare enantiopure hydrogenated products with high chemical activity and excellent enantioselectivity. Enlightened by these results, we developed a highly enantioselective method to construct both optically pure THQs and DHQs under the same catalytic conditions (Scheme 1D). A series of various substrates were converted to the corresponding products with excellent yields (up to 98%) and enantioselectivities (up to 99% ee). Furthermore, application of this methodology was conducted successfully under continuous flow conditions. This flow transformation greatly reduced the hazard of hydrogen accumulation in batch with shorter reaction time and is more friendly to scale-up. We also demonstrated the asymmetric hydrogenation process by which quinoxaline and quinoxalinone derivatives underwent anion binding between the substrate and ligand *via* a ^1^H NMR mechanism study. In addition, the deuterium labeling experiments and density functional theory (DFT) calculations were conducted to gain more insight into the outer-sphere process of this new transformation and the origin of product enantioselectivity.

## Results and discussion

At the outset, we chose 2-methylquinoxaline hydrochloride (1a) as the model substrate to examine the efficiency of the Rh–thiourea-catalyst in this new transformation (Table 1). To our delight, the corresponding hydrogenation product (2a) was isolated in 99% conversion with 91% ee under otherwise identical conditions to previously reported conditions (Table 1, entry 1).^18*a*^ Encouraged by this initial result, we systematically investigated the influence of reaction parameters to enhance the reactivity and enantioselectivity. It was observed that the reaction could smoothly occur using [Ir(cod)Cl]_2_ with a slight drop in enantioselectivity (Table 1, entry 2). Subsequently, we examined the effect of the solvents. When we replaced ^*i*^PrOH with CHCl_3_, 99% conversion and 90% ee were obtained (Table 1, entry 3). In order to simplify the catalytic conditions, we performed the reaction in a single solvent. Polar protic solvents were found to give low enantioselectivities of products (Table 1, entries 4 and 5), while nonpolar aprotic solvents such as CHCl_3_ and DCM could provide the target product with 93% and 94% ee separately (Table 1, entries 7 and 8). Finally, attempts to further improve the yield and/or enantioselectivity by tuning the reaction temperature, time, and pressure were also performed. The following optimum conditions were identified: [Rh(cod)Cl]_2_ (0.5 mol%), (*R*, *R*)-L1 (1 mol%) as a chiral ligand, and 18 h, in DCM and at 25 °C under a 1 MPa H_2_ atmosphere. Notably, this good enantioselectivity could be realized at a molar substrate-to-catalyst (S/C) ratio of 1000, and the higher S/C ratio of 1500 showed a slightly lower enantioselectivity (Table 1, entry 14).

**Table 1:** Optimization of the reaction conditions for asymmetric hydrogenation of 2-methylquinoxaline hydrochloride 1a^a^

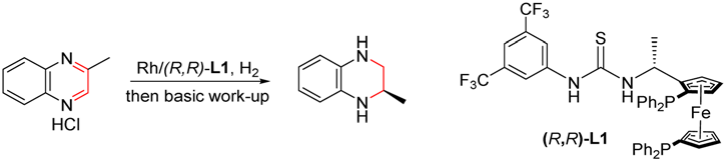						
Entry	Solvent	Temp. (°C)	Time (h)	H_2_ (MPa)	Conv. (%)	ee (%)
1	DCM : ^*i*^PrOH = 2 : 1	40	24	4	99	91
2^b^	DCM : ^*i*^PrOH = 2 : 1	40	24	4	99	85
3	DCM : CHCl_3_ = 2 : 1	40	24	4	99	90
4	EtOH	40	24	4	99	65
5	^*i*^PrOH	40	24	4	99	65
6	1,4-Dioxane	40	24	4	99	90
7	CHCl_3_	40	24	4	99	93
8	DCM	40	24	4	99	94
9	DCM	25	24	4	99	94
10	DCM	25	24	3	99	93
11	DCM	25	24	2	99	94
12	DCM	25	24	1	99	94
13	DCM	25	18	1	99/90^c^	94/90^c^
14^d^	DCM	25	18	1	99/99^e^	94/91^e^
15^f^	DCM	25	18	1	48	63

a Reaction conditions: 1a (0.25 mmol) in 2 mL solvent, 1a/[Rh(cod)Cl]_2_/ligand ratio = 100/0.5/1.

b Catalytic precursor is [Ir(cod)Cl]_2_.

c The reaction time is 12 h.

d S/C = 1000.

e S/C = 1500.

f Without HCl. Conversion was determined by ^1^H NMR analysis. ee was determined by HPLC.

To explore the effects of diverse thiourea ligands on the control of the enantioselectivity of Rh-catalyzed quinoxaline asymmetric hydrogenation, we also synthesized a series of analogs of the L1 ligand (Table 2). It was observed that the conversion rate and enantioselectivity of the reaction significantly dropped when using alkyl ester instead of aryl at the thiourea unit (L2). Furthermore, we found that the enantioselectivity of the reaction was sensitive to the substituents on the aryl ring (L1, L4, L5 and L6). This observation was also reported by Hunger's group where the conformational dynamics and hydrogen-bonding dynamics of thiourea-based ligands caused by the difference in substitutions on the benzene ring play an important role in catalytic activities.^19^ We also introduced an *N*-methyl on the thiourea unit (L3) which led to a slight decrease in catalytic activity. This phenomenon further confirmed the importance of secondary interaction between the chloride ion in the substrate and the hydrogen in the thiourea motif.

**Table 2:** Ligand evaluation for asymmetric hydrogenation of 2-methylquinoxaline hydrochloride 1a^a^

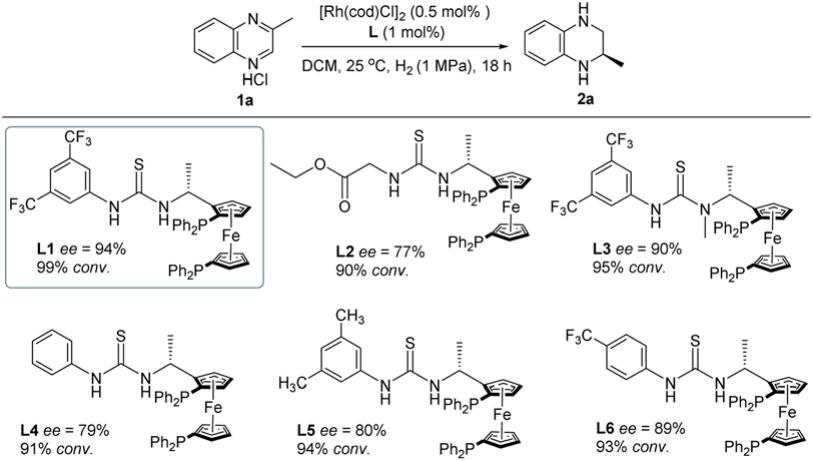

a Reaction conditions: 1a (0.25 mmol) in 2 mL solvent, 1a/[Rh(cod)Cl]_2_/ligand ratio = 100/0.5/1; conversion was determined by ^1^H NMR analysis; ee was determined by HPLC.

With the optimal reaction conditions in hand, we then evaluated the scope of quinoxaline derivatives (Table 3). Inspiringly, different alkyl substituents on *C*_2_ of quinoxalines were completely hydrogenated to the desired products with high yields and enantioselectivities (2a–2e). Although the substrate bearing a 2-isobutyl substituent afforded the desired product with moderate yield, the enantioselectivity wasn't eroded (2f). It is worth noting that the dehalogenation of halogenated aromatic compounds which was often seen under transition-metal-catalyzed hydrogenation was not observed here.^20^ Various halogenated groups such as Br, Cl and F were all tolerated under our hydrogenation system and delivered the desired products in excellent yields and enantioselectivities (2g–2j). In addition, the product enantioselectivity was found to be sensitive to the electron-donating groups on the aryl ring of the substrates (2k–2l). To further verify the practicability of this new Rh–thiourea-catalyzed quinoxaline asymmetric hydrogenation, we also magnified the reaction to the gram scale by using 6,7-difluoro-2-methyl-quinoxaline hydrochloride (1i) as the substrate. The reaction provided the corresponding product (2i) in 90% yield and 96% enantioselectivity without erosion.

**Table 3:** Scope of quinoxaline derivatives^a^

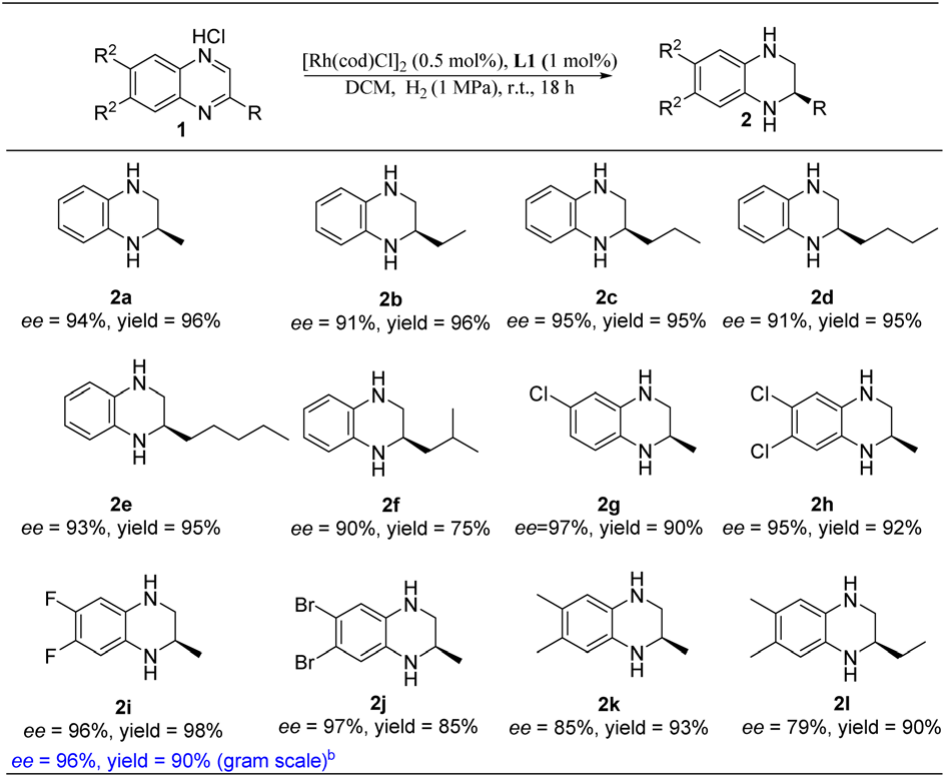

a Reaction conditions: 1a (0.25 mmol) in 4 mL dry DCM, 1a/[Rh(cod)Cl]_2_/L1 ratio = 100/0.5/1; yield was determined with isolated products; ee was determined by HPLC.

b 1i (1.08 g, 5 mmol) in 10 mL dry DCM, H_2_ (2 MPa), 24 h.

Encouraged by the excellent results of our catalytic system for asymmetric hydrogenation of quinoxaline derivatives, we envisioned that we could extend it to other heterocyclic substrates. The quinoxalinone substrate scope was evaluated as shown in Table 4. To our delight, substrates bearing various alkyl substituents at the N_1_ position gave the corresponding products in excellent yields (91–94%) and enantioselectivities (98–99% ee) (4a–4e). In addition, we performed the gram-scale reaction of 1,3-dimethyl-2-quinoxalinone hydrochloride (3a) and afforded 4a with undamaged yield and enantioselectivity. Furthermore, the substrate bearing an ethyl group at the C_3_ position was smoothly reduced with high enantioselectivity (4f). Substrates with substituents at C_6_ and C_7_ positions of quinoxalinones also performed well to deliver the desired products in excellent yield with 98–99% ee (4g–4i). Moreover, the introduction of various arylmethyls at the N_1_ position of substrates afforded the corresponding hydrogenation products with excellent yield and enantioselectivity (4j–4m). Unfortunately, our catalytic system was found to be not tolerant for the substituents of ^*i*^Pr, Cy, and the aromatic group (Table S1†). Among them, the configurations of 4g and 4h were assigned as *R via* single crystal X-ray diffraction (Tables S2 and S3†).

**Table 4:** Scope of quinoxalinone derivatives^a^

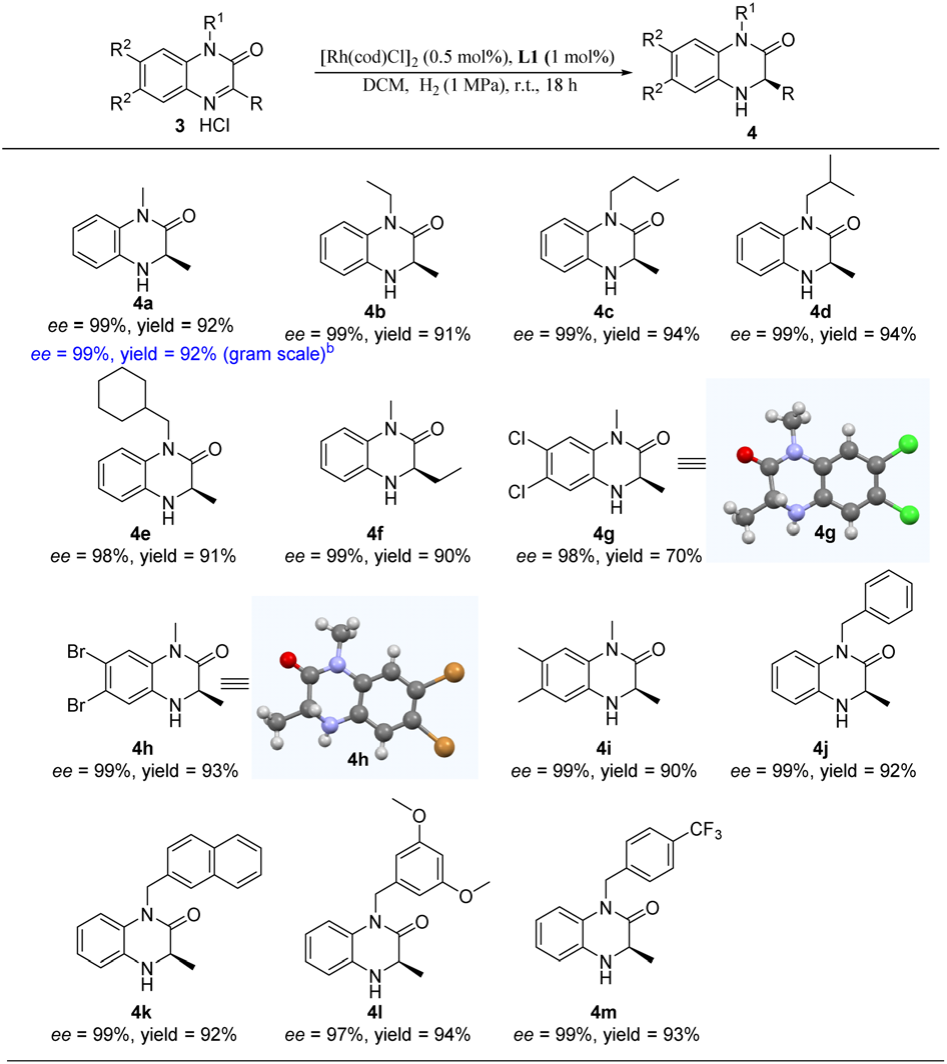

a Reaction conditions: 3 (0.25 mmol) in 4 mL dry DCM, 3/[Rh(cod)Cl]_2_/L1 ratio = 100/0.5/1; yield was determined with isolated products; ee was determined by HPLC.

b 3a (1.05 g, 5 mmol) in 10 mL dry DCM, H_2_ (2 MPa), 24 h.

**Scheme 2:**
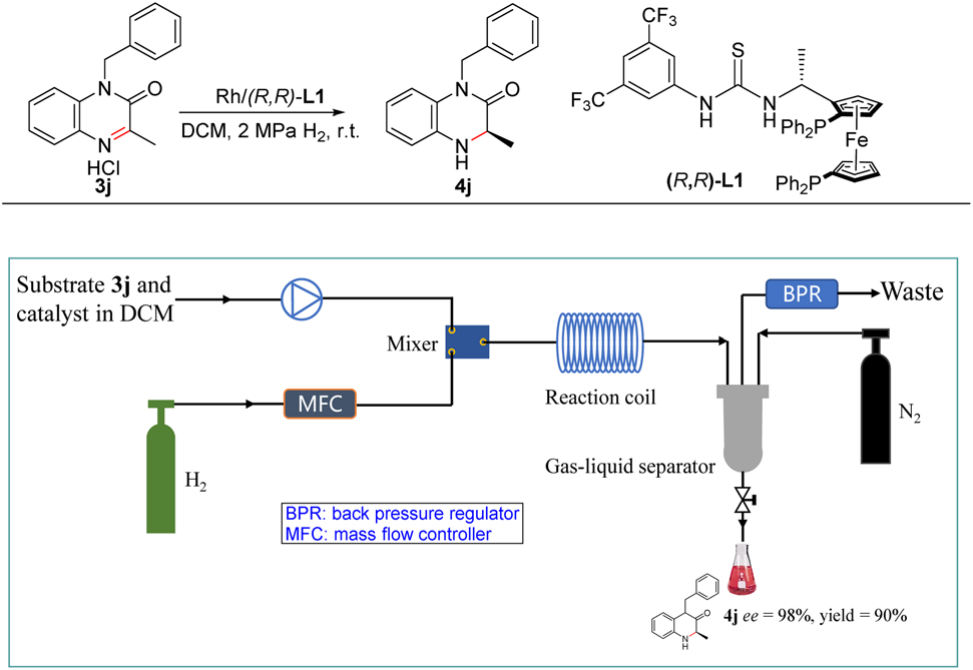
Asymmetric homogeneous hydrogenation of substrate 3j under continuous flow conditions.

Recently, flow chemistry received remarkable attention in asymmetric hydrogenation because of advantages such as short residence time, high surface area-to-volume ratio, excellent reproducibility, easy scale-up, *etc.*^21^ More importantly, this technology can reduce the hazards associated with the use of hydrogen gas which is highly flammable and forms an explosive mixture in air.^22^ Therefore, this new Rh-catalysed asymmetric hydrogenation system was also applied to the continuous flow conditions (Scheme 2). First, solution of substrate 3j and Rh–thiourea-catalyst was set in one stream, H_2_ gas was set in another stream, and the two phases were sufficiently mixed through a microchip mixer. Then the relative parameters were investigated including the concentration of the substrate, gas velocity, liquid velocity, the pressure of H_2_ and the residence time of this reaction (Table S5†). Finally, the desired product 4j was obtained in high yield with excellent enantioselectivity at room temperature and 2 MPa H_2_ within 25 min. Subsequently, a gram-scale continuous production was performed for this new Rh-catalysed asymmetric hydrogenation. 2.5 g product was obtained with 90% yield and 98% ee with more than 7.5 h with a high TON and TOF (180 and 428 h^−1^, respectively).

**Fig. 2:**
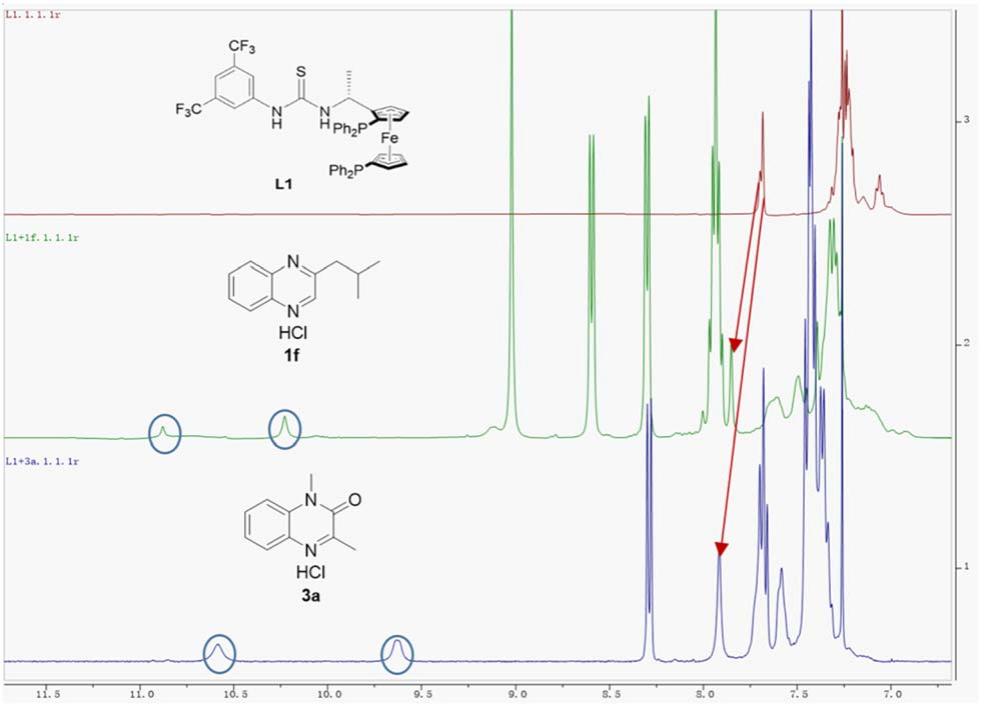
^1^H NMR study of the interaction of L1 (0.02 M) with 1f (0.06 M) and 3a (0.06 M) in CDCl_3._

In order to obtain insight into this new asymmetric hydrogenation mechanism, we first investigated the influence of hydrochloric acid. It was observed the model reaction using 2-methylquinoxaline as the substrate in the absence of hydrochloric acid gave 48% conversion with 63% ee in batch (Table 1 entry 15^*f*^). This result indicated that the strong Brønsted acid HCl was essential in this chemical transformation. Further observation reveals that the reaction rate is controlled by both Cl^−^ and H^+^, and Cl^−^ is also crucial for the ee value (Table S6†). We then mixed ligand L1 with quinoxaline hydrochloride 1f (3 eq.) and quinoxalinone hydrochloride 3a (3 eq.) in CDCl_3_, respectively. The ^1^H NMR study showed that the chemical shift of ligand L1 shifted significantly downfield in both mixtures compared to single L1 trace (Fig. 2). The original N–H peaks of thiourea were hidden in the aromatic peaks within 7.3–7.0 ppm, but they have shifted downfield to 10.92 ppm and 10.27 ppm in the mixture with 1f (marked by a blue circle in Fig. 2). The N–H peak of thiourea moved to 10.62 ppm and 9.66 ppm in the mixture with 3a (marked by a blue circle in Fig. 2). In addition, three hydrogen atoms on the 3,5-bis(trifluoromethyl)phenyl group of L1 were also found to be shifted slightly downfield in both mixtures with 1f and 3a (marked by the red arrows in Fig. 2). In contrast, when quinoxaline is mixed directly with the ligand without preparing the salt in CDCl_3_, no recognition was observed (Fig. S4†). These results were consistent with the literature report,^18*a*^ which showed that the anion bond formed between the chloride ion in the substrate and the hydrogen in the thiourea motif.

**Scheme 3:**
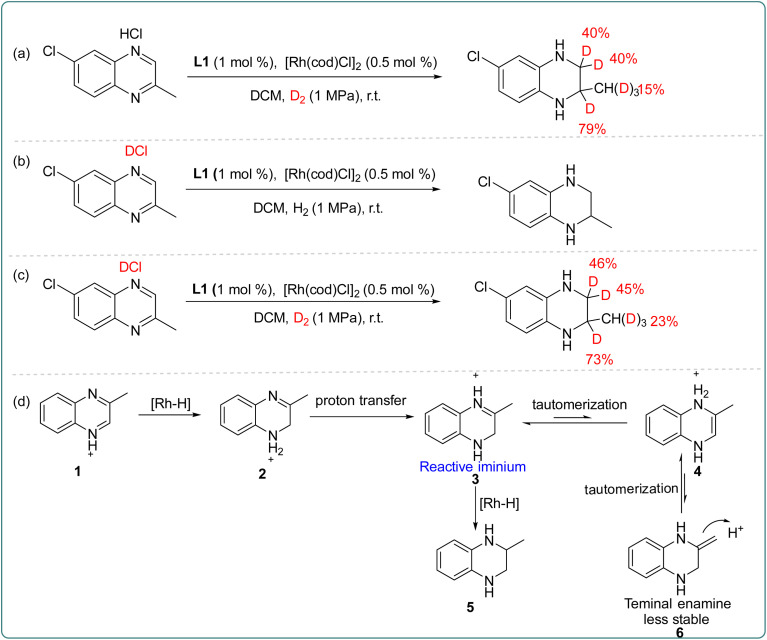
Deuterium labeling experiment and the proposed transformation path.

Subsequently, to further explore the source of hydrogen during the catalytic process, the deuterium labeling experiments were carried out as shown in Scheme 3. First, asymmetric hydrogenation of 6-chloro-2-methylquinoxaline hydrochloride was performed in D_2_ gas, and the D atom was added at 2- and 3-positions (Scheme 3a). The experiment revealed that a small amount of deuteration occurred both in the normal hydrogen atom at the 3-position and the methyl group at the 2-position, which may be caused by tautomerism in the transformation process. Secondly, asymmetric hydrogenation was carried out in deuterated hydrochloride, and no deuterium product was observed (Scheme 3b). Finally, 6-chloro-2-methylquinoxaline deuterated hydrochloride was hydrogenated in D_2_ (Scheme 3c), and the results were found to be similar to those in Scheme 3a. Based on these results, we proposed a possible transformation path (Scheme 3d). In the Rh–thiourea bisphosphine catalytic system, the oxidative addition of H_2_ occurred; then the Rh–H bond broke, and the hydride was transferred to the C_2_ position generating the partially reduced intermediate 2. The intermediate 2 would not directly undergo the next hydrogenation, but went through proton transfer to generate the imine intermediate 3, and then the enamine intermediates 4 and 6 were obtained through another tautomerism. In contrast, intermediate 3 was more stable and more prone to hydrogenation than intermediate 6. Therefore, the hydrogenation happened from intermediate 3 to give the final product.

**Scheme 4:**
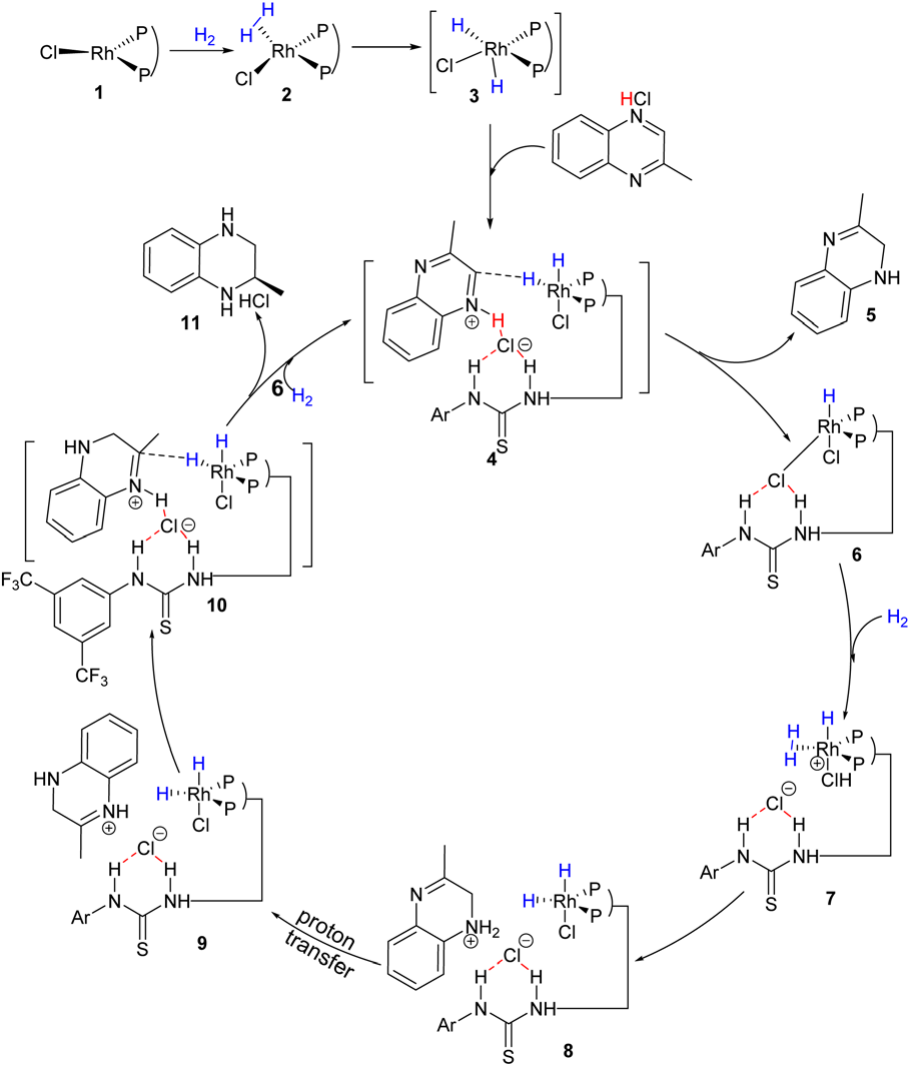
Outer-sphere mechanism for quinoxaline asymmetric hydrogenation.

**Fig. 3:**
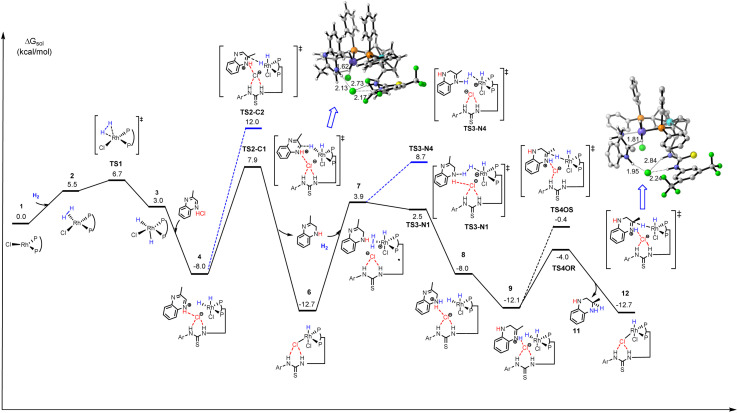
DFT calculated energy potential surface for the proposed pathway (Ar = 3,5-di-CF_3_Ph; the phenyl groups on the phosphorus atoms are omitted for clarity; the relative Gibbs energies are labeled in kcal mol^−1^).

To further explore in more detail this new asymmetric hydrogenation mechanism, DFT calculations for asymmetric hydrogenation of 2-alkyl quinoxaline were also conducted. First, the linear effect experiment (Table S7 and Fig. S5†) and high-resolution mass spectroscopy (Fig. S6†) were conducted for investigation of the binding manner between the ligand and the chloride ion. This was different from Jacobsen's case which involved a 2 : 1 ratio.^23^ The results suggested a 1 : 1 binding pattern. Here we proposed a plausible outer-sphere mechanism for this Rh-catalyzed quinoxaline asymmetric hydrogenation based on DFT calculations (Scheme 4 and Fig. 3).^24^ First, 2-methylquinoxaline was protonated by HCl, and then, a catalyst–substrate complex 4 was formed. Simultaneously, the chloride ion formed hydrogen bonds with the thiourea of L1 and the protonated 2-methylquinoxaline NH group. Then the hydride of the active rhodium complex was transferred to the substrate. Since previous protonation may happen on a different nitrogen, there were two possible carbon sites for hydrogen transfer (TS2-C_1_ and TS2-C_2_, Fig. 3). The calculation results showed that the hydrogen transferred to the carbon with alkyl substitution *via*TS2-C_2_ was 4.1 kcal mol^−1^ higher in energy than the other. The first hydrogenation led to partially reductive intermediate 5. Next, another H_2_ molecule coordinated with catalyst 6 formed intermediate 7, and the chloride ion facilitates heterolytic cleavage of dihydrogen to regenerate the active dihydride species and HCl, which was computed to be the rate-determining step. The transition states *via*TS3-N_1_ or TS3-N_4_ involved hydrogen transfer concerted with oxidative addition. Notably, the next protonation occurred at N_4_ directly as TS3-N_4_ showed 6.2 kcal mol^−1^ lower energy than TS3-N_1_, which can be attributed to the sp^3^ nitrogen being more basic than the sp^2^ nitrogen. Therefore, the intermediate 8 was first produced and went through the process of proton transfer to generate a more stable intermediate 9. Then the active dihydride species will undergo a second recognition of the substrate *via* anion binding between the substrate and the ligand along with the insertion of hydride from a rhodium dihydride complex. The second hydride transferred preferentially to the Si-face at C_2_ of the protonated quinoxaline in the *R* configuration (TS4O*R*) to generate product 11 and catalyst 6. TS4O*R* was computed to be lower in free energy than TS4O*S* by 3.8 kcal mol^−1^, partly due to a larger distortion of the metal–ligand part in TS4O*S*, as indicated by the largely distorted dihedral angle of −47.97° (Fig. S8†). In summary, our computational results were qualitatively consistent with the experimental observations and supported the cooperative effect of the Rh catalyst, Brønsted acid, and anion binding in asymmetric hydrogenation.

## Conclusions

In summary, a green, mild and practical asymmetric hydrogenation procedure to synthesize both enantiopure THQs and DHQs *via* a Rh–thiourea diphosphine catalytic system has been disclosed. The method allowed for the enantioselective transformation of various quinoxaline and quinoxalinone derivatives in excellent yields and enantioselectivities. In addition, the reactions were magnified to the gram scale under both batch and continues flow conditions without erosion of the catalyst activity and enantiomeric excess. The ^1^H NMR experiments show that the introduction of the strong Brønsted acid HCl played an important role both in activating the substrate and establishing anion binding between the substrate and the ligand in this new Rh-catalyzed asymmetric hydrogenation. Deuterium labeling experiments revealed that equilibrium tautomerism occurred between enamine and imine after the first hydrogenation. The later detailed DFT calculations further demonstrated an outer-sphere mechanism for this new transformation. We anticipate that this work will provide a milder and more practical synthetic tool for the efficient and highly enantioselective synthesis of both THQs and DHQs. The mechanistic studies, as well as the continuous flow application, will aid in enlightening further efficient and industrially friendly processes for asymmetric hydrogenation.

## Data availability

Our experimental or computational data associated with this article have been deposited in ESI.†

## Author contributions

The manuscript was written through contributions of all authors. All authors have given approval to the final version of the manuscript.

## Conflicts of interest

There are no conflicts to declare.

## Supplementary Material

SC-014-D3SC00803G-s001
