# Supplementary material for: Highly enantioselective synthesis of both tetrahydroquinoxalines and dihydroquinoxalinones via Rh–thiourea catalyzed asymmetric hydrogenation
Source: Chem Sci. 2023 Jul 6;14(34):9024–32. doi: 10.1039/d3sc00803g (PMC10466277; doi:10.1039/d3sc00803g)

Electronic Supplementary Material (ESI) for Chemical Science.
This journal is © The Royal Society of Chemistry 2023

Supporting Information

Highly Enantioselective Synthesis of both Tetrahydroquinoxalines and Dihydroquinoxalinones via Rh-thiourea Catalyzed Asymmetric Hydrogenation

Ana Xu[a], Chaoyi Li[a], Junrong Huang[a], Heng Pang[a], Chengyao Zhao[a], Lijuan Song[a]*, Hengzhi You [a,b]*, Xumu Zhang[c] and Fen-Er Chen[a,b,d]*

[a]School of science, Harbin Institute of Technology (Shenzhen), Guangdong, China. [b]Green Pharmaceutical Engineering Research Center, Harbin Institute of Technology (Shenzhen), Guangdong, China. [c]Shenzhen Grubbs Institute and Department of Chemistry, Southern University of Science and Technology, Shenzhen 518055, China. [d]Engineering Center of Catalysis and Synthesis for Chiral Molecules, Department of Chemistry, Fudan University, Shanghai, China.

* Corresponding authors

songlijuan@hit.edu.cn; youhengzhi@hit.edu.cn; rfchen@fudan.edu.cn

Table of contents

1. General remarks.. 1

2. General procedure for the synthesis of substrates and intermediates 1

   2.1. Synthesis of quinoxaline substrates.. 1

   2.2 Synthesis of quinoxalinone substrates.. 2

3. General procedure for preparation of quinoxaline/quinonalinone hydrochlorides............ 3

4. General procedure for asymmetric hydrogenation of quinoxaline/quinonalinone chlorides 4

5. Result of deuterium labeling experiments.. 4

6. Assignment of Absolute Configuration... 6

7. General procedure for asymmetric hydrogenation under continuous flow 8

8. The influence of H^+ and Cl^- respectively 10

9. linear Effect Experiment.. 12

10. Characterization data of chiral Tetrahydroquinoxalines and Dihydroquinoxalinones. 14

11. DFT computational studies 33

   11.1 Distortion calculation 33

   11.2 Method for DFT calculation... 34

   11.3 Cartesian Coordinates.. 37

12. Supporting References 60

13. NMR Spectra 62

1. General remarks

All the reactions dealing with air-or moisture-sensitive compounds were carried out in a dry reaction vessel under a positive pressure of argon or in the argon-filled glovebox. ^{1}H NMR and ^{13}C NMR spectra were recorded using Q. One Instruments Quantum-I 400M spectrometer. ^{1}H NMR and ^{13}C NMR chemical shifts were reported in parts per million (ppm) downfield from tetramethylsilane. Coupling constants (*J*) are reported in Hertz (Hz). The residual solvent peak was used as an internal reference: ^{1}H NMR (chloroform δ 7.26) and ^{13}C NMR (chloroform δ 77.0). The following abbreviations were used to explain the multiplicities: s = singlet, d = doublet, t = triplet, q = quartet, m = multiplet, br = broad. HRMS were obtained on Waters Xevo G2-XS QTof. All *substrates* were prepared according to the published procedures. Other reagents were received from commercial sources. Solvents were freshly dried and degassed according to the published procedures prior to use. Column chromatography purifications were performed by flash chromatography using Merck silica gel 60. Enatioselectivities were measured by HPLC (Thermo Ultimate 3000).

2. General procedure for the synthesis of substrates and intermediates

2.1. Synthesis of quinoxaline substrates

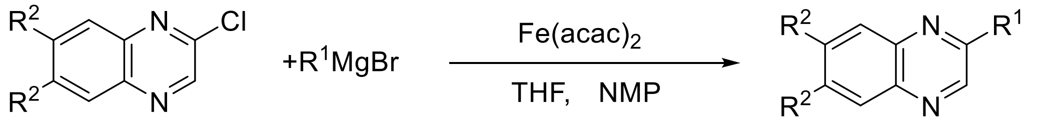

2-Alkyl-substituted quinoxalines: 2-Alkyl-substituted quinoxalines **1b-1g** were prepared by the iron-catalyzed Kumada reaction with 2-chloroquinoxalines according to the methods previously reported in the literature.[1] Under Ar atmosphere, 2-chloroquinoxaline (1.26 g, 7 mmol) and iron(II) acetylacetonate (88.9 mg, 0.35 mmol) were dissolved in 40 mL dry THF and 4 mL *N*-methyl-2-pyrrolidone was added. A Grignard solution (8 mmol) was added dropwise at room temperature over 10 min. After stirred for 20 min, the reaction mixture was diluted with ether (50 mL) and quenched with 1M aqueous HCl solution (15 mL). The ether layer was separated, washed with water and brine, dried over Na_2SO_4, and the solvent was removed under vacuum. Further purification was performed by a silica gel column eluted with petroleum ether/ethyl acetate (10/1, V/V) to give the pure product.

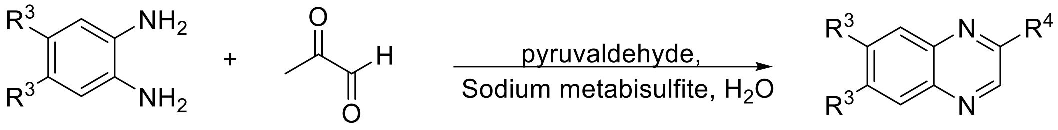

6,7-dichloro-2-methylquinoxaline (1h)[2]: Sodium metabisulfite (30 mmol) was added to a solution of pyruvaldehyde (10 mmol) in water, then, 1,2-phenylenediamine (7.8 mmol) was added and the solution was stirred at room temperature. After 18h the solution was adjusted to pH 10 by the addition of sodium carbonate (Na_2CO_3), the mixture was extracted with ether (3 × 30 mL). The organic phase was dried over magnesium sulfate ($MgSO_4$), filtered, and concentrated under reduced pressure. Purification by flash column chromatography (Ethyl acetate: Petroleum ether 4:1, v/v) gave **11** as a yellow solid (80%). Substrates **1i-1k** were prepared with the same method.

2.2 Synthesis of quinoxalinone substrates

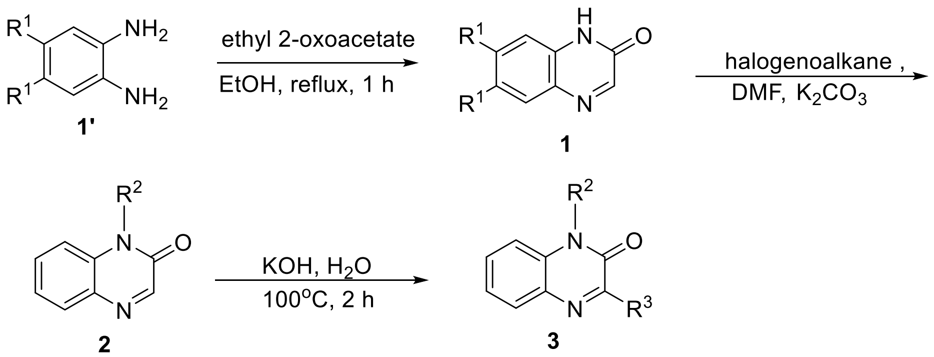

A mixture of o-phenylenediamine (5 mmol), ethyl 2-oxoacetate (6 mmol) and ethanol (20 mL) in a dried 50 mL round-bottom flask was stirred at reflux for 1 hour. After the completion (as indicated by TLC), the reaction mixture was filtered, washed with ethanol and then dried to give quinoxalinone 1. Subsequently, A mixture of quinoxalinone 1, K_2CO_3 (1.2 equiv.), corresponding halogenoalkane (1.6 equiv.) and DMF (20 ml) in a dried 50 mL round-bottom flask was stirred at room temperature overnight. After the completion (as indicated by TLC), the mixture was then extracted with ethyl acetate and the collected organic layer was washed with brine, dried with $MgSO_4$. The solvent was removed under reduced pressure, and the crude product was further purified by silica gel column chromatography (200-300 mesh silica gel, PE/EA = 5:1) to afford desired substrates 2.[3] To an oven-dried sealed tube charged with substrates 2 (800 mg, 1.0 equiv.), KOH (840 mg 3.0 equiv.), and trimethylsufoxonium iodide (2a) (3.3 g, 3.0 equiv.) was added H_2O (5 mL) at room temperature under air. The reaction mixture was allowed to stir at 100 °C for 2 h. The reaction mixture was cooled to room temperature and filtered through a bed of Na_2SO_4. The solid bed was washed with a mixture of CH_2Cl_2 and MeOH (9:1). The filtrate was concentrated under reduced pressure. The residue was purified by flash chromatography (CH_2Cl_2/acetone = 97:3) to afford **3** in 85% yield.[4]

2.3. Synthesis of ligands L1-L6

L1, L3-L6 were prepared according the according the literature, and All the spectral data are consistent with the literature values.[5, 6]

L2 was prepared as follow;

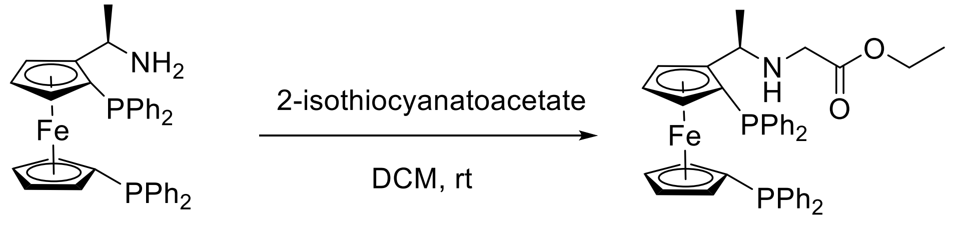

2 L2

Firstly, intermediate 2 (120 mg, 0.2 mmol) was dissolved in 5 mL methylene chloride, ethyl 2-isothiocyanatoacetate (36 mg, 0.25 mmol) were added. Subsequently, the mixture was reacted at room temperature for 6 h, and substrate 2 was completed by TLC detection. Next, 100 mL dichloromethane was added after the reaction was cooled to room temperature. The mixture was collected and column chromatography was used to purify coarse product, then Ligand **L2** was obtained (83 mg) with the yield of 60%. ^{1}H NMR (400 MHz, DMSO-d_6) δ 7.50 – 7.28 (m, 11H), 7.24 – 7.11 (m, 7H), 6.99 (td, J = 6.2, 5.0, 3.2 Hz, 2H), 5.33 (s, 1H), 4.54 (d, J = 2.8 Hz, 1H), 4.47 (q, J = 1.9 Hz, 1H), 4.22 – 4.15 (m, 2H), 4.13 – 3.92 (m, 4H), 3.77 – 3.62 (m, 1H), 3.45 (d, J = 11.1 Hz, 2H), 1.36 (d, J = 6.6 Hz, 3H), 1.16 (t, J = 7.1 Hz, 3H). ^{13}C NMR (101 MHz, Chloroform-d) δ 169.75, 139.05, 138.28 (d, J = 8.9 Hz), 135.94, 134.93 (d, J = 21.0 Hz), 133.69 (d, J = 20.0 Hz), 133.18 (d, J = 19.2 Hz), 132.60 (d, J = 18.7 Hz), 129.46, 128.66 (d, J = 21.7 Hz), 128.25 (t, J = 7.2 Hz), 95.53 (d, J = 23.8 Hz), 75.31 (d, J = 19.6 Hz), 74.31, 73.45 (d, J = 11.0 Hz), 73.02, 72.17, 71.53, 14.24. ^{31}P NMR (162 MHz, Chloroform-d) δ -17.86, -24.97.

$[\alpha]_D^{25}$ = -272.166 (c 0.1, $CHCl_3$).

HRMS (ESI) calcd for $C_{40}H_{40}FeO_2P_2$ $[(M+H)^+]$ 684.1878, found 684.1877.

3. General procedure for preparation of quinoxaline/quinonalinone hydrochlorides

Quinoxalines or quinoxalionnes (1mmol) was dissolved in 2 mL dry ethyl acetate, 1 mL hydrogen chloride solution (2 M) in 1,4-dioxane (2 eq.) was added dropwse. White or yellow precipitate was form. The resulting mixture was filtered and the solid was washed with dry ether (2 mL*2). The solid was collected and dried under vacuum. Quinoxalines or quinoxalionnes chloride was obtained.

4. General procedure for asymmetric hydrogenation of quinoxaline/quinonalinone chlorides

In the nitrogen-filled glovebox, solution of $[Rh(cod)Cl]_2$ (1.25 mg, 0.0025 mmol) and ligand (4.4 mg, 0.005 mmol) in 2.0 ml anhydrous solvent was stirred at room temperature for 30 min. A specified volume of the resulting solution (1 mL, 0.5% Rh catalyst) was transferred by syringe to a Score-Break ampule charged with substrate solution (0.25 mmol in 0.5 mL). The ampule was placed into an autoclave, which was then charged with 1 MPa H_2. The autoclave was stirred at room temperature for the indicated period of time. After release of H_2, the resulting mixture was concentrated under vacuum. Saturated potassium carbonate solution and dichloromethane was added and the mixture was stirred for 30 min. The organic layer was dried with anhydrous sodium sulfate. After removal of solvent, the crude product was analysed by ^{1}H NMR to determine the conversion. The enantiomeric excess was determined by HPLC analysis of the crude product.

Table S1. Scope of quinoxaline derivatives

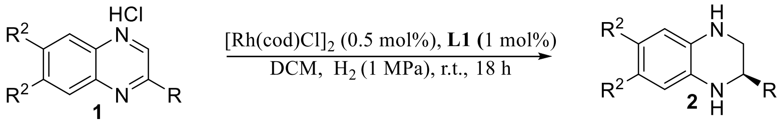

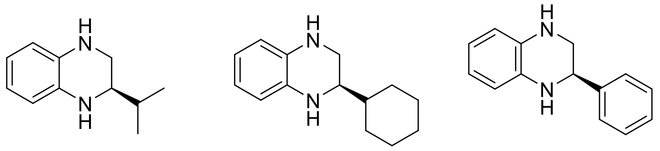

2m *ee* = 31%, yield = 55%

2n *ee* = 33%, *yield* = 80%

2o *ee* = 19%, yield = 75%

Reaction conditions: **1** (0.25 mmol) in 4 mL dry DCM, **1**/$[Rh(cod)Cl]_2$/ **L1** ratio = 100/0.5/1; yield was determined with isolated products; *ee* was determined by HPLC.

5. Result of deuterium labeling experiments

Following standard hydrogenation procedure, deuterium labeling experiments were conducted with specific modification.

(a)

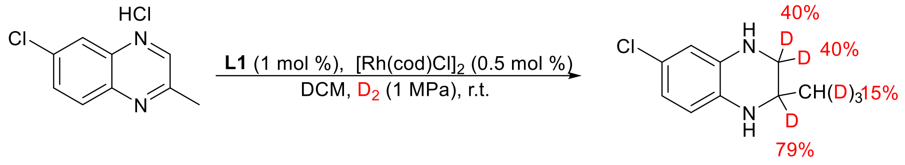
HCl
Cl
N
N
L1 (1 mol %), [Rh(cod)Cl]2 (0.5 mol %)
DCM, D2 (1 MPa), r.t.
H
N
N
H
D
D
D
CH(D)3
40%
40%
15%
79%

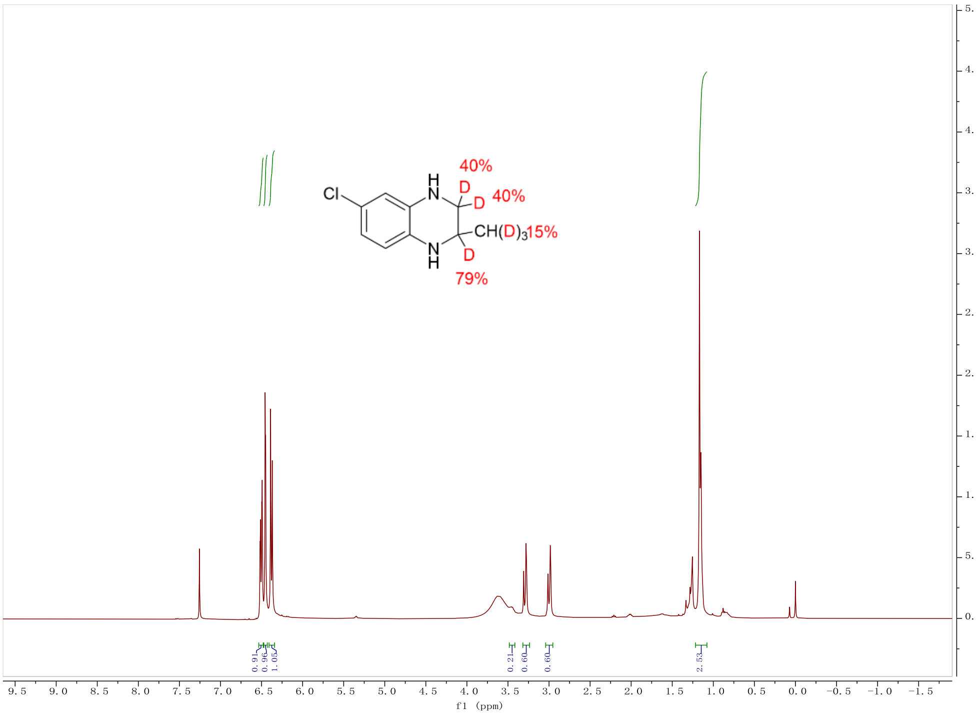
40%
40%
15%
79%
CH(D)3
f1 (ppm)

(b)

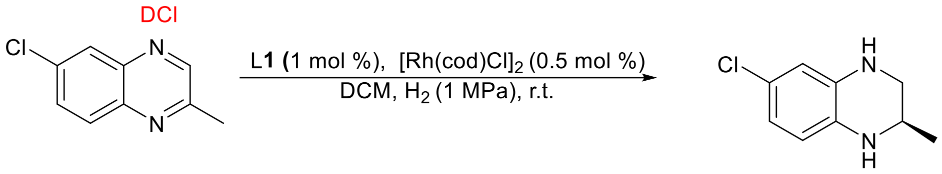
DCl
Cl
N
N
L1 (1 mol %), [Rh(cod)Cl]2 (0.5 mol %)
DCM, H2 (1 MPa), r.t.
H
N
N
H

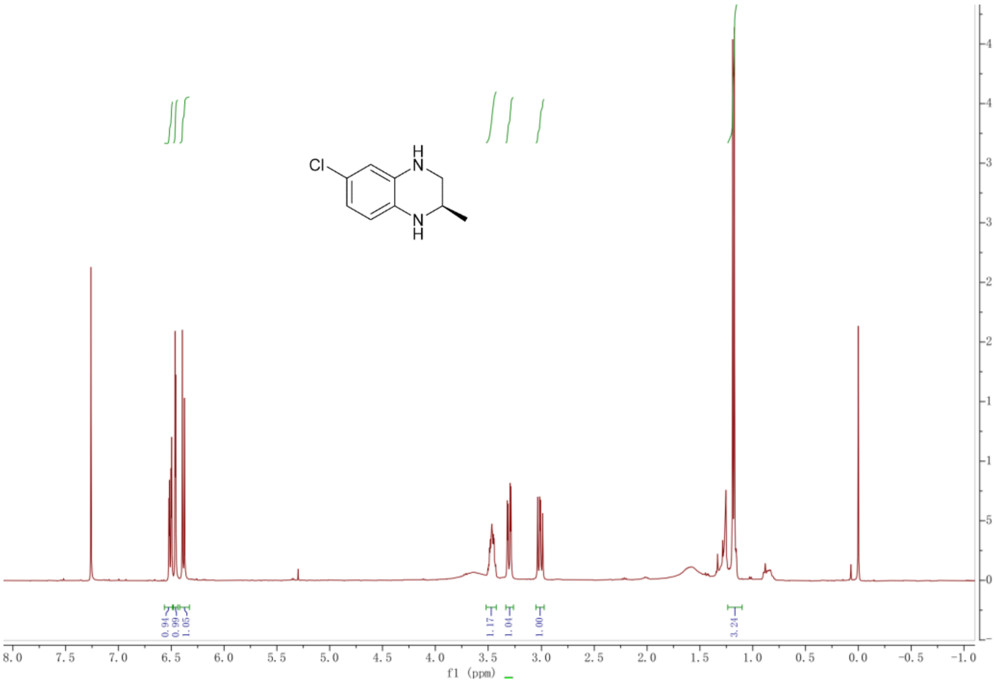
f1 (ppm)

(c)

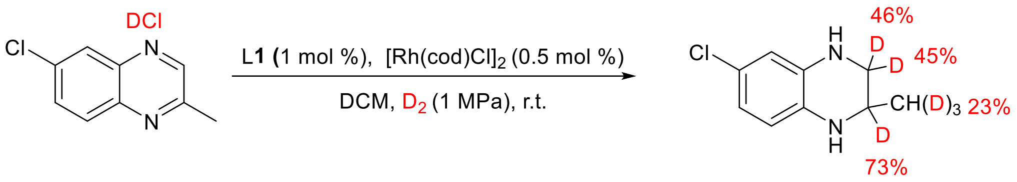

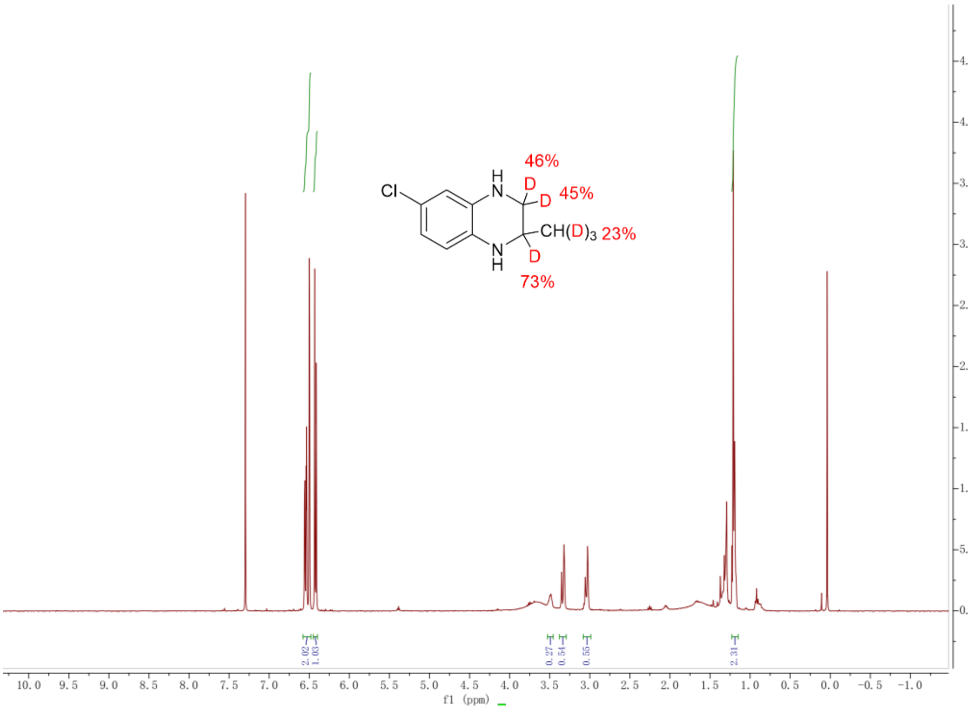

6. Assignment of Absolute Configuration

To assign the absolute configurations, crystal structures of compound **4g**, and **4h** were provided. Specifically, (*R*)-6,7-dichloro-1,3-dimethyl-3,4-dihydroquinoxalin-2(1H)-one (**4g**) and (*R*)-6,7-dibromo-1,3-dimethyl-3,4-dihydroquinoxalin-2(1H)-one (**4h**) were prepared according to GP D and crystallized from $CHCl_3$ at 25 °C. The absolute configuration of **4g**, and **4h** were unambiguously determined by single-crystal X-ray crystallography. And the X-ray data have been deposited at the Cambridge Crystallographic Data Centre (**4g**: CCDC 2182122, **4h**: CCDC 2182123 respectively).

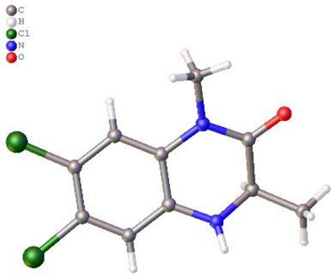

Figure S1. ORTEP drawing of compound **4g** (CCDC:2182122)

Table S2 Crystal data and structure refinement for 4g.

Identification code	N-8-EE_auto
Empirical formula	$C_{10}H_{10}Cl_2N_2O$

Formula weight	245.109
Temperature/K	300.73(13)
Crystal system	orthorhombic
Space group	$P2_12_12_1$
a/Å	7.2488(3)
b/Å	11.6665(3)
c/Å	12.7099(3)
α/°	90
β/°	90
γ/°	90
Volume/Å^3	1074.85(6)
Z	4
ρ_{calc}g/cm^3	1.515
μ/mm^{-1}	5.223
F(000)	508.3
Crystal size/mm^3	0.7 × 0.7 × 0.3
Radiation	Cu Kα (λ = 1.54184)
2Θ range for data collection/°	10.3 to 151.92
Index ranges	$-8 \leq h \leq 3$, $-11 \leq k \leq 14$, $-15 \leq l \leq 14$
Reflections collected	3792
Independent reflections	1960 [R_{int} = 0.0296, R_{sigma} = 0.0368]
Data/restraints/parameters	1960/0/139
Goodness-of-fit on F^2	0.987
Final R indexes [I>=2σ (I)]	R_1 = 0.0353, wR_2 = 0.0904
Final R indexes [all data]	R_1 = 0.0451, wR_2 = 0.1020
Largest diff. peak/hole / e Å^{-3}	0.21/-0.23
Flack parameter	-0.005(16)

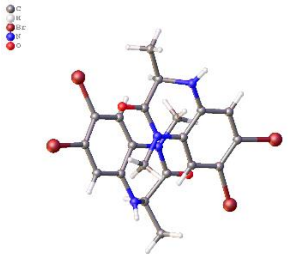

Figure S2. ORTEP drawing of compound **4h** (CCDC:2182123)

Table S3 Crystal data and structure refinement for 4h.

Identification code	N-9-ee_auto
Empirical formula	C20H20Br4N4O2
Formula weight	668.04
Temperature/K	300.00(10)

Crystal system	monoclinic
Space group	P21
a/Å	7.3314(2)
b/Å	12.7852(3)
c/Å	12.0154(2)
α/°	90
β/°	95.469(2)
γ/°	90
Volume/Å3	1121.12(4)
Z	2
ρcalcg/cm3	1.979
μ/mm-1	8.975
F(000)	648.0
Crystal size/mm3	1 × 0.5 × 0.5
Radiation	Cu Kα (λ = 1.54184)
2Θ range for data collection/°	7.392 to 153.974
Index ranges	-9 ≤ h ≤ 9, -6 ≤ k ≤ 15, -15 ≤ l ≤ 14
Reflections collected	7320
Independent reflections	3141 [Rint = 0.0355, Rsigma = 0.0395]
Data/restraints/parameters	3141/1/277
Goodness-of-fit on F2	1.195
Final R indexes [I>=2σ (I)]	R1 = 0.0364, wR2 = 0.1090
Final R indexes [all data]	R1 = 0.0471, wR2 = 0.1566
Largest diff. peak/hole / e Å-3	1.21/-1.14
Flack parameter	-0.04(7)

7. General procedure for asymmetric hydrogenation under continuous flow

All process parts, including fittings, tubes, valves and junctions that hold pressure were purchased from Shenzhen Yizheng Technology Co., LTD. The specification of the reaction coil is 0.5ml/m. The information of other main components is summarized in Table S3.

Table S4 Components details of reactor system

Name	Information
Pump	Sanotac high pressure HPLC pump AP0030（0-10 mL/min; 100 bar)
MFC	Beijing sevenstar flow CO. LTD, D08-1F, (0-100 sccm, 150 bar)
Relief check	X-tec Proportional ReliefValves RV-K2F (1000-1500 psi)
BPR	X-tec General-Purpose Back-PressureRegulators RBG (1000 psi)
Gas liquid separator	Shenzhen yizheng technology CO. LTD, (100 mL, 1000 psi)
Mixer	Shenzhen yizheng technology CO. LTD, (0.6 mL, 1000 psi)

In the nitrogen-filled glovebox, solution of $[Rh(cod)Cl]_2$ (5.6 mg, 0.0113 mmol) and

ligand (19.5mg, 0.0226 mmol) in 2.0 ml anhydrous DCM was stirred at room temperature for 30 min. Then the substrate **3j** (645mg, 2.26mmol) was dissolved in 45 mL anhydrous DCM and mixed with the above solution. The resulting mixture was filtered and the filtrate was added into a flask.

The process was washed by anhydrous DCM at a liquid flow rate of 5 mL/min and gas flow rate of 10 sccm (avoid back flow of liquid to gas flow meter) for 10 minutes and then pressurized the BPR. After the reactor was pressurized to 2 MPa, the aforehand reaction medium was pumped instead of solvent. Liquid flow rate was set at 0.4 mL/min and gas flow rate was keeping 10 sccm. The liquid holding capacity of the reaction coil can be adjusted according to the needs. The conversion and *ee* value were analyzed by NMR and HPLC. When reaction finished, system was depressurized by releasing the gas of Equilibar BPR slowly, and washed the whole system by pumping DCM for 10 minutes.

Table S5. The optimization of **3j** under continuous flow

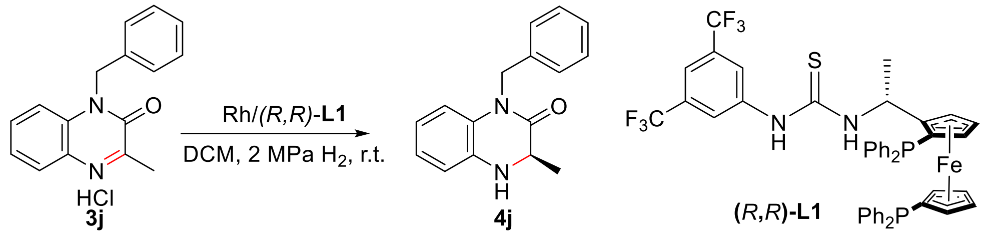

Conc (M)	Gas (sccm)	Liquid (mL/min)	H_2 (MPa)	Residence time (min)	Conversion[b]	*ee* (%)[b]
0.1	15	1	1	5.2	ND	--
0.1	15	0.5	1	7.5	ND	--
0.1	10	0.4	1.2	10.8	48%	--
0.1	5	0.4	1	14.5	50%	--
0.05	10	0.5	1	13	42%	--
0.05	10	0.5	2	13	45%	--
0.05	10	0.5	2	21	90%	--
0.05	10	1	2	30	75%	--
0.05	10	0.4	2	25	92%	98%
0.05	10	0.5	2	36	92%	98%
0.05[c]	10	0.5	2	25	ND	

Reaction conditions: DCM (30 mL); $[Rh(cod)Cl]_2$: 0.5% mol%; Ligand: 1%; S/C = 200; reactor volume: 30 mL; b. conversion and *ee* were determined by HPLC. c. S/C=1000

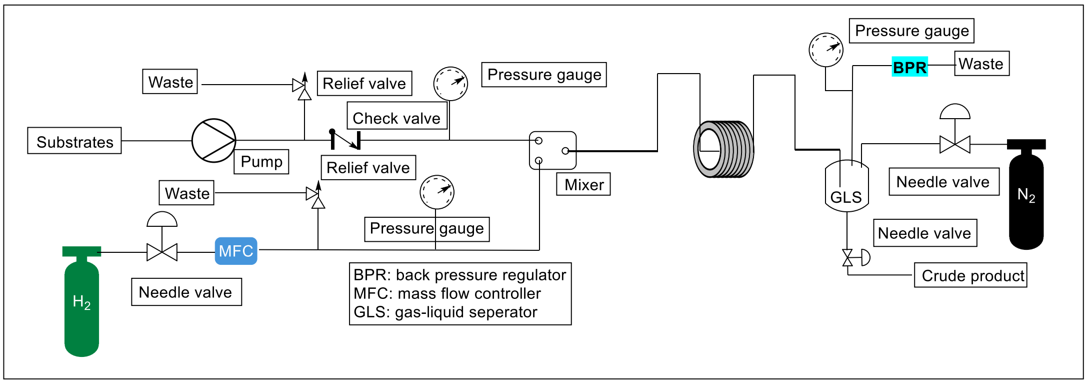

Scheme S1. Process scheme of **3j** under continuous flow.

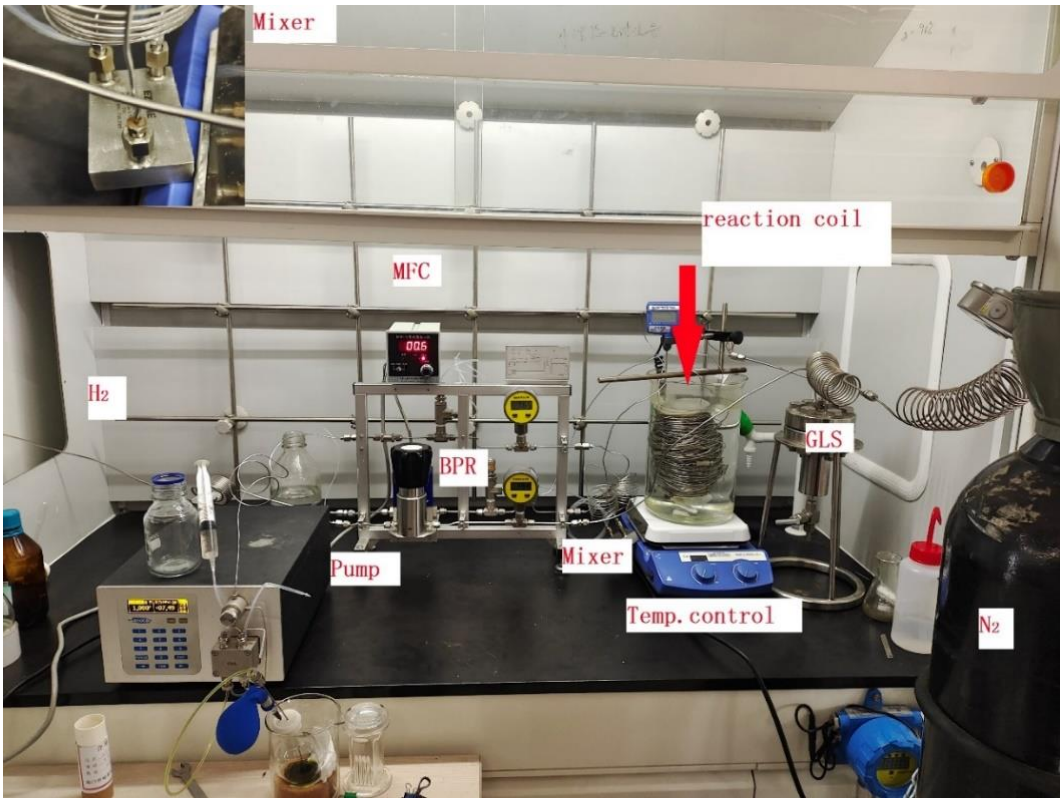

Figure S3. Set-up for asymmetric hydrogenation under continuous flow.

8. The influence of H^+ and Cl^- respectively

In the nitrogen-filled glovebox, solution of $[Rh(cod)Cl]_2$ (1.25 mg, 0.0025 mmol) and ligand (4.4 mg, 0.005 mmol) in 2.0 ml anhydrous solvent was stirred at room temperature for 30 min. A specified volume of the resulting solution (1 ml, 0.5% Rh catalyst) was transferred by syringe to a Score-Break ampule charged with substrate solution (0.25 mmol in 0.5 ml). Corresponding additives are added and then the ampule was placed into an autoclave, which was then charged with 1 MPa H_2. The autoclave was stirred at room temperature for the indicated period of time. After release of H_2, the resulting mixture was concentrated under vacuum. Saturated potassium carbonate solution and dichloromethane was added and the mixture was stirred for 30 min. The organic layer was dried with anhydrous sodium sulfate. After

removal of solvent, the crude product was analyzed by ^{1}H NMR to determine the conversion. The enantiomeric excess was determined by HPLC analysis of the crude product.

This result indicated that the HCl salt is crucial for *ee*s and yields (Table S6, entries 1-3). Moreover, in order to obtain more insights into H^+ and Cl^- role for *ee*s and yields respectively, detailed more additional experiments were also conducted. It was observed that the amount of HCl had an obvious influence on *ee*s and yields (Table S6, entries 1-4) and the existence of Cl^- was crucial for *ees* value while H^+ had a larger impact on the conversion of the starting materials (Table S6, entries 5-6). When the catalytic precursor was replaced by $Rh(cod)_2BF_4$ without Cl^-, the reaction did not occur (Table S6, entry 4[a]). Further observation revealed that the reaction rate was controlled by both Cl^- and H^+(Table S6, entries 7-10). These results were consistent with our DFT calculations (Figure S8), In comparison to TS4OR, much weaker Cl…H interactions showed in TS4OS. Therefore, Cl- could significantly improve the ee value of the products and facilitated the heterolytic cleavage of dihydrogen in the rate-determining step.

Table S6. The influence of H^+ and Cl^- respectively

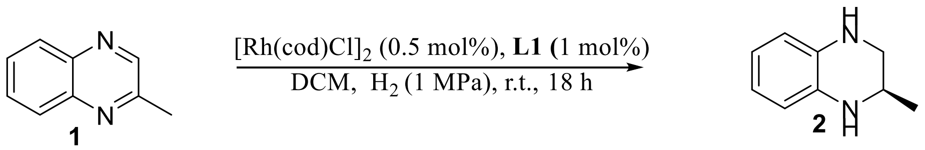

Entry	additive	Additive(eq)	*Conv* (%)	*ee* (%)
1	HCl	1	99	95
2	HCl	0.2	59	92
3	-	-	48	63
4[a]	-	-	0	0
5	KCl	1	27	91
6	KCl	0.2	30	89
7	$CF_3CSOOOH$	1	70	60
8	$CF_3CSOOOH$	0.2	60	55
9	CF_3COOH	1	60	70
10	CF_3COOH	0.2	70	27

Reaction conditions: **1** (0.25 mmol) in 4 mL dry DCM, **1**/$[Rh(cod)Cl]_2$/ **L1** ratio = 100/0.5/1; Additive (0.2 or 1 eq.); [a]Catalytic precursor is $Rh(cod)_2BF_4$; yield was determined with isolated products; *ee* was determined by HPLC.

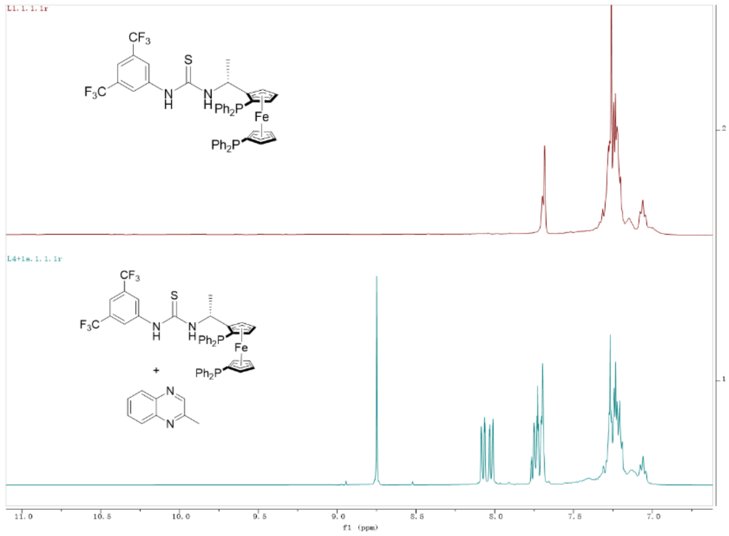

Figure S4. ^1H NMR study of interaction of Ligand (0.03 M) with 2-Methylquinoxaline (0.09 M) in $CDCl_3$.

9. linear Effect Experiment

In the nitrogen-filled glovebox, solution of $[Rh(cod)Cl]_2$ (3.6 mg, 0.0073 mmol) and (*R*)-**L1** (13.2 mg, 0.015 mmol) in 6.0 ml anhydrous solvent ($[Rh(cod)Cl]_2$ (1.25 mg, 0.0025 mmol) and (*S*)-**L1** (5 mg, 0.0057 mmol) in 2.0 ml anhydrous solvent) was stirred at room temperature for 30 min. A specified volume of the resulting solution (1 mL, 0.5% Rh catalyst) was transferred by syringe to a Score-Break ampule charged with substrate solution (0.25 mmol in 0.5 ml) as table S7. The ampule was placed into an autoclave, which was then charged with 1 MPa H_2. The autoclave was stirred at room temperature for the indicated period of time. After release of H_2, the resulting mixture was concentrated under vacuum. Saturated potassium carbonate solution and dichloromethane was added and the mixture was stirred for 30 min. The organic layer was dried with anhydrous sodium sulfate. After removal of solvent, the crude product was analysed by ^1H NMR to determine the conversion. The enantiomeric excess was determined by HPLC analysis of the crude product.

Table S7. Data for linear Effect Experiment

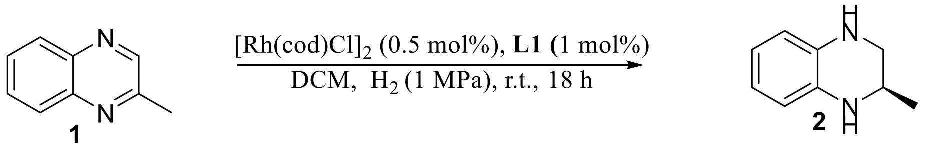

Entry	ee_{cat} (%)	(*R*)$_{cat}$-stock solution (μL)	(S)$_{cat}$-stock solution (μL)	*ee* (%)[a]
1	100	1000	0	94
2	80	900	100	78
3	60	800	200	61
4	50	750	250	50
5	40	700	300	42
6	0	0	1000	7

The preparations of **L1** are assumed to be enantiomerically pure. [a]Determined by HPLC analysis.

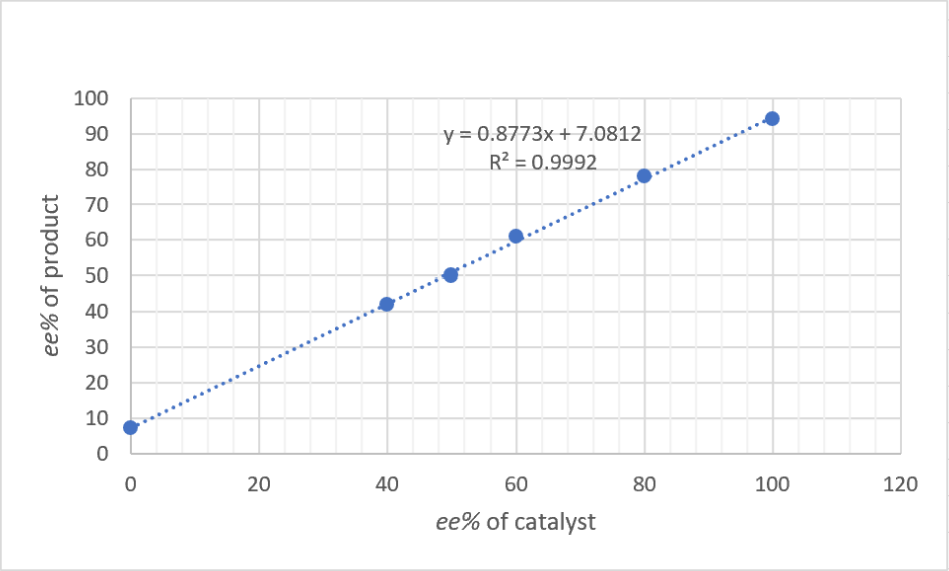

Figure S5. Illustration of the linear relationship between the *ee* of product and the *ee* of catalyst.

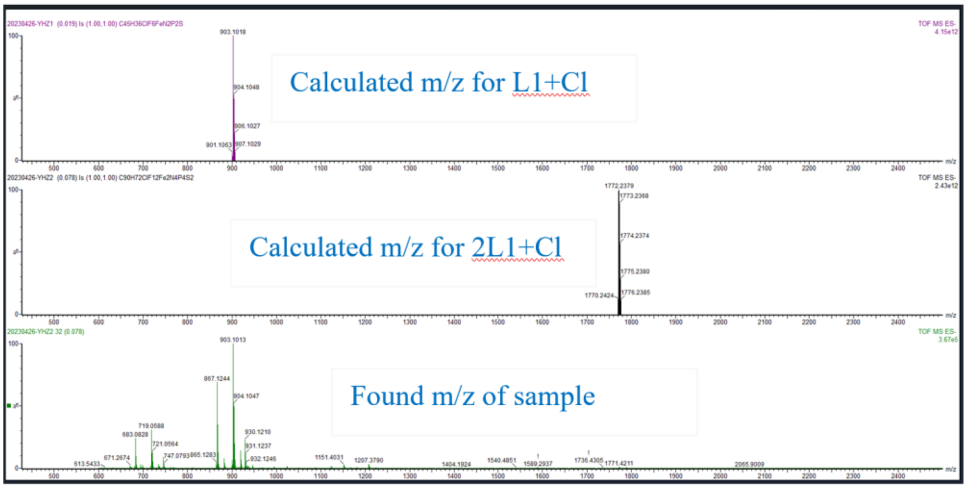

High-resolution mass spectroscopy (m/z; 500-2000) of solution of L1 (1 eq.) and TBAC (3 eq.).

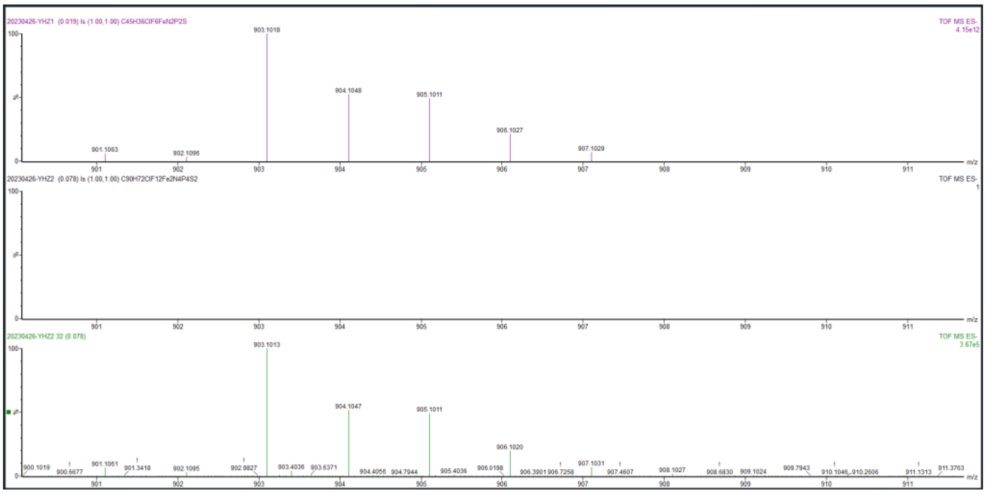

Figure S6. High-resolution mass spectroscopy (m/z; 901-911) of solution of **L1** (1 eq.) and TBAC (3 eq.).

10. Characterization data of chiral Tetrahydroquinoxalines and Dihydroquinoxalinones

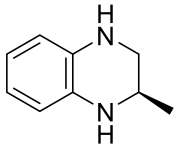

(*R*)-2-methyl-1,2,3,4-tetrahydroquinoxaline(2a)

Yellow solid. ^{1}H NMR (400 MHz, Chloroform-d) δ 6.63 (dt, J = 7.3, 3.6 Hz, 2H), 6.55 (dq, J = 6.4, 3.6 Hz, 2H), 3.55 (dq, J = 11.5, 5.3, 4.0 Hz, 1H), 3.43 (s, 2H), 3.36 (dd, J = 10.8, 3.0 Hz, 1H), 3.08 (dd, J = 10.7, 8.2 Hz, 1H), 1.23 (d, J = 6.3 Hz, 3H)。 ^{13}C NMR (101 MHz, $CDCl_3$) δ 133.65, 133.26, 118.78, 114.52, 48.31, 45.78, 19.98.

$[\alpha]_D^{25}$ = +11.3 (*c* 0.1, $CHCl_3$).

HRMS (ESI) calcd for $C_9H_{13}N_2^+$ $[(M+H)^+]$ 149.1073, found 149.1070.

Daicel Chiralpak OD-H column, isocratic elution: n-hexane/2-propanol 80:20, flow rate 0.5 mL/min, column temp 40 °C, retention times 15.240 min (major enantiomer), 18.470 min (minor enantiomer), 94% *ee*.[7]

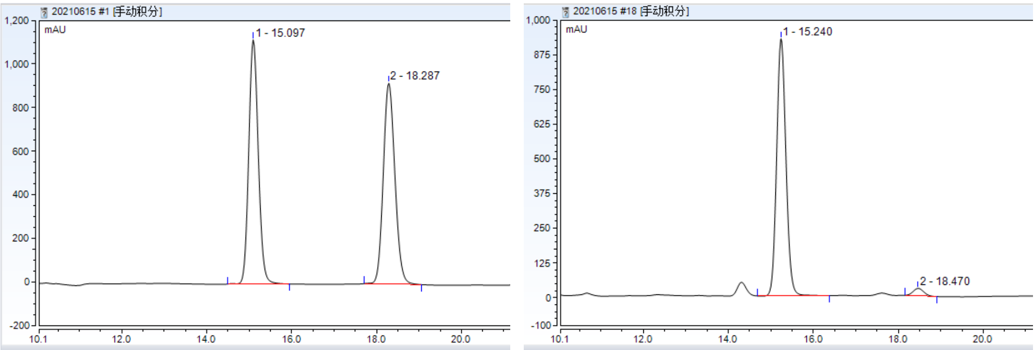

Peak #	RetTime min	Area %	Area mAU*min	Height mAU	Width (50%) min
1	15.097	49.98	292.0190	1121.30	0.239
2	18.287	50.02	292.1980	921.94	0.294

Peak #	RetTime min	Area %	Area mAU*min	Height mAU	Width (50%) min
1	15.240	96.85	234.5271	926.17	0.233
2	18.470	3.15	7.6231	26.70	0.271

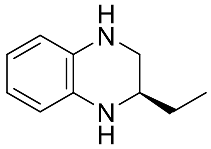

(*R*)-2-ethyl-1,2,3,4-tetrahydroquinoxaline(2b)

Yellow solid. ^{1}H NMR (400 MHz, Chloroform-*d*) δ 6.66 – 6.58 (m, 2H), 6.55 (dt, *J* = 4.7, 3.0 Hz, 2H), 3.54 – 3.24 (m, 3H), 3.10 (dd, *J* = 10.6, 7.8 Hz, 1H), 1.56 (p, *J* = 7.3 Hz, 2H), 1.06 (d, *J* = 7.5 Hz, 3H). ^{13}C NMR (101 MHz, Chloroform-*d*) δ 133.61, 133.50, 118.82, 118.64, 114.49, 51.78, 46.34, 27.20, 10.11.

$[\alpha]_D^{25}$ = +39.0 (*c* 0.1, $CHCl_3$).

HRMS (ESI) calcd for $C_{10}H_{15}N_2^+$ $[(M+H)^+]$ 163.1230, found 163.1232.

Daicel Chiralpak OD-H column, isocratic elution: n-hexane/2-propanol 80:20, flow rate 0.5 mL/min, column temp 40 °C, retention times 15.750 min (major enantiomer), 19.040 min (minor enantiomer), 91% *ee*.

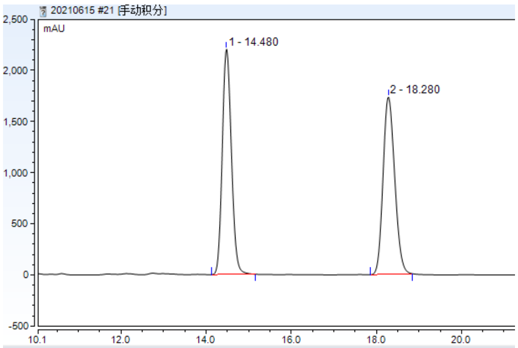

	A	B	C	D	E	F
1	Peak	RetTime	Area	Area	Height	Width (50%)
2	#	min	%	mAU*min	mAU	min
4	1	14.480	49.95	548.9646	2205.20	0.231
5	2	18.280	50.05	550.1722	1737.42	0.296

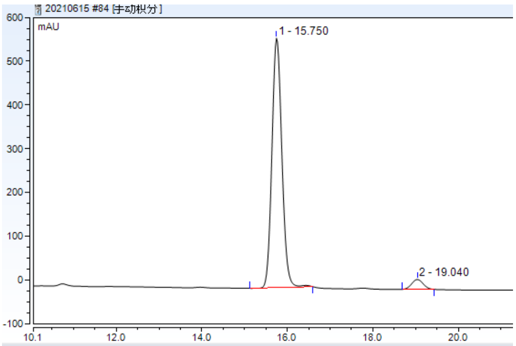

	A	B	C	D	E	F
1	Peak	RetTime	Area	Area	Height	Width (50%)
2	#	min	%	mAU*min	mAU	min
4	1	15.750	95.59	153.8499	570.94	0.248
5	2	19.040	4.41	7.0903	22.78	0.295

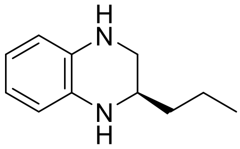

(*R*)-2-propyl-1,2,3,4-tetrahydroquinoxaline (2c)

Yellow solid. ^{1}H NMR (400 MHz, Chloroform-*d*) δ 6.60 – 6.52 (m, 2H), 6.47 (dd, *J* = 5.9, 3.3 Hz, 2H), 3.50 (s, 2H), 3.35 – 3.28 (m, 2H), 3.02 (dd, *J* = 11.1, 8.2 Hz, 1H), 1.46 – 1.31 (m, 4H), 0.99 – 0.92 (m, 3H).^{13}C NMR (101 MHz, Chloroform-*d*) δ 133.65, 133.57, 118.79, 118.67, 114.58, 114.52, 50.07, 46.76, 36.54, 18.95, 14.28.

$[\alpha]_D^{25}$ = +39.1 (*c* 0.1, $CHCl_3$).

HRMS (ESI) calcd for $C_{11}H_{17}N_2^+$ [(M+H)$^+$] 217.1699, found 217.1694.

Daicel Chiralpak OD-H column, isocratic elution: n-hexane/2-propanol 80:20, flow rate 0.5 mL/min, column temp 40 °C， retention times 14.817 min (major enantiomer), 18.130 min (minor enantiomer), 95% *ee*.

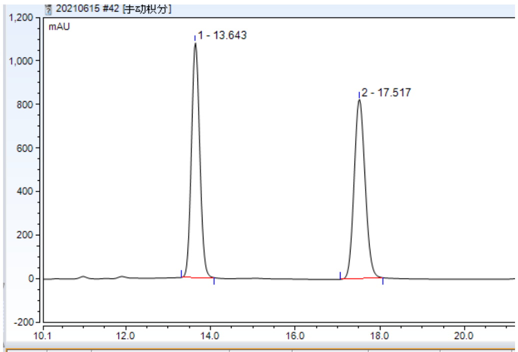

	A	B	C	D	E	F
1	Peak	RetTime	Area	Area	Height	Width (50%)
2	#	min	%	mAU*min	mAU	min
4	1	13.643	49.80	245.5421	1078.54	0.212
5	2	17.517	50.20	247.5039	823.78	0.280

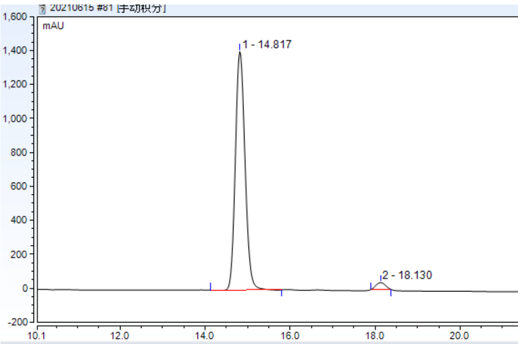

	A	B	C	D	E	F
1	Peak	RetTime	Area	Area	Height	Width (50%)
2	#	min	%	mAU*min	mAU	min
4	1	14.817	97.30	369.2388	1407.10	0.243
5	2	18.130	2.70	10.2409	40.35	0.253

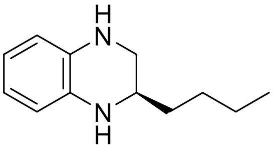

(*R*)-2-butyl-1,2,3,4-tetrahydroquinoxaline （2d）

Yellow solid. ^{1}H NMR (400 MHz, Chloroform-*d*) δ 6.59 (q, *J* = 4.7 Hz, 2H), 6.54 – 6.48 (m, 2H), 3.36 (dt, *J* = 9.2, 3.3 Hz, 2H), 3.07 (dd, *J* = 11.1, 8.2 Hz, 1H), 1.57 – 1.31 (m, 6H), 0.98 – 0.88 (m, 3H). ^{13}C NMR (101 MHz, Chloroform-*d*) δ 133.60, 133.47, 118.84, 118.68, 114.57, 114.53, 50.32, 46.74, 34.07, 27.89, 22.87, 14.12.

$[\alpha]_D^{25}$ = +35.0 (*c* 0.1, $CHCl_3$).

HRMS (ESI) calcd for $C_{12}H_{19}N_2^+$ $[(M+H)^+]$ 191.1543, found 191.1546.

Daicel Chiralpak OD-H column, isocratic elution: n-hexane/2-propanol 80:20, flow rate 0.5 mL/min, column temp 40 °C, retention times 13.187 min (major enantiomer), 16.650 min (minor enantiomer), 91% *ee*.

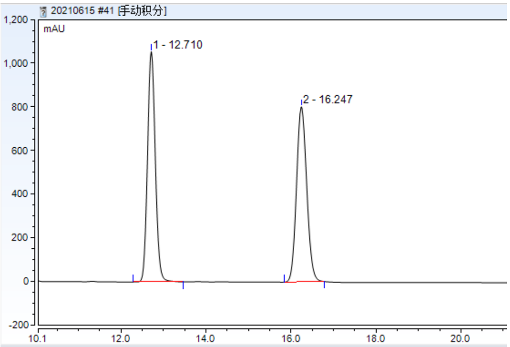

	A	B	C	D	E	F
1	Peak	RetTime	Area	Area	Height	Width (50%)
2	#	min	%	mAU*min	mAU	min
4	1	12.710	50.08	224.0757	1055.31	0.196
5	2	16.247	49.92	223.3799	802.77	0.259

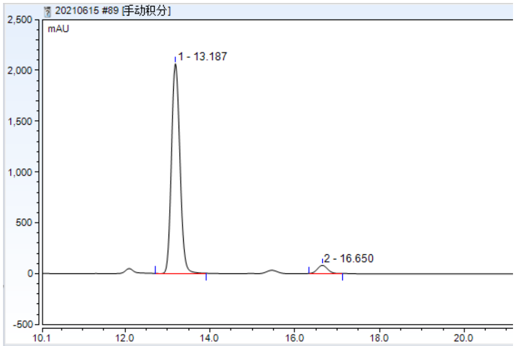

	A	B	C	D	E	F
1	Peak	RetTime	Area	Area	Height	Width (50%)
2	#	min	%	mAU*min	mAU	min
4	1	13.187	95.62	476.8279	2063.41	0.215
5	2	16.650	4.38	21.8295	79.89	0.259

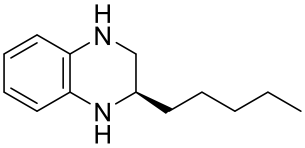

(*R*)-2-pentyl-1,2,3,4-tetrahydroquinoxaline (2e)

Yellow solid. ^{1}H NMR (400 MHz, Chloroform-*d*) δ 6.59 (q, *J* = 4.7 Hz, 2H), 6.54 – 6.48 (m, 2H), 3.55 – 3.19 (m, 2H), 3.07 (dd, *J* = 11.2, 8.3 Hz, 1H), 1.54 – 1.25 (m, 8H), 0.96 – 0.88 (m, 3H). ^{13}C NMR (101 MHz, Chloroform-*d*) δ 133.63, 133.52, 118.80, 118.66, 114.55, 114.50, 50.33, 46.76, 34.34, 31.98, 25.41, 22.69, 14.14.

$[\alpha]_D^{25}$ = +40.2 (*c* 0.1, $CHCl_3$).

HRMS (ESI) calcd for $C_{13}H_{21}N_2^+$ $[(M+H)^+]$ 205.1699 , found 205.1697.

Daicel Chiralpak OD-H column, isocratic elution: n-hexane/2-propanol 80:20, flow rate 0.5 mL/min, column temp 40 °C, retention times 12.783 min (major enantiomer), 16.117 min (minor enantiomer), 93% *ee*.

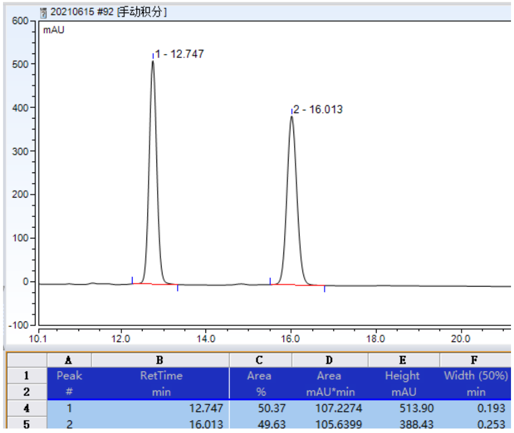

	Peak #	RetTime min	Area %	Area mAU*min	Height mAU	Width (50%) min
4	1	12.747	50.37	107.2274	513.90	0.193
5	2	16.013	49.63	105.6399	388.43	0.253

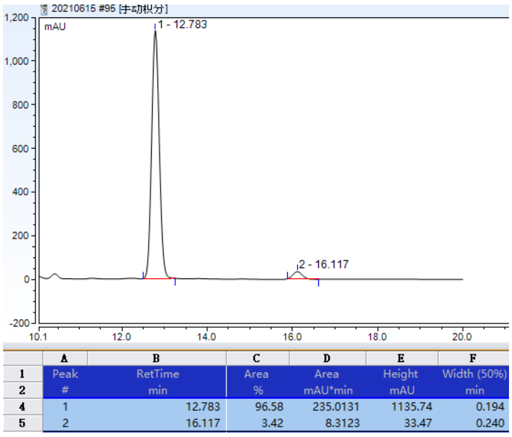

	Peak #	RetTime min	Area %	Area mAU*min	Height mAU	Width (50%) min
4	1	12.783	96.58	235.0131	1135.74	0.194
5	2	16.117	3.42	8.3123	33.47	0.240

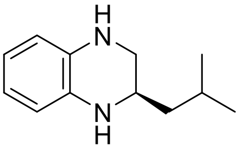

(*R*)-2-isobutyl-1,2,3,4-tetrahydroquinoxaline (2f)

Yellow solid. ^{1}H NMR (400 MHz, Chloroform-*d*) δ 6.63 (dt, *J* = 7.5, 3.7 Hz, 2H), 6.59 – 6.49 (m, 2H), 3.62 – 3.28 (m, 4H), 3.09 (dd, *J* = 10.7, 7.8 Hz, 1H), 1.80 (dp, *J* = 13.5, 6.7 Hz, 1H), 1.49 – 1.29 (m, 2H), 1.01 (dd, *J* = 6.6, 2.7 Hz, 6H). ^{13}C NMR (101 MHz, Chloroform-*d*) δ 133.58, 133.51, 118.81, 118.73, 114.64, 114.53, 48.18, 47.09, 43.40, 24.53, 23.24, 22.59.

$[\alpha]_D^{25}$ = +43.7 (*c* 0.1, $CHCl_3$).

HRMS (ESI) calcd for $C_{12}H_{19}N_2^+$ [(M+H)$^+$] 191.1543 , found 191.1543.

Daicel Chiralpak OD-H column, isocratic elution: n-hexane/2-propanol 80:20, flow rate 0.5 mL/min, column temp 40 °C， retention times 13.540 min (major enantiomer), 18.190 min (minor enantiomer), 90% *ee*.

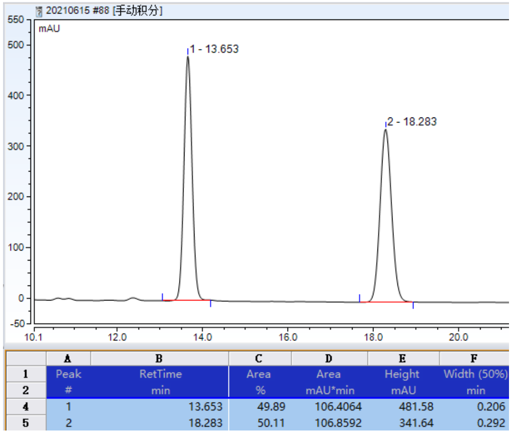

	Peak #	RetTime min	Area %	Area mAU*min	Height mAU	Width (50%) min
4	1	13.653	49.89	106.4064	481.58	0.206
5	2	18.283	50.11	106.8592	341.64	0.292

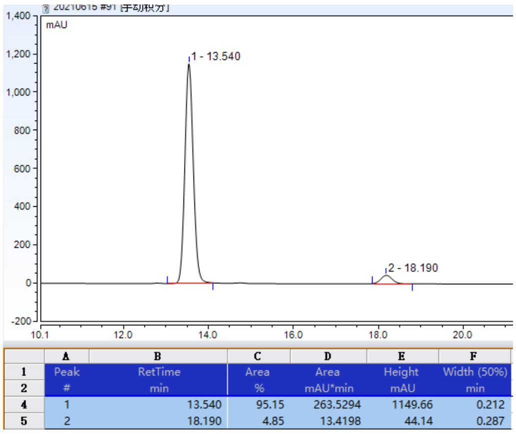

	Peak #	RetTime min	Area %	Area mAU*min	Height mAU	Width (50%) min
4	1	13.540	95.15	263.5294	1149.66	0.212
5	2	18.190	4.85	13.4198	44.14	0.287

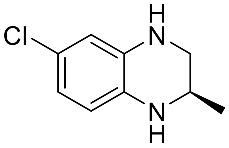

(*R*)-6-chloro-2-methyl-1,2,3,4-tetrahydroquinoxaline （2g）

White solid. ^{1}H NMR (400 MHz, Chloroform-*d*) δ 6.55 (dd, *J* = 8.3, 2.3 Hz, 1H), 6.49 (d, *J* = 2.3 Hz, 1H), 6.42 (d, *J* = 8.2 Hz, 1H), 3.49 (dqd, *J* = 8.9, 6.3, 2.8 Hz, 1H), 3.34 (dd, *J* = 10.8, 3.0 Hz, 1H), 3.04 (dd, *J* = 10.8, 8.1 Hz, 1H), 1.21 (d, *J* = 6.3 Hz, 3H). ^{13}C NMR (101 MHz, Chloroform-*d*) δ 134.34, 132.02, 123.16, 117.93, 115.05, 113.72, 47.92, 45.52, 19.80.

$[\alpha]_D^{25}$ = +32.7 (*c* 0.1, $CHCl_3$).

HRMS (ESI) calcd for $C_9H_{12}ClN_2^+$ [(M+H)$^+$] 183.0684 , found 183.0680.

Daicel Chiralpak OD-H column, isocratic elution: n-hexane/2-propanol 90:10, flow rate 1 mL/min, column temp 40 °C, retention times 11.077 min (major enantiomer), 14.627 min (minor enantiomer), 97% *ee*.

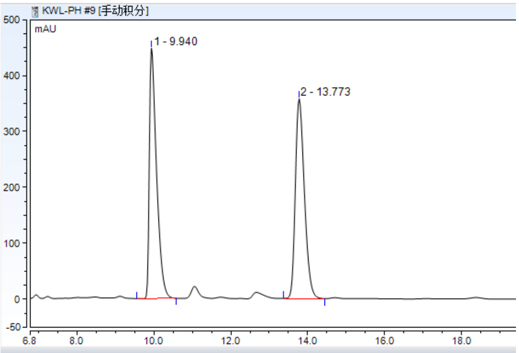

	A	B	C	D	E	F
1	Peak	RetTime	Area	Area	Height	Width (50%)
2	#	min	%	mAU*min	mAU	min
4	1	9.940	49.90	96.7001	448.11	0.194
5	2	13.773	50.10	97.1046	357.76	0.250

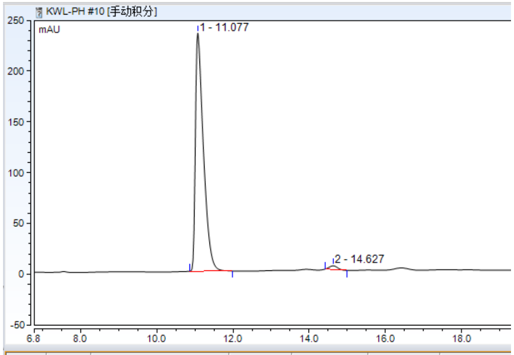

	A	B	C	D	E	F
1	Peak	RetTime	Area	Area	Height	Width (50%)
2	#	min	%	mAU*min	mAU	min
4	1	11.077	98.45	57.1271	234.58	0.220
5	2	14.627	1.55	0.8986	3.69	0.241

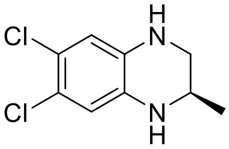

(*R*)-6,7-dichloro-2-methyl-1,2,3,4-tetrahydroquinoxaline （2h）

White solid. ^{1}H NMR (400 MHz, Chloroform-*d*) δ 6.54 (d, *J* = 3.6 Hz, 2H), 3.73 (s, 2H), 3.49 (dtd, *J* = 12.4, 6.3, 2.9 Hz, 1H), 3.33 (dd, *J* = 10.8, 3.0 Hz, 1H), 3.02 (dd, *J* = 10.8, 8.0 Hz, 1H), 1.21 (d, *J* = 6.3 Hz, 3H). ^{13}C NMR (101 MHz, Chloroform-*d*) δ 133.23, 132.92, 120.37, 120.30, 114.73, 114.62, 47.62, 45.40, 19.73.

$[\alpha]_D^{25}$ = +43.3 (*c* 0.1, $CHCl_3$).

HRMS (ESI) calcd for $C_9H_{12}ClN_2^+$ [(M+H)$^+$] 217.0294, found 217.0293.

Daicel Chiralpak OD-H column, isocratic elution: n-hexane/2-propanol 90:10, flow rate 1 mL/min, column temp 40 °C, retention times 11.533 min (major enantiomer), 12.073 min (minor enantiomer), 95% *ee*.

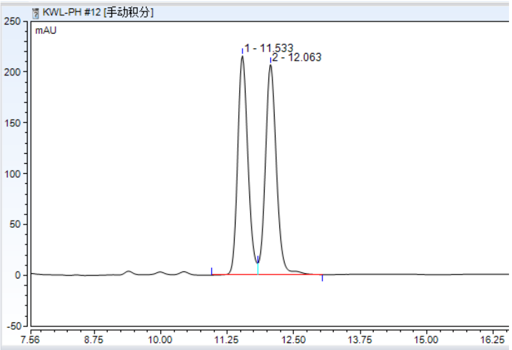

	A	B	C	D	E	F
1	Peak	RetTime	Area	Area	Height	Width (50%)
2	#	min	%	mAU*min	mAU	min
4	1	11.533	49.27	48.0837	215.81	0.205
5	2	12.063	50.73	49.5169	206.82	0.215

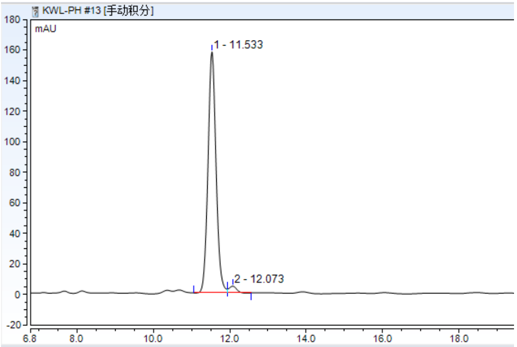

	A	B	C	D	E	F
1	Peak	RetTime	Area	Area	Height	Width (50%)
2	#	min	%	mAU*min	mAU	min
4	1	11.533	97.50	38.5235	157.80	0.222
5	2	12.073	2.50	0.9887	4.45	n.a.

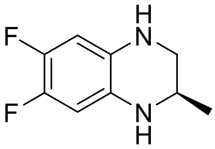

(*R*)-6,7-difluoro-2-methyl-1,2,3,4-tetrahydroquinoxaline (2i)

White solid. ^{1}H NMR (400 MHz, Chloroform-*d*) δ 6.32 (ddd, *J* = 11.5, 8.3, 3.7 Hz, 2H), 3.46 – 3.41 (m, 2H), 3.31 (dd, *J* = 10.9, 3.0 Hz, 1H), 2.99 (dd, *J* = 10.9, 8.1 Hz, 1H), 1.20 (d, *J* = 6.3 Hz, 3H).^{13}C NMR (101 MHz, Chloroform-*d*) δ 144.35 (d, *J* = 15.6 Hz), 141.99 (d, *J* = 15.4 Hz), 129.36 (dd, *J* = 6.8, 3.3 Hz), 128.99 (dd, *J* = 7.0, 3.3 Hz), 102.92 (t, *J* = 4.9 Hz), 102.82 – 102.62 (m), 47.95, 45.61, 19.67.

$[\alpha]_D^{25}$ = +44.7 (*c* 0.1, $CHCl_3$).

HRMS (ESI) calcd for $C_9H_{11}F_2N_2^+$ [$(M+H)^+$] 185.0885, found 185.0881.

Daicel Chiralpak AD-H column, isocratic elution: n-hexane/2-propanol 90:10, flow rate 1 mL/min, column temp 40 °C, retention times 11.977 min (major enantiomer), 13.203 min (minor enantiomer), 96% *ee*.

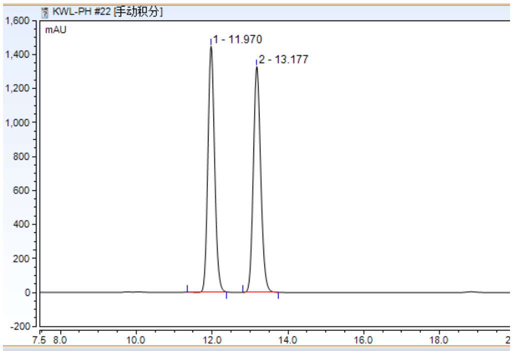

	A	B	C	D	E	F
1	Peak	RetTime	Area	Area	Height	Width (50%)
2	#	min	%	mAU*min	mAU	min
4	1	11.970	49.62	295.9617	1446.72	0.189
5	2	13.177	50.38	300.5014	1329.38	0.208

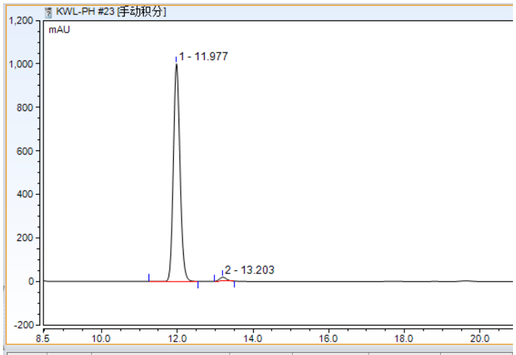

	A	B	C	D	E	F
1	Peak	RetTime	Area	Area	Height	Width (50%)
2	#	min	%	mAU*min	mAU	min
4	1	11.977	98.10	202.3947	1000.24	0.186
5	2	13.203	1.90	3.9294	18.63	0.198

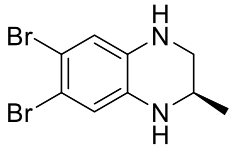

(*R*)-6,7-dibromo-2-methyl-1,2,3,4-tetrahydroquinoxaline (2j)

Yellow solid. ^{1}H NMR (400 MHz, Chloroform-*d*) δ 6.66 (d, *J* = 2.3 Hz, 2H), 3.64 (s, 2H), 3.43 (pd, *J* = 6.3, 2.8 Hz, 1H), 3.28 (dd, *J* = 10.8, 3.0 Hz, 1H), 2.95 (dd, *J* = 10.8, 8.0 Hz, 1H), 1.16 (d, *J* = 6.3 Hz, 3H). ^{13}C NMR (101 MHz, Chloroform-*d*) δ 134.03, 133.73, 117.61, 117.48, 111.31, 111.22, 47.49, 45.28, 19.70.

$[\alpha]_D^{25}$ = +27.7 (*c* 0.1, $CHCl_3$).

HRMS (ESI) calcd for $C_9H_{11}Br_2N_2^+$ [(M+H)$^+$] 304.9284, found 304.9283.

Daicel Chiralpak OD-H column, isocratic elution: n-hexane/2-propanol 90:10, flow rate 1 mL/min, column temp 40 °C, retention times 14.497 min (major enantiomer), 16.010 min (minor enantiomer), 97% *ee*.

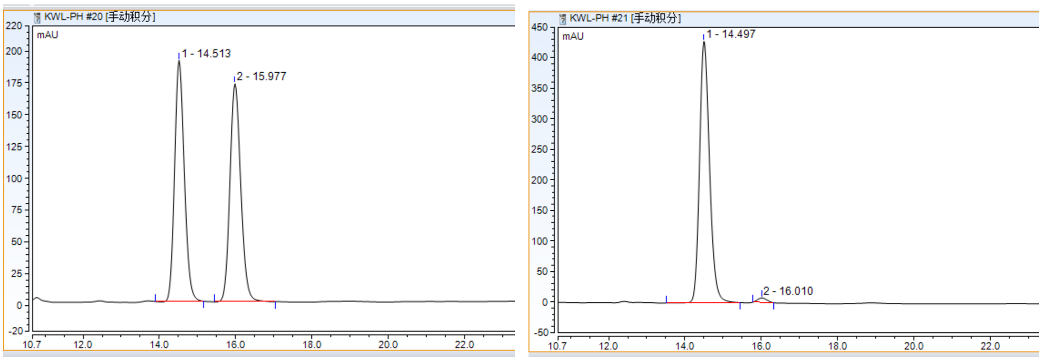

Peak #	RetTime min	Area %	Area mAU*min	Height mAU	Width (50%) min
1	14.513	49.68	55.6730	189.51	0.271
2	15.977	50.32	56.3813	171.36	0.302

Peak #	RetTime min	Area %	Area mAU*min	Height mAU	Width (50%) min
1	14.497	98.49	129.6300	428.01	0.276
2	16.010	1.51	1.9902	7.16	0.273

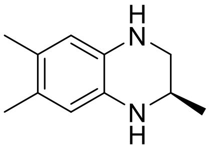

(*R*)-2,6,7-trimethyl-1,2,3,4-tetrahydroquinoxaline (2k)

Yellow solid. ^{1}H NMR (400 MHz, Chloroform-*d*) δ 6.35 (d, *J* = 4.7 Hz, 2H), 3.50 – 3.45 (m, 1H), 3.32 – 3.25 (m, 3H), 3.00 (t, *J* = 9.4 Hz, 1H), 2.11 (s, 6H), 1.18 (d, *J* = 6.3 Hz, 3H). ^{13}C NMR (101 MHz, Chloroform-*d*) δ 131.50, 131.04, 126.64, 126.55, 116.48, 48.67, 46.14, 19.97, 18.98.

$[\alpha]_D^{25}$ = +13.2 (*c* 0.1, $CHCl_3$).

HRMS (ESI) calcd for $C_{11}H_{17}N_2^+$ [(M+H)$^+$] 177.1386, found 177.1382.

Daicel Chiralpak OD-H column, isocratic elution: n-hexane/2-propanol 90:10, flow rate 0.8 mL/min, column temp 40 °C, retention times 11.753 min (major enantiomer), 15.973 min (minor enantiomer), 85% *ee*.

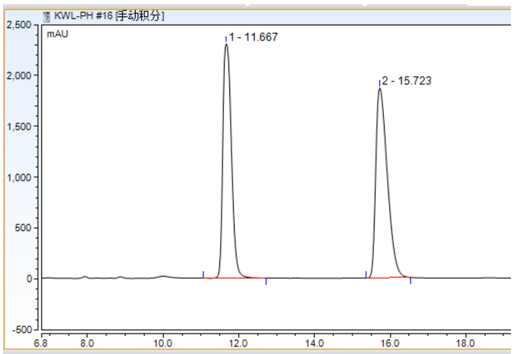

	A	B	C	D	E	F
1	Peak	RetTime	Area	Area	Height	Width (50%)
2	#	min	%	mAU*min	mAU	min
4	1	11.667	47.80	602.8204	2304.26	0.245
5	2	15.723	52.20	658.3218	1869.16	0.330

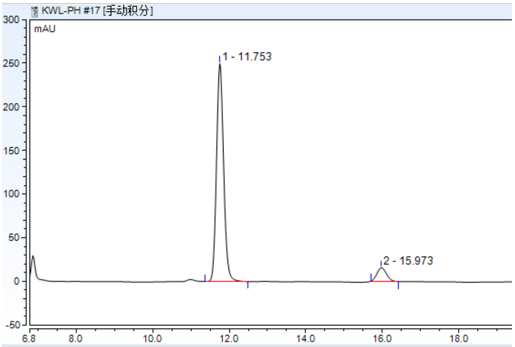

	A	B	C	D	E	F
1	Peak	RetTime	Area	Area	Height	Width (50%)
2	#	min	%	mAU*min	mAU	min
4	1	11.753	92.29	53.5577	249.71	0.196
5	2	15.973	7.71	4.4762	15.58	0.273

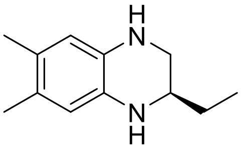

(*R*)-2-ethyl-6,7-dimethyl-1,2,3,4-tetrahydroquinoxaline (2l)

Gray solid. ^{1}H NMR (400 MHz, Chloroform-*d*) δ 6.37 (s, 2H), 3.38 (dd, *J* = 10.7, 2.8 Hz, 1H), 3.27 (qd, *J* = 6.7, 2.7 Hz, 1H), 3.06 (dd, *J* = 10.6, 7.7 Hz, 1H), 2.13 (s, 6H), 1.54 (p, *J* = 7.3 Hz, 2H), 1.03 (t, *J* = 7.5 Hz, 3H). ^{13}C NMR (101 MHz, Chloroform-*d*) δ 131.45, 131.27, 126.65, 126.37, 116.41, 116.36, 52.14, 46.69, 27.13, 18.94, 10.15.

$[\alpha]_D^{25}$ = +15.345 (*c* 0.1, $CHCl_3$).

HRMS (ESI) calcd for $C_{12}H_{19}N_2^+$ [$(M+H)^+$] 191.1543, found 191.1540.

Daicel Chiralpak OD-H column, isocratic elution: n-hexane/2-propanol 90:10, flow rate 0.8 mL/min, column temp 40 °C, retention times 11.753 min (major enantiomer), 15.973 min (minor enantiomer), 79% *ee*.

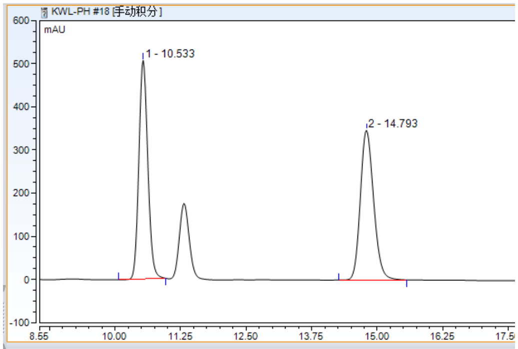

	A	B	C	D	E	F
1	Peak	RetTime	Area	Area	Height	Width (50%)
2	#	min	%	mAU*min	mAU	min
4	1	10.533	49.56	100.0305	506.50	0.182
5	2	14.793	50.44	101.8204	346.80	0.270

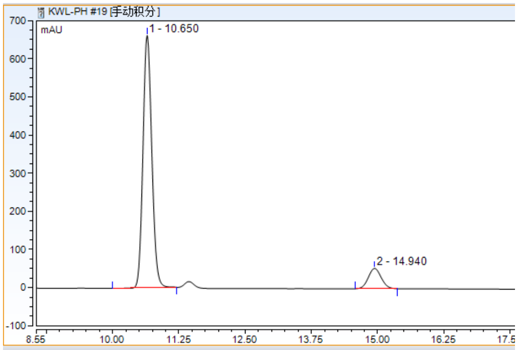

	A	B	C	D	E	F
1	Peak	RetTime	Area	Area	Height	Width (50%)
2	#	min	%	mAU*min	mAU	min
4	1	10.650	89.47	127.5901	661.57	0.178
5	2	14.940	10.53	15.0110	53.29	0.264

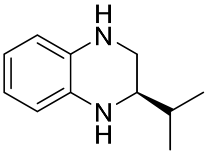

(*R*)-2-isopropyl-1,2,3,4-tetrahydroquinoxaline (2m)

White solid. ^{1}H NMR (400 MHz, Chloroform-*d*) δ 6.59 (ddt, *J* = 30.3, 6.5, 2.6 Hz, 4H), 3.41 (dd, *J* = 10.2, 2.4 Hz, 1H), 3.24 – 3.12 (m, 2H), 1.77 (dp, *J* = 15.0, 6.6 Hz, 1H), 1.05 (dd, *J* = 18.9, 6.8 Hz, 6H). ^{13}C NMR (101 MHz, Chloroform-*d*) δ 133.65, 133.21, 118.58, 118.17, 114.13, 114.10, 55.80, 43.85, 18.50, 18.36.

$[\alpha]_D^{25}$ = +18.342 (*c* 0.1, $CHCl_3$).

HRMS (ESI) calcd for $C_{11}H_{17}N_2^+$ [$(M+H)^+$] 177.1387, found 177.1382.

Daicel Chiralpak OD-H column, isocratic elution: n-hexane/2-propanol 80:20, flow rate 0.5 mL/min, column temp 40 °C, retention times 13.343 min (major enantiomer), 17.623 min (minor enantiomer), 31% *ee*.

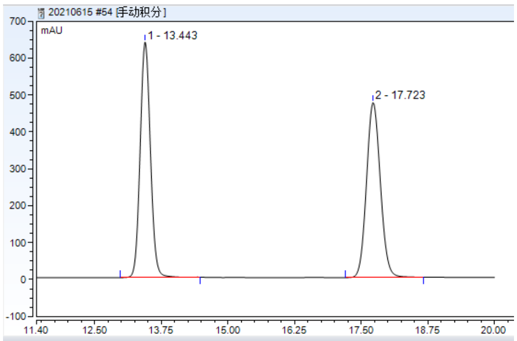

Peak #	RetTime min	Area %	Area mAU*min	Height mAU	Width (50%) min
1	13.443	49.91	142.1308	637.62	0.206
2	17.723	50.09	142.6468	474.25	0.279

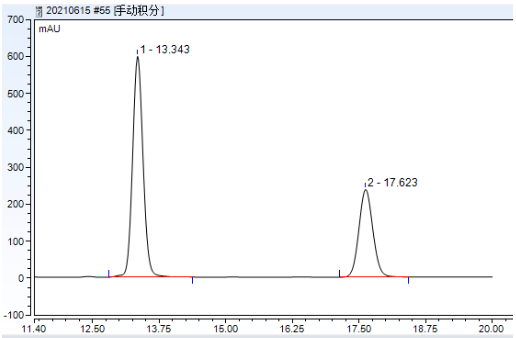

Peak #	RetTime min	Area %	Area mAU*min	Height mAU	Width (50%) min
1	13.343	65.55	136.5877	597.25	0.209
2	17.623	34.45	71.7943	237.19	0.280

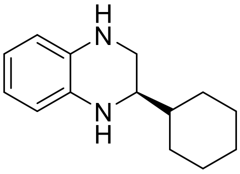

(*R*)-2-cyclohexyl-1,2,3,4-tetrahydroquinoxaline (2n)

Yellow solid. ^{1}H NMR (400 MHz, Chloroform-*d*) δ 6.82 – 6.37 (m, 4H), 3.41 (d, *J* = 10.4 Hz, 1H), 3.18 (dt, *J* = 24.0, 8.0 Hz, 2H), 1.83 (td, *J* = 39.8, 37.2, 12.0 Hz, 5H), 1.47 – 1.08 (m, 6H). ^{13}C NMR (126 MHz, Chloroform-*d*) δ 133.89, 133.53, 118.81, 118.39, 114.38, 55.24, 44.03, 40.79, 29.21, 29.00, 26.49, 26.22, 26.17.

$[\alpha]_D^{25}$ = +15.998 (*c* 0.1, $CHCl_3$).

HRMS (ESI) calcd for $C_{14}H_{21}N_2^+$ [$(M+H)^+$] 217.1700, found 217.1703.

Daicel Chiralpak OD-H column, isocratic elution: n-hexane/2-propanol 80:20, flow rate 0.5 mL/min, column temp 40 °C, retention times 13.840 min (major enantiomer),

17.250 min (minor enantiomer), 33% *ee*.

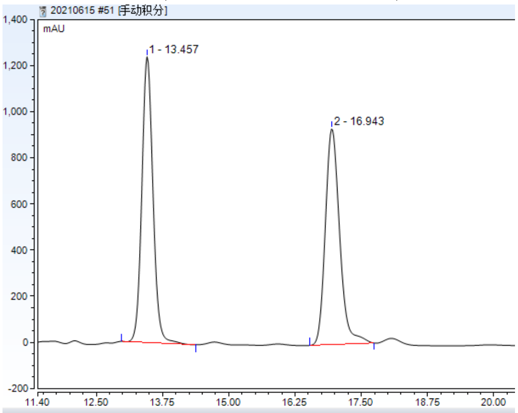

	A	B	C	D	E	F
1	Peak	RetTime	Area	Area	Height	Width (50%)
2	#	min	%	mAU*min	mAU	min
4	1	13.457	50.64	300.8635	1239.06	0.221
5	2	16.943	49.36	293.2323	936.39	0.282

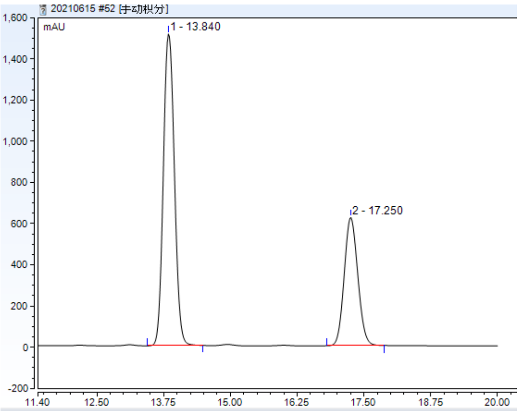

	A	B	C	D	E	F
1	Peak	RetTime	Area	Area	Height	Width (50%)
2	#	min	%	mAU*min	mAU	min
4	1	13.840	66.45	362.6872	1514.78	0.225
5	2	17.250	33.55	183.1099	622.57	0.275

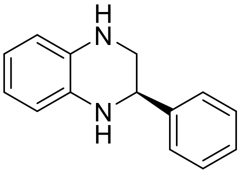

(*R*)-2-phenyl-1,2,3,4-tetrahydroquinoxaline (2o)

Yellow solid. ^{1}H NMR (400 MHz, Chloroform-*d*) δ 7.47 – 7.40 (m, 4H), 7.37 (ddd, *J* = 6.1, 5.0, 2.4 Hz, 1H), 6.74 – 6.67 (m, 2H), 6.66 – 6.60 (m, 2H), 4.53 (dd, *J* = 8.2, 3.1 Hz, 1H), 3.51 (dd, *J* = 11.1, 3.1 Hz, 1H), 3.37 (dd, *J* = 11.0, 8.2 Hz, 1H). ^{13}C NMR (101 MHz, Chloroform-*d*) δ 141.92, 134.22, 132.89, 128.74, 127.99, 127.09, 119.00, 118.87, 114.81, 114.52, 54.79, 49.22.

$[\alpha]_D^{25}$ = -28.524 (*c* 0.2, $CHCl_3$).

HRMS (ESI) calcd for $C_{14}H_{15}N_2^+$ [$(M+H)^+$] 211.1230, found 211.1232.

Daicel Chiralpak OD-H column, isocratic elution: n-hexane/2-propanol 90:10, flow rate 1 mL/min, column temp 40 ^{o}C, retention times 16.020 min (major enantiomer), 22.773 min (minor enantiomer), 19% *ee*.

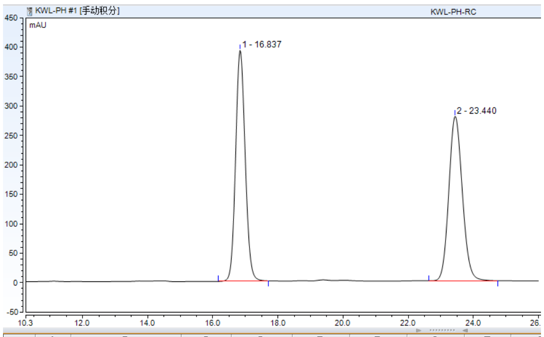

	A	B	C	D	E	F	G	H
1	Peak	RetTime	Area	Area	Height	Width (50%)		
2	#	min	%	mAU*min	mAU	min		
4	1	16.837	49.93	133.6309	392.44	0.314		
5	2	23.440	50.07	134.0033	279.54	0.443		

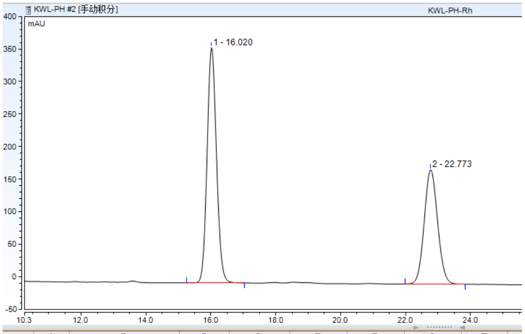

	A	B	C	D	E	F	G	H
1	Peak	RetTime	Area	Area	Height	Width (50%)		
2	#	min	%	mAU*min	mAU	min		
4	1	16.020	59.11	120.9660	361.48	0.310		
5	2	22.773	40.89	83.6877	175.65	0.443		

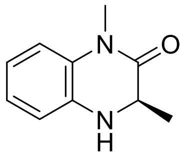

(*R*)-1,3-dimethyl-3,4-dihydroquinoxalin-2(1H)-one (4a)

Yellow solid. ^{1}H NMR (400 MHz, Chloroform-*d*) δ 7.00 – 6.85 (m, 3H), 6.80 – 6.74 (m, 1H), 3.98 (q, *J* = 6.7 Hz, 1H), 3.89 – 3.86 (m, 1H), 3.38 (d, *J* = 1.5 Hz, 3H), 1.47 (dd, *J* = 6.8, 1.6 Hz, 3H). ^{13}C NMR (101 MHz, Chloroform-*d*) δ 168.35, 134.62, 129.41, 123.57, 120.12, 114.80, 114.61, 52.28, 29.18, 17.77.

$[\alpha]_D^{25}$ = -90.0 (*c* 0.1, $CHCl_3$).

HRMS (ESI) calcd for $C_{10}H_{12}N_2O^+$ [$(M+H)^+$] 177.1022, found 177.1026.

Daicel Chiralpak AD-H column, isocratic elution: n-hexane/2-propanol 85:15, flow rate 1mL/min, column temp 30 °C, retention times 7.457 min (minor enantiomer), 7.957 min (major enantiomer), 99% *ee*.

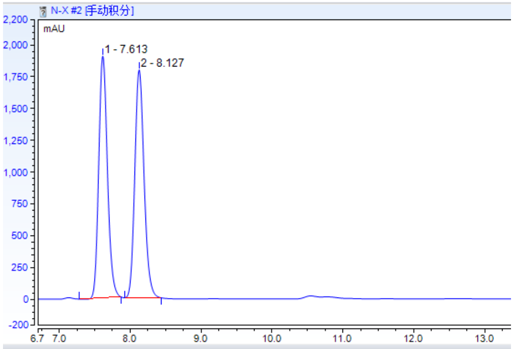

	A	B	C	D	E	F
1	Peak	RetTime	Area	Area	Height	Width (50%)
2	#	min	%	mAU*min	mAU	min
4	1	7.613	49.73	268.2696	1899.14	0.130
5	2	8.127	50.27	271.2094	1790.96	0.139

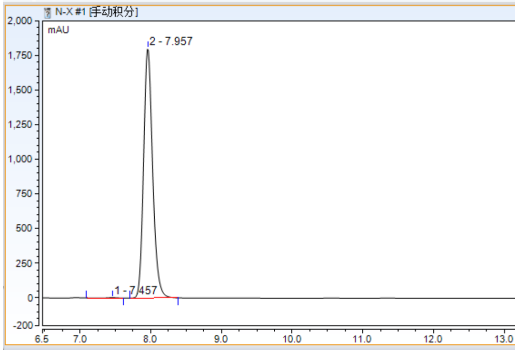

	A	B	C	D	E	F
1	Peak	RetTime	Area	Area	Height	Width (50%)
2	#	min	%	mAU*min	mAU	min
4	1	7.457	0.14	0.3824	3.37	0.117
5	2	7.957	99.86	266.6666	1801.23	0.134

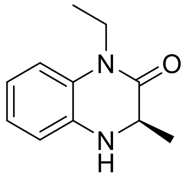

(*R*)-1-ethyl-3-methyl-3,4-dihydroquinoxalin-2(1H)-one (4b)

Yellow solid. ^{1}H NMR (400 MHz, Chloroform-*d*) δ 6.98 – 6.81 (m, 3H), 6.71 (dd, *J* = 7.6, 1.6 Hz, 1H), 3.98 (ddq, *J* = 28.4, 13.2, 6.9 Hz, 3H), 1.42 (d, *J* = 6.6 Hz, 3H), 1.26 (t, *J* = 7.1 Hz, 3H). ^{13}C NMR (101 MHz, Chloroform-*d*) δ 167.75, 135.38, 128.02, 123.39, 119.81, 114.65, 114.61, 52.21, 37.11, 17.77, 12.68.

$[\alpha]_D^{25}$ = -76.0 (*c* 0.1, $CHCl_3$).

HRMS (ESI) calcd for $C_{11}H_{15}N_2O^+$ [$(M+H)^+$] 191.1179, found 191.1171.

Daicel Chiralpak AD-H column, isocratic elution: n-hexane/2-propanol 85:15, flow rate 1mL/min, column temp 30 °C, retention times 6.883 min (minor enantiomer), 7.970 min (major enantiomer), 99% *ee*.

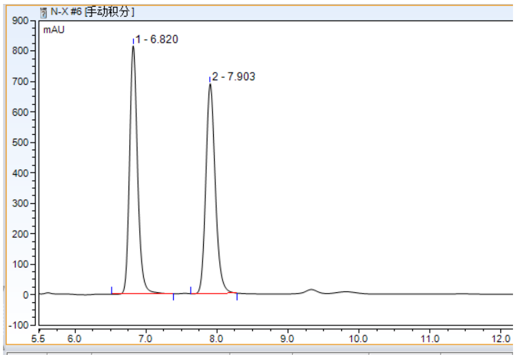

	A	B	C	D	E	F
1	Peak	RetTime	Area	Area	Height	Width (50%)
2	#	min	%	mAU*min	mAU	min
4	1	6.820	50.16	106.5510	814.47	0.117
5	2	7.903	49.84	105.8522	688.78	0.139

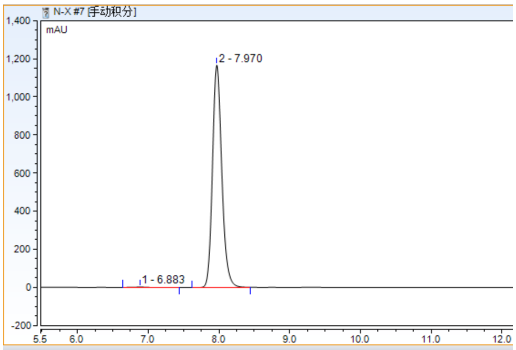

	A	B	C	D	E	F
1	Peak	RetTime	Area	Area	Height	Width (50%)
2	#	min	%	mAU*min	mAU	min
4	1	6.883	0.18	0.3198	2.38	0.121
5	2	7.970	99.82	178.1350	1166.23	0.138

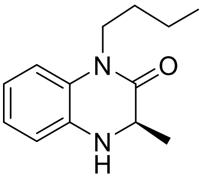

(*R*)-1-butyl-3-methyl-3,4-dihydroquinoxalin-2(1H)-one (4c)

Yellow solid. ^{1}H NMR (400 MHz, Chloroform-*d*) δ 6.96 – 6.80 (m, 3H), 6.71 (d, *J* = 7.4 Hz, 1H), 3.92 (dt, *J* = 9.7, 6.0 Hz, 4H), 1.63 (p, *J* = 7.8 Hz, 2H), 1.40 (ddd, *J* = 15.3, 7.1, 2.0 Hz, 5H), 0.95 (td, *J* = 7.4, 2.0 Hz, 3H). ^{13}C NMR (101 MHz, Chloroform-*d*) δ 167.95, 135.39, 128.21, 123.35, 119.75, 114.81, 114.64, 52.21, 41.88, 29.33, 17.80, 13.92.

$[\alpha]_D^{25}$ = -77.7 (*c* 0.1, $CHCl_3$).

HRMS (ESI) calcd for $C_{13}H_{18}N_2O^+$ [(M+H)$^+$] 219.1492, found 219.1495.

Daicel Chiralpak AD-H column, isocratic elution: n-hexane/2-propanol 85:15, flow rate 1mL/min, column temp 30 °C, retention times 6.123 min (minor enantiomer), 6.790 min (major enantiomer), 99% *ee*.

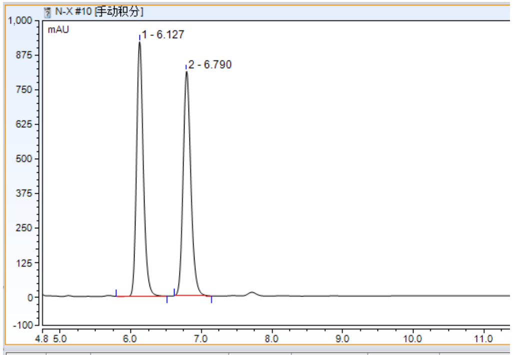

	A	B	C	D	E	F
1	Peak	RetTime	Area	Area	Height	Width (50%)
2	#	min	%	mAU*min	mAU	min
4	1	6.127	49.93	102.3220	918.23	0.100
5	2	6.790	50.07	102.5893	809.79	0.115

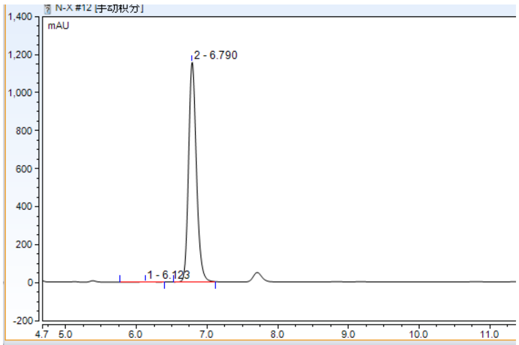

	A	B	C	D	E	F
1	Peak	RetTime	Area	Area	Height	Width (50%)
2	#	min	%	mAU*min	mAU	min
4	1	6.123	0.07	0.1001	1.15	0.092
5	2	6.790	99.93	148.3318	1155.01	0.116

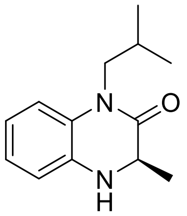

(*R*)-1-isobutyl-3-methyl-3,4-dihydroquinoxalin-2(1H)-one (4d)

White solid. ^{1}H NMR (400 MHz, Chloroform-*d*) δ 6.99 – 6.90 (m, 2H), 6.90 – 6.82 (m, 1H), 6.75 (d, *J* = 7.6 Hz, 1H), 4.00 – 3.85 (m, 3H), 3.78 (dd, *J* = 14.0, 7.1 Hz, 1H), 2.12 (hept, *J* = 6.9 Hz, 1H), 1.45 (d, *J* = 6.6 Hz, 3H), 0.96 (t, *J* = 6.3 Hz, 6H). ^{13}C NMR (101 MHz, Chloroform-*d*) δ 168.39, 135.55, 128.25, 123.35, 119.60, 115.33, 114.74, 52.26, 48.34, 26.45, 20.16, 20.02, 17.84.

$[\alpha]_D^{25}$ = -89.0 (*c* 0.1, $CHCl_3$).

HRMS (ESI) calcd for $C_{13}H_{18}N_2O^+$ $[(M+H)^+]$ 219.1492, found 219.1493.

Daicel Chiralpak AD-H column, isocratic elution: n-hexane/2-propanol 85:15, flow rate 1mL/min, column temp 30 °C, retention times 5.473 min (minor enantiomer), 5.970 min (major enantiomer), 99% *ee*.

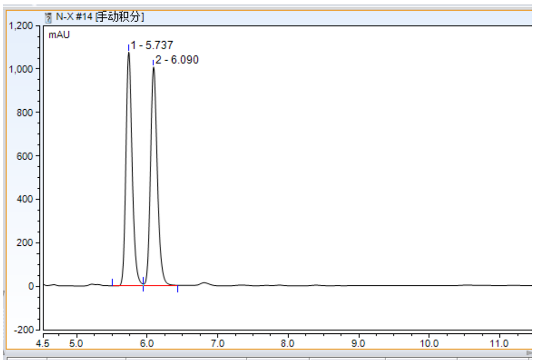

	A	B	C	D	E	F
1	Peak	RetTime	Area	Area	Height	Width (50%)
2	#	min	%	mAU*min	mAU	min
4	1	5.737	49.51	110.7391	1074.68	0.093
5	2	6.090	50.49	112.9337	1005.32	0.102

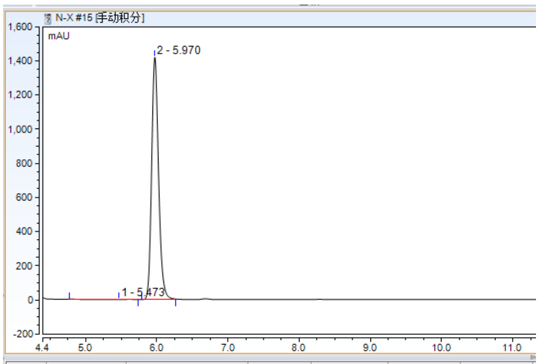

	A	B	C	D	E	F
1	Peak	RetTime	Area	Area	Height	Width (50%)
2	#	min	%	mAU*min	mAU	min
4	1	5.473	0.45	0.7269	1.02	0.888
5	2	5.970	99.55	159.4798	1418.05	0.102

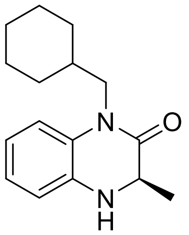

(*R*)-1-(cyclohexylmethyl)-3-methyl-3,4-dihydroquinoxalin-2(1H)-one (4e)

Yellow solid. ^{1}H NMR (400 MHz, Chloroform-*d*) δ 7.00 – 6.91 (m, 2H), 6.90 – 6.84 (m, 1H), 6.74 (d, *J* = 7.7 Hz, 1H), 4.02 – 3.76 (m, 4H), 1.78 – 1.58 (m, 6H), 1.45 (d, *J* = 6.6 Hz, 3H), 1.22 – 1.07 (m, 4H). ^{13}C NMR (101 MHz, Chloroform-*d*) δ 168.29, 135.40, 128.47, 123.28, 119.68, 115.29, 114.68, 52.23, 47.41, 35.84, 30.87, 30.75, 26.40, 25.92, 25.89, 17.88.

$[\alpha]_D^{25}$ = -57.7 (*c* 0.1, $CHCl_3$).

HRMS (ESI) calcd for $C_{16}H_{23}N_2O^+$ [$(M+H)^+$] 287.2052, found 287.2050.

Daicel Chiralpak OD-H column, isocratic elution: n-hexane/2-propanol 90:10, flow rate 1mL/min, column temp 40 °C, retention times 7.843 min (major enantiomer), 11.013 min (minor enantiomer), 98% *ee*.

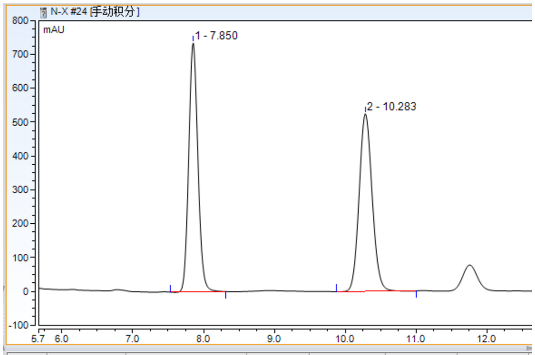

Peak #	RetTime min	Area %	Area mAU*min	Height mAU	Width (50%) min
1	7.850	49.74	110.5598	734.88	0.138
2	10.283	50.26	111.7110	524.71	0.197

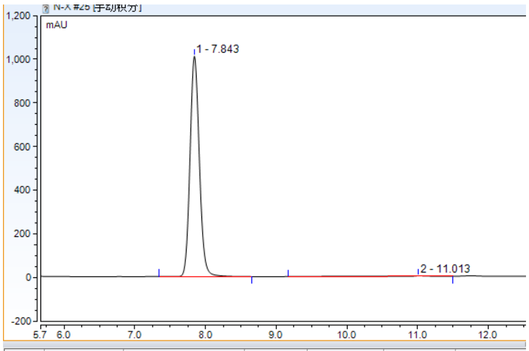

Peak #	RetTime min	Area %	Area mAU*min	Height mAU	Width (50%) min
1	7.843	99.03	154.3762	1009.52	0.140
2	11.013	0.97	1.5192	2.24	0.217

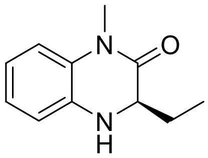

(*R*)-3-ethyl-1-methyl-3,4-dihydroquinoxalin-2(1H)-one (4f)
Yellow oil. ^{1}H NMR (400 MHz, Chloroform-*d*) δ 6.95 (ddd, *J* = 9.1, 7.1, 1.8 Hz, 2H), 6.90 – 6.82 (m, 1H), 6.74 (dd, *J* = 7.6, 1.5 Hz, 1H), 4.09 (s, 1H), 3.84 (ddd, *J* = 8.3, 4.6, 1.7 Hz, 1H), 3.39 (s, 3H), 1.95 – 1.67 (m, 2H), 1.04 (t, *J* = 7.4 Hz, 3H). ^{13}C NMR (101 MHz, Chloroform-*d*) δ 167.88, 134.66, 128.94, 123.57, 119.47, 114.61, 114.30, 57.90, 29.03, 24.84, 9.83.

$[\alpha]_D^{25}$ = -38.0 (*c* 0.1, $CHCl_3$).

HRMS (ESI) calcd for $C_{11}H_{15}N_2O^+$ [$(M+H)^+$] 191.1179, found 191.1175.

Daicel Chiralpak OD-H column, isocratic elution: n-hexane/2-propanol 90:10, flow rate 1mL/min, column temp 40 °C, retention times 10.703 min (major enantiomer), 14.223 min (minor enantiomer), 99% *ee*.

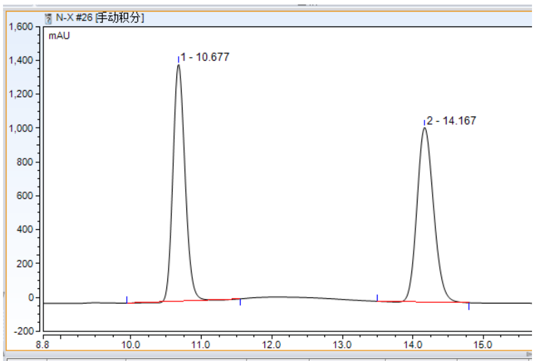

Peak #	RetTime min	Area %	Area mAU*min	Height mAU	Width (50%) min
1	10.677	49.52	275.9130	1400.62	0.184
2	14.167	50.48	281.2883	1031.31	0.254

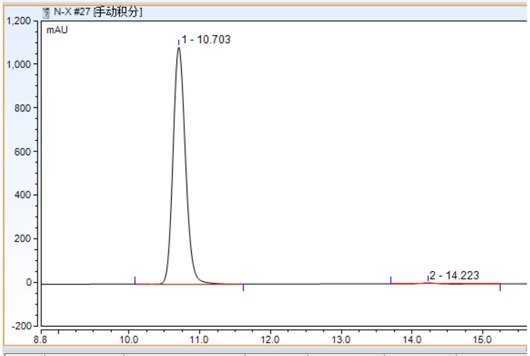

Peak #	RetTime min	Area %	Area mAU*min	Height mAU	Width (50%) min
1	10.703	99.58	221.0900	1087.48	0.187
2	14.223	0.42	0.9252	4.52	0.237

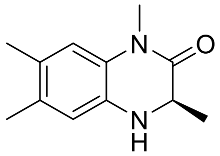

(*R*)-1,3,6,7-tetramethyl-3,4-dihydroquinoxalin-2(1H)-one (4g)

White solid. ^{1}H NMR (400 MHz, Chloroform-*d*) δ 6.73 (s, 1H), 6.54 (s, 1H), 3.93 (q, *J* = 6.7 Hz, 1H), 3.37 (s, 3H), 2.24 (s, 3H), 2.20 (s, 3H), 1.44 (d, *J* = 6.6 Hz, 3H). ^{13}C NMR (101 MHz, $CDCl_3$) δ 168.49, 132.74, 131.51, 127.68, 116.13, 115.88, 52.53, 29.14, 19.37, 19.20, 17.80.

$[\alpha]_D^{25}$ = -100.9 (*c* 0.1, $CHCl_3$).

HRMS (ESI) calcd for $C_{12}H_{17}N_2O^+$ $[(M+H)^+]$ 205.1335, found 205.1335.

Daicel Chiralpak AD-H column, isocratic elution: n-hexane/2-propanol 85:15, flow rate 1mL/min, column temp 30 °C, retention times 7.397 min (minor enantiomer), 9.617 min (major enantiomer), 99% *ee*.

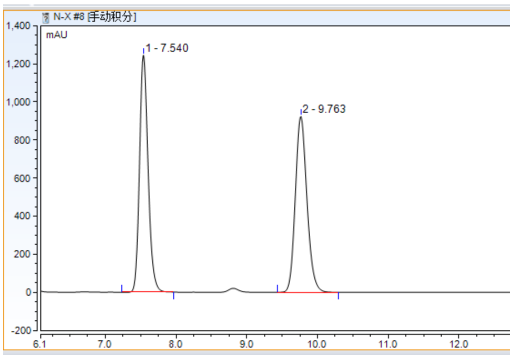

	A	B	C	D	E	F
1	Peak	RetTime	Area	Area	Height	Width (50%)
2	#	min	%	mAU*min	mAU	min
4	1	7.540	49.78	174.2260	1243.12	0.127
5	2	9.763	50.22	175.7702	924.33	0.174

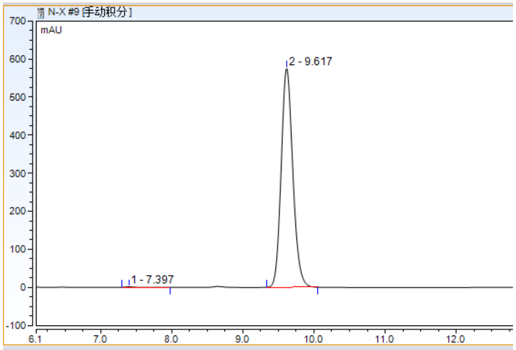

	A	B	C	D	E	F
1	Peak	RetTime	Area	Area	Height	Width (50%)
2	#	min	%	mAU*min	mAU	min
4	1	7.397	0.16	0.1760	1.43	0.115
5	2	9.617	99.84	109.0603	575.51	0.174

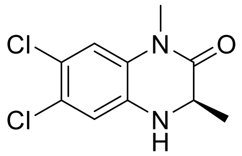

(*R*)-6,7-dichloro-1,3-dimethyl-3,4-dihydroquinoxalin-2(1H)-one (4h)

Yellow solid. ^{1}H NMR (400 MHz, Chloroform-*d*) δ 6.94 (s, 1H), 6.76 (s, 1H), 3.96 (q, *J* = 9.8, 8.2 Hz, 2H), 3.32 (s, 3H), 1.43 (d, *J* = 6.6 Hz, 3H). ^{13}C NMR (101 MHz, Chloroform-*d*) δ 167.64, 134.47, 128.89, 126.37, 122.31, 116.16, 115.09, 52.03, 29.30, 18.10.

$[\alpha]_D^{25}$ = -60.0 (*c* 0.1, $CHCl_3$).

HRMS (ESI) calcd for $C_{10}H_{11}Cl_2N_2O^+$ $[(M+H)^+]$ 245.0243, found 245.0244.

Daicel Chiralpak AD-H column, isocratic elution: n-hexane/2-propanol 90:10, flow rate 0.8 mL/min, column temp 40 °C, retention times 10.347 min (minor enantiomer), 12.353 min (major enantiomer), 98% *ee*.

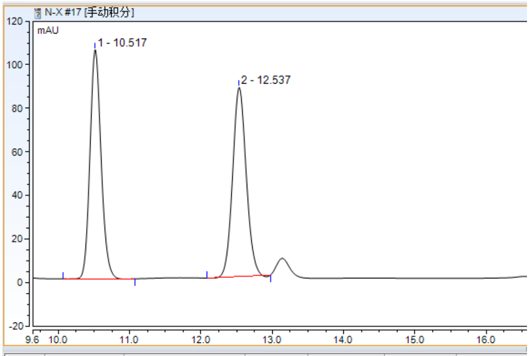

	A	B	C	D	E	F
1	Peak	RetTime	Area	Area	Height	Width (50%)
2	#	min	%	mAU*min	mAU	min
4	1	10.517	49.84	18.9993	105.20	0.165
5	2	12.537	50.16	19.1187	86.86	0.203

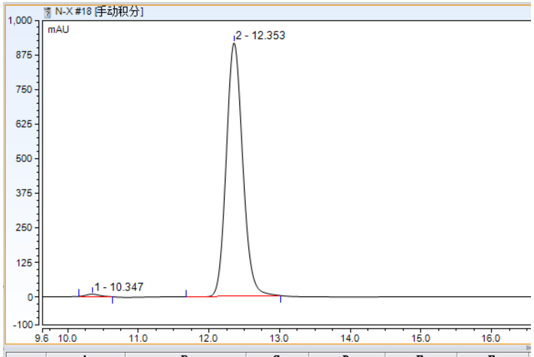

	A	B	C	D	E	F
1	Peak	RetTime	Area	Area	Height	Width (50%)
2	#	min	%	mAU*min	mAU	min
4	1	10.347	0.76	1.8883	8.69	0.203
5	2	12.353	99.24	246.7466	915.61	0.248

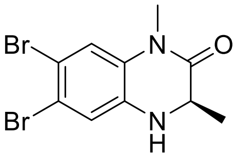

(*R*)-6,7-dibromo-1,3-dimethyl-3,4-dihydroquinoxalin-2(1H)-one (4i)

Yellow solid. ^{1}H NMR (400 MHz, Chloroform-*d*) δ 7.09 (s, 1H), 6.94 (s, 1H), 4.02 – 3.91 (m, 2H), 3.32 (s, 3H), 1.43 (d, *J* = 6.6 Hz, 3H). ^{13}C NMR (101 MHz, Chloroform-*d*) δ 167.60, 135.15, 129.63, 119.06, 118.11, 117.94, 113.33, 52.00, 29.26, 18.15.

$[\alpha]_D^{25}$ = -37.0 (*c* 0.1, $CHCl_3$).

HRMS (ESI) calcd for $C_{10}H_{11}Br_2N_2O^+$ $[(M+H)^+]$ 332.9233, found 332.9236.

Daicel Chiralpak AD-H column, isocratic elution: n-hexane/2-propanol 90:10, flow rate 0.8 mL/min, column temp 40 °C, retention times 11.947 min (minor enantiomer), 14.670 min (major enantiomer), 99% *ee*.

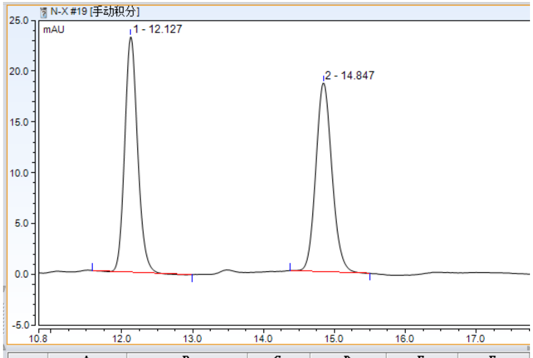

	A	B	C	D	E	F
1	Peak	RetTime	Area	Area	Height	Width (50%)
2	#	min	%	mAU*min	mAU	min
4	1	12.127	49.73	4.8634	23.19	0.193
5	2	14.847	50.27	4.9158	18.59	0.243

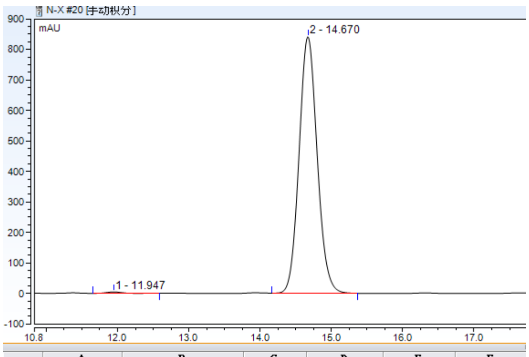

	A	B	C	D	E	F
1	Peak	RetTime	Area	Area	Height	Width (50%)
2	#	min	%	mAU*min	mAU	min
4	1	11.947	0.38	0.9393	4.36	0.219
5	2	14.670	99.62	247.2523	840.75	0.271

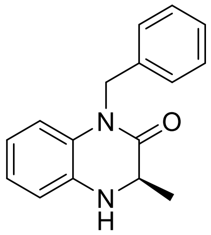

(*R*)-1-benzyl-3-methyl-3,4-dihydroquinoxalin-2(1H)-one (4j)

Yellow solid. ^{1}H NMR (400 MHz, Chloroform-*d*) δ 7.34 – 7.26 (m, 2H), 7.23 (s, 2H), 6.87 (td, *J* = 7.5, 1.3 Hz, 1H), 6.80 (d, *J* = 7.5 Hz, 1H), 6.70 (ddd, *J* = 7.9, 6.2, 1.6 Hz, 2H), 5.20 (d, *J* = 16.2 Hz, 1H), 5.10 (d, *J* = 16.2 Hz, 1H), 4.06 (q, *J* = 6.6 Hz, 1H), 3.99 (s, 1H), 1.49 (d, *J* = 6.6 Hz, 3H). ^{13}C NMR (101 MHz, Chloroform-*d*) δ 168.52, 136.83, 135.26, 128.86, 128.49, 127.23, 126.42, 123.70, 119.84, 115.66, 114.55, 52.39, 45.95, 17.87.

$[\alpha]_D^{25}$ = -74.7 (*c* 0.1, $CHCl_3$).

HRMS (ESI) calcd for $C_{16}H_{17}N_2O^+$ $[(M+H)^+]$ 253.1335, found 253.1333.

Daicel Chiralpak AD-H column, isocratic elution: n-hexane/2-propanol 85:15, flow rate 1 mL/min, column temp 30 ^{o}C, retention times 10.053 min (minor enantiomer), 10.683 min (major enantiomer), 99% ee

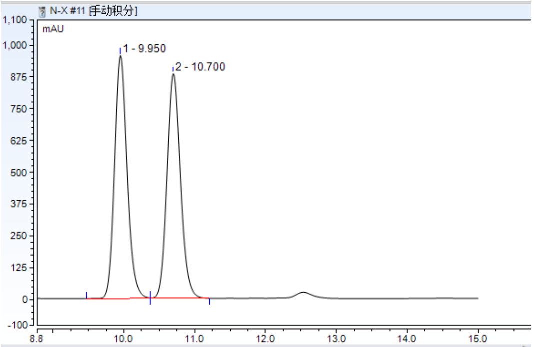

	A	B	C	D	E	F
1	Peak	RetTime	Area	Area	Height	Width (50%)
2	#	min	%	mAU*min	mAU	min
4	1	9.950	50.22	196.3179	955.47	0.187
5	2	10.700	49.78	194.6035	883.48	0.202

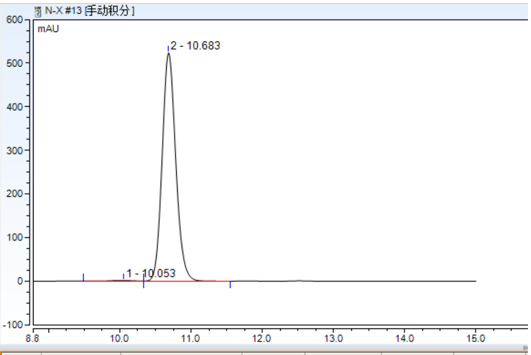

	A	B	C	D	E	F
1	Peak	RetTime	Area	Area	Height	Width (50%)
2	#	min	%	mAU*min	mAU	min
4	1	10.053	0.44	0.5062	1.27	0.365
5	2	10.683	99.56	115.6905	523.87	0.202

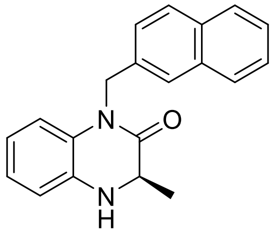

(*R*)-3-methyl-1-(naphthalen-2-ylmethyl)-3,4-dihydroquinoxalin-2(1H)-one (4k)

Yellow solid. ^{1}H NMR (400 MHz, Chloroform-*d*) δ 7.84 – 7.73 (m, 3H), 7.66 (s, 1H), 7.49 – 7.34 (m, 3H), 6.91 – 6.83 (m, 2H), 6.76 – 6.65 (m, 2H), 5.39 (d, *J* = 16.2 Hz, 1H), 5.25 (d, *J* = 16.2 Hz, 1H), 4.14 (q, *J* = 6.6 Hz, 1H), 3.95 (s, 1H), 1.55 (d, *J* = 6.6 Hz, 3H). ^{13}C NMR (101 MHz, Chloroform-*d*) δ 168.54, 135.26, 134.41, 133.51, 132.75, 128.73, 128.56, 127.80, 127.76, 126.24, 125.81, 125.05, 124.70, 123.70, 119.92, 115.74, 114.53, 52.44, 46.18, 17.88.

$[\alpha]_D^{25}$ = -36.0 (*c* 0.1, $CHCl_3$).

HRMS (ESI) calcd for $C_{20}H_{19}N_2O^+$ $[(M+H)^+]$ 303.1492, found 303.1483.

Daicel Chiralpak AD-H column, isocratic elution: n-hexane/2-propanol 85:15, flow rate 1 mL/min, column temp 30 °C, retention times 12.330 min (minor enantiomer), 13.887 min (major enantiomer), 99% *ee*.

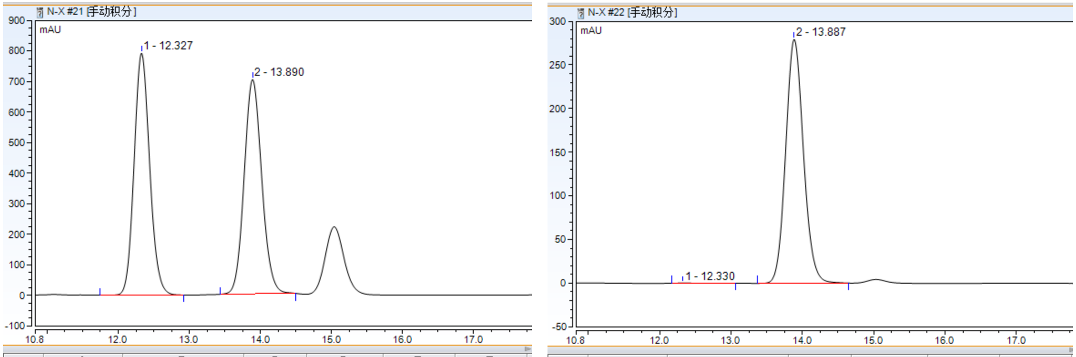

	A	B	C	D	E	F
1	Peak	RetTime	Area	Area	Height	Width (50%)
2	#	min	%	mAU*min	mAU	min
4	1	12.327	49.95	200.6491	792.29	0.233
5	2	13.890	50.05	201.0696	702.04	0.264

	A	B	C	D	E	F
1	Peak	RetTime	Area	Area	Height	Width (50%)
2	#	min	%	mAU*min	mAU	min
4	1	12.330	0.09	0.0727	0.28	0.228
5	2	13.887	99.91	80.7389	279.54	0.266

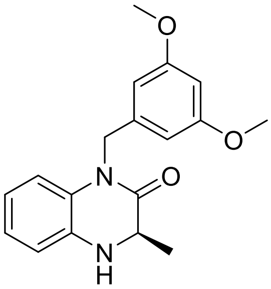

(*R*)-1-(3,5-dimethoxybenzyl)-3-methyl-3,4-dihydroquinoxalin-2(1H)-one (4l)

Yellow solid. ^{1}H NMR (400 MHz, Chloroform-*d*) δ 6.92 (td, *J* = 7.6, 1.4 Hz, 1H), 6.86 (dd, *J* = 8.0, 1.4 Hz, 1H), 6.81 – 6.71 (m, 2H), 6.42 (d, *J* = 2.2 Hz, 2H), 6.37 (d, *J* = 2.3 Hz, 1H), 5.19 – 5.05 (m, 2H), 4.16 – 4.07 (m, 1H), 3.96 (s, 1H), 3.79 (s, 6H), 1.54 (d, *J* = 6.6 Hz, 3H). ^{13}C NMR (101 MHz, Chloroform-*d*) δ 168.39, 161.23, 139.41, 135.08, 128.56, 123.69, 119.91, 115.69, 114.49, 104.44, 98.88, 55.33, 52.40, 46.06, 17.87.

$[\alpha]_D^{25}$ = -61.0 (*c* 0.1, $CHCl_3$).

HRMS (ESI) calcd for $C_{18}H_{21}N_2O^+$ $[(M+H)^+]$ 327.1543, found 327.1648.

Daicel Chiralpak AD-H column, isocratic elution: n-hexane/2-propanol 90:10, flow rate 0.8 mL/min, column temp 30 °C, retention times 27.317min (minor enantiomer), 30.300 min (major enantiomer), 99% *ee*.

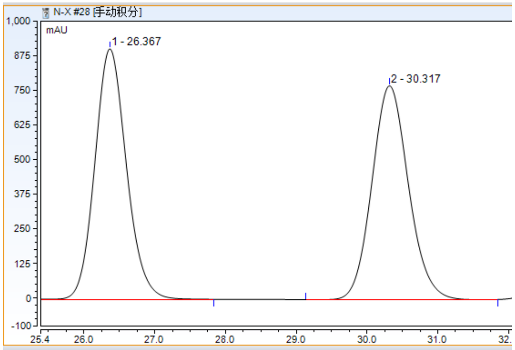

	A	B	C	D	E	F
1	Peak	RetTime	Area	Area	Height	Width (50%)
2	#	min	%	mAU*min	mAU	min
4	1	26.367	50.37	467.2929	903.49	0.474
5	2	30.317	49.63	460.3781	770.89	0.551

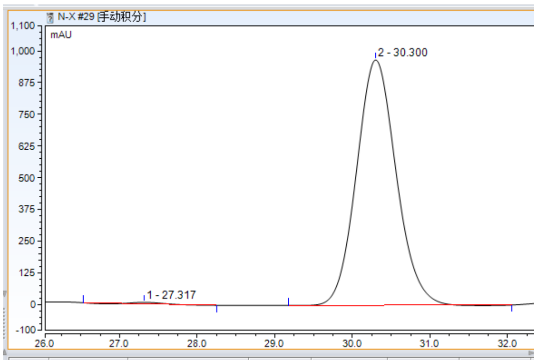

	A	B	C	D	E	F
1	Peak	RetTime	Area	Area	Height	Width (50%)
2	#	min	%	mAU*min	mAU	min
4	1	27.317	0.28	1.6200	6.18	0.424
5	2	30.300	99.72	571.3480	967.11	0.546

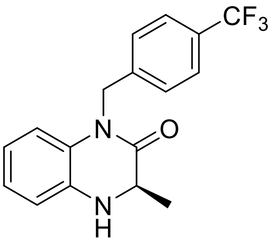

(*R*)-3-methyl-1-(4-(trifluoromethyl)benzyl)-3,4-dihydroquinoxalin-2(1H)-one (4m)
Yellow solid. [1] ^{1}H NMR (400 MHz, Chloroform-*d*) δ 7.61 (d, *J* = 8.1 Hz, 2H), 7.38 (d, *J* = 8.0 Hz, 2H), 6.94 (dq, *J* = 8.7, 4.4 Hz, 1H), 6.82 – 6.74 (m, 3H), 5.35 – 5.13 (m, 2H), 4.13 (qd, *J* = 6.6, 1.5 Hz, 1H), 3.99 (s, 1H), 1.55 (d, *J* = 6.6 Hz, 3H). ^{13}C NMR (101 MHz, Chloroform-*d*) δ 168.52, 141.01, 135.27, 129.58 (q, *J* = 32.4 Hz), 128.16, 126.75, 125.83 (q, *J* = 3.8 Hz), 124.15 (q, *J* = 272.1 Hz), 123.95, 119.95, 115.31, 114.72, 52.36, 45.54, 17.76.

$[\alpha]_D^{25}$ = -60.4 (*c* 0.1, $CHCl_3$).

HRMS (ESI) calcd for $C_{17}H_{16}F_3N_2O^+$ $[(M+H)^+]$ 335.1311, found 335.1312.

Daicel Chiralpak OD-H column, isocratic elution: n-hexane/2-propanol 90:10, flow rate 0.8 mL/min, column temp 30 ^{o}C, retention times 14.373 min (minor enantiomer), 16.993 min (major enantiomer), 99% *ee*.

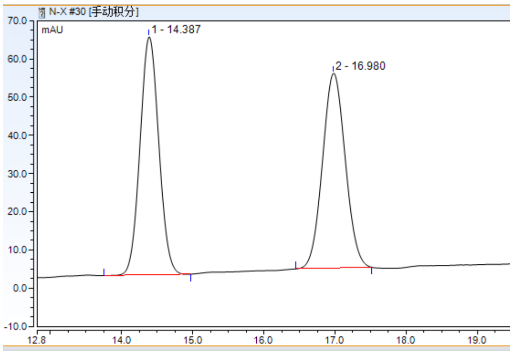

	A	B	C	D	E	F
1	Peak	RetTime	Area	Area	Height	Width (50%)
2	#	min	%	mAU*min	mAU	min
4	1	14.387	50.18	19.0292	62.25	0.286
5	2	16.980	49.82	18.8962	50.98	0.347

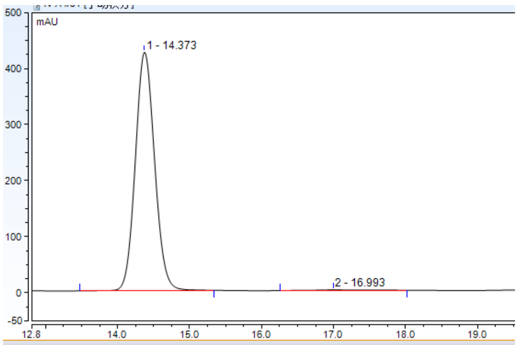

	A	B	C	D	E	F
1	Peak	RetTime	Area	Area	Height	Width (50%)
2	#	min	%	mAU*min	mAU	min
4	1	14.373	99.51	131.8318	425.49	0.287
5	2	16.993	0.49	0.6499	0.93	0.524

11.DFT computational studies

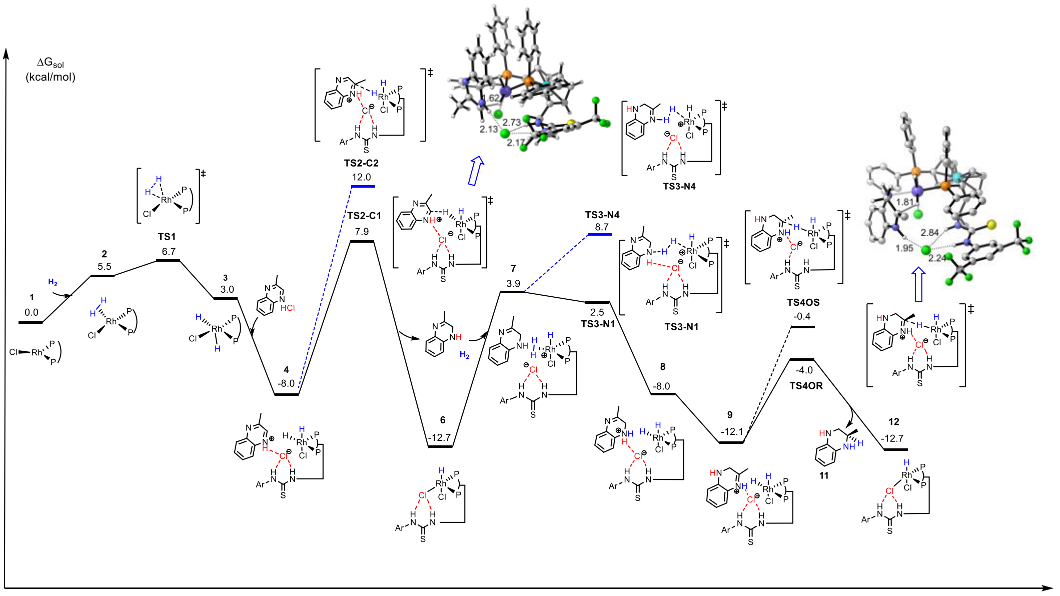

Figure S7. DFT calculated energy potential surface for the proposed pathway. (Ar=3,5-di-CF_3Ph; the phenyl groups on the phosphorus atoms are omitted for clarity; the relative Gibbs energies are labeled in kcal·mol^{-1}).

11.1 Distortion calculation

Table S8. Absolute distortion energies (Hartree) of the catalyst complex (E_{cat}), and of the substrate (E_{sub}), as well as electronic energy (EE) for TS4OR and TS4OS by SMD B3LYP/(6-311g* and SDD) method.

	TS4OR	TS4OS
sub	-458.619509	-458.6109310
cat	-4683.786887	-4683.728671
EE	-5142.532463	-5142.528497

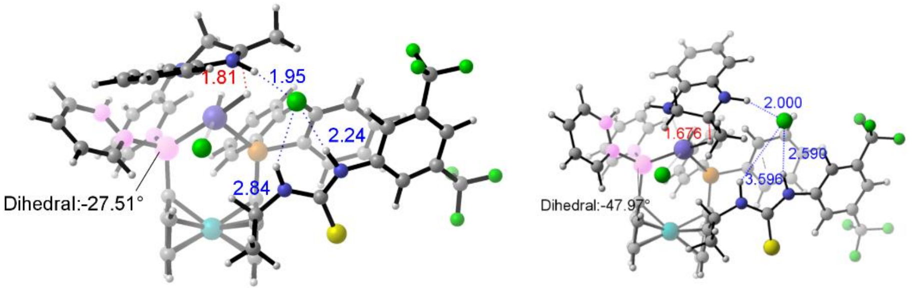

TS4OR
TS4OS

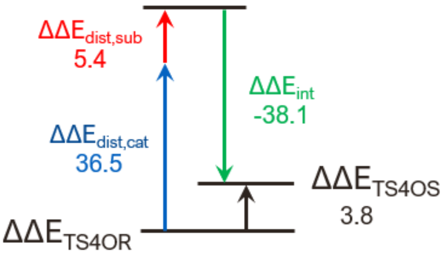

Figure S8. Computed transition states (TS4OS and TS4OR) with relative free energies (kcal mol^{-1}) and distortion model of TS4OR (relative to TS4OS).

11.2 Method for DFT calculation

All quantum chemistry calculations were conducted using Gaussian 09 packages[8] at China National Supercomputing Center in Shenzhen with Density functional theory (DFT) method. B3LYP functional was used for optimization of all geometries[9, 10]. SDD basis set was used for Rh and Fe[11], and 6-31g* basis set was used for other elements[9, 12]. Then frequency calculations (at 298.15 K) were performed on the optimized geometries to confirm the local minimums (without imaginary frequency) and the transition states (with one imaginary frequency). Solvent effect was included employing SMD solvation model with dichloromethane as the solvent[13]. In the above figure, the transition energy TS3-N1 is slightly lower than intermediate 7 while the electronic energy is inverse. We considered this because the transition state leads to an increase in the enthalpy and a decrease in the Gibbs energy. We calculated these two electronic energies through the PCM solvation method and IRC analysis to further prove this mechanism route.

Table S9. The electronic energies of intermediate 7 and TS3-N1 through different solvation methods and basis sets.

Structure	Calculated method	E(hartree)
7	None+6-31G*	-5141.580786
	SMD+6-31G*	-5141.655062
	PCM+6-311G*	-5142.462423
TS3N1	None+6-31G*	-5141.579643
	SMD+6-31G*	-5141.654368
	PCM+6-311G*	-5142.462251

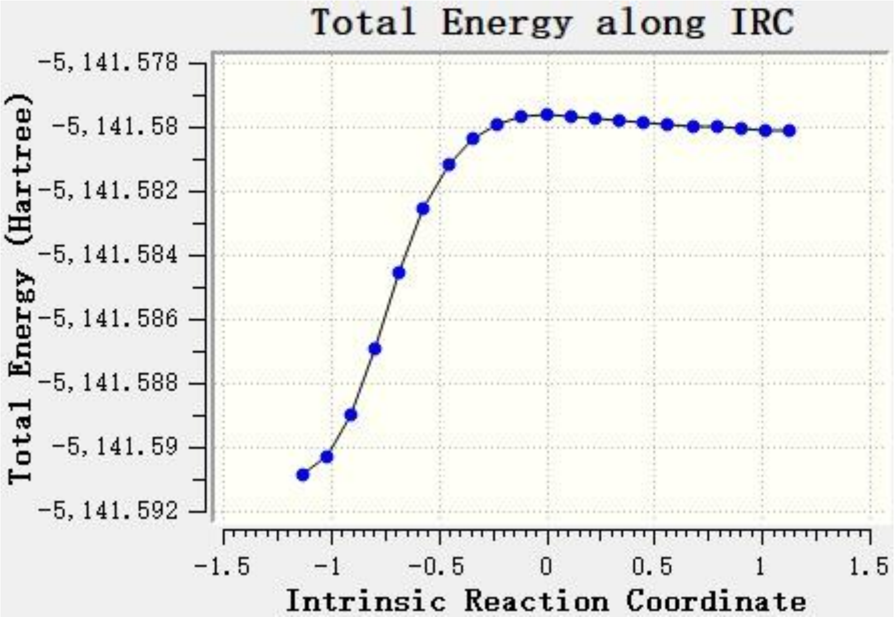

Figure S9. The IRC (Intrinsic Reaction Coordinate) analysis of TS3-N1 in B3LYP/(6-31g* and SDD) method.

Table S10. Absolute enthalpy, electronic and Gibbs free energies (in Hartree) for the intermediates and transition states by the B3LYP/ (6-311g* +SDD)-DCM//B3LYP/(6-31G*+SDD) method.

Num	Files	EE	Thermal Correlation	Gibbs free energy	Relative free energy(kcal/mol)
1	im1	-4221.813388	0.624558	-4221.18883	0
2	im2	-4222.999488	0.641778	-4222.35771	5.537770454
3	im3	-4223.002233	0.640558	-4222.361675	3.049695683
4	im4	-5141.330952	0.790809	-5140.540143	-8.077300997
5	sub2	-458.633438	0.141126	-458.492312	1.476529618
6	im6	-4682.687925	0.635113	-4682.052812	-12.67945494
7		-5142.512291	0.813542	-5141.698749	3.907601435
8	9	-5142.535249	0.817597	-5141.717652	-7.954202478
9		-5142.53917	0.814856	-5141.724314	-12.13457597
12		-4682.687937	0.635131	-4682.052806	-12.67568988
TS1		-4222.992576	0.63675	-4222.355826	6.719998164
TS2-C1		-5141.306987	0.792322	-5140.514665	7.910383496
TS2-C2		-5141.301203	0.793077	-5140.508126	12.01366746
TS3-N1		-5142.512595	0.811563	-5141.701032	2.474809222
TS3-N2		-5142.502287	0.811143	-5141.691144	8.679810422
TS3-Cl		-4683.839977	0.646738	-4683.193239	10.7127589
TS4R		-5142.532463	0.820835	-5141.711628	-4.174192529
TS4S		-5142.528497	0.822816	-5141.705681	-0.442394128

Substrate	Substrate	-457.434958	0.117998	-457.31696	
HCl	HCl	-460.832568	-0.011208	-460.843776	
H_2	H_2	-0.001353	-1.176352	-1.177705	

11.3 Cartesian Coordinates

HCl

Cl	0.00000000	0.00000000	0.07166700
H	0.00000000	0.00000000	-1.21833300

Substrate

C	-2.62739400	-1.05491700	-0.00000100
C	-1.33091600	-1.52048300	-0.00001000
C	-0.52225600	0.79645700	0.00001200
C	-1.86397800	1.24856000	0.00001200
C	-2.89609600	0.33646500	0.00000900
H	-3.45566600	-1.75763000	-0.00000500
H	-1.09897200	-2.58069900	-0.00002000
H	-2.03995000	2.31952400	0.00001700
H	-3.92605000	0.68115800	0.00001400
C	1.71484400	1.21623400	-0.00004100
C	2.00122400	-0.18583400	0.00001000
H	2.53735500	1.93142100	0.00000300
N	0.49758200	1.70631600	-0.00000500
N	1.03104200	-1.07802500	-0.00001500
C	3.42603400	-0.66870100	0.00001900
H	3.96669000	-0.30455600	-0.88227900
H	3.96659200	-0.30477900	0.88247000
H	3.44374400	-1.76023700	-0.00010900

H_2

H	0.00000000	0.00000000	0.37142100
H	0.00000000	0.00000000	-0.37142100

Intermediates:

1

C	-1.72308500	4.15039100	0.98580900
C	-1.29850800	4.05559500	-0.36731500
H	-1.33219800	4.84787800	-1.10403400
C	-0.82087400	2.73687700	-0.62082200
C	-0.97243000	1.98651500	0.60850800
C	-1.51063900	2.88590900	1.59436200
H	-1.73415100	2.63276300	2.61880200
C	1.21759400	0.18209000	1.24194800
C	1.88647200	1.33683700	1.66889000
H	1.38666300	2.29852500	1.64503600
C	3.20966800	1.25330800	2.10424600
H	3.73750000	2.15541600	2.39725300
C	3.86432800	0.02249100	2.13829500
H	4.89761100	-0.02697100	2.46422500
C	3.20859100	-1.12810400	1.69551200
H	3.73141200	-2.07832600	1.67003500
C	1.89541800	-1.04728200	1.23313300
H	1.39601800	-1.93213100	0.85320400
C	-1.27325200	-0.20999600	2.53286400
C	-2.53029600	0.24367600	2.95407900
H	-3.08108500	0.93023200	2.33345800
H	-4.06490100	0.18580000	4.45522700
C	-2.39917800	-1.08630500	4.96503200
H	-2.83259300	-1.42662200	5.90093000
C	-1.15047500	-1.55526400	4.55500100
H	-0.60137500	-2.25904800	5.17410600
C	-0.58888700	-1.11909000	3.35540300
H	0.38876500	-1.48536700	3.06877000
P	-0.57590800	0.22133800	0.87261200
Fe	-2.81564600	2.68275900	-0.00168200
C	-4.04775800	1.07175200	-0.41425300
C	-4.59493500	1.84980200	0.66299100
H	-4.80907400	1.49325800	1.65864500
C	-4.82749800	3.17294800	0.18967500
C	-4.44090200	3.22673100	-1.18105200
C	-3.96557900	1.93891400	-1.55341300
H	-3.60381000	1.65010100	-2.52541400
C	-4.25207500	-1.48264200	0.95061300
C	-5.57759200	-1.20391100	1.30876100
H	-6.13961500	-0.44576700	0.77225200
C	-6.17857900	-1.90128300	2.35625900
H	-7.20435000	-1.67740200	2.63558400
C	-5.46602300	-2.89014200	3.03944800
H	-5.93579200	-3.42912900	3.85735200
C	-4.15351100	-3.18752000	2.66882100
H	-3.59449700	-3.95169500	3.20016800
C	-3.54761700	-2.48581300	1.62885000
H	-2.51664700	-2.68240000	1.34896300
C	-4.17100600	-1.37083700	-1.92439100
C	-3.54633000	-2.50861400	-2.46227900
H	-2.66706200	-2.93256600	-1.97561300
C	-4.05142200	-3.11457200	-3.61219100
H	-3.55401500	-3.98749200	-4.02395700
C	-5.19170900	-2.59525500	-4.22802200
H	-5.58688100	-3.06511300	-5.12393500
C	-5.82572800	-1.47299000	-3.68997000

H	-6.71695000	-1.07215900	-4.16460600
C	-5.31823000	-0.86027500	-2.54426200
H	-5.81086200	0.01619800	-2.13633200
P	-3.39835800	-0.63276800	-0.42969800
H	-2.15922300	5.01864500	1.46174000
H	-4.46666800	4.10095500	-1.81771800
H	-5.20092500	3.99778600	0.78164900
S	2.81160200	3.14438800	-1.32047800
N	0.88087100	1.36043900	-1.78149200
H	0.56903200	0.39909300	-1.90936600
N	2.93233200	0.43725000	-1.69400500
H	2.36416100	-0.38709400	-1.89247600
F	5.38286700	-3.71182100	0.55011200
F	5.36598900	-3.89671800	-1.61493600
F	7.24062300	-3.53236400	-0.56384600
F	6.79714800	1.71178800	1.79383000
F	7.29303500	2.67470500	-0.08953900
F	8.50463300	1.01661600	0.63848600
C	-0.13374300	2.38231400	-1.93541300
C	2.20580300	1.59983200	-1.60120000
C	4.20674000	0.09716000	-1.23234800
C	4.50660300	-1.27555500	-1.23729200
H	3.77807000	-1.98263300	-1.62130000
C	5.70245200	-1.73712000	-0.70183500
C	6.63130300	-0.85291500	-0.15227900
H	7.55576600	-1.21554400	0.27844400
C	6.33623100	0.50751200	-0.17670400
C	5.15089300	0.99621800	-0.72415300
H	4.93761600	2.05461900	-0.72189900
C	5.92856400	-3.21772400	-0.59478500
C	7.24064400	1.47838700	0.52764100
Rh	-1.24404200	-1.08782600	-0.75891300
Cl	0.65942100	-2.02938200	-1.83298100
H	0.40294200	3.28901000	-2.22764700
C	-1.05654100	1.97014600	-3.08470700
H	-0.46332500	1.85012900	-3.99699500
H	-1.54685600	1.01706800	-2.85860900
H	-1.81795600	2.73415800	-3.26070300

2

C	-1.33780900	3.96620500	1.43527700
C	-0.98994700	4.03104000	0.05974500
H	-0.99686500	4.91808600	-0.56059100
C	-0.62468400	2.73255100	-0.39616200
C	-0.77072800	1.82959500	0.72686000
C	-1.18437900	2.61998100	1.85569300
H	-1.36731900	2.24622800	2.85064700
C	1.30963900	-0.14950300	1.07047000
C	2.02617300	0.90615400	1.65086700
H	1.57252500	1.88527300	1.74972100
C	3.33544600	0.70547800	2.08873800
H	3.89930400	1.53547400	2.50252200
C	3.93221800	-0.54929200	1.97082600
H	4.95556200	-0.69119600	2.30163300
C	3.23338300	-1.59796700	1.37145000
H	3.71536400	-2.55908000	1.22598100
C	1.93179000	-1.39784300	0.91070700
H	1.40038500	-2.20520900	0.42042100
C	-1.14926800	-0.57673700	2.35690400
C	-2.32097400	-0.06487600	2.92853800
H	-2.84186900	0.74036500	2.43970100
C	-2.83066100	-0.57871900	4.11915200
H	-3.74399800	-0.16186700	4.53325300
C	-2.19022200	-1.64085200	4.75393900
H	-2.59039200	-2.05121300	5.67643800
C	-1.03253200	-2.17478500	4.18797400
H	-0.51920800	-3.00167200	4.67056100
C	-0.51340800	-1.64617500	3.00649000
H	0.39931700	-2.06553700	2.60349000
P	-0.48501600	0.02431100	0.72962400
Fe	-2.58063300	2.71559200	0.32915800
C	-3.94602500	1.25386000	-0.20133600
C	-4.39272600	1.93491600	0.98188400
H	-4.59020000	1.47962700	1.94001300
C	-4.53935100	3.31893700	0.67809500
C	-4.20119800	3.51054900	-0.69315900
C	-3.83360100	2.24626500	-1.23295800
H	-3.52147100	2.05256600	-2.24540300
C	-4.22377600	-1.43414900	0.92386400
C	-5.47223000	-1.13065000	1.48363300
H	-6.04946000	-0.29311500	1.10427300
C	-5.98345900	-1.91511700	2.51626500
H	-6.94820300	-1.67146800	2.95247400
C	-5.26443700	-3.02056700	2.97835100
H	-5.66535300	-3.63018800	3.78314200
C	-4.03640300	-3.34485600	2.40080400
H	-3.47294400	-4.20141100	2.75764400
C	-3.51687500	-2.55443100	1.37720400
H	-2.55079500	-2.78246700	0.93667500

C	-4.80008200	-0.99335800	-1.77019300
C	-4.83401900	-2.34535400	-2.14948300
H	-4.13092800	-3.04781600	-1.70993600
C	-5.77089600	-2.80123200	-3.07236700
H	-5.78141900	-3.84982400	-3.35540100
C	-6.69356400	-1.91220300	-3.63101800
H	-7.42213800	-2.26546800	-4.35486300
C	-6.67764400	-0.57272500	-3.24817100
H	-7.39796400	0.12360000	-3.66835400
C	-5.73985000	-0.11407700	-2.31851600
H	-5.75261500	0.92746900	-2.02105800
P	-3.54343000	-0.50117100	-0.50146900
H	-1.68309300	4.78699300	2.04980700
H	-4.18187400	4.45475500	-1.22067500
H	-4.82311000	4.09174700	1.37999200
S	2.89767700	3.23745400	-0.99903400
N	1.01977800	1.49902500	-1.75440500
H	0.74010000	0.55688300	-2.02494300
N	3.07897800	0.59725100	-1.69979200
H	2.52111200	-0.20787800	-1.98530700
F	5.61779500	-3.78150500	0.00016700
F	5.59430800	-3.65164200	-2.16909800
F	7.46336800	-3.39070000	-1.07773300
F	6.77123700	1.44718800	2.04661200
F	7.35144800	2.66428400	0.34187300
F	8.55175400	0.93537500	0.90508400
C	-0.01399200	2.51351700	-1.77583400
C	2.33102200	1.73185600	-1.49207900
C	4.35020800	0.22337700	-1.25845800
C	4.68491200	-1.12747100	-1.45300900
H	3.98632700	-1.78500600	-1.96055200
C	5.87685700	-1.63771400	-0.95518200
C	6.76657100	-0.82348500	-0.25292800
H	7.68689600	-1.22536900	0.15119800
C	6.43800700	0.51935100	-0.08916900
C	5.25823700	1.05924400	-0.59951500
H	5.01854000	2.10175600	-0.45189800
C	6.14293800	-3.11161600	-1.06174700
C	7.28747900	1.39438400	0.78739600
Rh	-1.42416600	-1.01913400	-1.11926700
Cl	0.68490000	-1.71603700	-2.12316800
H	-2.06305800	-2.15252000	-2.33147300
H	-2.03160200	-1.44427000	-2.73741700
H	0.48913900	3.45385500	-2.01838900
C	-0.99058200	2.17752100	-2.90367700
H	-0.43906600	2.08895400	-3.84538100
H	-1.49750800	1.22596300	-2.70259700
H	-1.73545600	2.96977300	-3.01015200

3

C	-1.23046900	3.97728100	1.20295800
C	-0.91307100	3.91960800	-0.18086000
H	-0.91937200	4.75056900	-0.87404900
C	-0.58127200	2.57951200	-0.52507400
C	-0.70271100	1.78104300	0.67552200
C	-1.08637000	2.66858300	1.73635700
H	-1.25797800	2.38387400	2.76279000
C	1.34930600	-0.21997500	1.07252200
C	2.09653300	0.80289900	1.67244500
H	1.65656200	1.78274000	1.82100200
C	3.41286900	0.56722900	2.06806200
H	3.99981500	1.36983400	2.50357400
C	3.98769000	-0.69071500	1.88441600
H	5.01676200	-0.86031400	2.18358100
C	3.26020600	-1.70462700	1.26054700
H	3.72675900	-2.66542900	1.06733600
C	1.94848200	-1.46879300	0.84348500
H	1.39064000	-2.24816400	0.33559500
C	-1.09619100	-0.55314500	2.40583700
C	-2.22604700	0.02328500	2.99945900
H	-2.72116300	0.84458600	2.50937600
C	-2.72582500	-0.44799700	4.21230100
H	-3.60504100	0.01960000	4.64624500
C	-2.11704800	-1.52776500	4.84881100
H	-2.50822000	-1.90243600	5.79018800
C	-1.00335700	-2.12682500	4.25921700
H	-0.51624100	-2.96917400	4.74248400
C	-0.49448900	-1.64340700	3.05416500
H	0.38548600	-2.11215500	2.63021800
P	-0.44651600	-0.02556100	0.75138300
Fe	-2.52148800	2.66653800	0.23532200
C	-3.94466800	1.22554300	-0.21976200
C	-4.31011300	1.92079000	0.98277600
H	-4.47188000	1.46892900	1.94856700
C	-4.44814700	3.30506100	0.68039300
C	-4.18442400	3.48342500	-0.70885300
C	-3.87716700	2.21007800	-1.26209600
H	-3.65800400	2.00413400	-2.29707900
C	-4.30571300	-1.39753200	0.97117500

C	-5.54811100	-1.02230900	1.50560700
H	-6.11473600	-0.21672800	1.05045700
C	-6.06887000	-1.68923300	2.61218900
H	-7.02835800	-1.38602200	3.02155200
C	-5.36657900	-2.75389600	3.18248800
H	-5.77450300	-3.27495600	4.04388800
C	-4.14459000	-3.14822500	2.63981700
H	-3.58862900	-3.96967600	3.08017000
C	-3.61487800	-2.47225800	1.54159600
H	-2.65153300	-2.76563700	1.14319700
C	-4.98937300	-0.93967000	-1.73489900
C	-4.93743900	-2.14908000	-2.44214800
H	-4.07347100	-2.79632300	-2.33656600
C	-5.98162600	-2.51501800	-3.28987000
H	-5.92621800	-3.45284800	-3.83488500
C	-7.08669500	-1.67604400	-3.44649700
H	-7.89535000	-1.95817800	-4.11456100
C	-7.14654600	-0.47254600	-2.74394100
H	-8.00235900	0.18638100	-2.86064000
C	-6.10714400	-0.10708500	-1.88744700
H	-6.16901900	0.82968000	-1.34474000
P	-3.65335300	-0.55191400	-0.52334000
H	-1.55027300	4.85382200	1.75073000
H	-4.17785700	4.42436200	-1.24264700
H	-4.67630000	4.08627700	1.39315900
S	2.82735600	3.13814900	-1.13027800
N	1.06496300	1.24780400	-1.77939900
H	0.86021700	0.27717800	-2.02671800
N	3.17201100	0.48309100	-1.71768500
H	2.66870700	-0.35542300	-2.00114000
F	5.91709800	-3.70999400	-0.00752700
F	6.02604500	-3.56613200	-2.17313200
F	7.80355900	-3.20566700	-0.96292200
F	6.77348900	1.51790300	2.10527900
F	7.20296900	2.84988200	0.44156100
F	8.57803100	1.22973300	0.92434500
C	-0.03495700	2.18430400	-1.88971000
C	2.35671400	1.57995000	-1.54437200
C	4.46162800	0.19802900	-1.26423900
C	4.89587900	-1.12326100	-1.45578400
H	4.25767100	-1.82907100	-1.97842300
C	6.11076000	-1.55003200	-0.93432600
C	6.92804900	-0.67567700	-0.21810800
H	7.86702900	-1.01153600	0.20286600

C	6.50133500	0.64056700	-0.05866600
C	5.29408000	1.09508600	-0.58549500
H	4.97529600	2.11599700	-0.43627500
C	6.47136200	-3.00461900	-1.03006700
C	7.27292100	1.56503400	0.83926300
Rh	-1.54007000	-1.06988000	-1.18541600
H	-1.75847400	-2.45462300	-0.60429700
Cl	0.50867500	-1.82968100	-2.25558600
H	-2.21272900	-1.75635200	-2.42565700
C	-1.05104500	1.58687300	-2.86373600
H	-1.80822600	2.32674700	-3.12411700
H	-0.55080500	1.24982500	-3.77575500
H	-1.58743000	0.71448000	-2.44576900
H	0.36707000	3.10249500	-2.33116100

4

C	0.59291900	-3.50533000	-2.46362600
H	0.45903000	-3.79294100	-3.50195500
C	0.02659600	-2.38774600	-1.85015900
C	0.20173200	-2.44529200	-0.40202000
C	0.36992100	-3.75369300	0.12563800
H	0.62586500	-3.87915700	1.16892600
C	-1.50925100	-0.51017200	0.96929300
C	-1.89840700	0.82298400	1.13036000
H	-1.20693500	1.60806300	0.86174700
C	-3.16810100	1.12840000	1.62002700
H	-3.48006900	2.16364700	1.71985200
C	-4.05738300	0.10251100	1.94026700
H	-5.05220900	0.34574300	2.29786200
C	-3.69073700	-1.22921300	1.73606200
H	-4.40968800	-2.02122100	1.91962000
C	-2.41847700	-1.53969700	1.25672100
H	-2.13618800	-2.57503400	1.10099800
C	0.74100200	-1.41419100	2.35665300
C	1.75126500	-2.34587600	2.62117700
H	2.24706400	-2.83928400	1.80219800
C	2.13268600	-2.64292600	3.92775900
H	2.92075600	-3.36860700	4.10246900
C	1.53415600	-1.98034100	4.99743200
H	1.83718100	-2.20115100	6.01676400
C	0.54819400	-1.02469400	4.74798700
H	0.07443600	-0.50059000	5.57332500
C	0.14738800	-0.74823600	3.44136500
H	-0.63674300	-0.02031300	3.26957400

P	0.24100400	-0.92604200	0.64755800
Fe	1.86766900	-3.25796300	-1.25324700
C	3.69596700	-2.21134400	-0.65153700
C	3.68278300	-3.51659200	-0.08000600
H	3.75122200	-3.75199900	0.97113600
C	3.57528200	-4.47838200	-1.12809500
C	3.53642600	-3.77355700	-2.36581400
C	3.57462500	-2.38032100	-2.07188200
H	3.50655900	-1.56694300	-2.78308600
C	4.24192000	-0.72429800	1.82503800
C	5.24860400	-1.62326000	2.20195000
H	5.69437900	-2.28047100	1.46169000
C	5.68869600	-1.66879600	3.52426600
H	6.46398900	-2.37390100	3.81093300
C	5.14489400	-0.80093000	4.47454200
H	5.48983700	-0.83785500	5.50397100
C	4.16081800	0.11382800	4.09807400
H	3.72884600	0.78536600	4.83415300
C	3.70832100	0.15011300	2.78057900
H	2.91986800	0.83805400	2.49312900
C	5.15089800	0.27056700	-0.63479700
C	5.25404700	1.65661800	-0.45584400
H	4.45598200	2.20067200	0.03774100
C	6.36751000	2.34660000	-0.92772100
H	6.43061300	3.42105300	-0.78238800
C	7.38387300	1.65916800	-1.59668300
H	8.24935000	2.19677100	-1.97373600
C	7.28267500	0.28002400	-1.78225500
H	8.06955600	-0.25897000	-2.30258300
C	6.17210800	-0.41556800	-1.29875600
H	6.10042800	-1.48839900	-1.44583300
P	3.67800400	-0.55098900	0.09015500
H	-0.20501400	-4.56974200	-0.29475100
H	3.44572900	-4.21192600	-3.35006500
H	3.53078600	-5.55085500	-0.99644900
S	-3.07938400	-2.55908800	-2.17975000
N	-1.48916500	-0.41887000	-2.12564700
H	-1.36048900	0.58392100	-1.98986300
N	-3.65655700	0.08678000	-1.79595700
H	-3.25539700	1.02861000	-1.83315600
F	-6.24193700	2.90870900	1.75096600
F	-6.62760700	3.76766600	-0.20807800
F	-8.24127200	2.80700300	0.89739400
F	-6.95683500	-2.77379600	1.32711500

F	-7.51482800	-3.17065400	-0.73502000
F	-8.84157400	-2.01589800	0.55058400
C	-0.33139100	-1.13138900	-2.64342700
H	0.49418200	-0.43001200	-2.48945000
C	-0.42669400	-1.36149300	-4.15611000
H	-1.20585600	-2.08776100	-4.39790100
H	0.53228000	-1.70943300	-4.55312200
H	-0.66669900	-0.41005100	-4.63905900
C	-2.74775000	-0.91397800	-2.03174700
C	-4.91427500	0.06963000	-1.19232100
C	-5.39481900	1.31936400	-0.76563400
H	-4.80842400	2.21160000	-0.96238800
C	-6.56821000	1.40452400	-0.02813700
C	-7.30863700	0.26306900	0.28348000
H	-8.21489000	0.33166400	0.87131800
C	-6.84596600	-0.96525800	-0.18204600
C	-5.67083800	-1.07769000	-0.92479400
H	-5.32025900	-2.04273000	-1.25979800
C	-6.93054900	2.72340000	0.58787500
C	-7.54731000	-2.22890100	0.22816100
Rh	1.56866600	0.53064400	-0.38251700
Cl	-2.13413800	2.98128800	-1.77865900
H	0.23540500	1.29909100	-0.73098000
H	1.46243100	1.25683000	0.96104500
Cl	2.46223400	1.30237600	-2.52021800
C	2.89315500	4.31220400	-1.63668200
C	2.22924200	4.18575700	0.57302300
C	0.86809300	4.02391300	0.19966100
C	1.54611700	4.06286900	-2.04586200
H	3.59685400	4.27268100	2.22930500
C	2.55485200	4.16041200	1.94973600
C	-0.15381800	3.90415700	1.15946000
H	-0.39876400	3.68370500	-1.43674800
H	1.25516800	3.95987800	-3.08262800
C	0.20011000	3.88524700	2.49284800
C	1.55559700	3.99947600	2.88733900
H	-1.18118800	3.80946900	0.82391200
H	-0.57035300	3.77433500	3.24916900
H	1.80559500	3.97353900	3.94345500
N	0.59468800	3.96872200	-1.14178700
N	3.21044400	4.35995800	-0.35988800
C	3.97501300	4.45653200	-2.66377800
H	4.38154800	3.46425000	-2.89007800
H	3.59407500	4.88838500	-3.59394900

H	4.77958400	5.08204200	-2.27141100

5

C	-2.94658300	0.30137200	0.01929000
C	-1.89792500	1.21572800	-0.07812700
C	-0.57427900	0.76470300	-0.09453300
C	-0.31024500	-0.62343000	-0.03366700
C	-1.37352200	-1.52332200	0.05703300
C	-2.69124200	-1.07004300	0.09550100
H	-3.96957500	0.66692200	0.03862600
H	-2.10151300	2.28285000	-0.13716800
H	-1.13623000	-2.58219100	0.09254200
H	-3.51093400	-1.77748800	0.17279200
C	1.97757600	-0.29748400	0.01397200
N	1.00436100	-1.11971200	-0.12535000
N	0.51556000	1.61809700	-0.23868600
H	0.30699300	2.59901100	-0.10110300
C	3.39241300	-0.78374400	-0.08389200
H	3.91228400	-0.30413800	-0.92413600
H	3.95599100	-0.52712700	0.82430400
H	3.40738200	-1.86637700	-0.22290700
C	1.77269700	1.17480400	0.34302500
H	2.59077600	1.77335900	-0.07204000
H	1.81204000	1.29498600	1.44571800

6

C	-1.16260600	3.89854600	1.61348700
C	-0.83927300	3.98665700	0.23378200
H	-0.85579400	4.88657600	-0.36271000
C	-0.51993500	2.69226200	-0.25995800
C	-0.66233200	1.76705700	0.84979900
C	-1.04096800	2.54348000	2.00512800
H	-1.20145800	2.15736100	2.99876300
C	1.38684000	-0.26916700	1.17364300
C	1.98020300	-1.52941300	0.99865300
H	1.43191900	-2.31967500	0.50045300
C	3.28250400	-1.75441000	1.44181800
H	3.74731300	-2.72060300	1.27821200
C	4.00440400	-0.72366600	2.04704400
H	5.02901100	-0.88619500	2.36383900
C	3.42980100	0.53792100	2.19103900
H	4.01089100	1.35248700	2.61059400
C	2.12172100	0.76886000	1.76485900
H	1.68607400	1.75342400	1.87843400
C	-1.09945600	-0.62748300	2.47445600
C	-2.26417300	-0.09012000	3.03752300
H	-2.76794600	0.72632700	2.54746500
C	-2.78739300	-0.59501000	4.22628200
H	-3.69468400	-0.16195100	4.63632000
C	-2.17014400	-1.67019900	4.86168100
H	-2.58233800	-2.07291200	5.78214000
C	-1.02095400	-2.22903900	4.30116300
H	-0.52725700	-3.06607300	4.78620600
C	-0.48549000	-1.70996500	3.12338300
H	0.42100500	-2.14556000	2.72244800
P	-0.39643000	-0.03199800	0.87010700
Fe	-2.44747500	2.68803000	0.48403600
C	-3.81134600	1.25557900	-0.11468800
C	-4.30461800	1.95264800	1.04357200
H	-4.56317000	1.50767100	1.99202500
C	-4.40944200	3.33563900	0.71940800
C	-3.99127400	3.50705400	-0.63314600
C	-3.61757200	2.23453300	-1.14853800
H	-3.24158500	2.01486400	-2.13801200
C	-4.17992200	-1.41186400	1.04610600
C	-5.43595500	-1.04094600	1.54968700
H	-5.96176400	-0.19071300	1.12876200
C	-6.02593800	-1.77571000	2.57610500
H	-6.99495300	-1.47538100	2.96445400
C	-5.38263500	-2.90409100	3.08994200
H	-5.84458300	-3.47680500	3.88890100
C	-4.15336700	-3.30075700	2.56398800
H	-3.64895700	-4.17978200	2.95288100
C	-3.55542300	-2.55927800	1.54678700
H	-2.59007800	-2.85996900	1.15712500
C	-4.63832600	-1.02383600	-1.67791500
C	-4.46561000	-2.28757800	-2.25824800
H	-3.60079500	-2.89094400	-1.99705200
C	-5.38442800	-2.75730700	-3.19186400
H	-5.23828100	-3.73226300	-3.64713500
C	-6.47986600	-1.96862300	-3.55411900
H	-7.19186800	-2.33250000	-4.28915900
C	-6.65388100	-0.71180300	-2.97584200
H	-7.50166200	-0.09378100	-3.25752800
C	-5.73692600	-0.23765900	-2.03556400
H	-5.87437500	0.74355000	-1.59402800
P	-3.45457600	-0.51046700	-0.37820000
H	-1.47304400	4.71515100	2.25150300
H	-3.92156600	4.44693000	-1.16389600

H	-4.71913200	4.12045100	1.39661000
S	2.70399000	3.14672100	-0.70170400
N	0.94445000	1.49346800	-1.84443600
H	0.73279100	0.60994700	-2.30160000
N	3.00118400	0.60634400	-1.67574300
H	2.46715200	-0.19846300	-2.00526500
F	5.64137400	-3.77466000	-0.02398600
F	5.53654000	-3.61470600	-2.18911900
F	7.43733100	-3.32789900	-1.16109900
F	6.77671000	1.30766300	2.12864800
F	7.15891300	2.69926100	0.50246900
F	8.50248700	1.01659100	0.83666700
C	-0.19994500	2.38439600	-1.70763600
H	-1.01248100	1.77404500	-2.09579400
C	-0.12264800	3.62477100	-2.60351800
H	0.65625300	4.31524000	-2.27703100
H	-1.09041300	4.13909200	-2.60863900
H	0.09457600	3.30550700	-3.62653400
C	2.21744800	1.70972600	-1.42882100
C	4.26902700	0.23510300	-1.22092700
C	4.61833100	-1.10911100	-1.43728400
H	3.92619700	-1.76637400	-1.95354700
C	5.82084400	-1.61184700	-0.95705500
C	6.70455100	-0.79792900	-0.24782800
H	7.63451800	-1.19381500	0.14059500
C	6.35820900	0.53678000	-0.05475200
C	5.16802700	1.06993700	-0.54671800
H	4.91402500	2.10447000	-0.37334100
C	6.11256900	-3.07864300	-1.09494600
C	7.20686200	1.39604400	0.83793300
Rh	-1.27413800	-0.97781000	-0.97972200
Cl	0.74883900	-1.81612000	-1.99413600
H	-1.16221300	-2.17649400	-0.01731400
Cl	-1.90155300	-0.19887400	-3.18976200

7

C	0.34848900	-4.64879000	0.77576000
C	0.08595700	-4.37203800	-0.59158400
H	-0.03406000	-5.10713300	-1.37282700
C	0.00709000	-2.96650400	-0.78092300
C	0.25561300	-2.35442600	0.50716300
C	0.44429900	-3.41400700	1.46485400
H	0.62194900	-3.28961900	2.52088800
C	-1.43321900	-0.06465100	1.04308400
C	-1.83189500	1.24990500	0.78097800
H	-1.14971200	1.93620100	0.30609900
C	-3.11078700	1.67575300	1.13429100
H	-3.43194200	2.68641300	0.90395000
C	-3.99698600	0.78980700	1.74526800
H	-4.99904900	1.12070400	1.99507200
C	-3.61847400	-0.53431900	1.97294100
H	-4.33374700	-1.23787700	2.38605400
C	-2.33985100	-0.96640400	1.62484600
H	-2.05549100	-1.99886200	1.79079200
C	0.94718100	-0.35776100	2.55702800
C	1.91396200	-1.19212400	3.13363800
H	2.30753300	-2.03028400	2.57789300
C	2.39057900	-0.93897500	4.41866200
H	3.14071300	-1.59485500	4.84940000
C	1.93573900	0.17117400	5.12868000
H	2.31715300	0.37462000	6.12490700
C	1.00305900	1.03028900	4.54532300
H	0.65623600	1.90913100	5.08029800
C	0.50482100	0.76628900	3.27163200
H	-0.22288100	1.44035100	2.83500000
P	0.30190900	-0.58260800	0.85403400
Fe	1.87379600	-3.47761000	-0.03311200
C	3.53057200	-2.26395000	-0.25035500
C	3.78479700	-3.27543200	0.73791200
H	4.03437800	-3.10195700	1.77379700
C	3.64486800	-4.54815100	0.11262000
C	3.30372400	-4.33760900	-1.25639700
C	3.21969600	-2.93510600	-1.48446600
H	2.96158800	-2.43658500	-2.40918500
C	4.23668000	0.01850900	1.49272900
C	5.32496400	-0.67577800	2.04342800
H	5.73504000	-1.54158000	1.53423900
C	5.90227500	-0.23800600	3.23381600
H	6.73891600	-0.78522700	3.65864700
C	5.42144700	0.91150400	3.86613800
H	5.87676700	1.25250300	4.79147600
C	4.36659300	1.62578000	3.29912500
H	3.98573600	2.52385800	3.77455900
C	3.77558800	1.18115200	2.11890600
H	2.95215600	1.74275000	1.69737700
C	4.95817600	0.05709300	-1.20979300
C	5.22324400	1.43095900	-1.30603800
H	4.57700000	2.15913700	-0.82252400

C	6.31526100	1.87118100	-2.04877800
H	6.51748900	2.93583200	-2.12105200
C	7.14176800	0.95354100	-2.70238900
H	7.98958600	1.30276300	-3.28467300
C	6.87526000	-0.41159200	-2.60629300
H	7.51332700	-1.13043900	-3.11223300
C	5.78672400	-0.86373200	-1.85795600
H	5.58783800	-1.92683100	-1.78608200
P	3.56652400	-0.45731100	-0.13929200
H	0.48332200	-5.62840500	1.21450600
H	3.09224700	-5.10882000	-1.98467600
H	3.74130000	-5.50677600	0.60467500
S	-3.11038600	-3.24836300	-1.04176300
N	-1.44161900	-1.37037600	-1.98187200
H	-1.32570900	-0.44575300	-2.40323400
N	-3.56776800	-0.65821400	-1.76250200
H	-3.12578000	0.19502600	-2.14102400
F	-6.00868700	3.38400100	0.52387200
F	-6.30231500	3.50517000	-1.62725800
F	-7.99128600	3.07979100	-0.31767400
F	-6.93646400	-2.05448700	2.14536100
F	-7.61799500	-3.08760300	0.36006600
F	-8.80838400	-1.46985800	1.20413900
C	-0.29311600	-2.26980200	-2.09383200
H	0.53692600	-1.59522200	-2.31313600
C	-0.40317300	-3.23091500	-3.28227600
H	-1.24857000	-3.91114800	-3.16124700
H	0.52014200	-3.81161600	-3.38732000
H	-0.54614100	-2.64962700	-4.19735200
C	-2.71074200	-1.71398300	-1.61611200
C	-4.81737000	-0.39325800	-1.19917000
C	-5.22813700	0.95127400	-1.26237900
H	-4.59549100	1.67450100	-1.76781100
C	-6.38705400	1.36067200	-0.61741600
C	-7.18270900	0.45273800	0.08494300
H	-8.07770100	0.77938500	0.59880300
C	-6.78958700	-0.88207800	0.10546400
C	-5.63073300	-1.32049400	-0.53634300
H	-5.33599600	-2.35782200	-0.48981300
C	-6.68182400	2.82892000	-0.52706500
C	-7.54693600	-1.87363700	0.94148600
Rh	1.51599200	0.42792900	-0.78245200
Cl	-1.96263700	1.86689000	-2.74845100
H	1.59850400	1.44727900	0.35735600
Cl	2.23052300	0.26660200	-3.05652200
H	-0.02034900	0.98970000	-1.52445600
H	0.30265300	1.67694200	-1.20483100
C	0.35429600	4.43223800	2.51484900
C	-0.20621800	4.19725600	1.25854700
C	0.62015900	4.03181900	0.13883500
C	2.02611400	4.11719500	0.29297000
C	2.56568600	4.36465200	1.55685500
C	1.74023800	4.51766500	2.67231500
H	-0.30199700	4.56310300	3.37082200
H	-1.28463500	4.14944200	1.14010600
H	3.64660300	4.42568800	1.64059400
H	2.17130400	4.71508500	3.64906600
C	2.40857800	3.87613500	-1.98476200
N	2.89964400	3.92179900	-0.79599400
N	0.14744700	3.67493900	-1.11050400
H	-0.85262500	3.59422400	-1.27594400
C	3.30804700	3.72918900	-3.17150200
H	3.18128300	2.73400100	-3.61348500
H	3.04127900	4.46564000	-3.94111700
H	4.35135400	3.86446700	-2.88478700
C	0.93009100	4.01973300	-2.28646200
H	0.63619900	3.34600300	-3.09766800
H	0.73792200	5.05239200	-2.63425700

8

C	0.38233000	-4.67214900	-0.87248900
C	0.15967200	-3.86579700	-2.02035600
H	0.08104000	-4.22060400	-3.03695900
C	0.06843800	-2.50505800	-1.62169900
C	0.24981900	-2.46730500	-0.18854900
C	0.43253800	-3.82031200	0.26058500
H	0.58630900	-4.13146200	1.28193600
C	-1.48594100	-0.56328700	1.16100900
C	-1.85572200	0.76386900	1.40806200
H	-1.13083700	1.55136500	1.25188900
C	-3.14576200	1.06065300	1.84529800
H	-3.44430000	2.09113400	2.01239400
C	-4.07178000	0.03339700	2.03020700
H	-5.08195200	0.27211000	2.34425700
C	-3.71803200	-1.28780800	1.75165400
H	-4.46082400	-2.07415500	1.83660400
C	-2.42593900	-1.59129600	1.32190800
H	-2.15728600	-2.61735800	1.09555400
C	0.84209400	-1.47899300	2.48862000

C	1.87007600	-2.41604800	2.66358700
H	2.32975500	-2.87602500	1.80134900
C	2.31151300	-2.75857600	3.93937600
H	3.11208800	-3.48307700	4.05193500
C	1.75533800	-2.14415200	5.06081200
H	2.10711200	-2.40199200	6.05551900
C	0.74908800	-1.19122300	4.89751900
H	0.30868900	-0.70769300	5.76484700
C	0.28877100	-0.86470500	3.62193500
H	-0.51096400	-0.14077600	3.51080400
P	0.26114600	-0.96225000	0.82150800
Fe	1.91922800	-3.28239000	-1.09392300
C	3.62480600	-2.11407800	-0.77296200
C	3.82295700	-3.43923300	-0.26216200
H	4.04893000	-3.69668000	0.76227800
C	3.67137600	-4.35985200	-1.34121900
C	3.37468300	-3.61594000	-2.52164700
C	3.32873000	-2.23540500	-2.17407400
H	3.10835300	-1.40531500	-2.83268800
C	4.26307500	-0.68480600	1.73113300
C	5.33578400	-1.53325600	2.03655800
H	5.79738400	-2.12756400	1.25431800
C	5.82574500	-1.60167400	3.34000500
H	6.65190600	-2.26806400	3.57186600
C	5.26829100	-0.80200000	4.34104500
H	5.65359600	-0.85508300	5.35533600
C	4.22188400	0.06850100	4.03480600
H	3.78219000	0.68943200	4.80933100
C	3.71811100	0.12411300	2.73663400
H	2.88362200	0.77673100	2.50227600
C	5.06047300	0.37620600	-0.74094100
C	5.17131000	1.75869900	-0.53343000
H	4.39167000	2.30001300	-0.00627600
C	6.27542700	2.45072400	-1.02463000
H	6.35547400	3.51965000	-0.85141300
C	7.26540400	1.77601600	-1.74366700
H	8.12163700	2.31934200	-2.13352000
C	7.15001500	0.40327600	-1.96401300
H	7.91489800	-0.12573300	-2.52573400
C	6.05312300	-0.29808900	-1.45923300
H	5.96984100	-1.36672800	-1.63022700
P	3.63173900	-0.47978200	0.02558200
H	0.52117400	-5.74504000	-0.86402700
H	3.16725500	-4.03025900	-3.49904800

H	3.73313800	-5.43740300	-1.26561600
S	-3.04720400	-2.63288900	-2.19234400
N	-1.36367600	-0.55258600	-2.17030200
H	-1.20073900	0.44204900	-2.02893500
N	-3.50936800	0.02922700	-1.80730100
H	-3.08922900	0.96419200	-1.86295000
F	-6.08014200	2.86219000	1.80171800
F	-6.32108900	3.79492800	-0.14703800
F	-8.03403500	2.88487800	0.84595600
F	-6.96579700	-2.74979400	1.24014100
F	-7.49493400	-3.09864700	-0.83819200
F	-8.80466200	-1.91263900	0.43627300
C	-0.17829300	-1.32540700	-2.54331300
H	0.63731600	-0.60850100	-2.39938700
C	-0.17028300	-1.70148900	-4.02808700
H	-0.99716300	-2.37579700	-4.26148400
H	0.77787200	-2.18209200	-4.29266500
H	-0.27606900	-0.79348300	-4.62785100
C	-2.63944300	-1.00380200	-2.05158100
C	-4.77178000	0.05032700	-1.20971800
C	-5.20897900	1.31030600	-0.76303100
H	-4.59004600	2.18359600	-0.94263500
C	-6.38814300	1.42640900	-0.03916800
C	-7.17761900	0.30987400	0.24196300
H	-8.08898400	0.40389000	0.81851700
C	-6.75720300	-0.92609600	-0.24095400
C	-5.57740900	-1.07030200	-0.97114900
H	-5.26337400	-2.04166400	-1.32206600
C	-6.71445700	2.74463900	0.59790800
C	-7.51261300	-2.16811500	0.13628900
Rh	1.47915700	0.50908600	-0.34317900
Cl	-2.20257800	2.99204600	-1.96252600
H	1.47073700	1.35315600	0.92684700
Cl	2.31722800	1.55805800	-2.39545000
H	0.08818500	1.21810000	-0.61511200
H	0.92436100	2.88836600	-1.42552900
C	-0.28574300	4.13345000	2.40100600
C	-0.46584200	3.96207600	1.02517600
C	0.63503600	4.10309000	0.19609300
C	1.90973900	4.43358800	0.67624100
C	2.07280300	4.58414100	2.05674600
C	0.98019600	4.43132900	2.91193100
H	-1.13448000	4.02499800	3.06885200
H	-1.42965900	3.71239500	0.59202900

H	3.05964200	4.83221800	2.43361400
H	1.11583300	4.55547300	3.98212200
C	2.79227400	4.77583500	-1.43338700
N	3.01628700	4.60777100	-0.18000400
N	0.54574700	3.84354800	-1.23608000
H	-0.47108600	3.75765900	-1.56448600
C	3.92284700	4.96554200	-2.39294200
H	4.05020500	4.04846800	-2.98223000
H	3.71268900	5.78397800	-3.09325200
H	4.84749200	5.17290800	-1.85272500
C	1.38507100	4.78306300	-2.01721700
H	1.38151200	4.44374100	-3.05348500
H	0.94189700	5.78460500	-1.96118800

9

C	0.30342400	-4.65897300	-0.54603000
C	0.09637200	-3.93338400	-1.74993900
H	-0.00886800	-4.35840000	-2.73673500
C	0.06519400	-2.54399200	-1.45299300
C	0.26018900	-2.40633200	-0.02856100
C	0.39736100	-3.72606800	0.52060000
H	0.55518300	-3.96443200	1.56130100
C	-1.47173800	-0.44803100	1.18636500
C	-1.90478500	0.87822600	1.28431800
H	-1.23558000	1.67442200	0.99238900
C	-3.19693300	1.16358600	1.72469100
H	-3.54231100	2.19165700	1.76971600
C	-4.06569000	0.12415200	2.05517100
H	-5.07986100	0.35003600	2.36684200
C	-3.65444500	-1.20299300	1.91490600
H	-4.35662400	-2.00830900	2.10492600
C	-2.35920400	-1.49252200	1.48764200
H	-2.04483100	-2.52465800	1.37611500
C	0.90991300	-1.14712300	2.53532600
C	1.90056800	-2.09999400	2.80548200
H	2.30181000	-2.70083600	2.00243200
C	2.38448800	-2.27437100	4.10090300
H	3.15591900	-3.01484900	4.28922000
C	1.90462400	-1.47976000	5.14108200
H	2.28812300	-1.60953600	6.14897600
C	0.93519800	-0.51048900	4.87791300
H	0.55538400	0.11402300	5.68175500
C	0.43600900	-0.34774600	3.58654100
H	-0.33050700	0.39590200	3.39696200
P	0.28526300	-0.81298200	0.83707400
Fe	1.88366700	-3.34386900	-0.88127600
C	3.59006100	-2.16846700	-0.69832500
C	3.77964200	-3.42535500	-0.03389700
H	4.00321200	-3.55965400	1.01418300
C	3.62145400	-4.46712000	-0.99546900
C	3.33366900	-3.86696300	-2.25733900
C	3.30001100	-2.45415000	-2.07623400
H	3.09146900	-1.70875900	-2.83256300
C	4.29956500	-0.46799500	1.59562000
C	5.37067600	-1.29216200	1.96702800
H	5.80053000	-1.98109700	1.24685400
C	5.90053300	-1.21814400	3.25454700
H	6.72494700	-1.86712400	3.53688400
C	5.38431800	-0.30104800	4.17357200
H	5.79979400	-0.24405800	5.17576600
C	4.33904900	0.54349700	3.79889900
H	3.92856600	1.25539900	4.50892400
C	3.79622900	0.45830200	2.51814800
H	2.96813300	1.09831600	2.23603000
C	5.06315100	0.24924300	-0.99851200
C	5.19661800	1.64386500	-1.01786400
H	4.40997600	2.25747200	-0.59302200
C	6.30948800	2.23330800	-1.61287400
H	6.40599400	3.31647900	-1.63129300
C	7.29316800	1.43788100	-2.20555900
H	8.15648900	1.89859900	-2.67736700
C	7.15946000	0.04916800	-2.19599200
H	7.91956400	-0.57468000	-2.65800200
C	6.04986700	-0.54520900	-1.59168600
H	5.95215600	-1.62605300	-1.58709400
P	3.61547600	-0.44894600	-0.10685100
H	0.40492400	-5.73258600	-0.45857400
H	3.12056200	-4.39249400	-3.17851300
H	3.67135000	-5.52846800	-0.79108800
S	-3.03249100	-2.68920000	-2.21226500
N	-1.33830600	-0.61778100	-2.14290000
H	-1.15892000	0.36528800	-1.94871900
N	-3.48066400	-0.04339800	-1.73047000
H	-3.05911600	0.89210000	-1.74041400
F	-6.21540600	2.92751600	1.61176800
F	-6.51779900	3.68679800	-0.40280700
F	-8.17246300	2.76696400	0.67571200
F	-6.82434400	-2.78317900	1.45422900

F	-7.35105800	-3.27333100	-0.59552200
F	-8.70144300	-2.07060000	0.61934500
C	-0.14618700	-1.41524200	-2.44369400
H	0.67235200	-0.69602600	-2.30972600
C	-0.08699400	-1.86201700	-3.90675100
H	-0.91299600	-2.53644700	-4.14047500
H	0.86317700	-2.36838400	-4.10708100
H	-0.15475700	-0.98260500	-4.55281400
C	-2.61453200	-1.06848500	-2.01632100
C	-4.75570900	-0.04142800	-1.15846900
C	-5.26081300	1.22434000	-0.81046200
H	-4.68176500	2.11210200	-1.04315300
C	-6.45132600	1.33461100	-0.10430100
C	-7.18997700	0.20484900	0.25115100
H	-8.11246200	0.29595100	0.81002000
C	-6.70237500	-1.04075300	-0.13379700
C	-5.50661900	-1.17975200	-0.83819500
H	-5.14007600	-2.15828800	-1.10873800
C	-6.84747200	2.67962600	0.42848400
C	-7.40125300	-2.28880600	0.32368200
Rh	1.46067400	0.58985000	-0.45805100
Cl	-2.21710000	2.95968500	-1.62724600
H	1.52176900	1.47283500	0.77817300
Cl	2.23634200	1.63835500	-2.49979900
H	0.09881500	1.34505900	-0.68737100
H	-0.52808400	3.76614400	-1.18611000
C	1.77352000	3.80084800	2.95470600
C	2.69896100	4.02974800	1.93699200
C	2.26131100	4.20031000	0.61653100
C	0.88098700	4.10348800	0.35863100
C	-0.04530600	3.90525300	1.37865600
C	0.40095500	3.75159600	2.68914800
H	2.13263200	3.66892300	3.97126000
H	3.76240900	4.06748600	2.15668800
H	-1.09839400	3.85332300	1.12187600
H	-0.31173000	3.58726900	3.48982400
C	1.22646200	4.38762100	-1.99018400
N	0.45500600	4.13341100	-0.98410700
N	3.11704300	4.38609900	-0.44612700
H	4.08861200	4.55971400	-0.23516200
C	0.66696500	4.42685300	-3.36970700
H	0.60334300	5.47458300	-3.69763700
H	1.32878800	3.89187900	-4.05363400
H	-0.32976800	3.98067500	-3.39506300
C	2.64129600	4.84002500	-1.73872000
H	2.65544500	5.94390900	-1.84041100
H	3.27586800	4.42597100	-2.52671000

12

C	-1.16209500	-3.89809300	-1.61391100
C	-0.83906600	-3.98632500	-0.23413500
H	-0.85583500	-4.88620700	0.36241300
C	-0.51990400	-2.69197300	0.25978700
C	-0.66198700	-1.76666100	-0.84993000
C	-1.04036600	-2.54297900	-2.00541600
H	-1.20044800	-2.15675700	-2.99907800
C	1.38680600	0.27006700	-1.17339000
C	1.97994800	1.53035600	-0.99788700
H	1.43151100	2.32027000	-0.49933300
C	3.28218500	1.75577700	-1.44100800
H	3.74688000	2.72196300	-1.27702300
C	4.00422700	0.72543300	-2.04675400
H	5.02878100	0.88831600	-2.36354600
C	3.42986600	-0.53619200	-2.19124900
H	4.01107600	-1.35044200	-2.61127300
C	2.12184700	-0.76757200	-1.76506500
H	1.68639600	-1.75218400	-1.87897300
C	-1.09939600	0.62819100	-2.47426100
C	-2.26386800	0.09065500	-3.03765600
H	-2.76742000	-0.72612100	-2.54793200
C	-2.78704900	0.59571100	-4.22636900
H	-3.69412300	0.16245800	-4.63669000
C	-2.17001900	1.67122900	-4.86140600
H	-2.58218200	2.07405200	-5.78183000
C	-1.02108700	2.23027000	-4.30055200
H	-0.52755800	3.06756400	-4.78531600
C	-0.48566700	1.71105200	-3.12281700
H	0.42061400	2.14684300	-2.72162700
P	-0.39643700	0.03253500	-0.86995300
Fe	-2.44715700	-2.68782300	-0.48457700
C	-3.81131600	-1.25595700	0.11431100
C	-4.30423500	-1.95290800	-1.04420900
H	-4.56285700	-1.50773000	-1.99255000
C	-4.40865300	-3.33602500	-0.72045500
C	-3.99062100	-3.50768100	0.63213200
C	-3.61737000	-2.23517200	1.14792700
H	-3.24150600	-2.01568300	2.13749400
C	-4.18028900	1.41158700	-1.04594300

C	-5.43620000	1.04050600	-1.54970200
H	-5.96190400	0.19014500	-1.12890900
C	-6.02618000	1.77529200	-2.57611300
H	-6.99510900	1.47487200	-2.96460300
C	-5.38297700	2.90381700	-3.08975100
H	-5.84488600	3.47655100	-3.88871900
C	-4.15383500	3.30062400	-2.56362100
H	-3.64953500	4.17977900	-2.95237400
C	-3.55588400	2.55914900	-1.54640900
H	-2.59067800	2.85997400	-1.15651800
C	-4.63896100	1.02294800	1.67798400
C	-4.46679500	2.28711200	2.25770300
H	-3.60225400	2.89073600	1.99619600
C	-5.38574000	2.75683300	3.19116300
H	-5.24008100	3.73210600	3.64591200
C	-6.48078300	1.96775500	3.55387000
H	-7.19292000	2.33161400	4.28879200
C	-6.65423400	0.71059800	2.97623600
H	-7.50167800	0.09230800	3.25833000
C	-5.73713400	0.23646000	2.03605500
H	-5.87410900	-0.74500500	1.59493800
P	-3.45503600	0.51006600	0.37828900
H	-1.47243700	-4.71466000	-2.25202500
H	-3.92055700	-4.44770700	1.16257000
H	-4.71802200	-4.12075400	-1.39789900
S	2.70397900	-3.14666700	0.70151500
N	0.94445400	-1.49323800	1.84405800
H	0.73322500	-0.61007700	2.30210100
N	3.00113100	-0.60611600	1.67493200
H	2.46711200	0.19890600	2.00404100
F	5.53697700	3.61437500	2.18906100
F	7.43853400	3.32678600	1.16267100
F	5.64365900	3.77389800	0.02395600
F	6.77784100	-1.30638500	-2.12920600
F	7.15761700	-2.69996500	-0.50414100
F	8.50293600	-1.01815200	-0.83566300
C	-0.20002300	-2.38401300	1.70742000
H	-1.01254000	-1.77357600	2.09551300
C	-0.12271200	-3.62437000	2.60332500
H	0.65643000	-4.31462700	2.27694300
H	-1.09033200	-4.13897600	2.60826500
H	0.09419800	-3.30506900	3.62639900
C	2.21741900	-1.70954700	1.42829800
C	4.26932900	-0.23533500	1.22082400
C	4.61906600	1.10869800	1.43764800
H	3.92712400	1.76603300	1.95406800
C	5.82168800	1.61123900	0.95751600
C	6.70518100	0.79725500	0.24808700
H	7.63524500	1.19298500	-0.14027500
C	6.35847100	-0.53731400	0.05467600
C	5.16810100	-1.07024700	0.54641200
H	4.91374000	-2.10461700	0.37260000
C	6.11374800	3.07795700	1.09546000
C	7.20697200	-1.39646200	-0.83827500
Rh	-1.27461400	0.97763400	0.98007300
Cl	0.74837400	1.81588200	1.99471000
H	-1.16304300	2.17662900	0.01806900
Cl	-1.90236300	0.19903400	3.19011200

Transition States

TS1

C	-1.11038200	3.73008400	1.78046200
C	-0.80934200	3.85280500	0.39643000
H	-0.80852400	4.77067400	-0.17291000
C	-0.55307700	2.55888500	-0.13759800
C	-0.69252700	1.61152200	0.94681000
C	-1.02463800	2.35677300	2.12837500
H	-1.19696700	1.93692900	3.10762700
C	1.34733300	-0.41755900	1.11146500
C	2.10004600	0.56498800	1.76978500
H	1.66506400	1.53392600	1.98394900
C	3.42163800	0.30545300	2.13290900
H	4.01275000	1.07988100	2.61123100
C	3.99618900	-0.93502100	1.85743600
H	5.03018300	-1.12105400	2.12849200
C	3.26074300	-1.90841900	1.18012500
H	3.72473400	-2.85336800	0.91702700
C	1.94339400	-1.64991500	0.79883100
H	1.38203100	-2.39587900	0.24765300
C	-1.14589500	-0.89878200	2.36337400
C	-2.31634700	-0.39897900	2.94929200
H	-2.80301200	0.46065800	2.51757600
C	-2.86188000	-0.99504200	4.08414800
H	-3.77215000	-0.58957500	4.51588800
C	-2.25810900	-2.12005300	4.64387500
H	-2.68653600	-2.59185900	5.52333000
C	-1.10078900	-2.63677600	4.06122100

H	-0.61708000	-3.51056800	4.48857000
C	-0.54527300	-2.03046400	2.93447100
H	0.36702600	-2.43469400	2.51271800
P	-0.44467700	-0.18366400	0.80770900
Fe	-2.45227800	2.61359700	0.63171300
C	-3.85111400	1.28690800	-0.10606700
C	-4.32568400	1.85121100	1.12756300
H	-4.58804200	1.29795900	2.01629300
C	-4.40086000	3.26556500	0.97577400
C	-3.98821600	3.59239100	-0.34940800
C	-3.64245600	2.38089400	-1.01380900
H	-3.27461000	2.28850200	-2.02635700
C	-4.26600300	-1.49238100	0.72771900
C	-5.50050100	-1.21280400	1.32978600
H	-6.07000500	-0.34098600	1.02315400
C	-6.00870300	-2.06413600	2.30940500
H	-6.96243600	-1.83898000	2.77839000
C	-5.30107100	-3.21224300	2.67529100
H	-5.69967600	-3.87446800	3.43854300
C	-4.08855800	-3.51214700	2.05410200
H	-3.53532700	-4.40288800	2.33455400
C	-3.57184200	-2.65500800	1.08423300
H	-2.62082700	-2.87172600	0.60710400
C	-4.89879800	-0.66439900	-1.88815400
C	-4.93246700	-1.85830200	-2.62357500
H	-4.15819700	-2.60529000	-2.47862200
C	-5.95548000	-2.09829400	-3.53821400
H	-5.96466300	-3.02622800	-4.10257000
C	-6.96046100	-1.14768600	-3.73157400
H	-7.75441200	-1.33169500	-4.44953900
C	-6.93993400	0.03731400	-2.99742000
H	-7.71985600	0.78040000	-3.13806100
C	-5.91860000	0.27737100	-2.07585800
H	-5.91945200	1.19988200	-1.50705400
P	-3.58113000	-0.44521800	-0.61254500
H	-1.38691900	4.53649900	2.44651000
H	-3.89773900	4.58828300	-0.76198000
H	-4.68600100	3.96886200	1.74680800
S	2.63359400	3.16993100	-0.69122900
N	0.91911300	1.42583400	-1.77489600
H	0.73896300	0.48796400	-2.13117900
N	3.01605800	0.64468600	-1.67611900
H	2.51900200	-0.16698000	-2.04020200
F	5.86438300	-3.67697100	-0.30824900
F	5.78194800	-3.38961600	-2.46102300
F	7.65336100	-3.07919900	-1.38677800
F	6.74818200	1.31941400	2.16309300
F	7.07007200	2.81776400	0.62098100
F	8.49089300	1.18443100	0.86813300
C	-0.28126800	2.23197900	-1.59191200
H	-1.07102000	1.53964300	-1.91120500
C	-0.32461800	3.45301500	-2.51556200
H	0.46147900	4.16546100	-2.25868300
H	-1.29879800	3.94888200	-2.44464600
H	-0.17622900	3.12793900	-3.54936600
C	2.18821600	1.71018000	-1.39559300
C	4.30572600	0.31770400	-1.25303000
C	4.72793300	-0.98783300	-1.55586200
H	4.07314600	-1.64746400	-2.11642500
C	5.95428700	-1.45636100	-1.10286400
C	6.79342000	-0.64326600	-0.34058000
H	7.74326800	-1.01274800	0.02478300
C	6.37529900	0.65552900	-0.06276500
C	5.15708300	1.15377600	-0.52100000
H	4.84543500	2.15893400	-0.28042100
C	6.31769700	-2.89603700	-1.32607800
C	7.17821700	1.50130000	0.88363100
Rh	-1.48900300	-1.01786100	-1.26341100
Cl	0.60279600	-1.74802300	-2.25776900
H	-2.13144500	-2.02280700	-2.29343200
H	-1.97638600	-0.84959700	-2.75019300

TS2-C1

C	0.56168400	-4.51670200	0.85152500
C	0.30412500	-4.28900900	-0.52596800
H	0.18939100	-5.04900400	-1.28406100
C	0.21424700	-2.89137200	-0.75968300
C	0.45987900	-2.23323000	0.50197700
C	0.64922200	-3.25691100	1.49824900
H	0.82444000	-3.09739300	2.54993800
C	-1.33044800	-0.04836300	0.96791700
C	-1.86744100	1.15329100	0.50988800
H	-1.27508700	1.77366700	-0.13981100
C	-3.16743700	1.52082100	0.85795600
H	-3.60178700	2.43337900	0.46349800
C	-3.93422500	0.68212000	1.66310000
H	-4.95372000	0.95711800	1.91157200
C	-3.41966300	-0.54689500	2.08365200

H	-4.04756800	-1.22939800	2.64673600
C	-2.12194600	-0.91642600	1.73951000
H	-1.72931400	-1.87422200	2.06241600
C	1.03895900	-0.13658800	2.47351200
C	2.00810200	-0.92000400	3.11154900
H	2.43850000	-1.77071500	2.60358000
C	2.44322900	-0.60162100	4.39786600
H	3.19824300	-1.21902900	4.87454600
C	1.94257000	0.52426700	5.04836300
H	2.29021900	0.77794200	6.04550100
C	1.00260000	1.33266100	4.40502700
H	0.61358800	2.21935000	4.89716500
C	0.54722300	1.00266900	3.13090100
H	-0.19485900	1.63146800	2.65055600
P	0.43898100	-0.44333900	0.76115200
Fe	2.08728900	-3.37071900	0.02113500
C	3.75167200	-2.15944800	-0.18050700
C	3.97391400	-3.14728600	0.83896700
H	4.18582400	-2.95042700	1.87937100
C	3.85133300	-4.43529700	0.24047500
C	3.55019500	-4.25702300	-1.14255300
C	3.47359000	-2.86003600	-1.40634200
H	3.23346700	-2.38355000	-2.34690500
C	4.40231600	0.17727100	1.51599800
C	5.47351600	-0.49952600	2.11628900
H	5.87823200	-1.39526000	1.65719700
C	6.03857400	-0.01126200	3.29388600
H	6.86272600	-0.54623900	3.75716000
C	5.56008400	1.17007500	3.86520100
H	6.00385600	1.54910100	4.78129800
C	4.51841100	1.86550300	3.25066900
H	4.13856100	2.78371200	3.68834100
C	3.94148000	1.37180200	2.08323700
H	3.11725200	1.90338500	1.62264800
C	5.07039800	0.21364500	-1.19706400
C	5.23936900	1.59857300	-1.34025600
H	4.56928400	2.28553400	-0.83022800
C	6.25903700	2.09793400	-2.14502200
H	6.38069500	3.17171800	-2.25529700
C	7.11627800	1.22091600	-2.81519500
H	7.90793500	1.61111900	-3.44815700
C	6.95164700	-0.15605600	-2.67035900
H	7.61546400	-0.84226200	-3.18844500
C	5.93191100	-0.66260500	-1.86183700

H	5.80729900	-1.73453100	-1.75790900
P	3.71560000	-0.35178000	-0.09832700
H	0.70079700	-5.47957400	1.32477800
H	3.36006200	-5.04527400	-1.85846200
H	3.93116700	-5.38161400	0.75864100
S	-2.88561100	-3.38436400	-1.10157800
N	-1.32199100	-1.39317100	-1.97824700
H	-1.22595100	-0.43464200	-2.31063200
N	-3.49854000	-0.81548700	-1.76200000
H	-3.13753900	0.06171800	-2.16338700
F	-6.22761000	3.16854200	0.10248500
F	-6.81145200	2.94120300	-1.97876800
F	-8.25277300	2.54425900	-0.39400300
F	-6.56883700	-2.01958800	2.49826200
F	-7.02864100	-3.44739800	0.92541300
F	-8.49621100	-1.94139600	1.49537200
C	-0.11500600	-2.22212900	-2.07726500
H	0.67200500	-1.49772300	-2.28300500
C	-0.14705300	-3.18816700	-3.26444000
H	-0.94975500	-3.92074100	-3.16119000
H	0.81331300	-3.70959700	-3.34522700
H	-0.30406800	-2.61877600	-4.18467800
C	-2.57075300	-1.81453100	-1.62973400
C	-4.75853700	-0.64230600	-1.18136500
C	-5.33615900	0.62653900	-1.36587100
H	-4.82381900	1.35812600	-1.98267200
C	-6.50238900	0.97195700	-0.69486500
C	-7.14822000	0.06615900	0.14702600
H	-8.04945300	0.34407200	0.67827800
C	-6.59417900	-1.20416500	0.28579100
C	-5.42130700	-1.57426100	-0.37102400
H	-4.99860000	-2.55666500	-0.22695200
C	-6.96000900	2.39929000	-0.75546700
C	-7.17939500	-2.15852900	1.28664600
Rh	1.68181700	0.58271900	-0.81042500
Cl	-2.38413200	1.98863800	-2.81660000
H	0.47445500	1.52867200	-1.48225800
H	1.65621400	1.60362200	0.33790900
Cl	2.32435500	0.12400800	-3.10535700
C	1.91765000	3.54016600	-1.84181000
C	1.30791300	4.19739300	0.28541100
C	-0.07492200	4.08983900	-0.02212100
C	0.54713300	3.04382000	-2.04337300
H	2.74766100	4.63744900	1.80920700

C	1.68736000	4.55358000	1.59342500
C	-1.04905900	4.39126600	0.94279100
H	-1.36597900	3.39015100	-1.57245300
H	0.20717600	2.69545700	-3.01272200
C	-0.64532800	4.72565800	2.22536000
C	0.72272400	4.79296100	2.55739800
H	-2.10028200	4.31854600	0.68593700
H	-1.39538200	4.93208900	2.98236300
H	1.01848800	5.05454100	3.56847200
N	-0.39687100	3.64499000	-1.28575600
N	2.26968100	4.05860600	-0.69891200
C	2.88238600	3.52573500	-2.98603300
H	3.10299900	2.50256800	-3.30412300
H	2.44637700	4.04878500	-3.84624700
H	3.80367300	4.02916100	-2.68942600

TS2-C2

C	0.42342000	-4.46667200	0.70641300
C	0.22604200	-4.18971500	-0.67232200
H	0.11621900	-4.92307400	-1.45676600
C	0.18049700	-2.78431700	-0.86395500
C	0.40091200	-2.17420000	0.42527300
C	0.52043900	-3.23068000	1.39695400
H	0.65800700	-3.10827700	2.45919400
C	-1.29477500	0.09388500	0.88338900
C	-1.73166200	1.37630600	0.55522600
H	-1.08064900	2.03668200	0.00566500
C	-3.01251900	1.79268300	0.92123600
H	-3.36937700	2.77152500	0.62101700
C	-3.85291400	0.93149200	1.62125600
H	-4.85360700	1.25540600	1.88477100
C	-3.43359600	-0.36879700	1.91944700
H	-4.11501800	-1.05967500	2.40493000
C	-2.16141100	-0.79338100	1.54827600
H	-1.84509000	-1.80660300	1.76816800
C	1.02352600	-0.12495000	2.43567100
C	1.91385600	-0.97645600	3.09945700
H	2.30435000	-1.84802800	2.59617600
C	2.31850200	-0.70255700	4.40587900
H	3.01242900	-1.37379800	4.90227700
C	1.86322500	0.44322500	5.05421500
H	2.18585800	0.66151100	6.06792700
C	0.99949800	1.31598900	4.38848600
H	0.64461500	2.21677700	4.88069300
C	0.57647500	1.03315500	3.09232700
H	-0.10952100	1.71139100	2.59651200
P	0.45194200	-0.39195400	0.70602700
Fe	2.01569100	-3.34322300	-0.01889200
C	3.72657700	-2.18202800	-0.12413300
C	3.85856500	-3.17520500	0.90723900
H	4.01894500	-2.98417200	1.95772700
C	3.73240900	-4.45981400	0.30369700
C	3.51979600	-4.27573600	-1.09447900
C	3.50261000	-2.87884400	-1.36404000
H	3.33076200	-2.40088300	-2.31735500
C	4.35816400	0.12539700	1.63041000
C	5.38036100	-0.58712400	2.27327400
H	5.77407000	-1.49550900	1.82988400
C	5.91243400	-0.11685100	3.47307700
H	6.69865300	-0.67832900	3.96959200
C	5.44945800	1.07974700	4.02459300
H	5.86693800	1.44493700	4.95844800
C	4.45791900	1.80933500	3.36785900
H	4.09261900	2.74083800	3.78928700
C	3.91457800	1.33608500	2.17605100
H	3.13329500	1.89851800	1.67862700
C	5.17043700	0.17690900	-1.03657000
C	5.39013700	1.55864000	-1.14336200
H	4.72389400	2.26207900	-0.65152800
C	6.46166600	2.03596400	-1.89304600
H	6.62245800	3.10689900	-1.97733900
C	7.32157000	1.14187500	-2.53710400
H	8.15413200	1.51590500	-3.12581200
C	7.10867900	-0.23119700	-2.42154100
H	7.77566100	-0.93055200	-2.91746400
C	6.03517500	-0.71712700	-1.67254000
H	5.87047800	-1.78578100	-1.59468600
P	3.74753300	-0.37355700	-0.02108500
H	0.51436900	-5.44751500	1.15365300
H	3.34861800	-5.05905800	-1.82059500
H	3.75058100	-5.40606400	0.82782300
S	-2.91002400	-3.31687000	-1.30990700
N	-1.36347200	-1.25304000	-2.02922200
H	-1.27313300	-0.26587100	-2.25889200
N	-3.53309400	-0.69542100	-1.69476300
H	-3.19040900	0.21896500	-2.02211500
F	-6.35229000	3.07420800	0.48900300
F	-6.93084700	2.98862900	-1.60444700

F	-8.36014800	2.43463800	-0.05631400
F	-6.44992600	-2.35685000	2.46242100
F	-7.03825300	-3.59249100	0.77523400
F	-8.43375400	-2.13569100	1.59772800
C	-0.13739500	-2.05429900	-2.15358800
H	0.63958200	-1.30536400	-2.30638300
C	-0.12610000	-2.95775100	-3.38885600
H	-0.91451800	-3.71120600	-3.34079100
H	0.84807400	-3.45198900	-3.47407300
H	-0.27712200	-2.34615200	-4.28267400
C	-2.60525500	-1.70195800	-1.68632900
C	-4.79279700	-0.59367400	-1.09279600
C	-5.40442700	0.66962100	-1.18097500
H	-4.91911400	1.45683800	-1.74869700
C	-6.56878400	0.93704100	-0.47242200
C	-7.17830200	-0.04028800	0.31549700
H	-8.07690200	0.17715500	0.87811000
C	-6.59154000	-1.30180700	0.35760600
C	-5.41964600	-1.59487400	-0.33998800
H	-4.97332500	-2.57499400	-0.27170300
C	-7.06354200	2.35279300	-0.42561600
C	-7.13732900	-2.34889600	1.28516900
Rh	1.78571900	0.58530100	-0.83410700
Cl	-2.59289700	2.25665600	-2.54496900
H	0.61556800	1.56761500	-1.67278400
H	1.68536900	1.59851600	0.31942800
Cl	2.63174800	0.00197000	-3.05098200
H	-1.03953700	3.52840800	-1.87025800
C	0.85920900	2.90358000	-2.25589400
C	0.23827600	4.18938700	-0.34325200
C	1.59646700	4.20750200	0.06924200
C	2.26507200	3.20098400	-1.88146900
H	-1.79866300	4.65500900	0.18093000
C	-0.76617900	4.66340300	0.51579400
C	1.91796300	4.66830500	1.35695400
H	3.04312000	2.87133000	-2.56518100
C	0.91620600	5.08945800	2.21846100
C	-0.42404900	5.09521300	1.78858900
H	2.96446100	4.67934600	1.64529200
H	1.16495200	5.43285800	3.21758300
H	-1.20451600	5.43928400	2.46032500
N	2.61197900	3.81759900	-0.79671200
N	-0.04986400	3.70117000	-1.59876300
C	0.57948400	2.63396900	-3.71655200
H	0.77171000	3.55908100	-4.27377600
H	1.24124100	1.84900000	-4.08175700
H	-0.46424600	2.34761800	-3.85543400

TS3-N1

C	0.30674600	-4.65833100	0.46948500
C	0.05722200	-4.27046000	-0.87327600
H	-0.06720600	-4.93896500	-1.71152100
C	-0.00102800	-2.85350700	-0.94870200
C	0.24516800	-2.34969900	0.38396600
C	0.41459600	-3.48309600	1.25579000
H	0.59057600	-3.44647000	2.31901500
C	-1.44886100	-0.11880100	1.06517200
C	-1.87476300	1.19116000	0.83308800
H	-1.21109700	1.89622500	0.36159700
C	-3.16155900	1.58534600	1.19555600
H	-3.50204400	2.59362600	0.98316700
C	-4.03055000	0.66824500	1.78390000
H	-5.04007400	0.97204700	2.03871300
C	-3.62646200	-0.65418100	1.97791900
H	-4.32980400	-1.38157100	2.36998300
C	-2.33903200	-1.05216900	1.62255700
H	-2.03401600	-2.08272700	1.76410600
C	0.91798900	-0.50664100	2.57108500
C	1.88227400	-1.37995500	3.09105300
H	2.28533800	-2.17034000	2.47462400
C	2.34305700	-1.22727700	4.39759900
H	3.09185200	-1.91170600	4.78392000
C	1.87370100	-0.17939200	5.18827100
H	2.24176600	-0.05460400	6.20234500
C	0.94041000	0.71739400	4.66526700
H	0.57718700	1.54401800	5.26870000
C	0.45862500	0.55309300	3.36828400
H	-0.27572200	1.24893800	2.97791400
P	0.29445600	-0.60483800	0.84834500
Fe	1.85329800	-3.45035400	-0.23316700
C	3.53535900	-2.25310200	-0.34319600
C	3.76129600	-3.33947700	0.56821400
H	4.00405800	-3.24815400	1.61630700
C	3.60381900	-4.55968000	-0.15121200
C	3.28093600	-4.24151900	-1.50376200
C	3.22454200	-2.82445100	-1.62655700
H	2.98595400	-2.25503600	-2.51501800
C	4.24837900	-0.10386800	1.56492200

C	5.31331300	-0.84998900	2.09134500
H	5.71909500	-1.68803100	1.53445900
C	5.87213000	-0.50104600	3.31945800
H	6.69067600	-1.08890500	3.72487300
C	5.39622000	0.61276100	4.01583500
H	5.83695300	0.88506300	4.97053600
C	4.36353500	1.37958000	3.47657900
H	3.98708800	2.24930700	4.00584600
C	3.79026700	1.02222600	2.25850300
H	2.97810700	1.61643100	1.85891400
C	5.02179800	0.08990600	-1.11395300
C	5.32218600	1.45954400	-1.11662900
H	4.68036300	2.16951200	-0.60228600
C	6.44292800	1.92007400	-1.80222000
H	6.67196600	2.98169700	-1.79993000
C	7.26390400	1.02653700	-2.49479600
H	8.13443800	1.39119600	-3.03244600
C	6.96191200	-0.33451300	-2.49480700
H	7.59497600	-1.03515100	-3.03173400
C	5.84509000	-0.80643200	-1.80218900
H	5.62040400	-1.86674800	-1.80387100
P	3.59381600	-0.45824200	-0.10558200
H	0.42647300	-5.67160400	0.82907900
H	3.06268100	-4.95208800	-2.28955100
H	3.67758100	-5.55389200	0.26904800
S	-3.11076000	-3.14552800	-1.30444500
N	-1.45065300	-1.16940200	-2.02985200
H	-1.32743500	-0.20042600	-2.33008800
N	-3.59122700	-0.50523600	-1.78719200
H	-3.16336300	0.38157700	-2.09610300
F	-6.09831900	3.34858400	0.67989500
F	-6.47755500	3.55851800	-1.45064000
F	-8.10348900	3.02730400	-0.10109500
F	-6.93088700	-2.17943500	2.05119700
F	-7.54930000	-3.16416400	0.21622300
F	-8.81221000	-1.62936900	1.10852700
C	-0.28572100	-2.04009600	-2.19631600
H	0.53849900	-1.33543500	-2.32965400
C	-0.35154600	-2.88504800	-3.47242200
H	-1.19021100	-3.58338000	-3.43720700
H	0.58199300	-3.44320800	-3.60518300
H	-0.47964500	-2.22258900	-4.33281400
C	-2.72041600	-1.55871700	-1.72133200
C	-4.84940600	-0.30615800	-1.21522400

C	-5.29791900	1.02740400	-1.20597800
H	-4.68666200	1.79459200	-1.67163900
C	-6.46602200	1.36926900	-0.53785300
C	-7.23503100	0.40372800	0.11491500
H	-8.13678200	0.67739900	0.64732700
C	-6.80500700	-0.91908700	0.06240800
C	-5.63549300	-1.28961200	-0.60182500
H	-5.31266200	-2.31951900	-0.61174100
C	-6.79641900	2.82285800	-0.36935800
C	-7.53258200	-1.97306600	0.84673400
Rh	1.54174500	0.48084800	-0.71000000
Cl	-2.08911300	2.16803100	-2.56190000
H	1.64432400	1.45296600	0.46599300
Cl	2.30250800	0.53949800	-2.98366400
H	0.06710800	1.11569300	-1.29757200
H	0.38086700	1.95462400	-1.18149200
C	0.17252400	4.38824500	2.41631400
C	-0.26182200	4.14429000	1.11114500
C	0.67272800	3.97743500	0.08825900
C	2.05284200	4.09278200	0.36171200
C	2.46953100	4.33835400	1.67242300
C	1.53693900	4.47543400	2.70218600
H	-0.56218400	4.51587000	3.20579600
H	-1.32180800	4.08326300	0.88498200
H	3.53599500	4.42042800	1.85849700
H	1.87193900	4.66663300	3.71713900
C	2.64064200	3.92879700	-1.87611900
N	3.02694600	3.96406800	-0.64972600
N	0.31983600	3.54894600	-1.20189300
H	-0.67588000	3.54709900	-1.45728900
C	3.64637600	3.87763300	-2.98272800
H	3.66232000	2.87183600	-3.41809800
H	3.37139400	4.57798700	-3.78187200
H	4.64046200	4.12332700	-2.60773100
C	1.18652000	3.98055900	-2.29865100
H	1.01301400	3.31023200	-3.14496100
H	0.94358100	5.00539400	-2.62779900

TS3-N2

C	0.00326700	-4.68024500	-0.02136000
C	-0.21892600	-4.13965100	-1.31497400
H	-0.37700100	-4.70586100	-2.22012100
C	-0.20146700	-2.72198800	-1.23741500
C	0.05703700	-2.37500600	0.14068300

C	0.16774700	-3.60172400	0.88520800
H	0.34521900	-3.68615800	1.94561900
C	-1.52783100	-0.11737600	1.06119300
C	-1.88709500	1.22922300	0.93310200
H	-1.22181500	1.93302400	0.45482900
C	-3.14199600	1.66020200	1.36458400
H	-3.43056400	2.69727700	1.23043500
C	-4.04357900	0.74995900	1.91327500
H	-5.02698200	1.08789500	2.22133500
C	-3.71041800	-0.60397500	1.99342700
H	-4.44273000	-1.32096200	2.34992500
C	-2.45614000	-1.04043300	1.57207900
H	-2.20357700	-2.09367900	1.62752000
C	0.79119000	-0.83467400	2.51408800
C	1.74640900	-1.78249300	2.90474800
H	2.13281100	-2.48782200	2.18377800
C	2.21387900	-1.82123300	4.21700200
H	2.95761700	-2.56038400	4.49763600
C	1.75770700	-0.89204000	5.15063500
H	2.12983300	-0.91525600	6.17067900
C	0.82312100	0.07107100	4.76634200
H	0.45765200	0.79616400	5.48799100
C	0.33604600	0.09642500	3.46038300
H	-0.41061300	0.83156400	3.18119600
P	0.18131600	-0.68911600	0.78626400
Fe	1.61492200	-3.49394300	-0.59682500
C	3.37554200	-2.40471500	-0.58015800
C	3.52152600	-3.59914400	0.20476200
H	3.75973800	-3.64517000	1.25677200
C	3.28746900	-4.71716000	-0.64801500
C	2.99424300	-4.22717300	-1.95541800
C	3.03533300	-2.80519100	-1.91806600
H	2.83678300	-2.12277300	-2.73373700
C	4.18811100	-0.52448500	1.54686400
C	5.19545900	-1.39834500	1.98156300
H	5.53858900	-2.19861100	1.33481300
C	5.77792500	-1.22881300	3.23650800
H	6.55045000	-1.91668900	3.56844200
C	5.38291900	-0.16829000	4.05572500
H	5.83848600	-0.03773300	5.03316900
C	4.41212700	0.72764000	3.60863400
H	4.10081000	1.55990100	4.23136900
C	3.81725000	0.55145200	2.36153300
H	3.05579800	1.24893900	2.03520500
C	4.91743400	-0.01621200	-1.09805500
C	5.10561200	1.37223000	-1.14016800
H	4.38051400	2.03831200	-0.68444100
C	6.20873800	1.90409200	-1.79967300
H	6.33758500	2.98194600	-1.84038400
C	7.12848500	1.05792100	-2.42573200
H	7.98446600	1.47497000	-2.94860700
C	6.94111000	-0.32335700	-2.38493800
H	7.65048900	-0.98499900	-2.87386600
C	5.83801800	-0.86339500	-1.71954000
H	5.69225100	-1.93846500	-1.69613800
P	3.49039200	-0.65280500	-0.14245000
H	0.06997100	-5.73076600	0.22836500
H	2.73112500	-4.82798600	-2.81558300
H	3.29016000	-5.75504700	-0.34276400
S	-3.31030200	-2.89381100	-1.60648900
N	-1.59346600	-0.89150700	-2.11813300
H	-1.44293800	0.11138900	-2.27533700
N	-3.70730200	-0.20225100	-1.77157500
H	-3.24875000	0.69829600	-1.99549600
F	-6.07152300	3.49523400	1.07125800
F	-6.48041700	3.88034000	-1.02897300
F	-8.09479500	3.27490100	0.30310900
F	-7.01183100	-2.06008200	2.04970100
F	-7.62605400	-2.94651800	0.16346600
F	-8.88832200	-1.45787200	1.13125200
C	-0.44989500	-1.76227900	-2.38291700
H	0.39407600	-1.06814800	-2.43013800
C	-0.52761200	-2.45294100	-3.74832300
H	-1.38691300	-3.12519700	-3.79954500
H	0.39078000	-3.02058200	-3.93692400
H	-0.62809400	-1.69252000	-4.52747500
C	-2.86772200	-1.28122300	-1.83896800
C	-4.94216700	-0.02478100	-1.15101300
C	-5.35737700	1.31366900	-1.01916900
H	-4.73754600	2.10193000	-1.43606800
C	-6.49972900	1.62559600	-0.29412100
C	-7.27445300	0.62714600	0.29966800
H	-8.15603800	0.87616800	0.87621400
C	-6.87695000	-0.69638400	0.12992200
C	-5.73496600	-1.03745200	-0.59468000
H	-5.43620600	-2.06967100	-0.69812000
C	-6.79580200	3.06700900	-0.00333800
C	-7.60810000	-1.78950700	0.85437800

Rh	1.50379200	0.48361100	-0.62428700
Cl	-2.01946400	2.36589500	-2.28747700
H	1.68260600	1.26849800	0.67007300
Cl	2.30303300	0.77704600	-2.86611200
H	0.12987000	1.40146900	-1.06536000
H	0.55696500	2.25815900	-0.94396000
C	3.14646100	4.08202100	2.84295100
C	3.78417500	4.46212600	1.66255300
C	3.11260600	4.35911200	0.43908800
C	1.80381400	3.83156100	0.41202600
C	1.17126500	3.46546000	1.60316900
C	1.83622900	3.59546400	2.82231600
H	3.67596200	4.18024400	3.78602500
H	4.79925100	4.85039100	1.68534300
H	0.15935600	3.08018300	1.56111700
H	1.34019200	3.30748000	3.74298100
C	1.60153000	4.16890400	-1.90190100
N	1.17769500	3.58854800	-0.83130800
N	3.68205700	4.69446600	-0.78458100
H	4.53977300	5.22870100	-0.72353800
C	0.98329600	3.92423200	-3.23174000
H	0.79494600	4.88885700	-3.72310800
H	1.68551400	3.36220500	-3.85812200
H	0.04897200	3.36448800	-3.14107000
C	2.75167300	5.15526600	-1.80890100
H	2.34048000	6.16211900	-1.60077600
H	3.26900900	5.19831700	-2.77021400

TS3-Cl

C	-1.11289000	3.96316100	-1.23373200
C	-0.78110600	3.09313300	-2.30626400
H	-0.75078600	3.36099800	-3.35171000
C	-0.52547400	1.79322800	-1.78915300
C	-0.70298000	1.86060900	-0.35366100
C	-1.05806100	3.21667500	-0.02934700
H	-1.25634000	3.59612000	0.96081700
C	1.30320100	0.45252900	1.19875500
C	1.90272200	-0.72282600	1.67498800
H	1.33502100	-1.64711500	1.68946900
C	3.22377400	-0.70392600	2.12412400
H	3.69318900	-1.61789000	2.47286000
C	3.95533100	0.48403600	2.08871500
H	4.99041400	0.49549900	2.41270200
C	3.37549800	1.64683500	1.58241000
H	3.96616600	2.55343900	1.50324300
C	2.05154200	1.63725400	1.14423000
H	1.60911500	2.54345700	0.74667100
C	-1.17520800	1.10505000	2.42287700
C	-2.34771900	1.86999500	2.48712500
H	-2.84669300	2.16256500	1.57614700
C	-2.88212400	2.25366900	3.71502900
H	-3.79574700	2.83958500	3.74108300
C	-2.26749700	1.85478100	4.90119200
H	-2.68966300	2.14324500	5.85936300
C	-1.10999600	1.07673200	4.84973100
H	-0.62204800	0.76206400	5.76773100
C	-0.56234400	0.70881500	3.62090000
H	0.34801400	0.12033200	3.59766400
P	-0.48805700	0.49728800	0.82603800
Fe	-2.44774900	2.35738200	-1.31860400
C	-3.91179600	0.93981200	-0.90737900
C	-4.34685600	2.24041600	-0.48261600
H	-4.62375300	2.51799800	0.52348400
C	-4.36086500	3.10119300	-1.61903000
C	-3.93807300	2.34542300	-2.75235800
C	-3.65005000	1.02000900	-2.31648200
H	-3.29237700	0.20127700	-2.92701700
C	-4.43277100	-0.35469800	1.65828600
C	-5.65885000	0.31164400	1.80098400
H	-6.16829400	0.70435100	0.92705100
C	-6.23815000	0.45676100	3.06030300
H	-7.18385000	0.98188500	3.16139700
C	-5.61425600	-0.08763300	4.18594600
H	-6.06882600	0.02388600	5.16623600
C	-4.41423900	-0.78382800	4.04567700
H	-3.92406000	-1.21118500	4.91501400
C	-3.82511000	-0.91323100	2.78893400
H	-2.87791900	-1.43011600	2.68532400
C	-4.90288700	-1.73043700	-0.75943600
C	-4.77675200	-3.10749800	-0.53216400
H	-3.92044000	-3.48785400	0.01507500
C	-5.73158900	-3.98998700	-1.03318100
H	-5.62080100	-5.05641000	-0.86030400
C	-6.81284600	-3.50687000	-1.77284400
H	-7.55090400	-4.19701000	-2.17160700
C	-6.93976400	-2.13682400	-2.00589500
H	-7.77765600	-1.75644800	-2.58349200
C	-5.99019400	-1.24913200	-1.49883800

H	-6.09543200	-0.18515600	-1.68471800
P	-3.66566200	-0.61647700	0.01123800
H	-1.39082000	5.00515700	-1.31986900
H	-3.80746500	2.72341600	-3.75756100
H	-4.61323500	4.15325500	-1.61174700
S	2.60950200	2.02001300	-2.43225700
N	1.01888600	-0.10890100	-2.18725400
H	0.86985700	-1.06740900	-1.89479700
N	3.18296200	-0.55167700	-1.72350500
H	2.80307200	-1.49230000	-1.65928500
F	5.94874000	-2.86519700	2.18415500
F	6.14731600	-3.97463100	0.32565900
F	7.85904400	-2.90383500	1.14807000
F	6.59143600	2.64213100	1.01974000
F	6.96609400	2.86827100	-1.11033900
F	8.42113600	1.85023500	0.15224300
C	-0.20458000	0.56894700	-2.62029800
H	-0.97602100	-0.17806700	-2.41682300
C	-0.22670200	0.82470700	-4.13014500
H	0.54781200	1.53583200	-4.42407000
H	-1.20586000	1.21320800	-4.42954600
H	-0.05780600	-0.11981600	-4.65441100
C	2.26897700	0.40985900	-2.10036600
C	4.44102800	-0.44990600	-1.11745000
C	4.92254200	-1.62114400	-0.51066700
H	4.33698900	-2.53303400	-0.55879200
C	6.11975000	-1.61083400	0.19437200
C	6.87054900	-0.44190000	0.31312400
H	7.79625200	-0.43017800	0.87450900
C	6.39534900	0.70974400	-0.30942100
C	5.20598200	0.71997500	-1.03596000
H	4.85367100	1.62660000	-1.50371100
C	6.52633400	-2.84234700	0.9518190
C	7.10143600	2.01554900	-0.07733100
Rh	-1.37308300	-1.35829200	-0.05737200
Cl	1.79799200	-3.77850000	-1.28679900
H	-1.33840500	-1.80032000	1.40611100
Cl	-1.75760800	-2.75804400	-1.96433100
H	0.70283100	-3.19496700	-0.83472700
H	0.10964900	-1.93267700	-0.05957100

TS4OR

C	-0.56391300	-4.32453400	1.84543400
C	-0.21401100	-3.32825900	2.79381600
H	-0.10485200	-3.47727900	3.85762900
C	-0.04277800	-2.08507100	2.12771000
C	-0.32899600	-2.30685700	0.72853500
C	-0.61809800	-3.71149900	0.56902400
H	-0.84488100	-4.21164300	-0.35784200
C	1.53179600	-0.93770100	-0.95398800
C	2.12220900	0.27200200	-1.32405700
H	1.56294500	1.18582600	-1.20417500
C	3.43445200	0.30693300	-1.79827000
H	3.89135200	1.25416700	-2.06630100
C	4.17629000	-0.87127900	-1.87313400
H	5.20563700	-0.84883000	-2.21433000
C	3.61682900	-2.07470100	-1.43873600
H	4.21953400	-2.97725000	-1.43178500
C	2.30096700	-2.11333000	-0.98438100
H	1.87092900	-3.05360100	-0.65782700
C	-0.76122200	-2.10525200	-2.10800400
C	-1.90102800	-2.91852400	-2.10021900
H	-2.49596400	-3.00944000	-1.20192200
C	-2.28871200	-3.61957700	-3.24193800
H	-3.17695000	-4.24355800	-3.20819400
C	-1.55867000	-3.49464000	-4.42270100
H	-1.86132400	-4.03655700	-5.31418100
C	-0.43418900	-2.66679800	-4.44967000
H	0.14453800	-2.56196900	-5.36314600
C	-0.03392800	-1.98277500	-3.30211300
H	0.85164100	-1.35818700	-3.33545300
P	-0.26794500	-1.09665600	-0.63326300
Fe	-1.97484800	-2.78829500	1.82262100
C	-3.55866000	-1.58556500	1.22422600
C	-3.91798400	-2.97657100	1.13202300
H	-4.21905800	-3.49359500	0.23317000
C	-3.80155000	-3.55778500	2.42759300
C	-3.36129000	-2.54407100	3.32880200
C	-3.19243200	-1.33588300	2.59276500
H	-2.79717400	-0.40579600	2.97201300
C	-4.54930300	-1.13759100	-1.45959300
C	-5.73372100	-1.84651500	-1.19602500
H	-6.07523500	-1.96524800	-0.17312600
C	-6.48504700	-2.37960400	-2.24068800
H	-7.39478800	-2.93183600	-2.02308700
C	-6.07737400	-2.19170900	-3.56523900
H	-6.66647800	-2.60501000	-4.37899300
C	-4.91739900	-1.46830400	-3.83657400

H	-4.58859700	-1.32337800	-4.86133900
C	-4.15696900	-0.94648200	-2.78824600
H	-3.23793600	-0.40938800	-2.99504900
C	-4.92072500	0.85580300	0.47674500
C	-5.89691400	1.31290000	-0.42562100
H	-5.91146900	0.94416000	-1.44553600
C	-6.88430700	2.21103300	-0.01427100
H	-7.63747300	2.54131700	-0.72420500
C	-6.91243600	2.66641600	1.30482300
H	-7.68745200	3.35459800	1.62975300
C	-5.93068300	2.23740700	2.19956500
H	-5.92838400	2.60027400	3.22309300
C	-4.93582100	1.35125300	1.78831800
H	-4.16315500	1.06498100	2.48550800
P	-3.61381300	-0.32553500	-0.09716900
H	-0.78092000	-5.36287500	2.05842000
H	-3.13276000	-2.67933700	4.37746300
H	-3.97494000	-4.59752800	2.67100200
S	3.12872200	-2.09993300	2.58224500
N	1.42342000	-0.08848100	2.17355800
H	1.22578800	0.86931300	1.88334200
N	3.56777300	0.44086200	1.68443100
H	3.13177500	1.35513100	1.51556500
F	6.66776500	3.87929100	-0.28741100
F	8.16263200	2.73970800	-1.38838800
F	6.11627400	2.85294000	-2.12053000
F	6.97948800	-2.73987100	-1.00775300
F	7.40593400	-2.94379900	1.11442300
F	8.82622800	-1.93236000	-0.19163400
C	0.29581000	-0.77682600	2.80247100
H	-0.52843100	-0.09862200	2.60089200
C	0.43006800	-0.88013300	4.32507200
H	1.25033900	-1.53764900	4.61909800
H	-0.50600700	-1.25388600	4.75609500
H	0.61564100	0.11801600	4.73243800
C	2.70717300	-0.53059300	2.13098400
C	4.84258900	0.35447100	1.11823700
C	5.31508300	1.52841600	0.50620900
H	4.71335500	2.43110000	0.55169500
C	6.50184100	1.51652200	-0.21723200
C	7.26270600	0.35411600	-0.33756200
H	8.17746500	0.34310200	-0.91606900
C	6.80615600	-0.79354200	0.30740300
C	5.62290800	-0.80747200	1.04317200
H	5.27897100	-1.71572500	1.51398300
C	6.87167400	2.74819900	-0.99079400
C	7.51118100	-2.09738800	0.07194800
Rh	-1.59130300	0.78432800	-0.65783100
Cl	2.26362000	3.32043000	0.87972200
H	-0.25798100	1.60912100	-1.09975700
H	-1.68318200	0.42088400	-2.17069400
Cl	-1.50226600	1.65937900	1.66849700
C	-0.29808300	3.32362700	-1.67206300
C	-1.43128700	4.37745700	0.13654000
C	-2.64891700	3.79128300	-0.24384000
H	-0.42322800	5.70920000	1.48055400
C	-1.38209900	5.29830500	1.18231400
C	-3.82539500	4.16224600	0.39213900
H	-3.51319200	2.57064500	-1.61930100
C	-3.77916700	5.09692100	1.42586300
C	-2.56211400	5.65444200	1.82825100
H	-4.76526200	3.71443500	0.09470300
H	-4.70133500	5.38008400	1.92327800
H	-2.53252500	6.36903000	2.64433700
N	-2.58543300	2.77682800	-1.25616700
N	-0.26195200	4.00921200	-0.54499900
C	0.94483500	3.22913000	-2.50040900
H	0.94702300	2.32395000	-3.10995000
H	0.96774400	4.10041700	-3.17098800
H	1.83246700	3.26103300	-1.86299600
H	0.65339200	3.98740500	-0.00493500
C	-1.65843200	3.21237300	-2.32355700
H	-1.64132600	2.47098000	-3.11882700
H	-1.94678700	4.19626900	-2.73140200

TS4OS

C	1.38395700	-4.46658100	0.34242700
C	1.07160900	-4.10652500	-0.99323500
H	1.03753900	-4.77676900	-1.83846900
C	0.79671100	-2.71418400	-1.05295900
C	0.98336100	-2.18630700	0.28066800
C	1.32368300	-3.29567200	1.13782500
H	1.50101800	-3.24622700	2.19961600
C	-1.02430900	-0.26857300	1.07005000
C	-1.59653900	1.00751000	1.07385100
H	-0.99702500	1.85490300	0.77302200
C	-2.91495600	1.17837600	1.49635700
H	-3.36157000	2.16474600	1.49674600

C	-3.66220100	0.07558600	1.91031700
H	-4.69334900	0.21186900	2.21796200
C	-3.10170200	-1.20234000	1.88604800
H	-3.70061700	-2.06268700	2.16459900
C	-1.78345700	-1.38000100	1.46936000
H	-1.35713000	-2.37514300	1.44135900
C	1.36976800	-0.42365100	2.56546400
C	2.49720300	-1.13608000	2.99463500
H	3.03938200	-1.75550900	2.29684500
C	2.93656900	-1.05257000	4.31454000
H	3.81549300	-1.61127300	4.62116100
C	2.27515100	-0.22596600	5.22078100
H	2.62177900	-0.15188300	6.24739800
C	1.16757500	0.51111400	4.79784000
H	0.64266200	1.15714000	5.49579200
C	0.71165200	0.40856000	3.48412900
H	-0.16650700	0.96673100	3.18011500
P	0.76742400	-0.46699900	0.82167000
Fe	2.73402100	-3.04127200	-0.35283900
C	4.23232000	-1.62018000	-0.40956000
C	4.60404500	-2.69995100	0.46402500
H	4.82936700	-2.61821300	1.51679700
C	4.61901100	-3.90269600	-0.29981200
C	4.26010800	-3.58126400	-1.64245100
C	4.01058600	-2.18195500	-1.71614900
H	3.69339900	-1.61997100	-2.58305600
C	4.68989700	0.48093600	1.59979100
C	5.87990200	-0.12844400	2.02386400
H	6.37965300	-0.84965500	1.38563900
C	6.43644700	0.20265700	3.25838200
H	7.35317400	-0.28166000	3.58258500
C	5.82776900	1.16535400	4.06729600
H	6.26381200	1.42219100	5.02838900
C	4.66472300	1.80118000	3.63299400
H	4.18391200	2.54827500	4.25712600
C	4.09805900	1.45963200	2.40664700
H	3.17428000	1.92784600	2.08570200
C	5.24224100	1.00492500	-1.08388800
C	5.21300300	2.40656200	-1.09412200
H	4.44423000	2.93470400	-0.53515800
C	6.15741700	3.12333400	-1.82283300
H	6.12446900	4.20906300	-1.83056000
C	7.13965000	2.44535400	-2.55036800
H	7.87292900	3.00345400	-3.12511200
C	7.17422400	1.05165600	-2.53779300
H	7.93569800	0.52007600	-3.10130600
C	6.22979300	0.32956700	-1.80485400
H	6.25848800	-0.75443200	-1.80595800
P	3.98832300	0.14127800	-0.06117900
H	1.65404400	-5.45398300	0.69219200
H	4.14115300	-4.28593500	-2.45436400
H	4.82453500	-4.89202900	0.08667100
S	-2.21582000	-3.56771600	-1.26009200
N	-0.96651200	-1.33417900	-2.08476400
H	-1.04635300	-0.38649100	-2.45655200
N	-3.19872100	-1.10168100	-1.85247500
H	-2.99278100	-0.21423400	-2.33745300
F	-6.38430800	2.60693000	-1.08897200
F	-6.28834200	2.22686800	1.05055500
F	-6.02564700	-3.29408900	2.14138900
F	-6.62933900	-4.30451100	0.31519300
F	-8.01888500	-2.97041700	1.33286800
C	0.34102600	-1.96678300	-2.28735400
H	1.01294300	-1.12172800	-2.43462300
C	0.39011400	-2.81255500	-3.56406700
H	-0.30788800	-3.65143700	-3.51198200
H	1.40505200	-3.19316300	-3.72510700
H	0.12100300	-2.18458000	-4.41790100
C	-2.13167500	-1.95585800	-1.75020300
C	-4.42197000	-1.07955500	-1.18493400
C	-5.04475100	0.18134800	-1.10952100
H	-4.58024700	1.02591700	-1.60618100
C	-6.20434300	0.34689300	-0.36646300
C	-6.79246400	-0.72925600	0.30435800
H	-7.68975400	-0.59364800	0.89499900
C	-6.19184400	-1.97849700	0.19105100
C	-5.02814000	-2.17313800	-0.55467700
H	-4.57128300	-3.14922900	-0.61990700
C	-6.72510800	-3.13800200	0.98271000
Rh	1.83200200	0.94783100	-0.56146300
Cl	-2.45525000	1.45548400	-3.49030700
H	.46746000	1.88449100	-0.99358500
H	1.69629700	1.79419500	0.72459900
Cl	2.51132500	0.77254400	-2.87235900
C	-0.02789800	3.37064700	-1.51826800
C	-2.09371500	3.92422900	-0.38225800
C	-1.39887300	4.41014800	0.74308300
H	-3.99084900	3.60524100	-1.32988400

C	-3.48447700	3.99870900	-0.45482500
C	-2.12817100	4.96295300	1.79614000
H	0.45895100	4.75809900	1.49562800
C	-3.52181500	5.03696600	1.72344900
C	-4.20201800	4.56113300	0.60057400
H	-1.60230700	5.32929800	2.67454800
H	-4.07468700	5.46945300	2.55170900
H	-5.28304400	4.59934500	0.55092900
N	-0.01009200	4.22581500	0.77284000
N	-1.35985200	3.36444300	-1.43565300
C	0.54415000	3.33239300	-2.90851000

12. Supporting References

1. Jie, Q.; Chen, F.; Ding, Z. Y.; He, Y. M.; Xu, L. J.; Fan, Q. H., Asymmetric hydrogenation of 2- and 2,3-substituted quinoxalines with chiral cationic ruthenium diamine catalysts, *Org. Lett.*, **2011**, *12* (24), 6568-6571.
2. Tang, H.; Wub, J.; Zhang, W.; Lei Zhao, L.; Zhang, Y., H.; Shen, C. W., Design, synthesis and biological evaluation of novel non-azole derivatives as potential antifungal agents, *Chinese. Chem. Lett.*, **2015**, *26* (9), 1161-1164.
3. Huang, L.; Xu, J.; He, L.; Liang, C.; Ouyang, Y.; Yu, Y.; Li, W.; Zhang, P., Rapid alkenylation of quinoxalin-2(1H)-ones enabled by the sequential Mannich-type reaction and solar photocatalysis, *Chinese Chem. Lett.*, **2021**, *32* (11), 3627-3631.
4. Ghosh, P.; Kwon, N. Y.; Kim, S.; Han, S.; Lee, S. H.; An, W.; Mishra, N. K.; Han, S. B.; Kim, I. S., C−H Methylation of Iminoamido Heterocycles with Sulfur Ylides**, *Angew. Chem. Int. Ed.*, **2020**, *60* (1), 191-196.
5. Zhao, Q. Y.; Li, S. K.; Huang, K. X.; Wang, R.; Zhang, X. M., A Novel Chiral Bisphosphine-Thiourea Ligand for Asymmetric Hydrogenation of β,β-Disubstituted Nitroalkenes, *Org. Lett.*, **2013**, *15* (15), 4014-4017.
6. Hayashi, T.; Mise, T.; Fukushima, M.; Kagotani, M.; Nagashima, N.; Hamada, Y.; Matsumoto, A.; Kawakami, S.; Konishi, M.; Yamamoto, K.; Kumada, M., Asymmetric synthesis catalyzed by chiral ferrocenylphosphine-Transition metal complexes . preparation of chiral ferrocenylphosphines, *Bull. chem. soc. jpn.*, **1980**, *53* (1138-1151).
7. Sun, S.; Nagorny, P., Exploration of chiral diastereomeric spiroketal (SPIROL)-based phosphinite ligands in asymmetric hydrogenation of heterocycles, *Chem. Commun*, **2020**, *56*, 8432-8435.
8. M. J. Frisch, G. W. T., H. B. Schlegel, G. E. Scuseria, M. A. Robb, J. R. Cheeseman, G. Scalmani, V. Barone, B. Mennucci, G. A. Petersson, H. Nakatsuji, M. Caricato, X. Li, H. P. Hratchian, A. F. Izmaylov, J. Bloino, G. Zheng, J. L. Sonnenberg, M. Hada, M. Ehara, K. Toyota, R. Fukuda, J. Hasegawa, M. Ishida, T. Nakajima, Y. Honda, O. Kitao, H. Nakai, T. Vreven, J. A. Montgomery, Jr., J. E. Peralta, F. Ogliaro, M. Bearpark, J. J. Heyd, E. Brothers, K. N. Kudin, V. N. Staroverov, R. Kobayashi, J. Normand, K. Raghavachari, A. Rendell, J. C. Burant, S. S. Iyengar, J. Tomasi, M. Cossi, N. Rega, J. M. Millam, M. Klene, J. E. Knox, J. B. Cross, V. Bakken, C. Adamo, J. Jaramillo, R. Gomperts, R. E. Stratmann, O. Yazyev, A. J. Austin, R. Cammi, C. Pomelli, J. W. Ochterski, R. L. Martin, K. Morokuma, V. G. Zakrzewski, G. A. Voth, P. Salvador, J. J. Dannenberg, S. Dapprich, A. D. Daniels, Ö. Farkas, J. B. Foresman, J. V. Ortiz, J. Cioslowski, and D. J. Fox, Gaussian 09 (Gaussian, Inc., Wallingford CT, 2009). *Gaussian 09*, 2009.
9. Petersson, G. A.; Bennett, A.; Tensfeldt, T. G.; Al-Laham, M. A.; Shirley, W. A.; Mantzaris, J., A complete basis set model chemistry. I. The total energies of closed-shell atoms and hydrides of the first-row elements, *J. Chem. Phys.*, **1988**, *89* (4), 2193-2218.
10. Becke, A. D., Density-functional thermochemistry. III. The role of exact exchange, *J. Chem. Phys.*, **1993**, *98* (7), 5648-5652.

11. Yang, T.; Sun, Y.; Wang, H.; Lin, Z.; Wen, J.; Zhang, X., Iridium-Catalyzed Enantioselective Hydrogenation of Oxocarbenium Ions: A Case of Ionic Hydrogenation, *Angew. Chem. Int. Ed.*, **2020**, *59* (15), 6108-6114.
12. Wen, J. L.; Fan, X. R.; Tan, R. C.; Chien, H. C.; Zhou, Q. H.; Chung, L. W.; Zhang, X. M., Brønsted-Acid-Promoted Rh-Catalyzed Asymmetric Hydrogenation of N-Unprotected Indoles: A Cocatalysis of Transition Metal and Anion Binding, *Org. Lett.*, **2018**, *20*, 2143−2147.
13. Marenich, A. V.; Cramer, C. J.; Truhlar, D. G., Universal solvation model based on solute electron density and on a continuum model of the solvent defined by the bulk dielectric constant and atomic surface tensions, *J. Phys. Chem B.*, **2009**, *113* (18), 6378-96.

13.NMR Spectra

^{1}H NMR spectra for L2

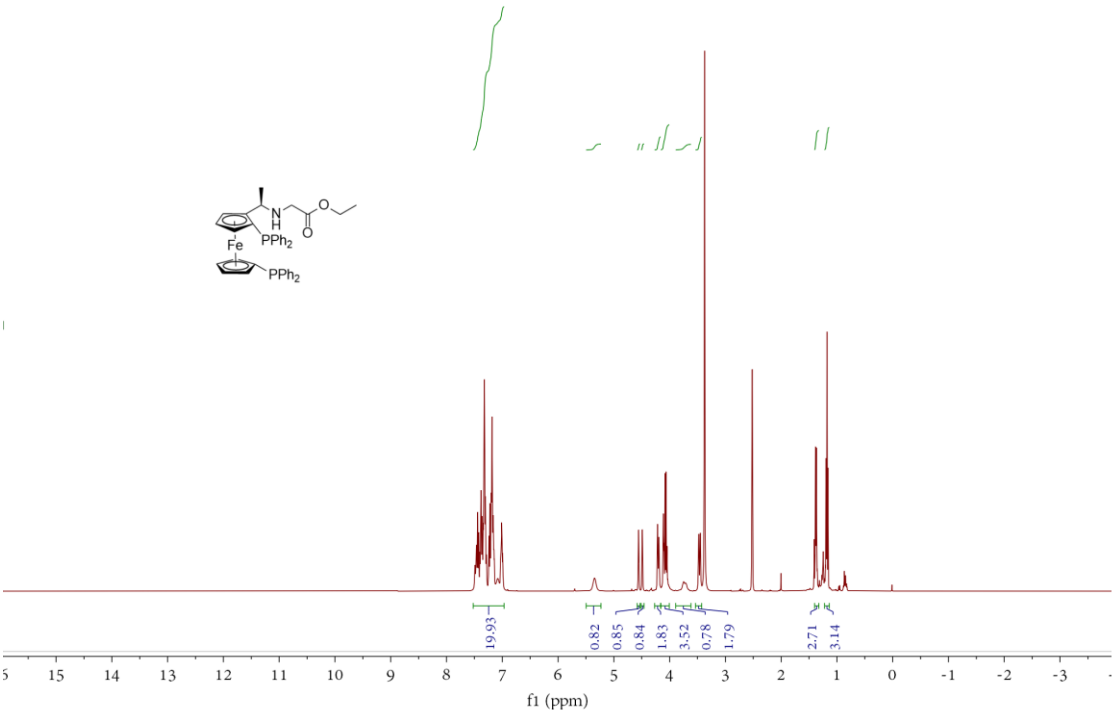

^{13}C NMR spectra for L2

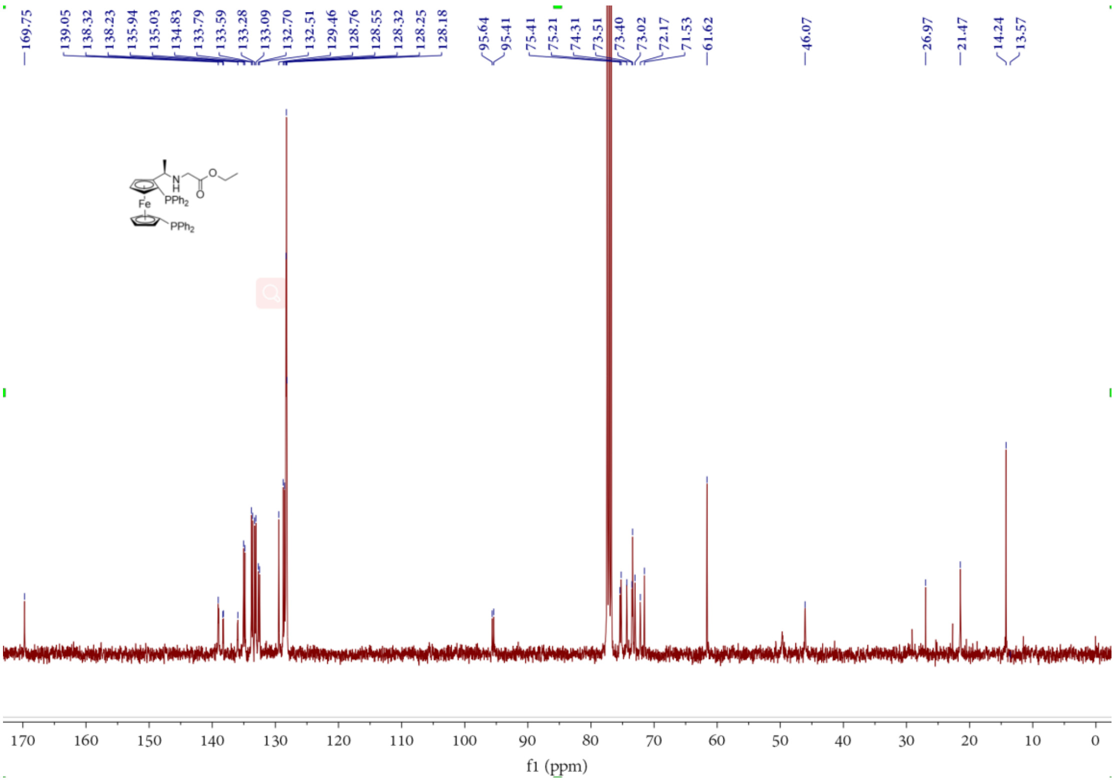

^{31}P NMR spectra for L2

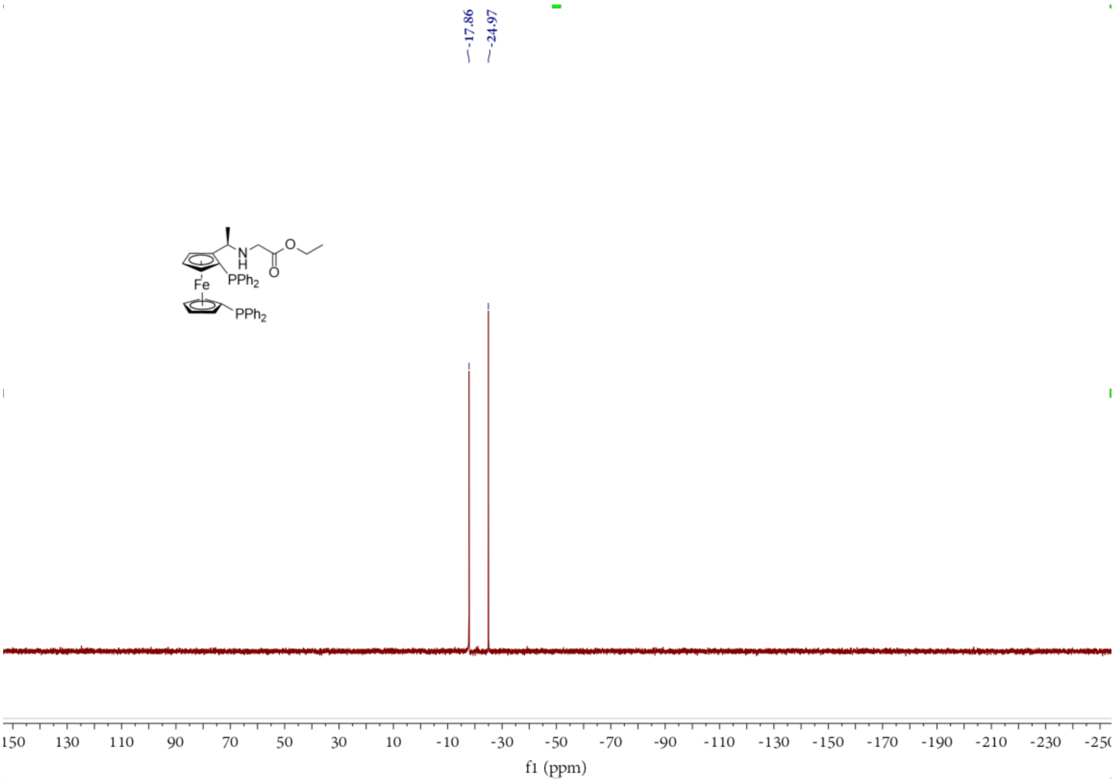

^{1}H NMR spectra for 2a

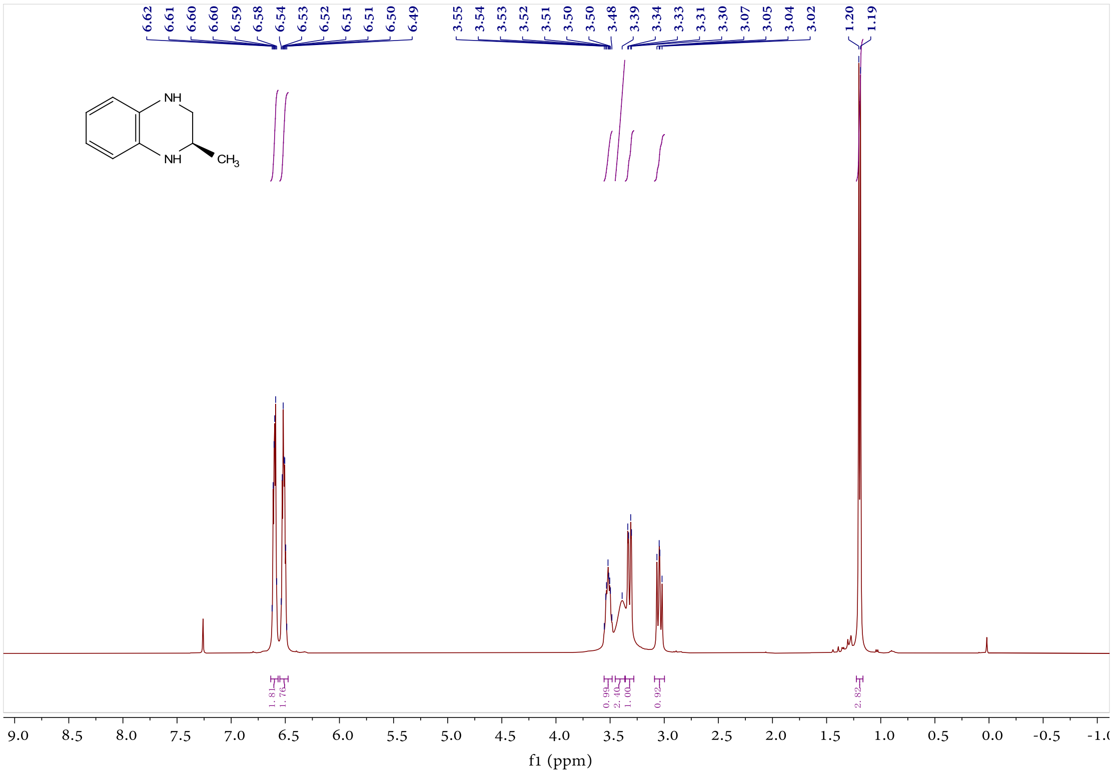

^{13}C NMR spectra for 2a

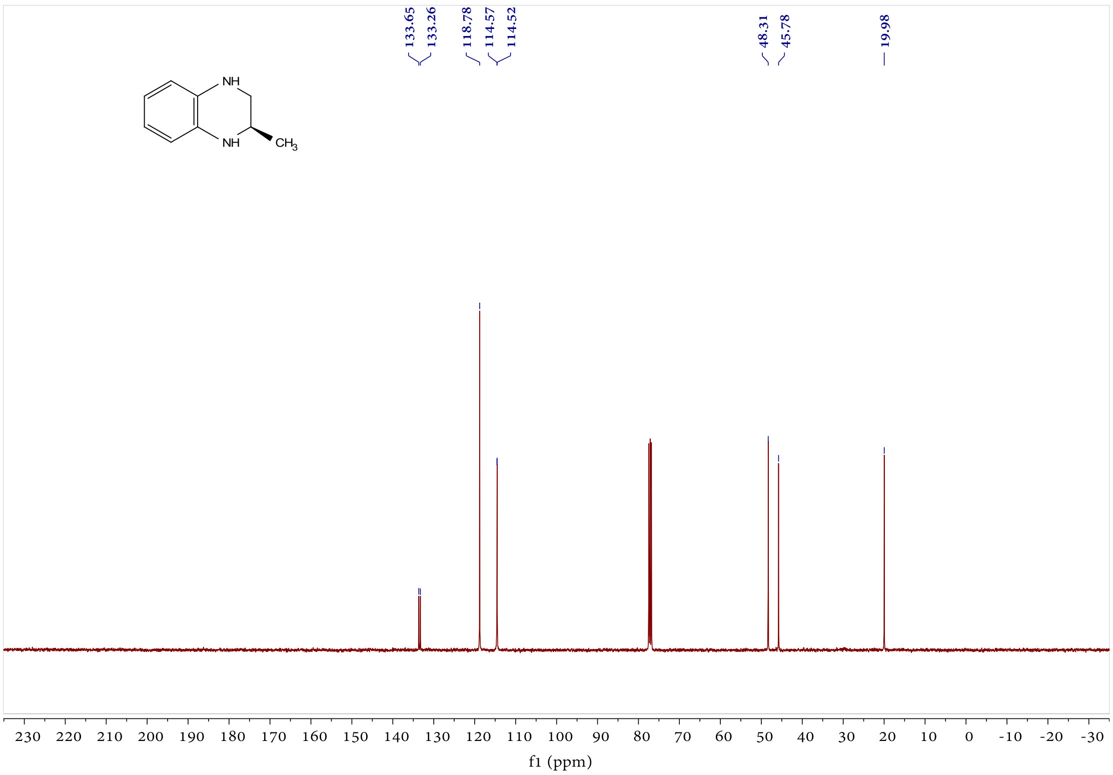

^{1}H NMR spectra for 2b

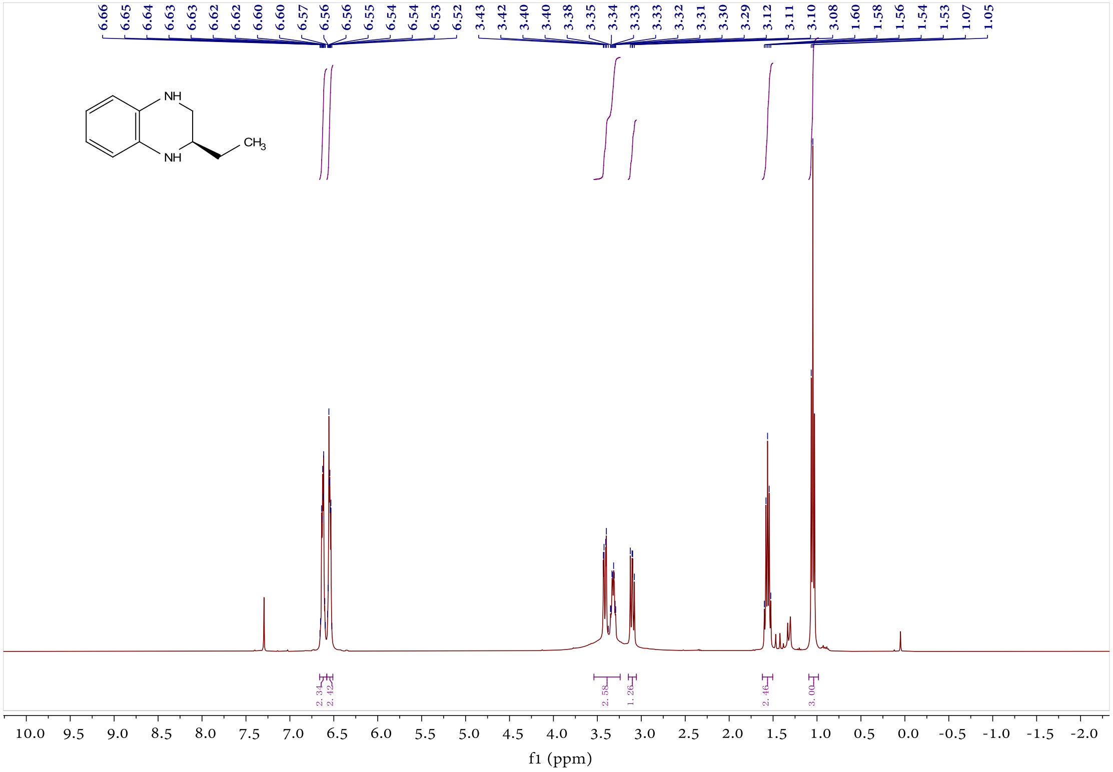

^{13}C NMR spectra for 2b

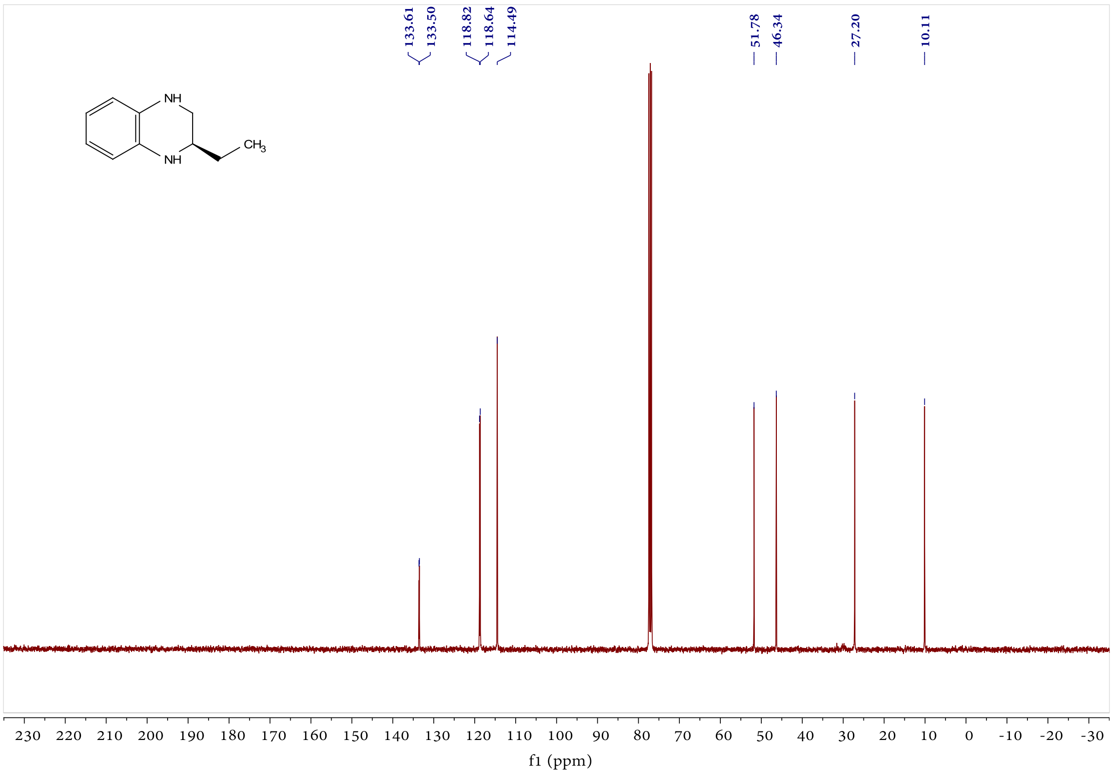

^{1}H NMR spectra for 2c

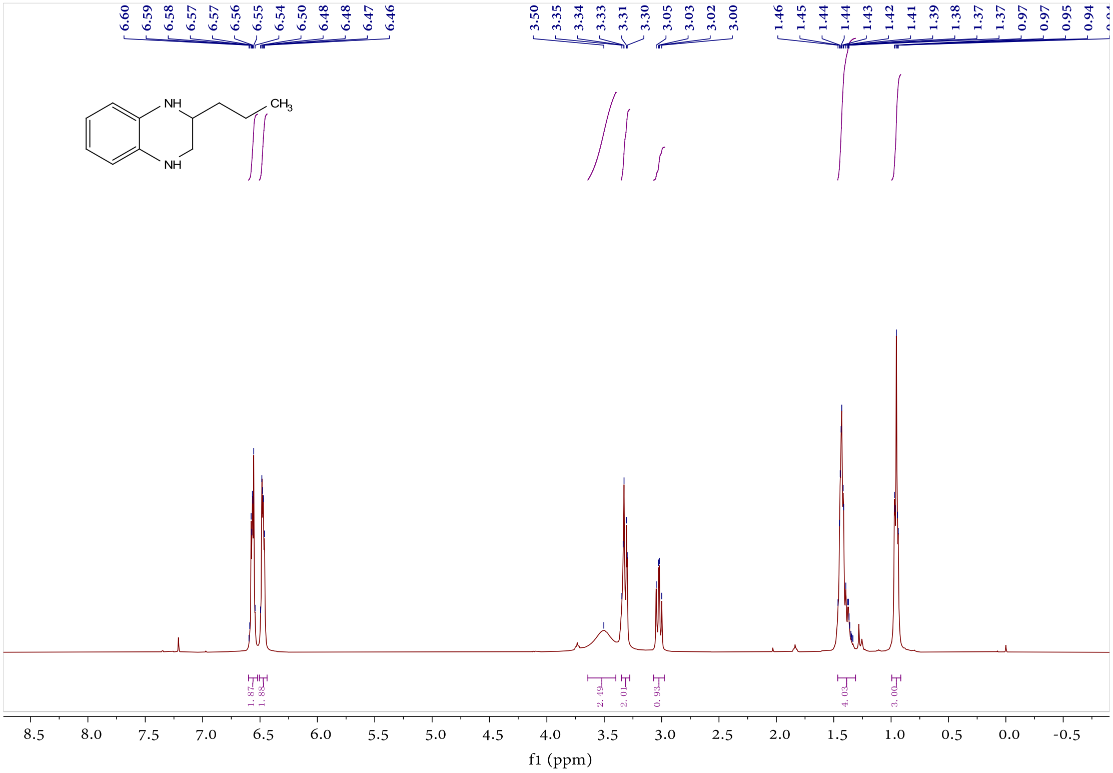

^{13}C NMR spectra for 2c

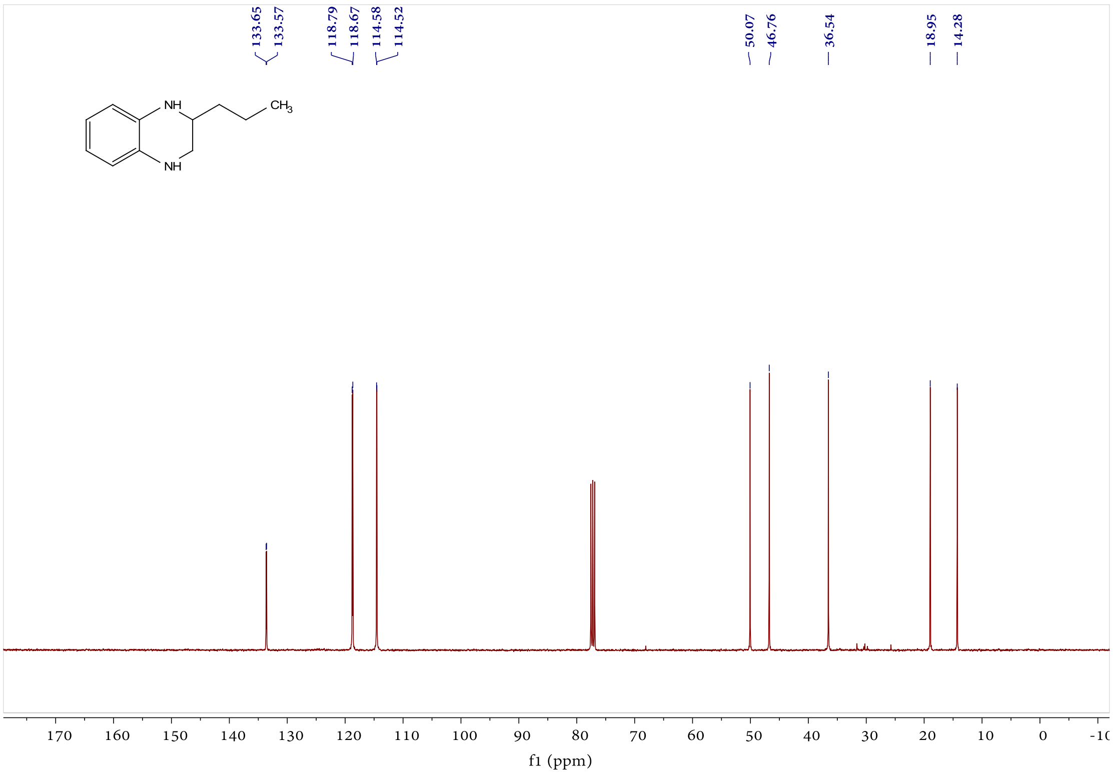

^{1}H NMR spectra for 2d

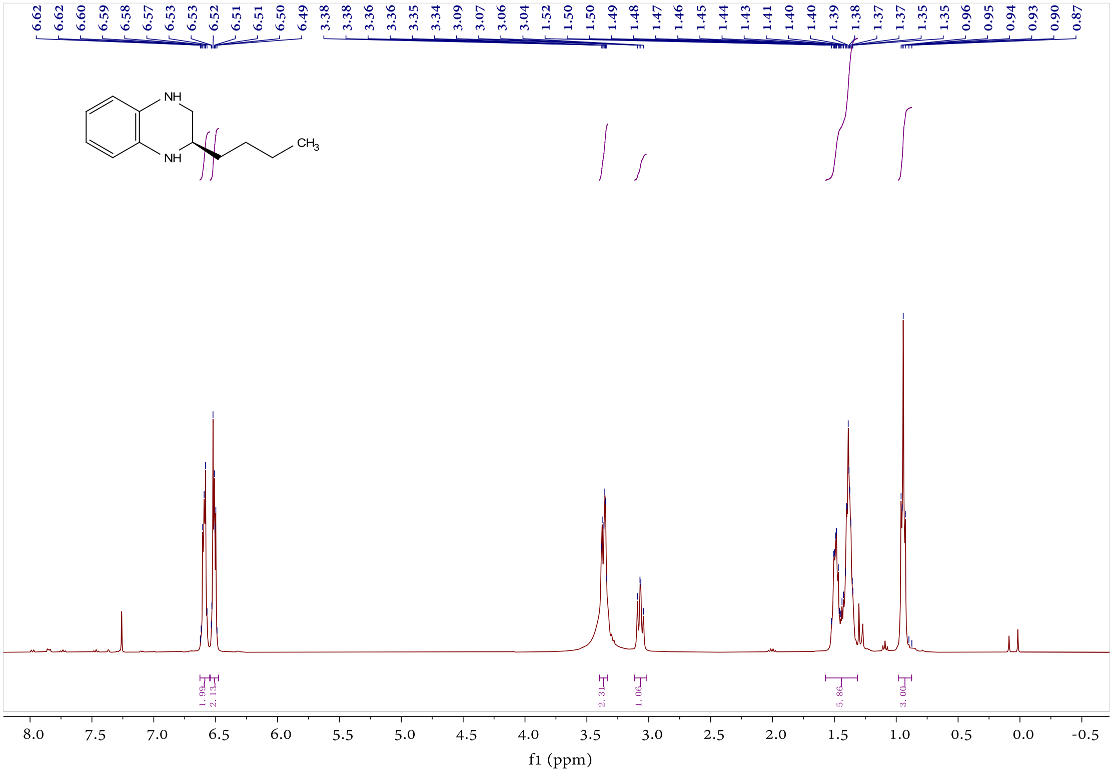

^{13}C NMR spectra for 2d

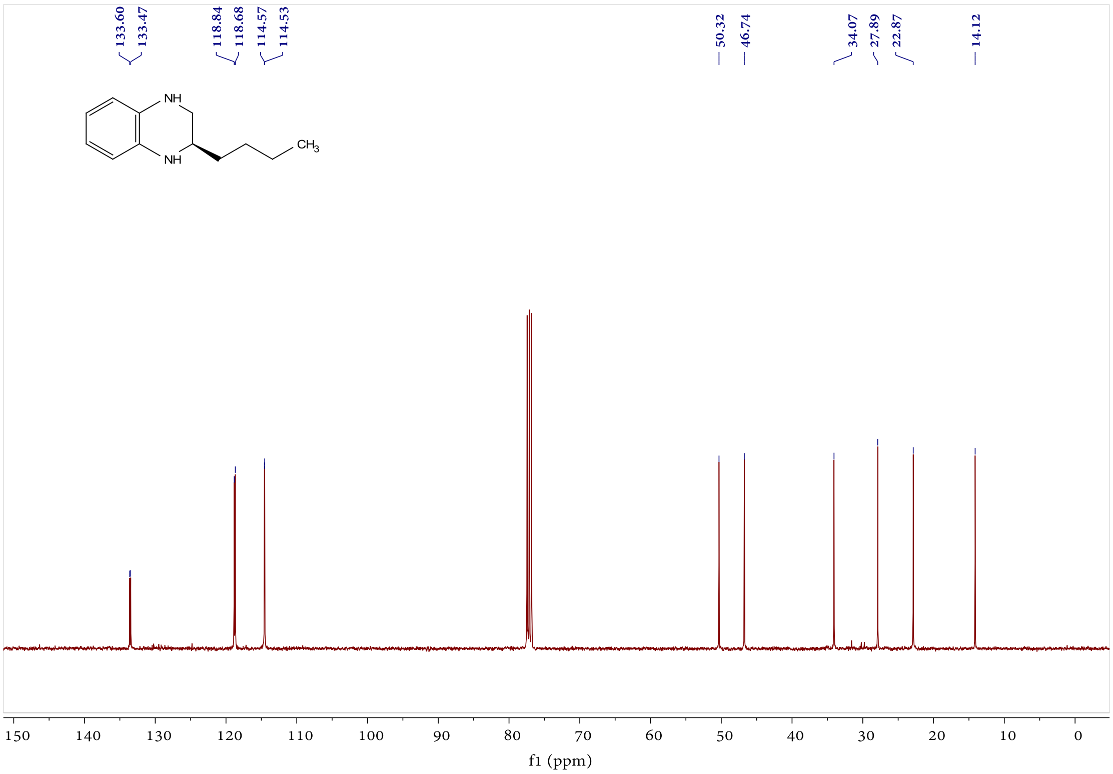

^{1}H NMR spectra for 2e

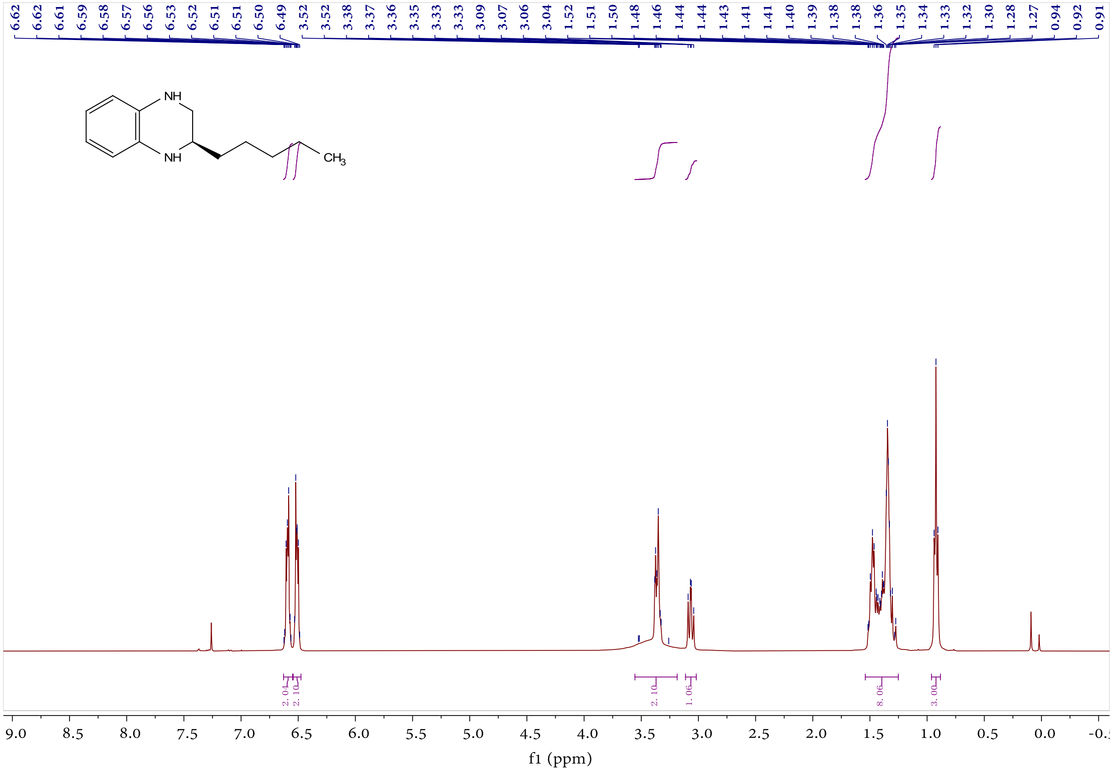

^{13}C NMR spectra for 2e

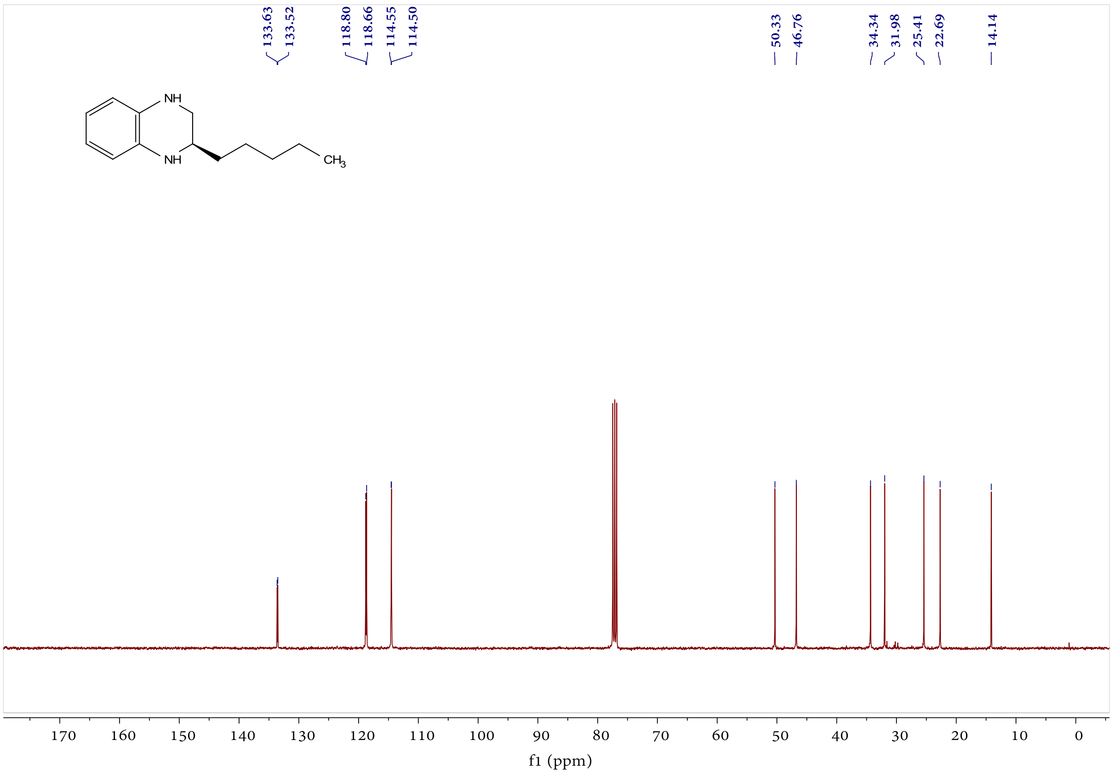

^{1}H NMR spectra for 2f

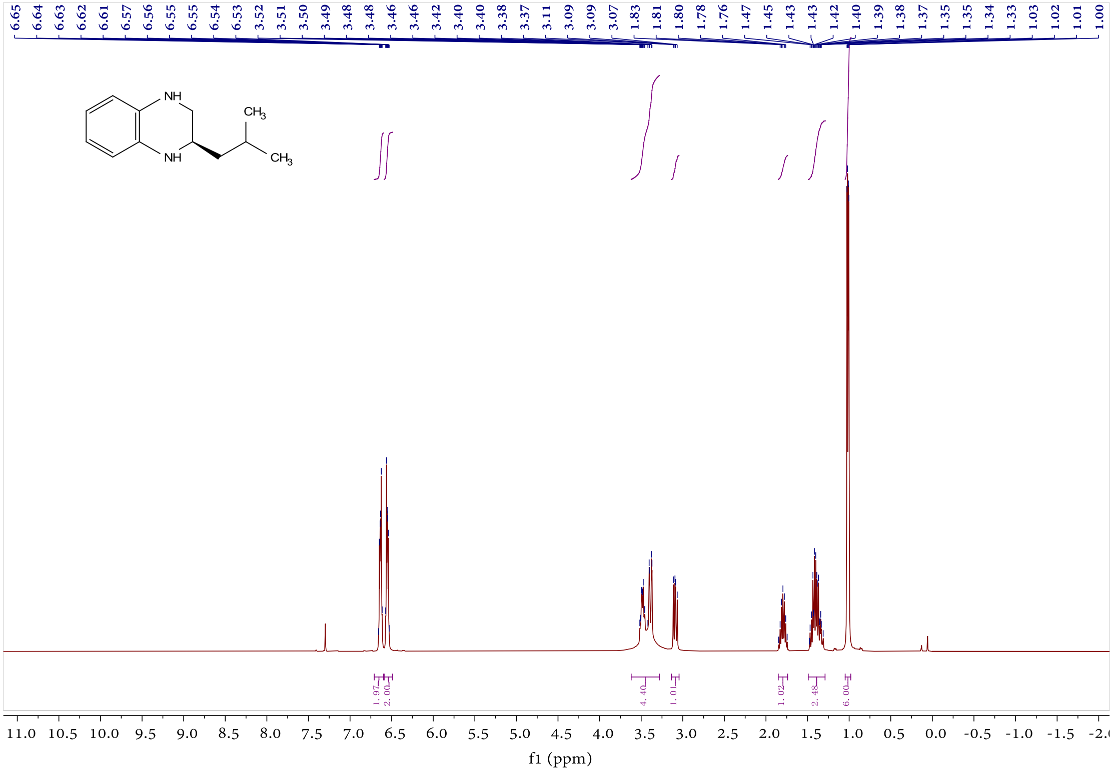

^{13}C NMR spectra for 2f

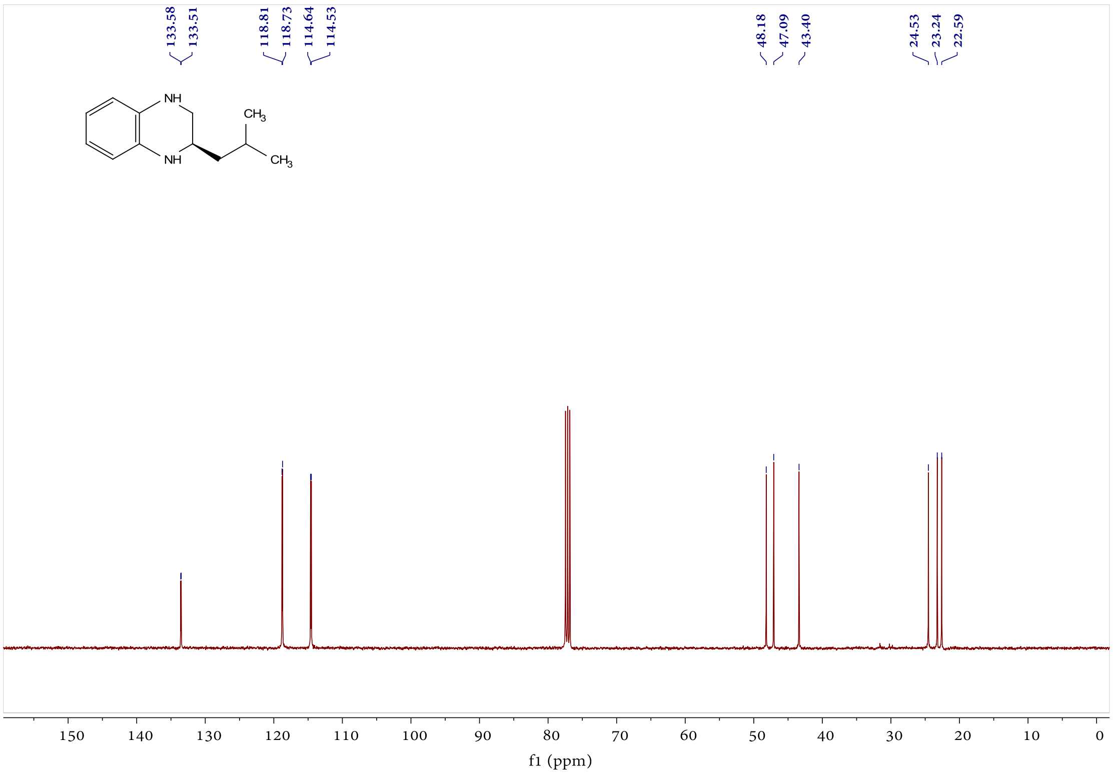

^{1}H NMR spectra for 2g

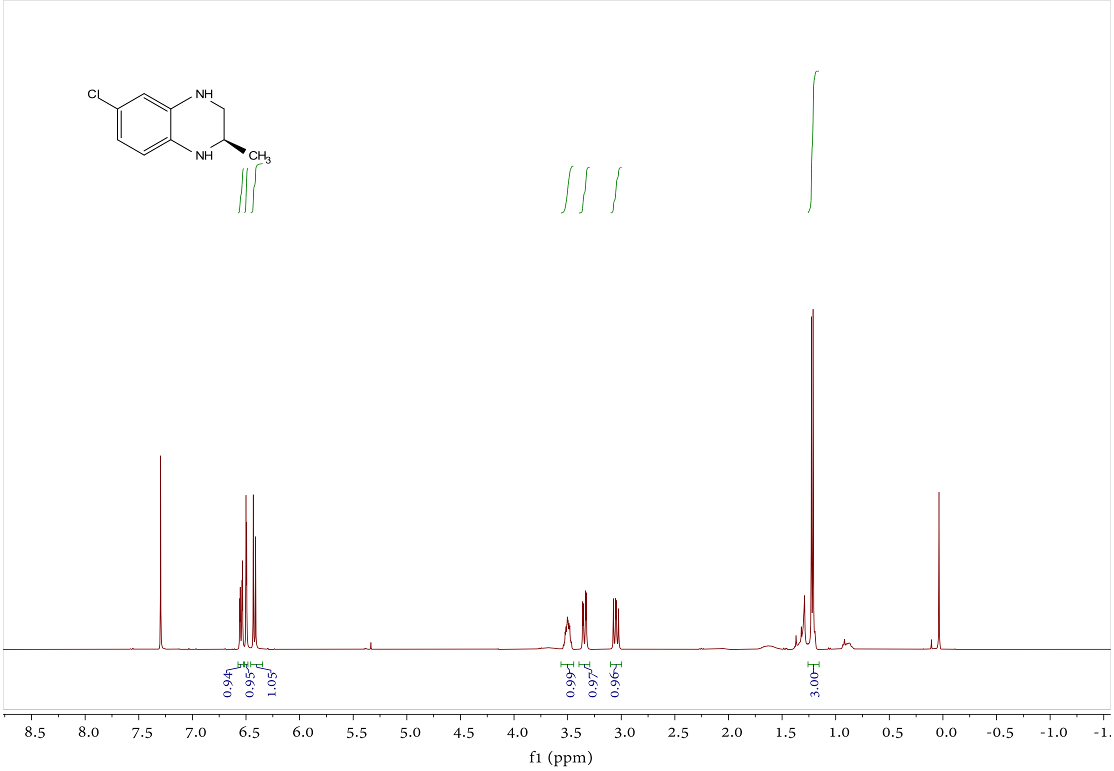

^{13}C NMR spectra for 2g

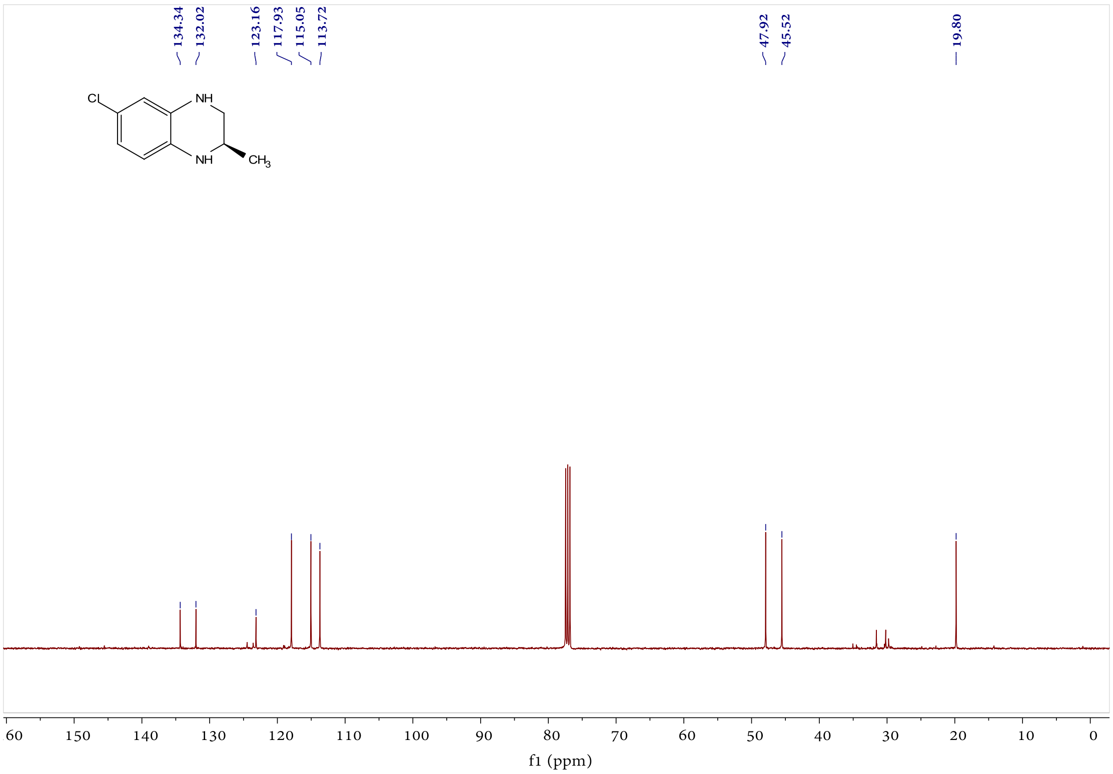

^{1}H NMR spectra for 2h

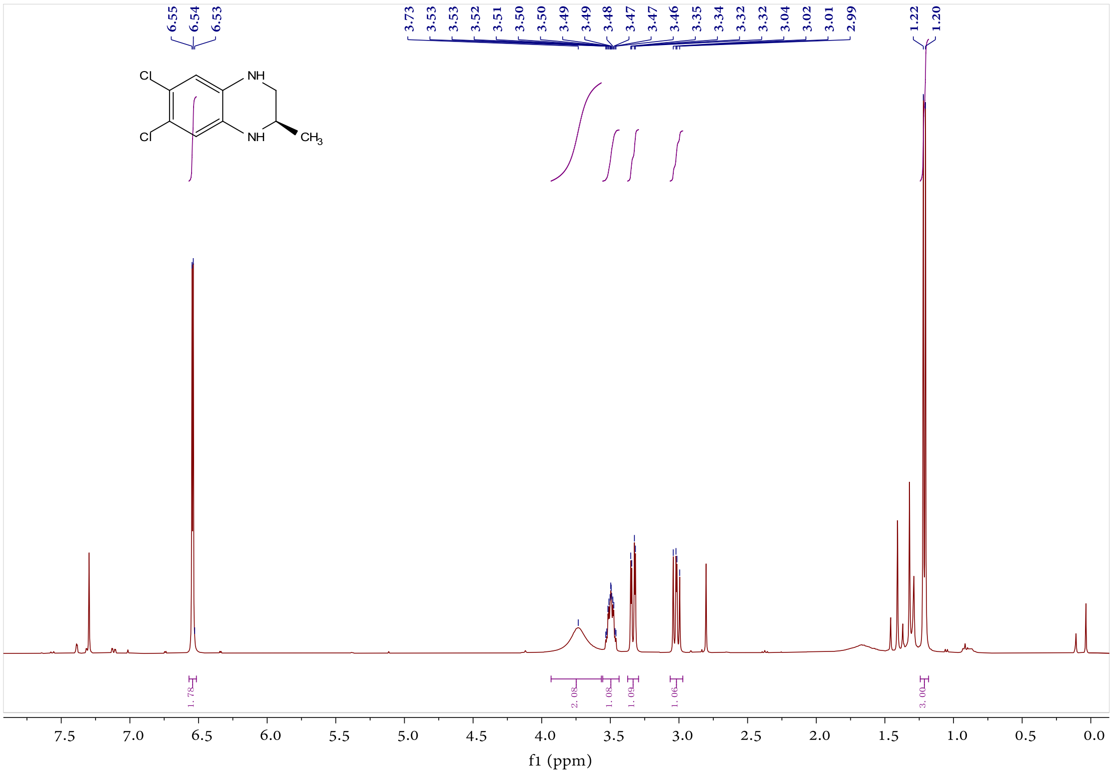

^{13}C NMR spectra for 2h

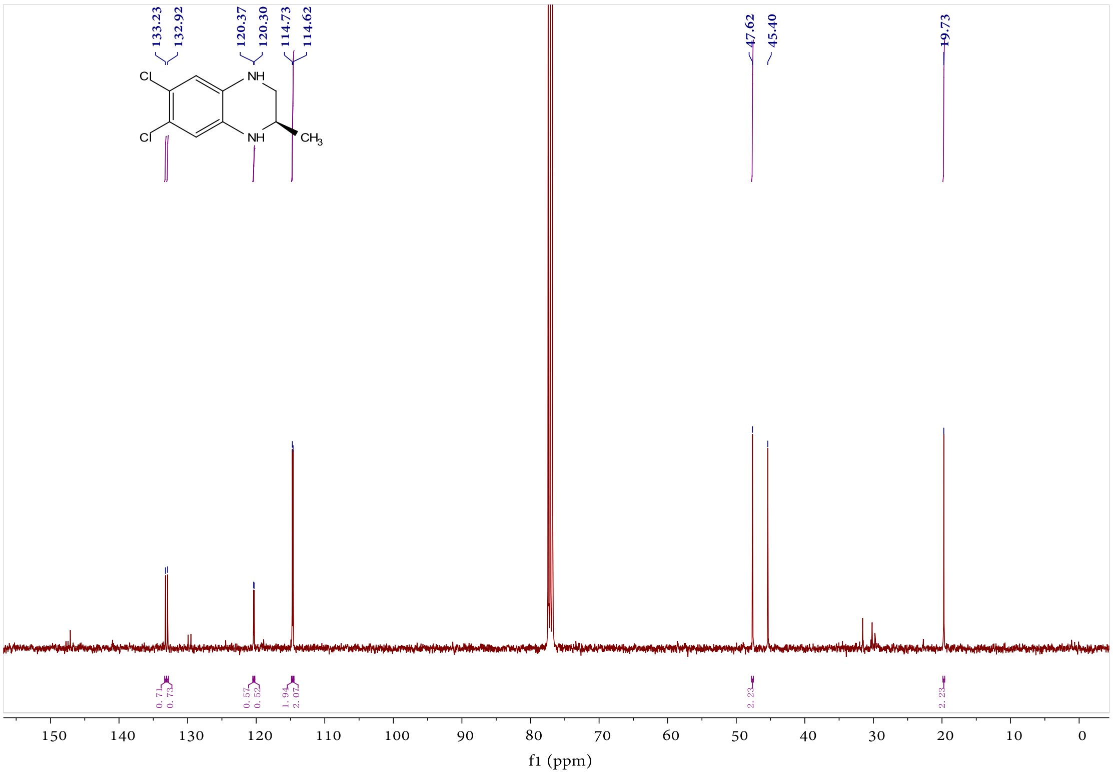

^{1}H NMR spectra for 2i

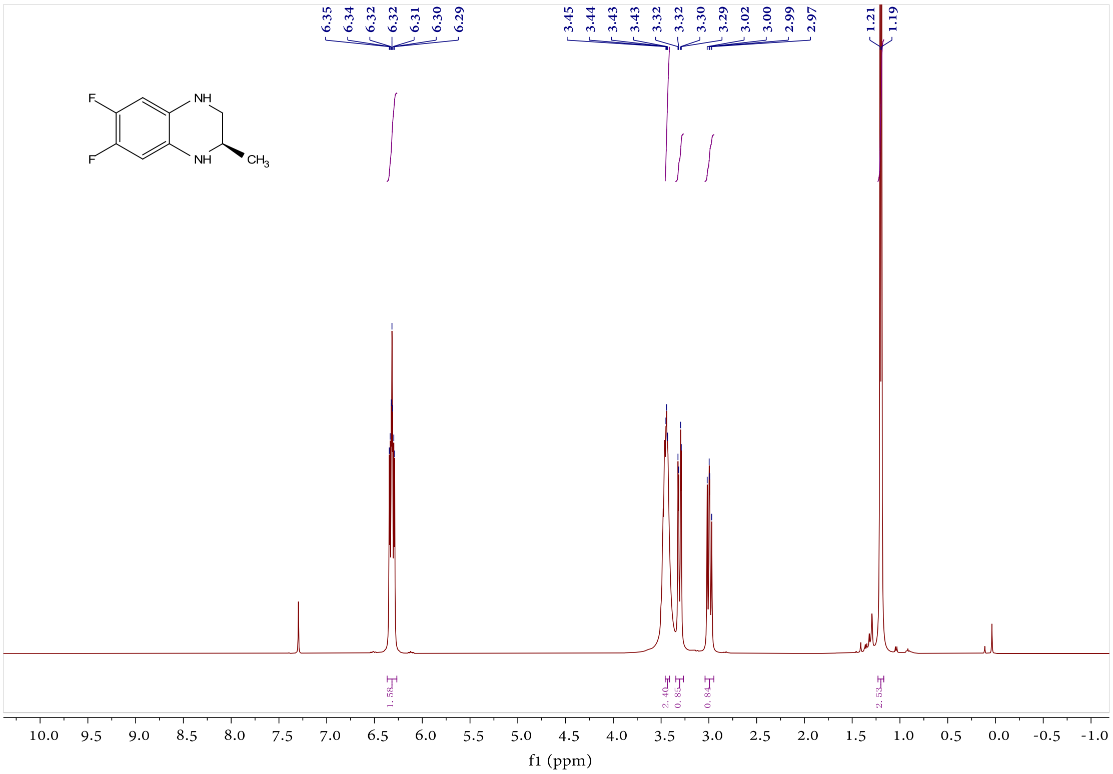

^{13}C NMR spectra for 2i

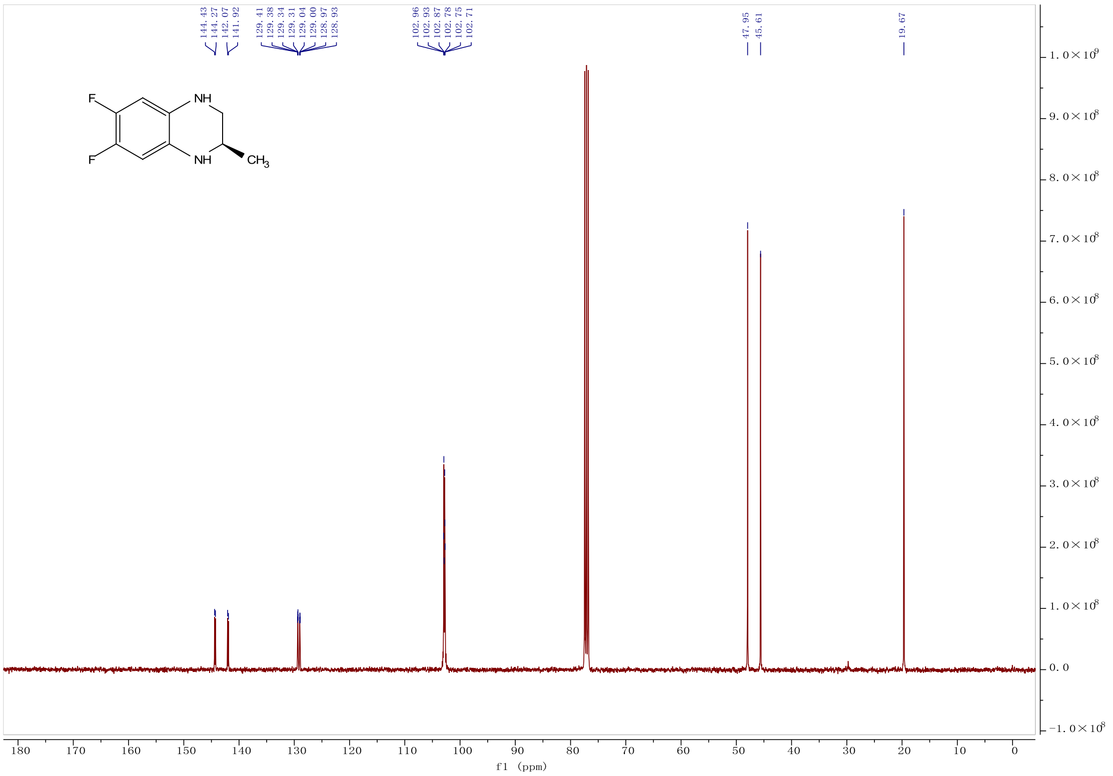

^{1}H NMR spectra for 2j

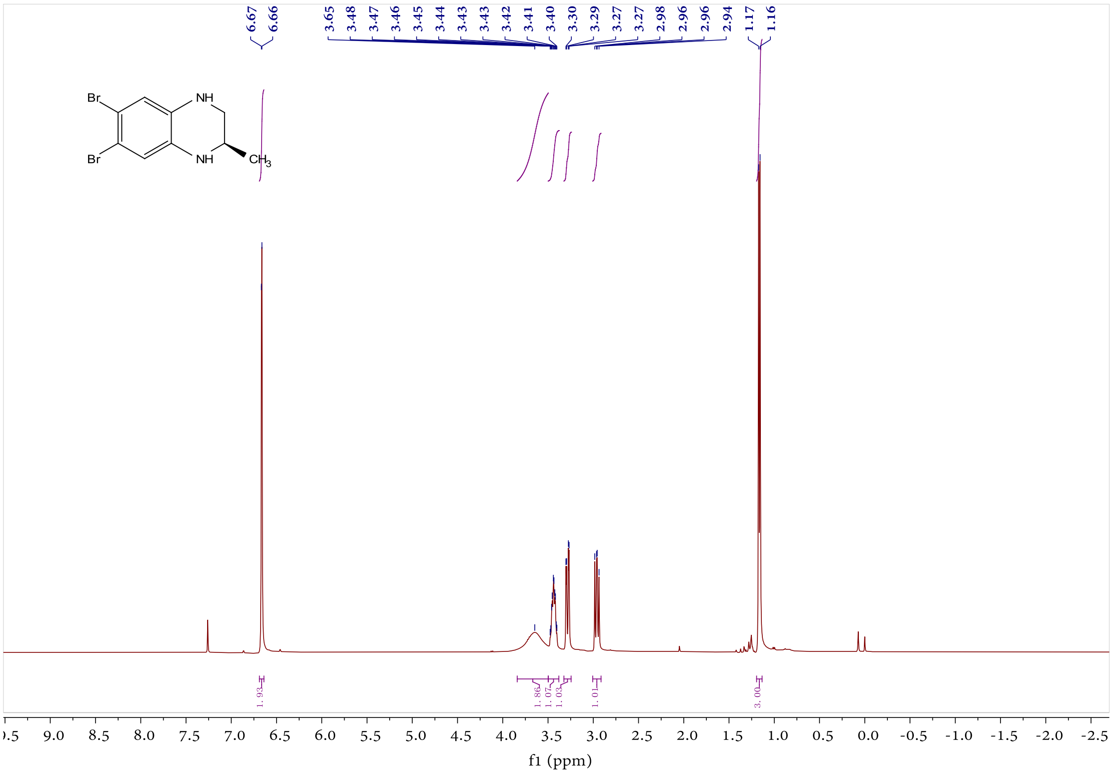

^{13}C NMR spectra for 2j

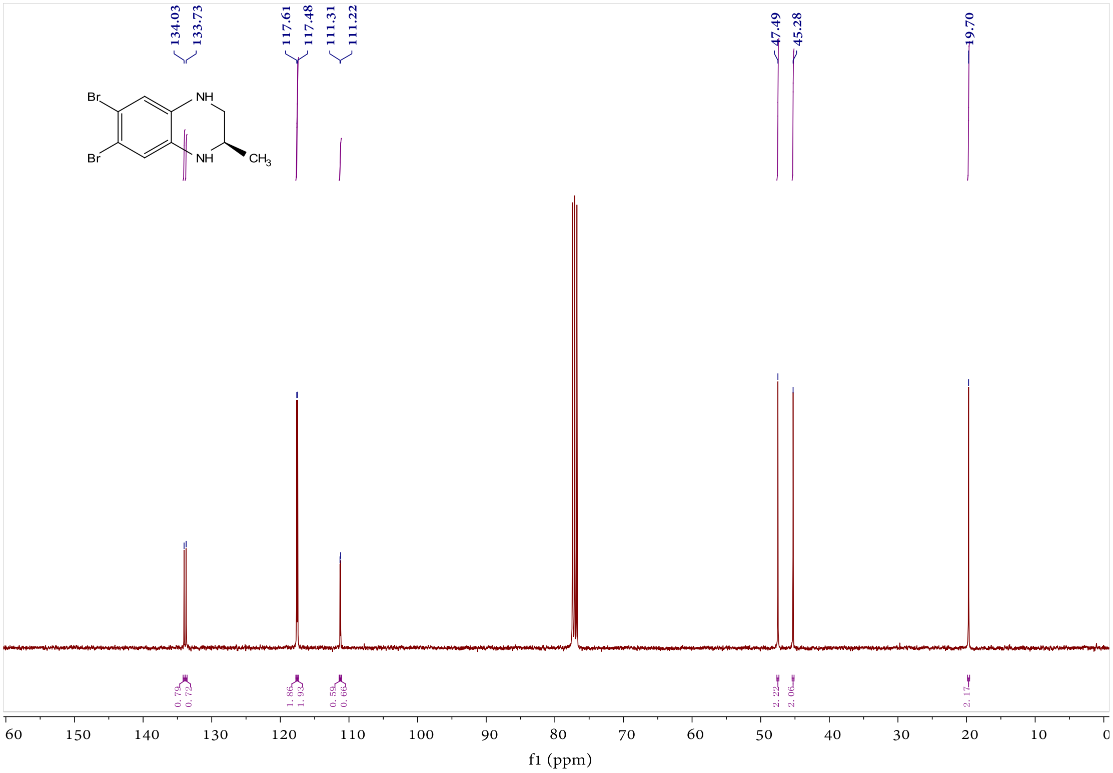

^{1}H NMR spectra for 2k

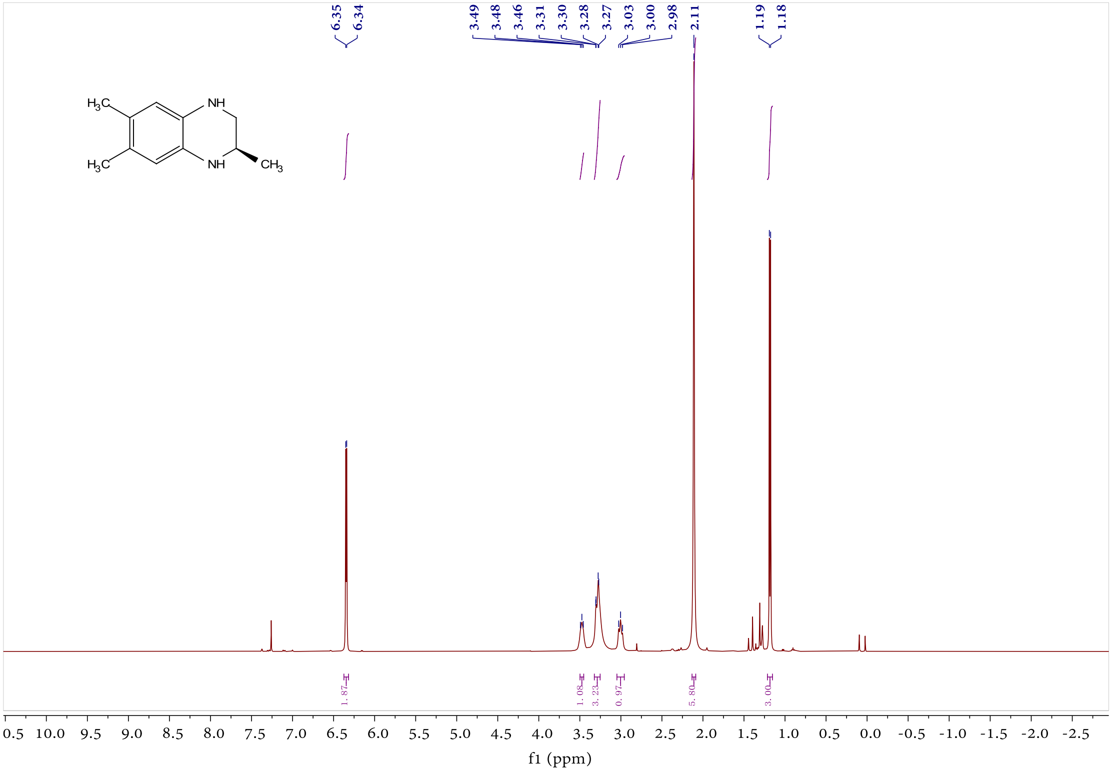

^{13}C NMR spectra for 2k

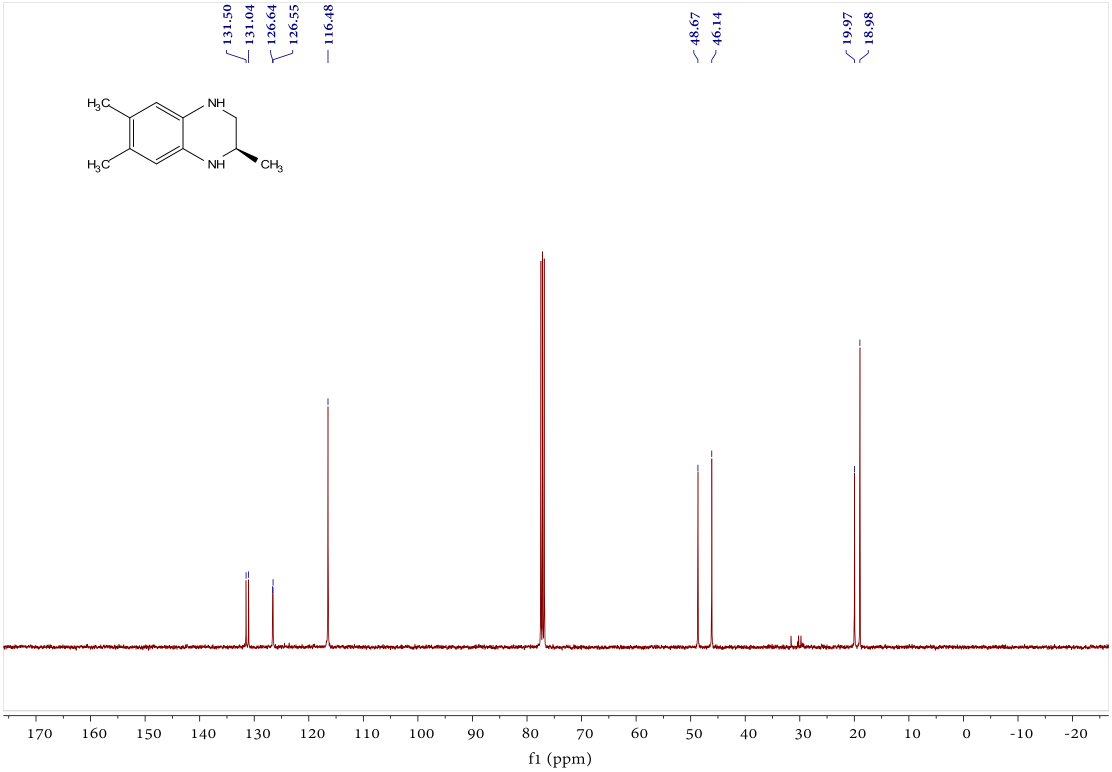

^{1}H NMR spectra for 2l

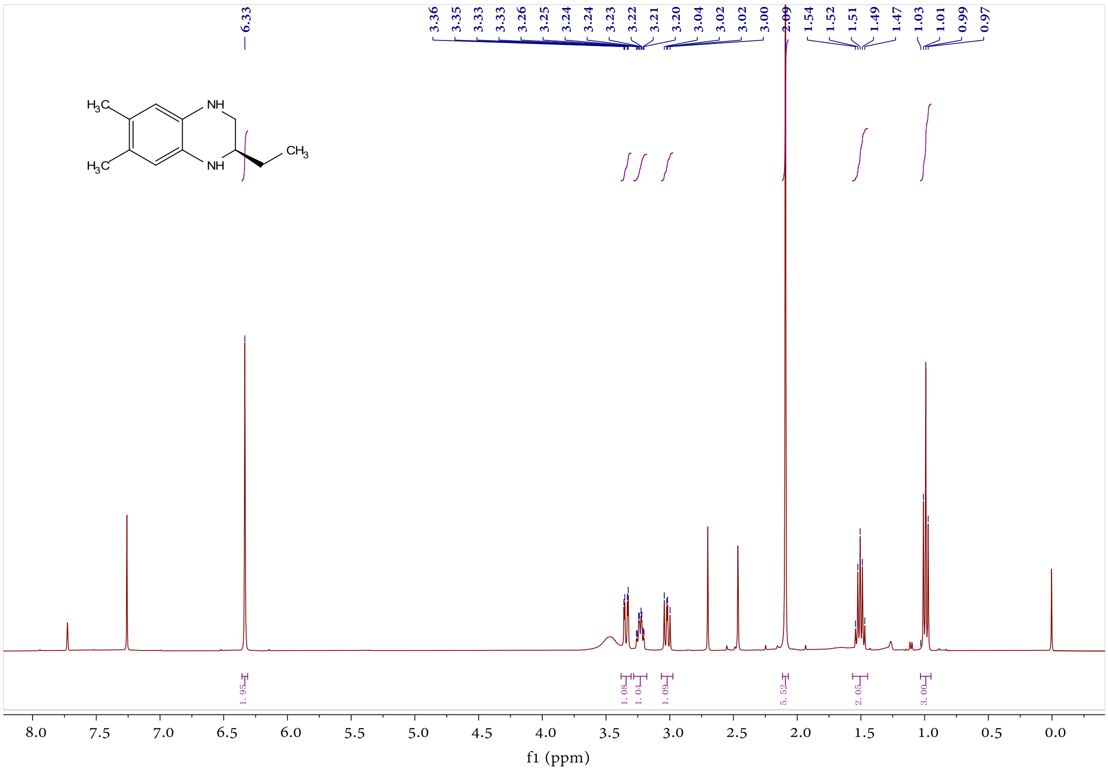

^{13}C NMR spectra for 2l

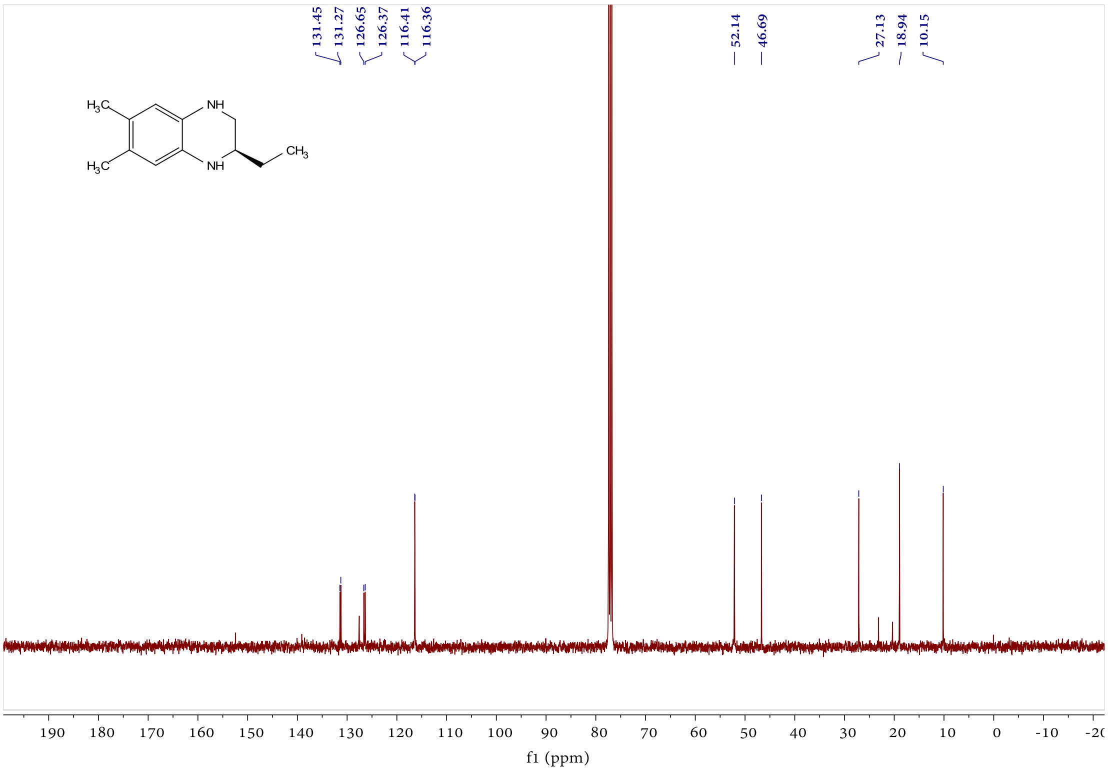

1H NMR spectra for 2m

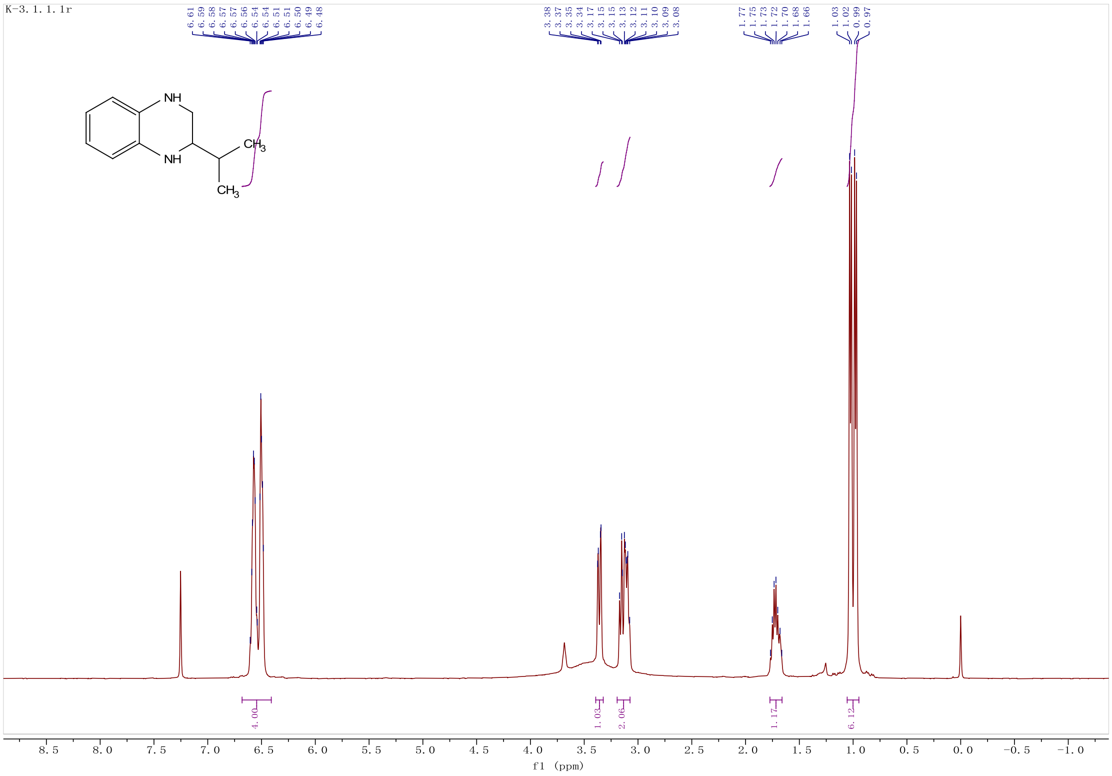

13C NMR spectra for 2m

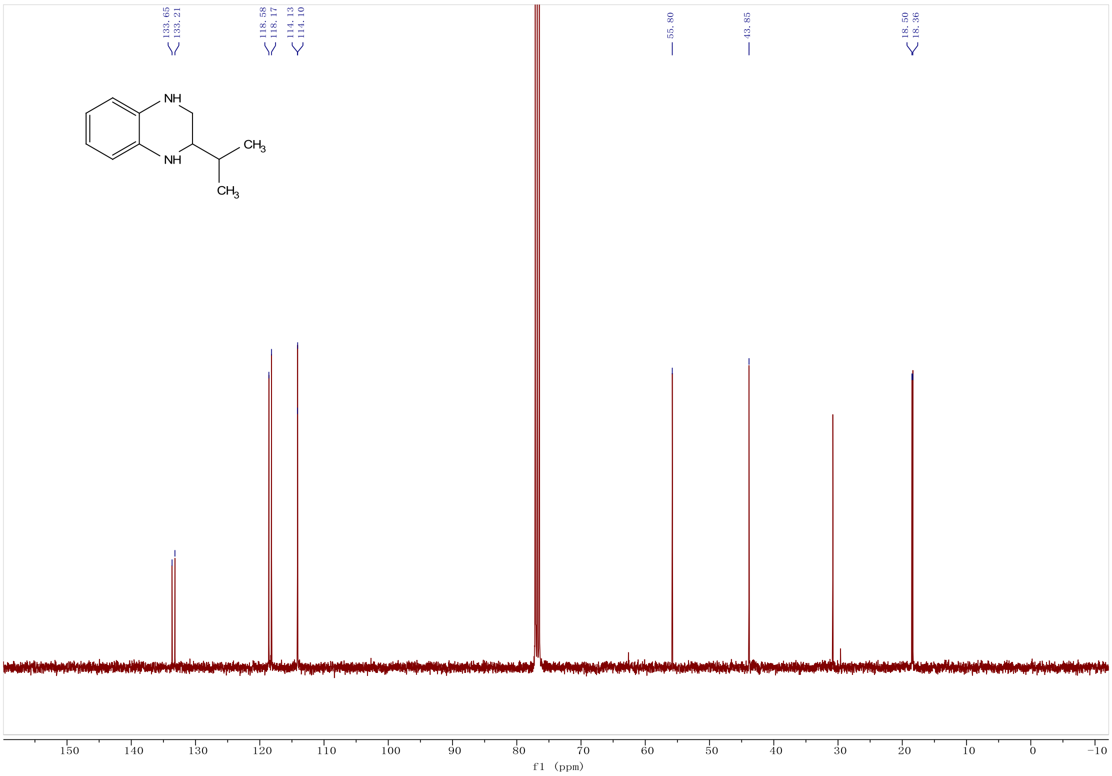

^{1}H NMR spectra for 2n

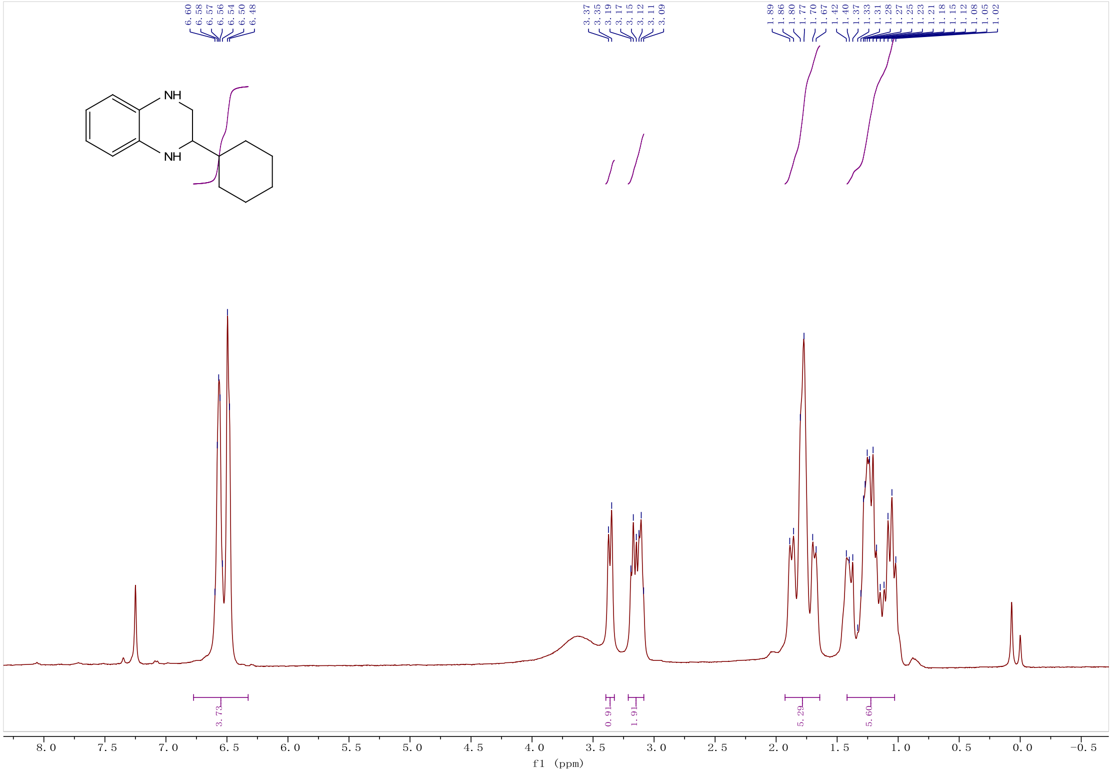

^{13}C NMR spectra for 2n

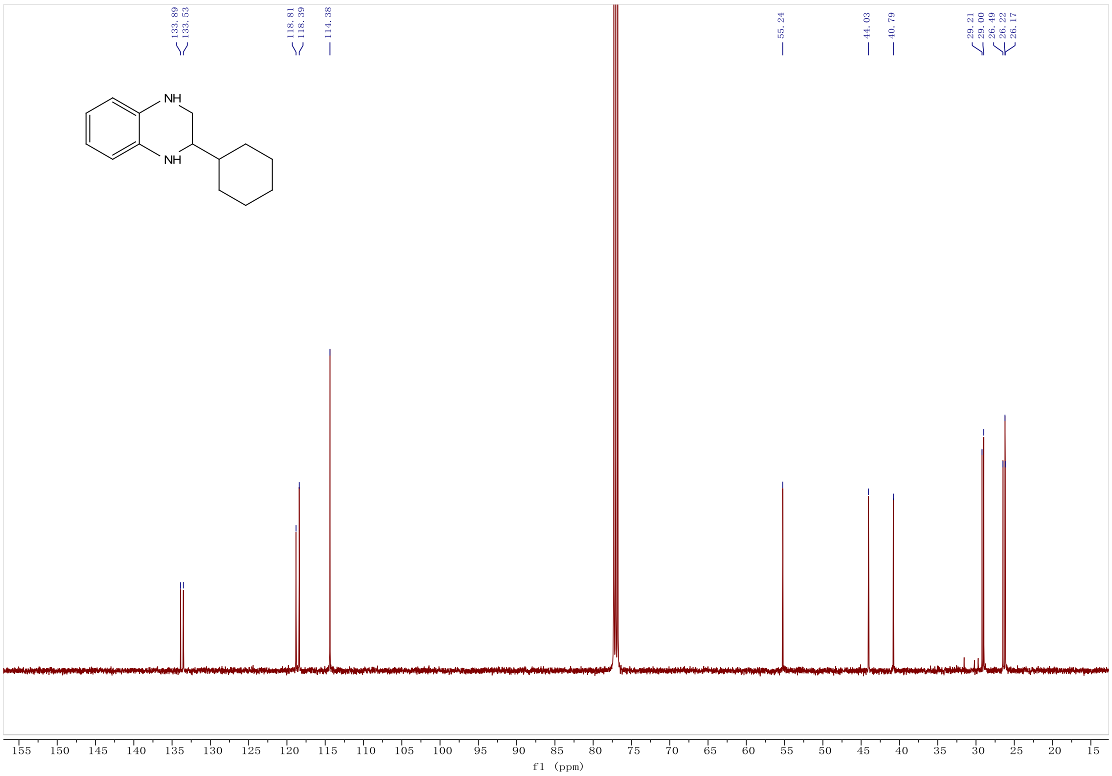

^{1}H NMR spectra for 2o

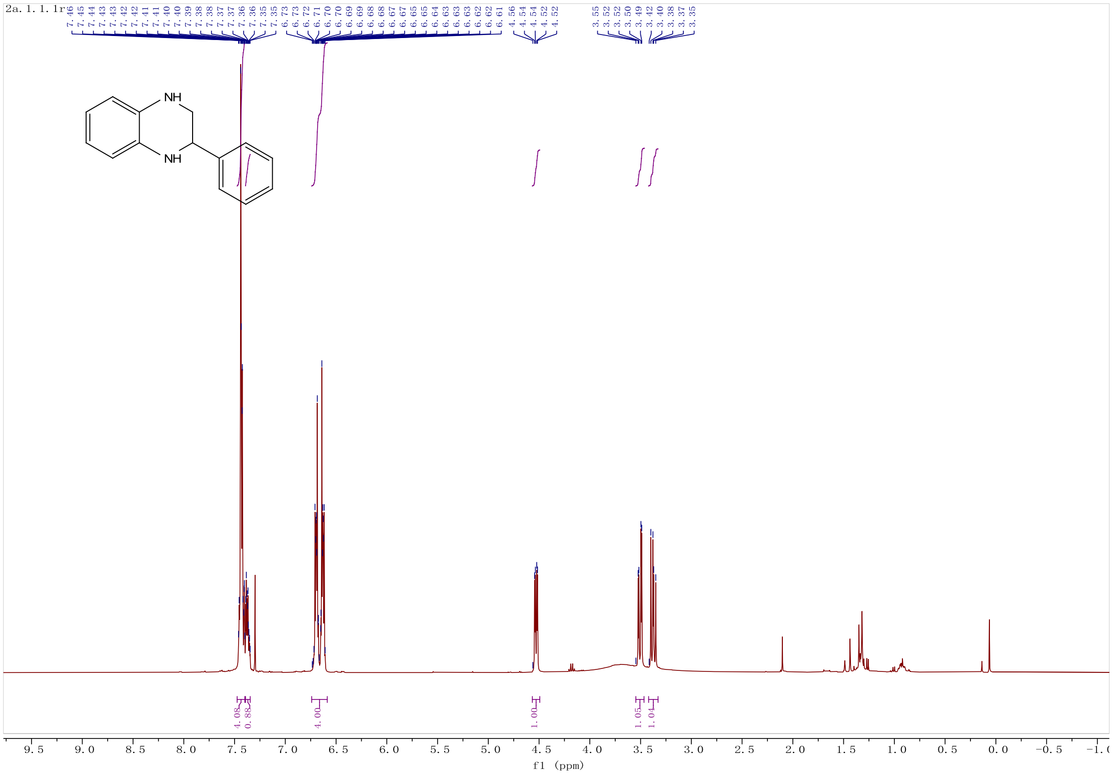

^{13}C NMR spectra for 2o

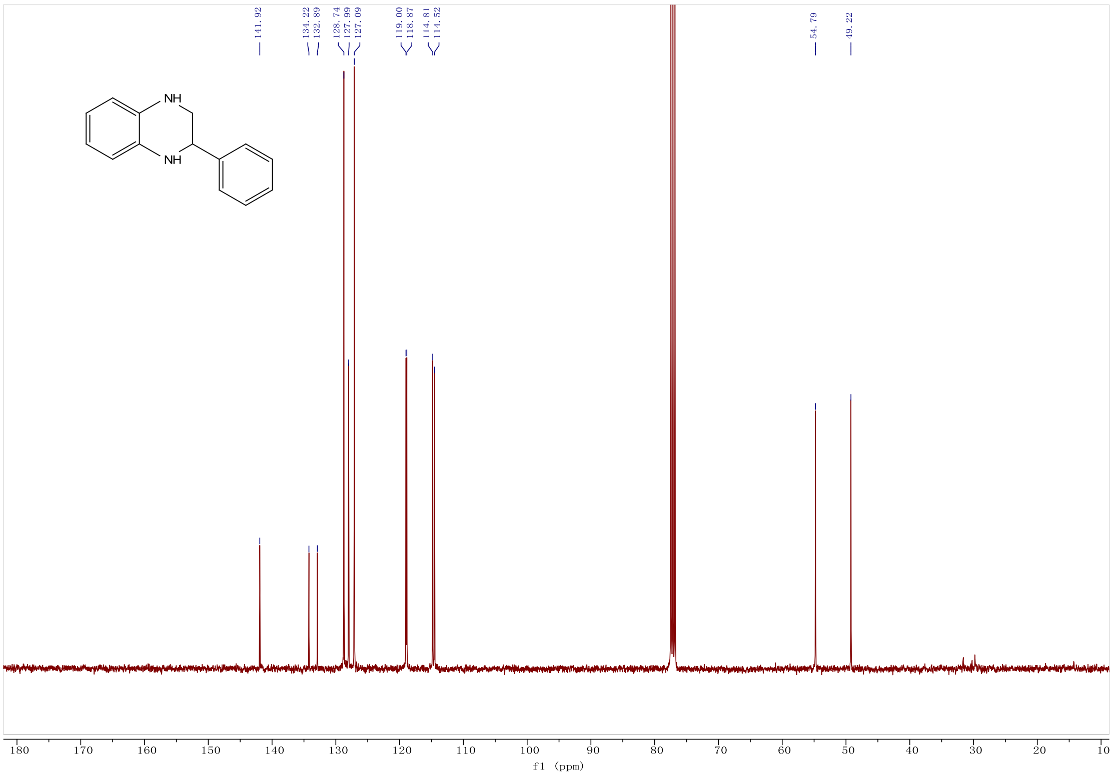

^{1}H NMR spectra for 4a

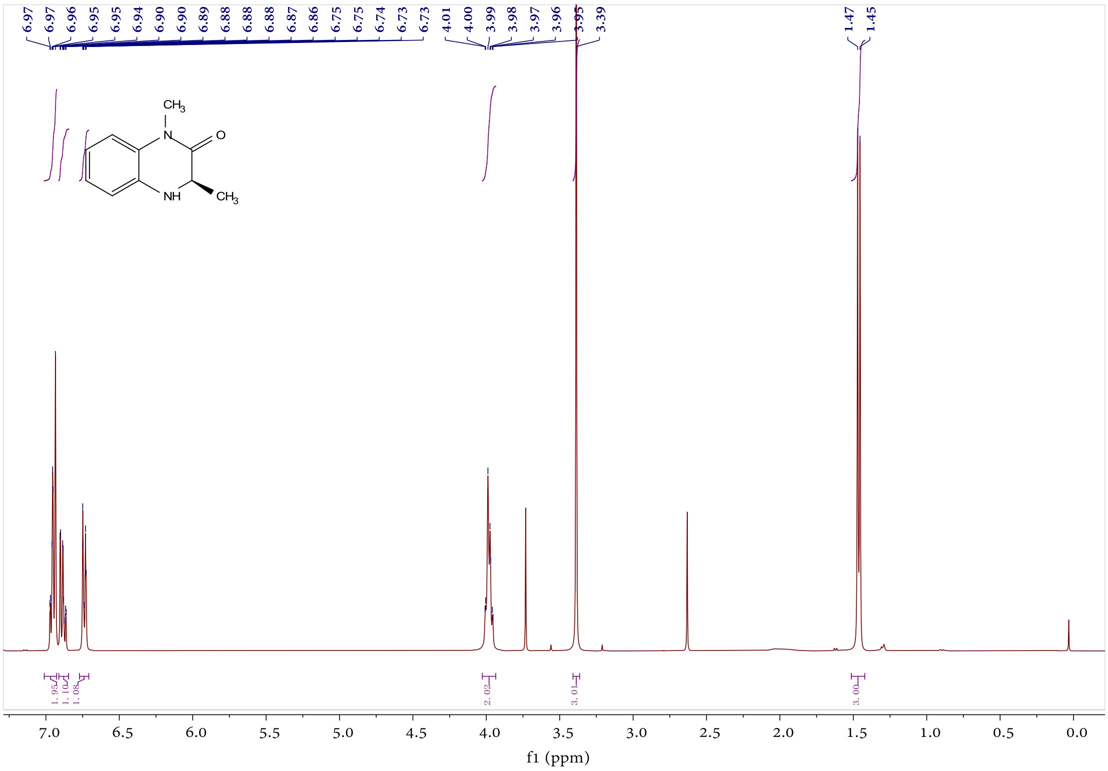

^{13}C NMR spectra for 4a

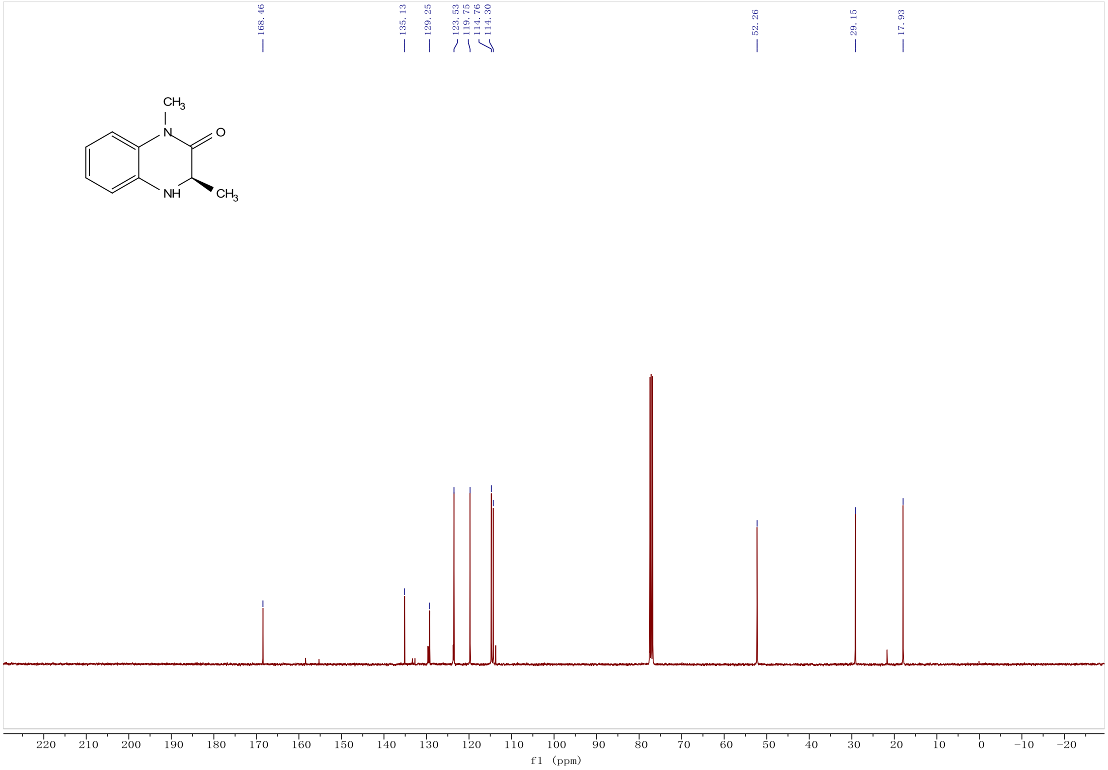

^{1}H NMR spectra for 4b

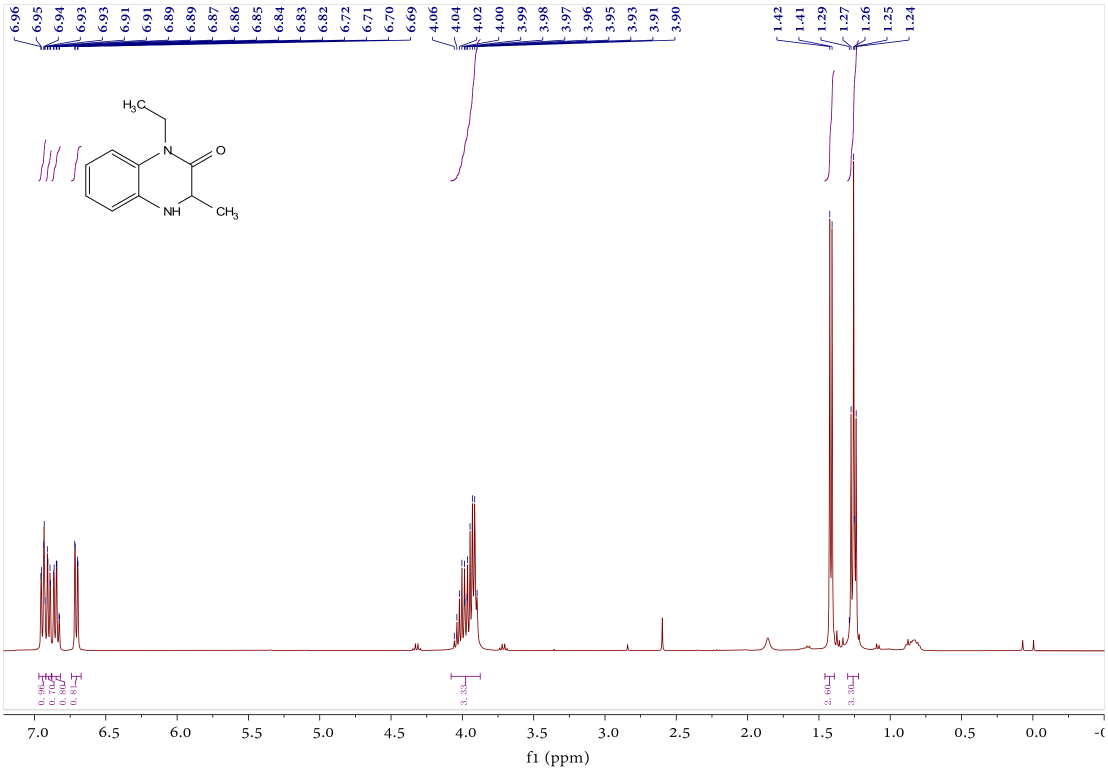

^{13}C NMR spectra for 4b

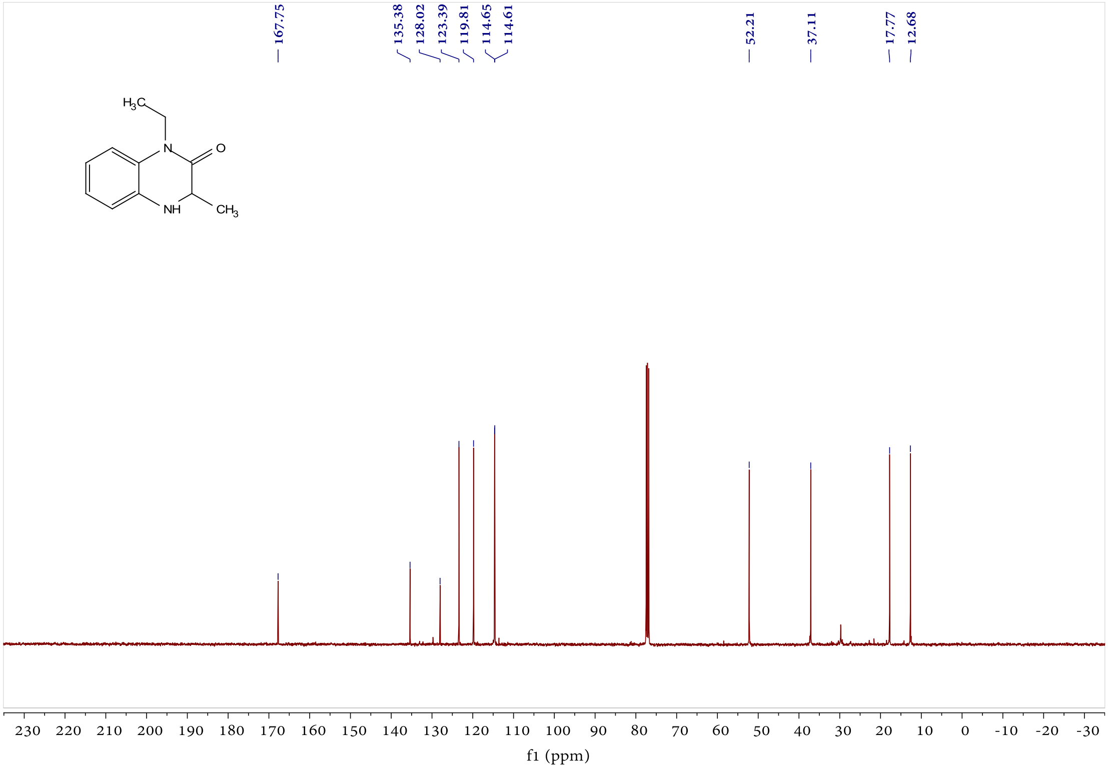

^{1}H NMR spectra for 4c

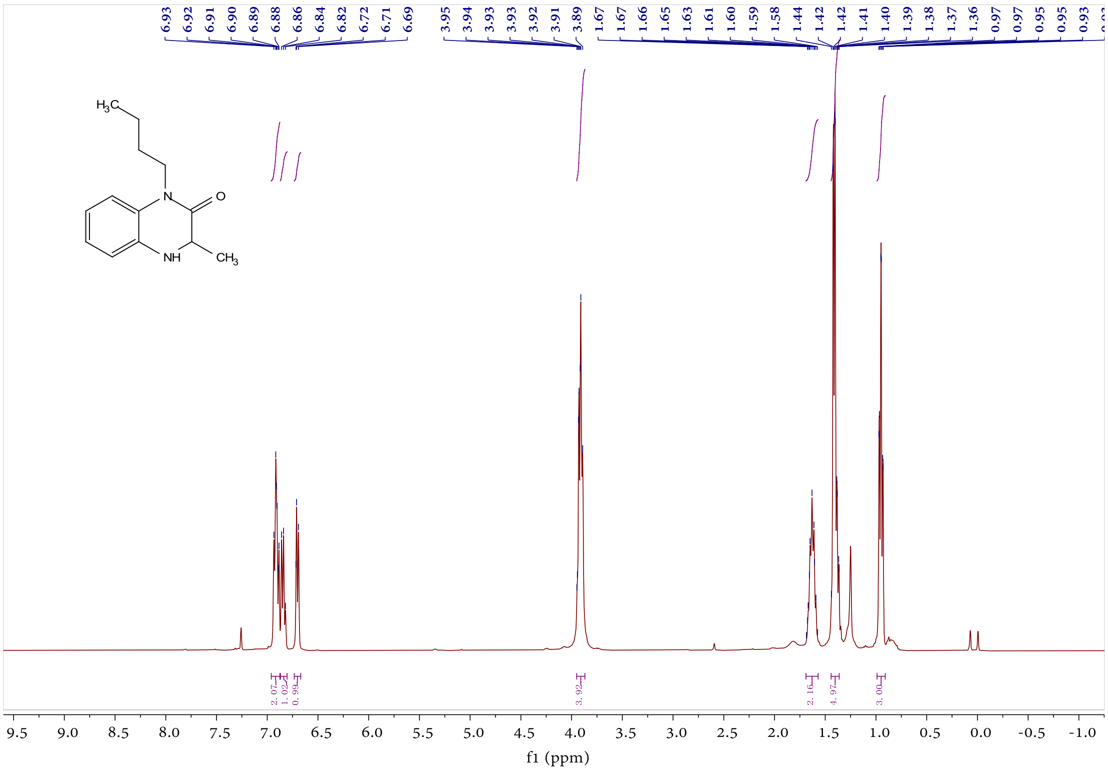

^{13}C NMR spectra for 4c

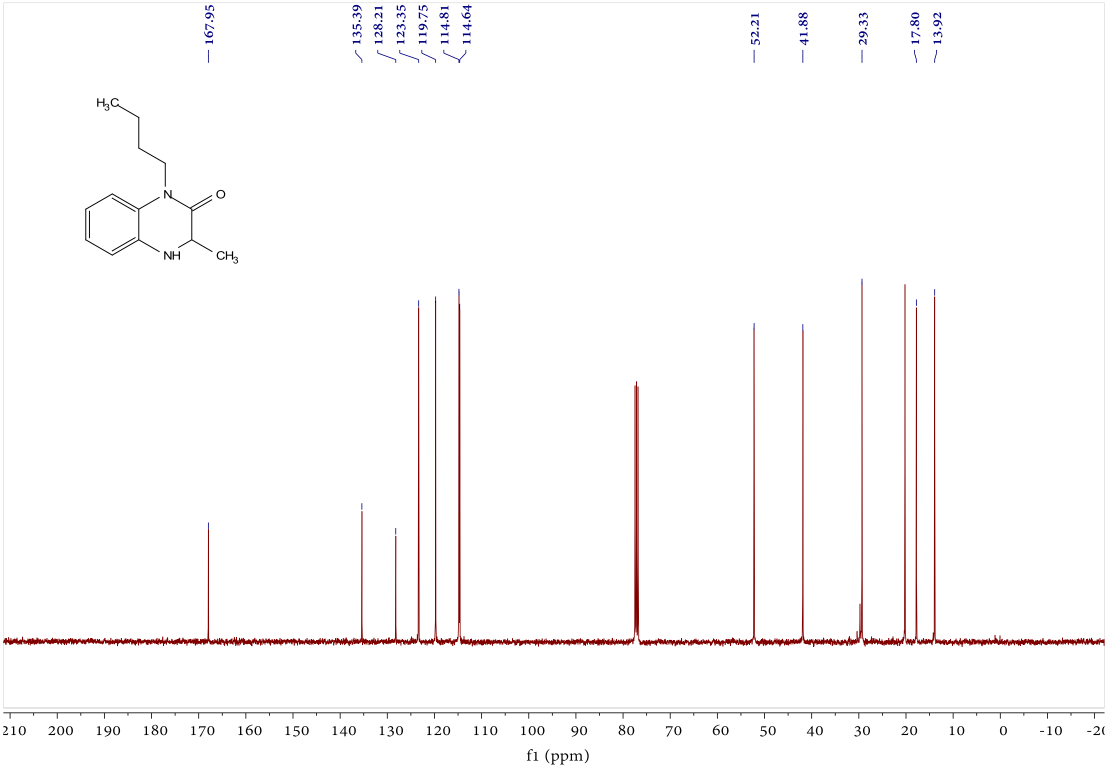

^{1}H NMR spectra for 4d

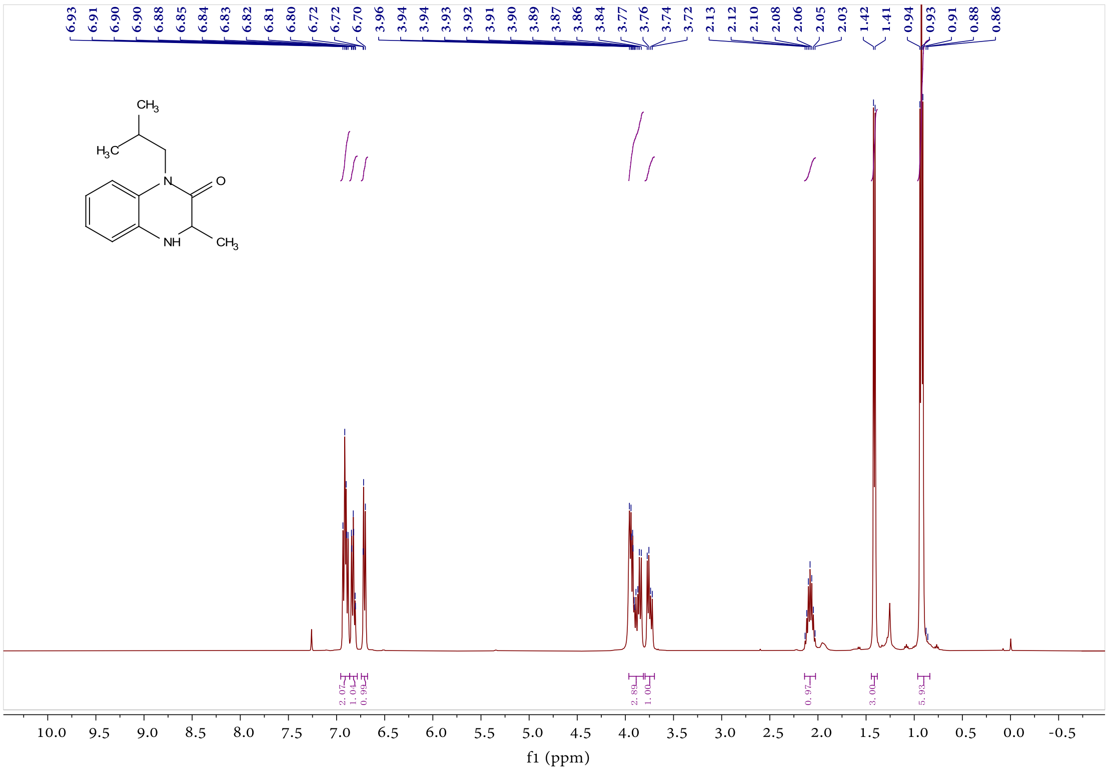

^{13}C NMR spectra for 4d

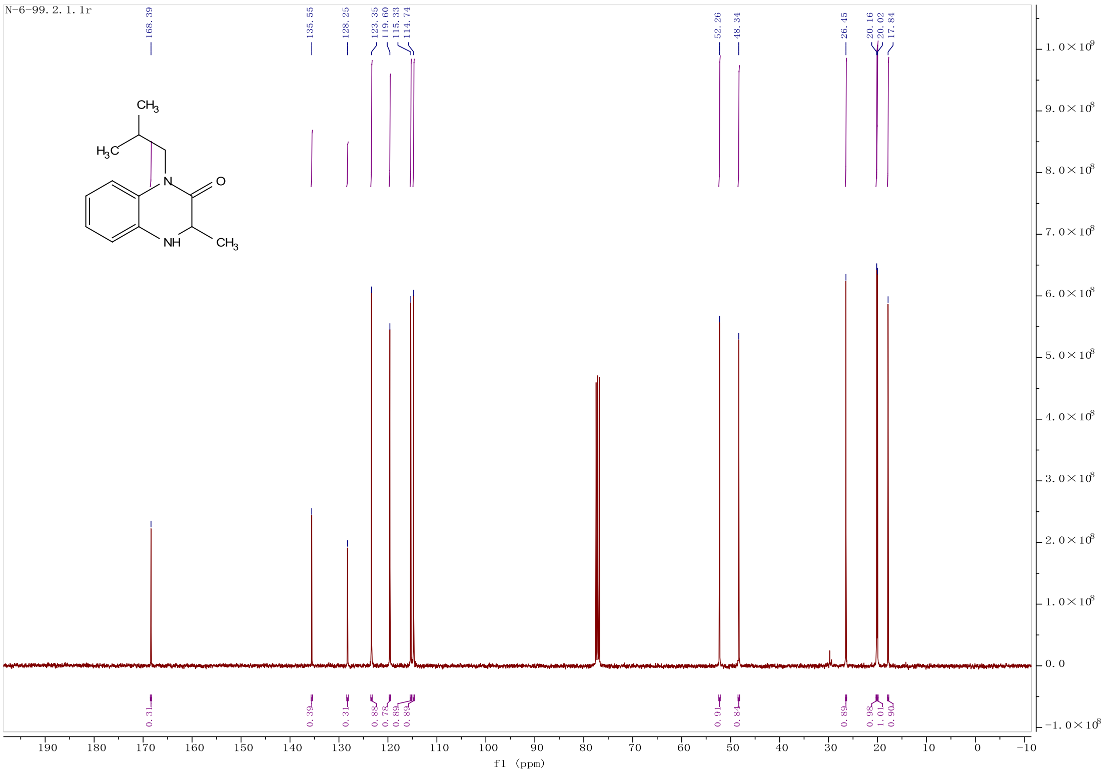

^{1}H NMR spectra for 4e

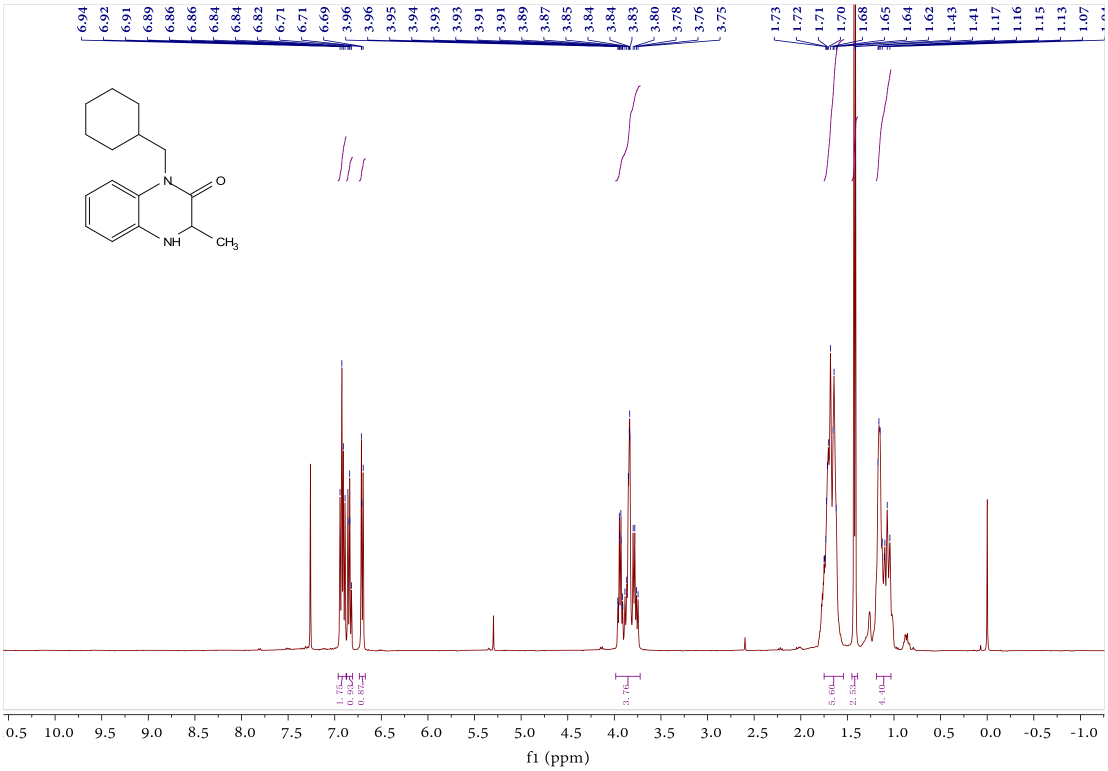

^{13}C NMR spectra for 4e

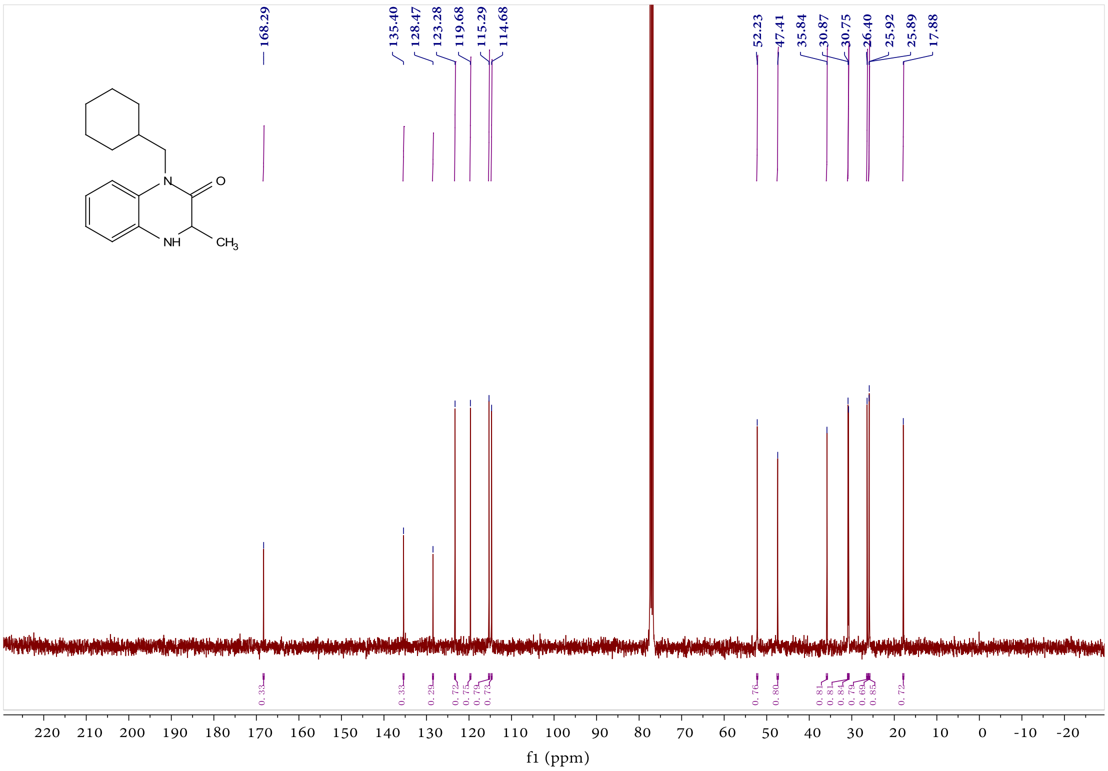

^{1}H NMR spectra for 4f

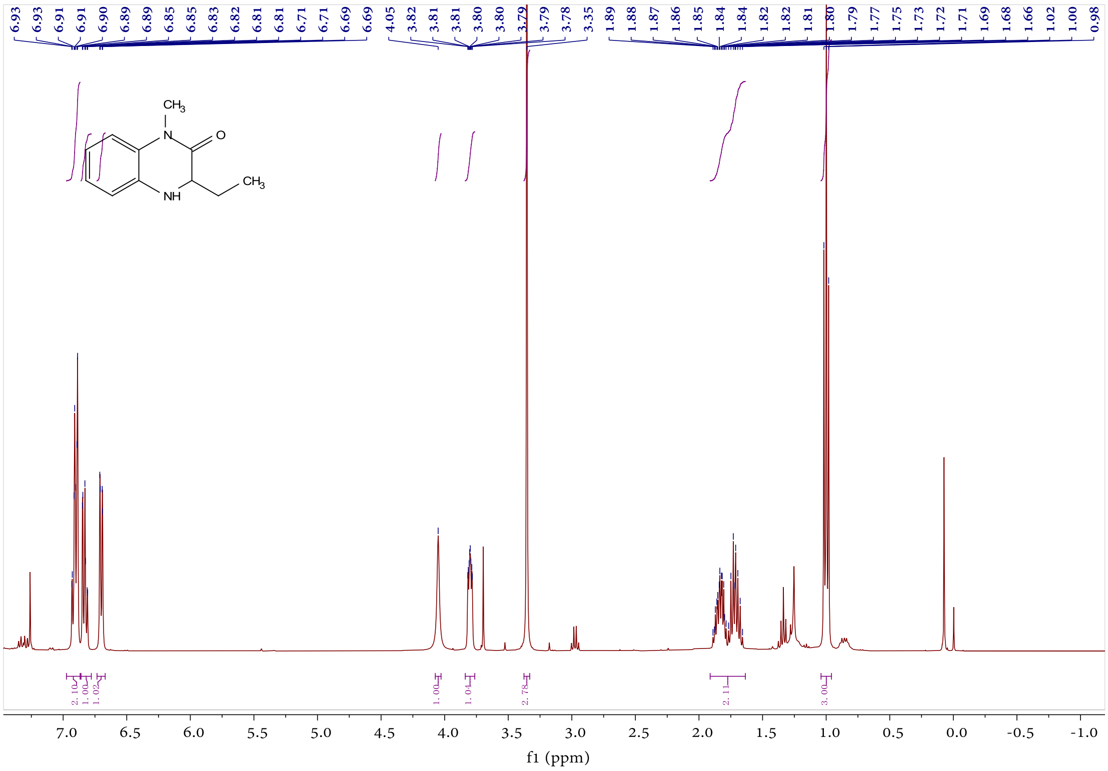

^{13}C NMR spectra for 4f

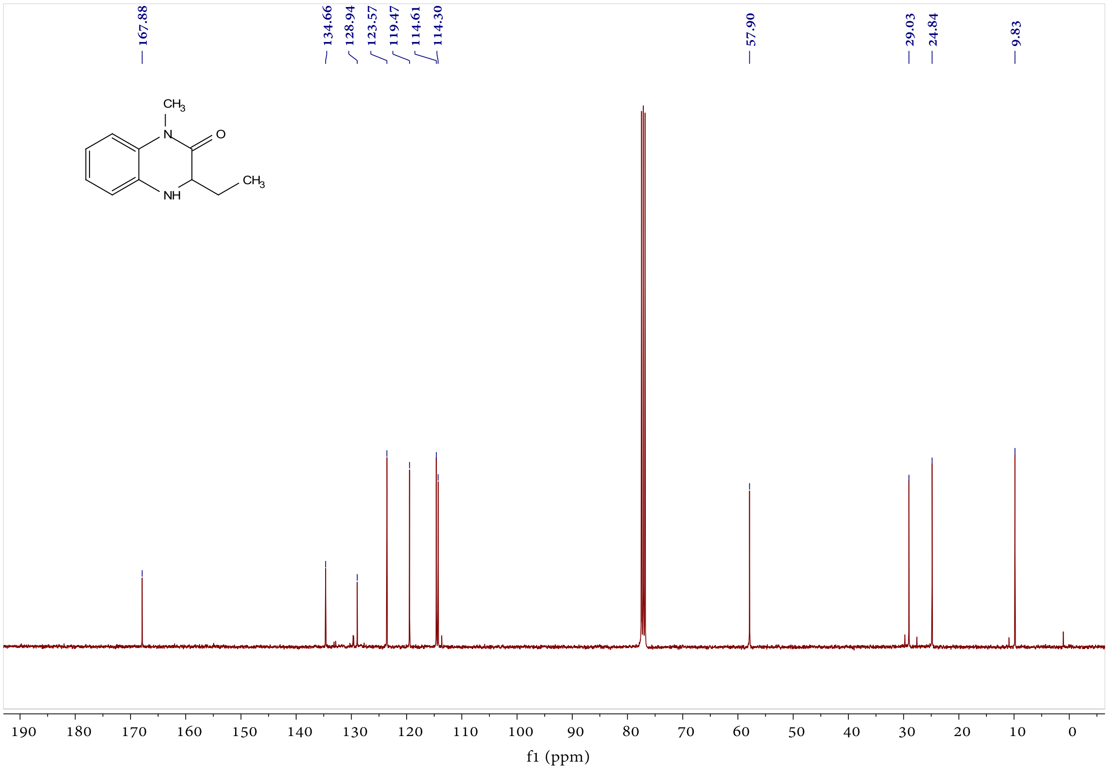

^{1}H NMR spectra for 4g

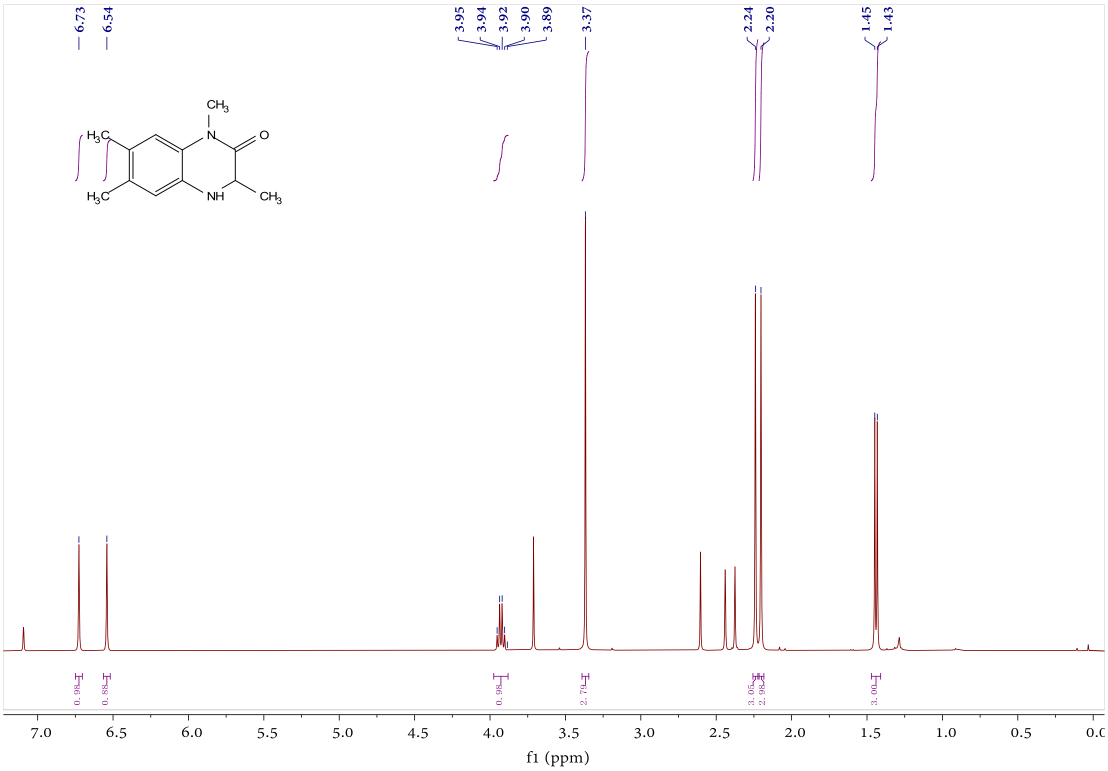

^{13}C NMR spectra for 4g

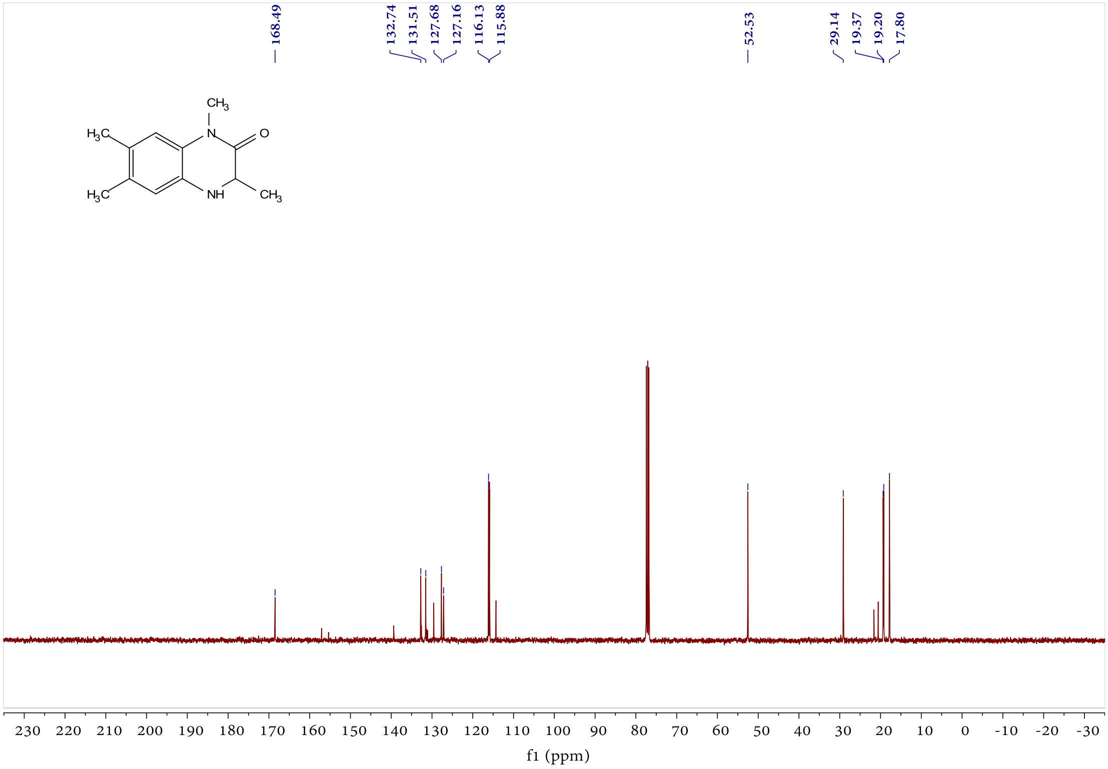

^{1}H NMR spectra for 4h

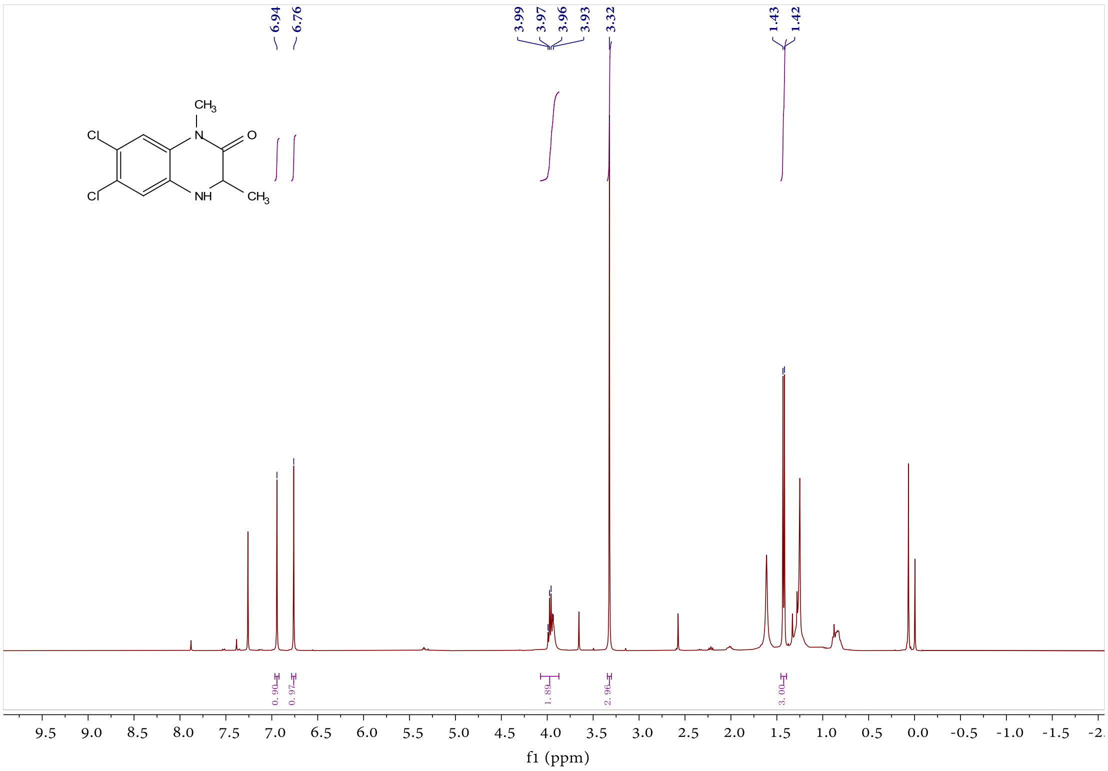

^{13}C NMR spectra for 4h

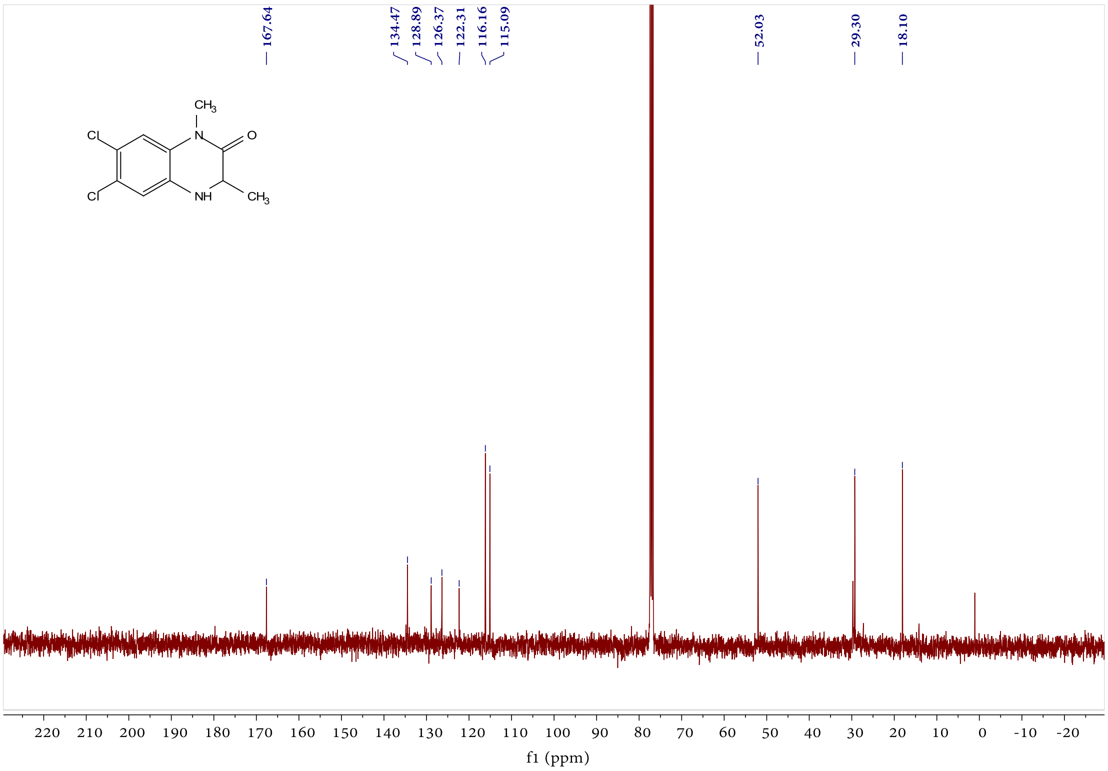

^{1}H NMR spectra for 4i

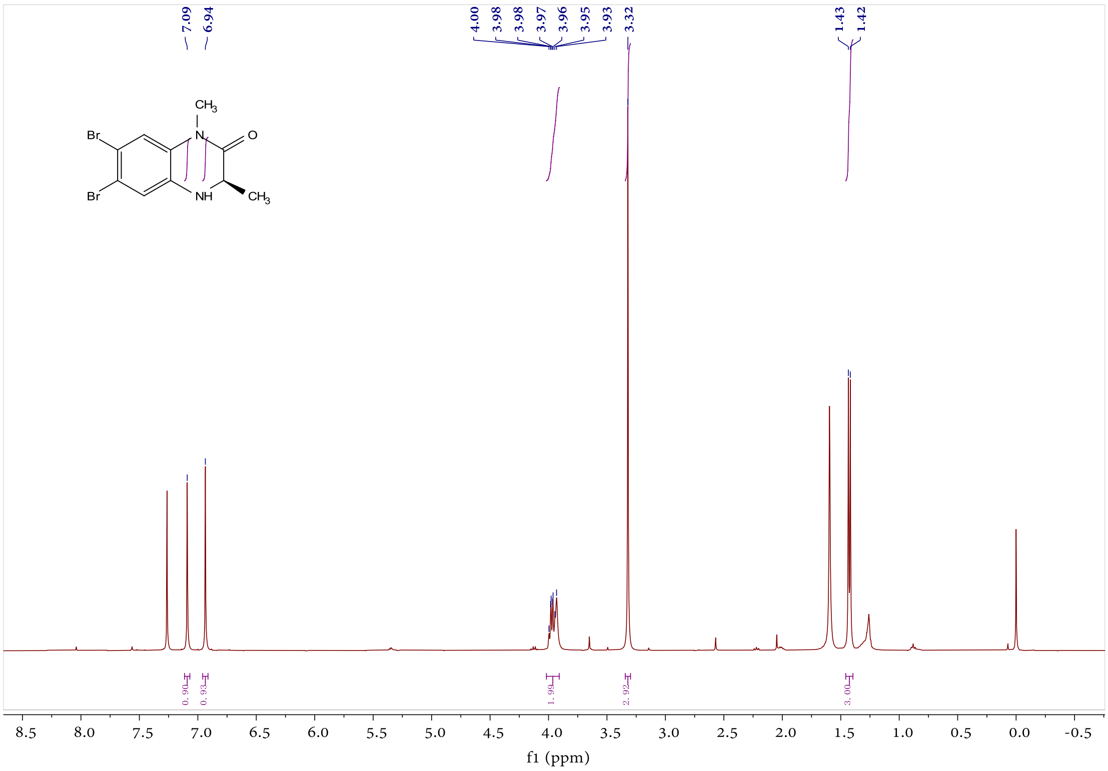

^{13}C NMR spectra for 4i

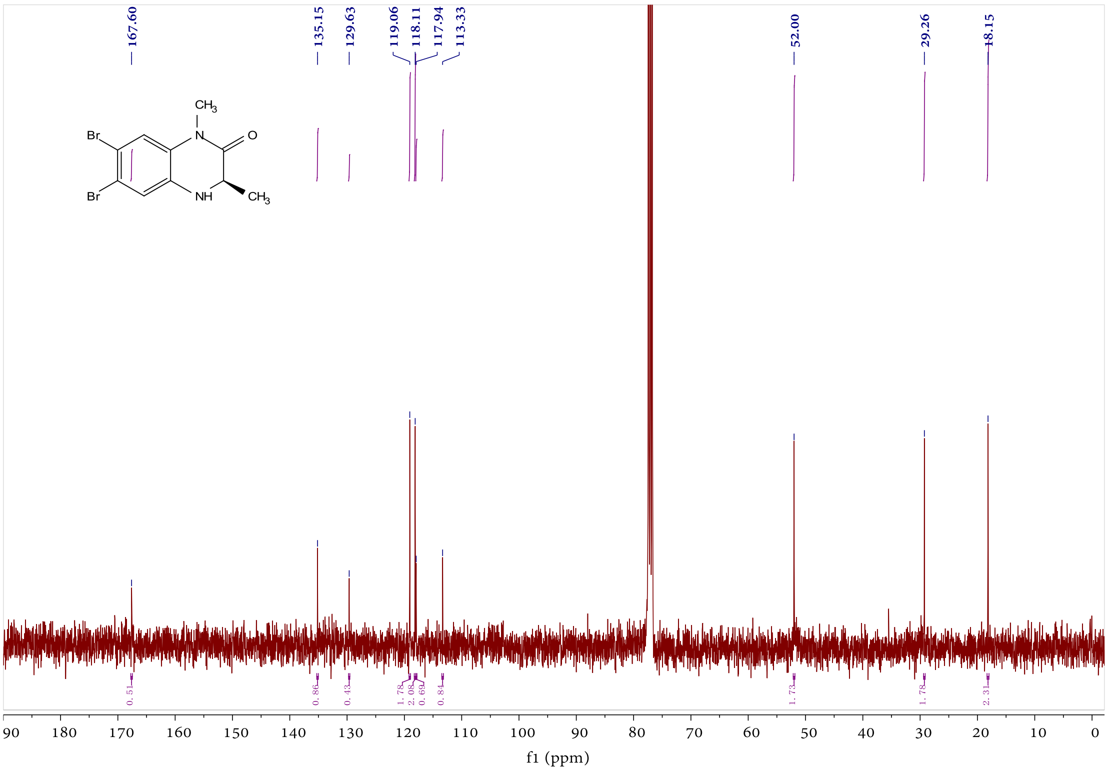

^{1}H NMR spectra for 4j

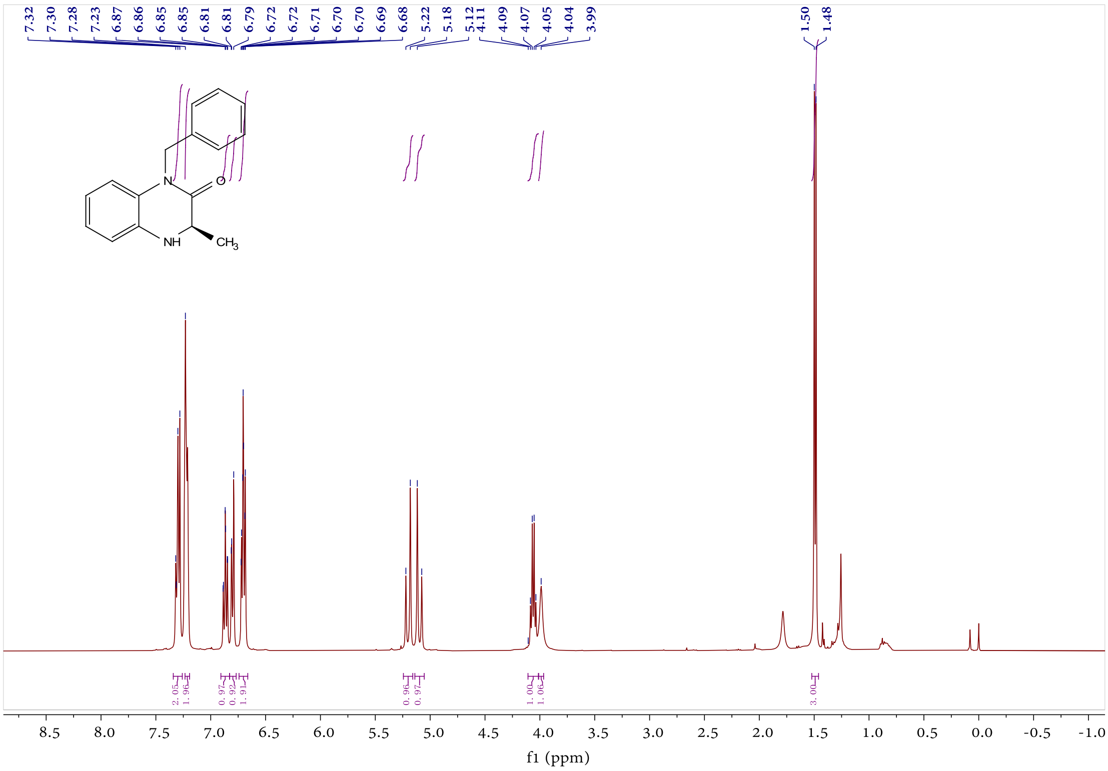

^{13}C NMR spectra for 4j

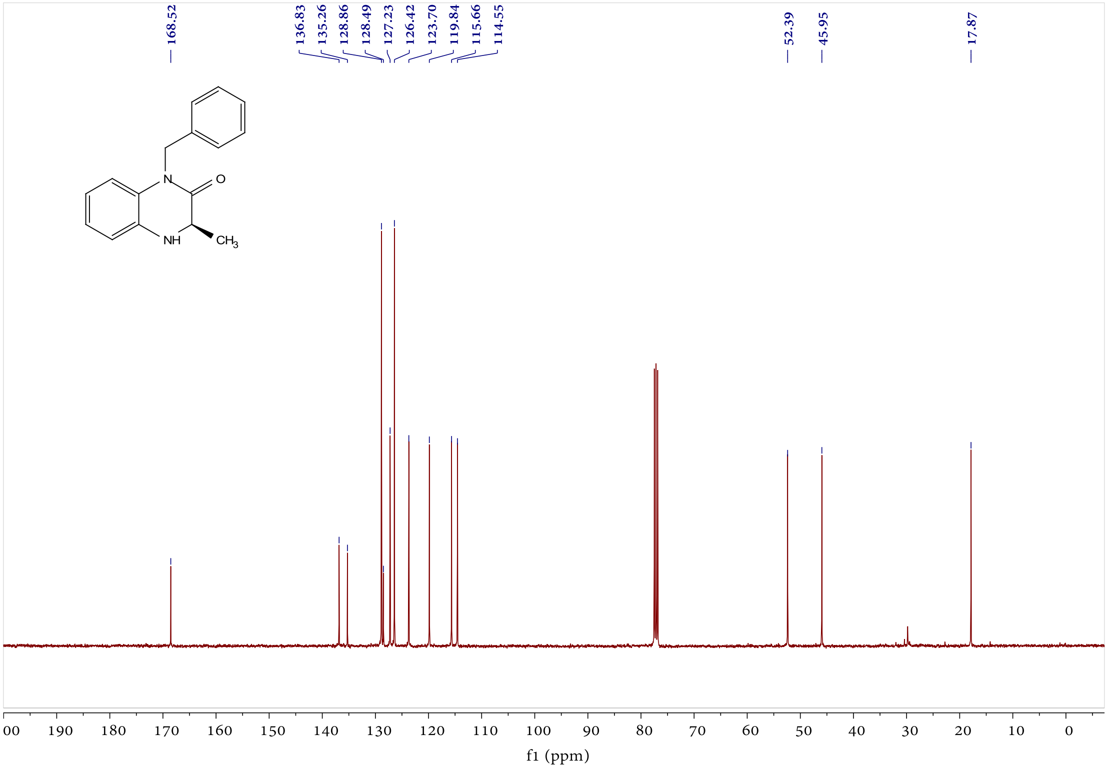

^{1}H NMR spectra for 4k

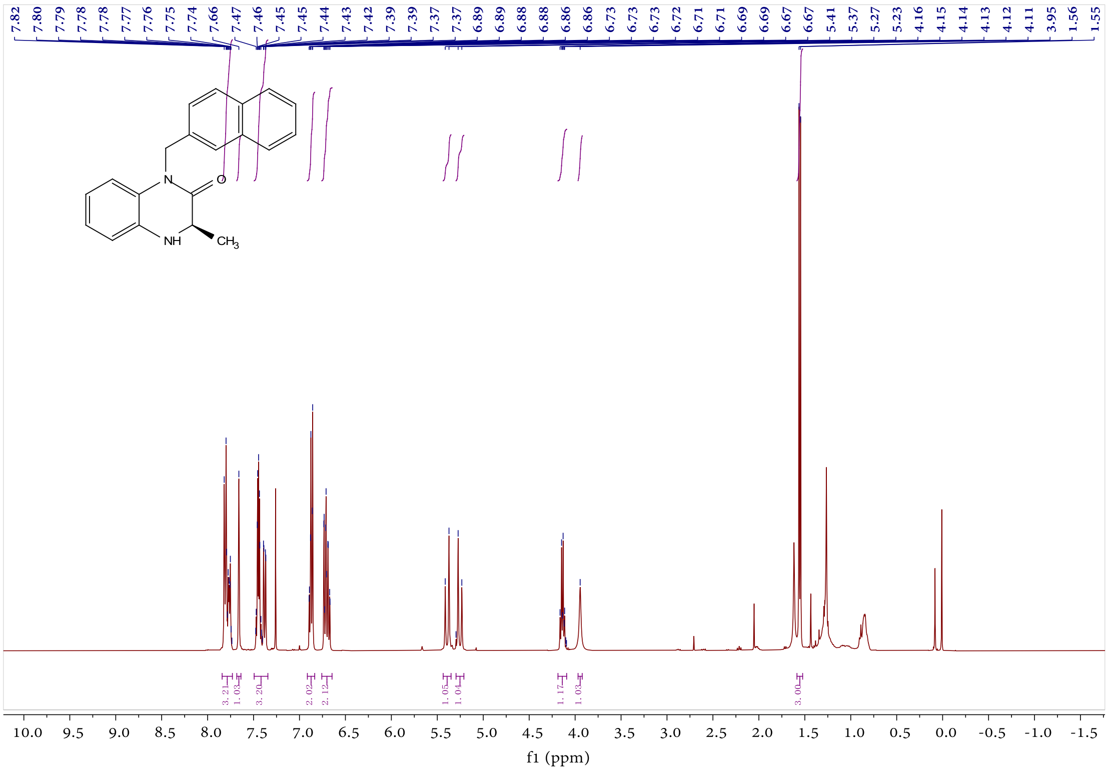

^{13}C NMR spectra for 4k

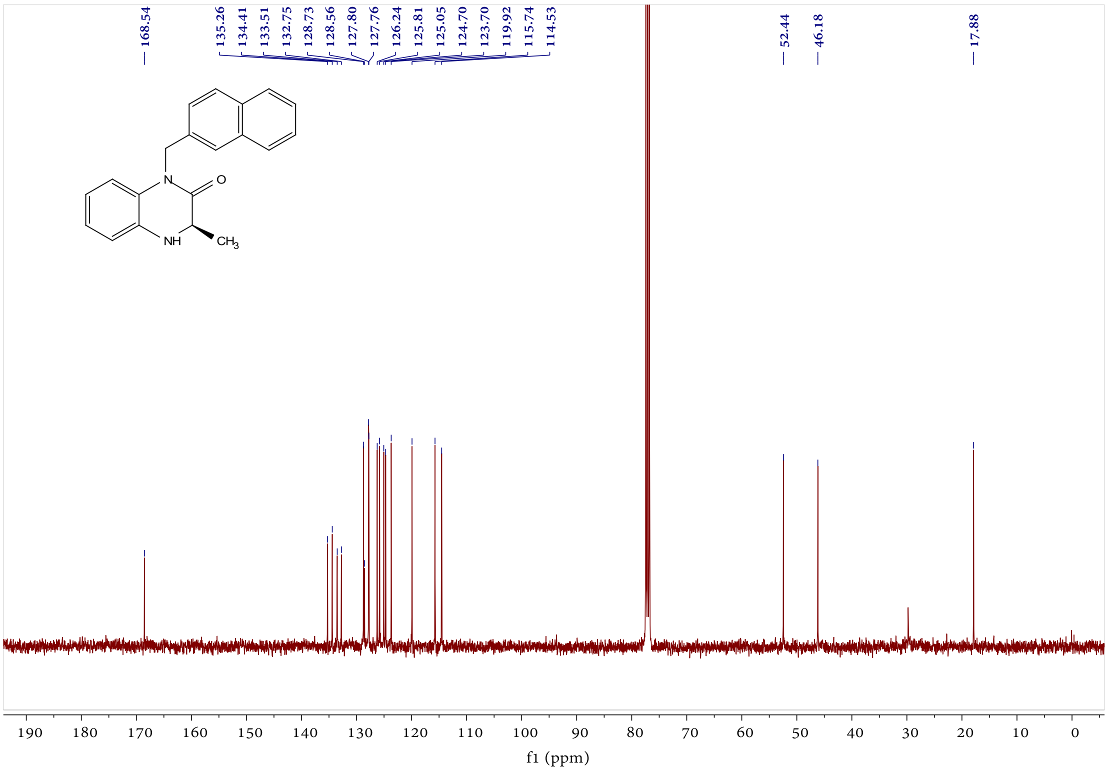

^{1}H NMR spectra for 4l

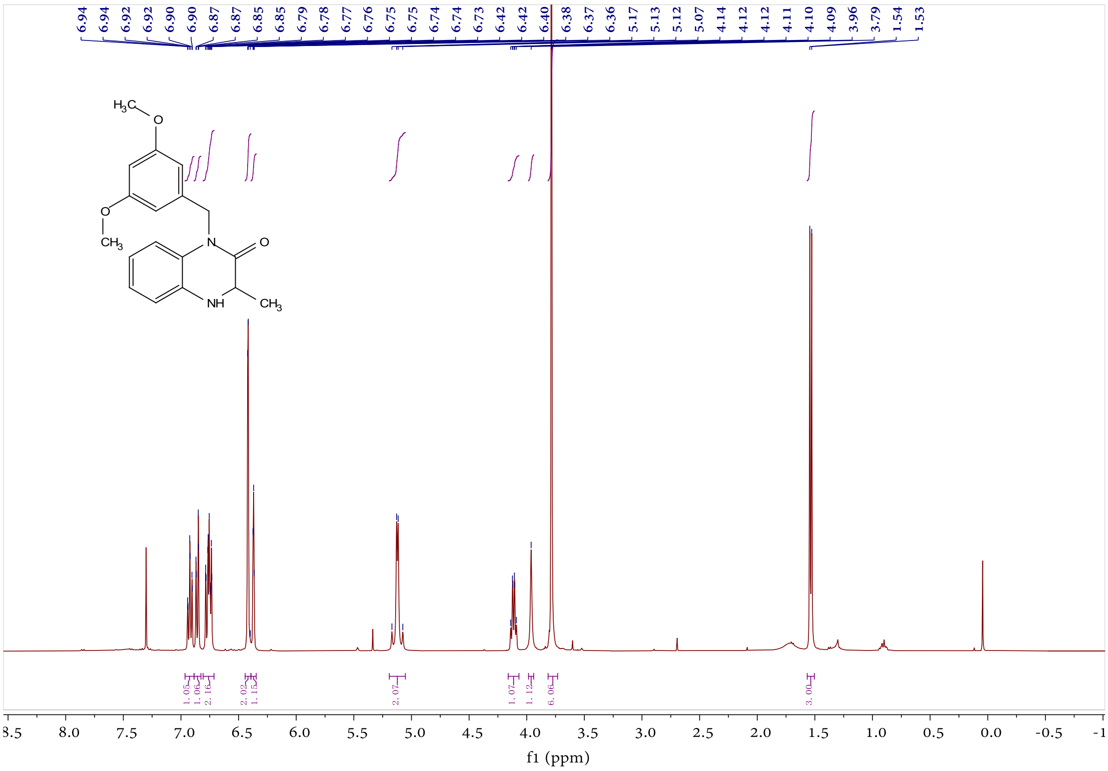

^{13}C NMR spectra for 4l

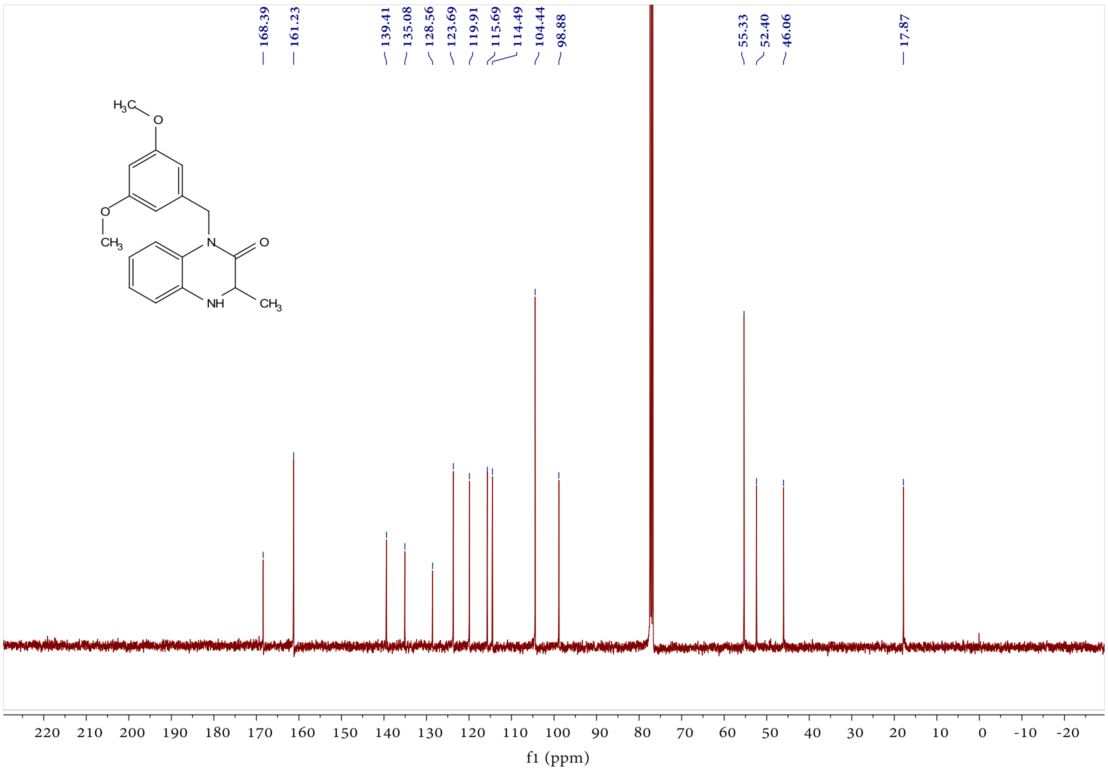

^{1}H NMR spectra for 4m

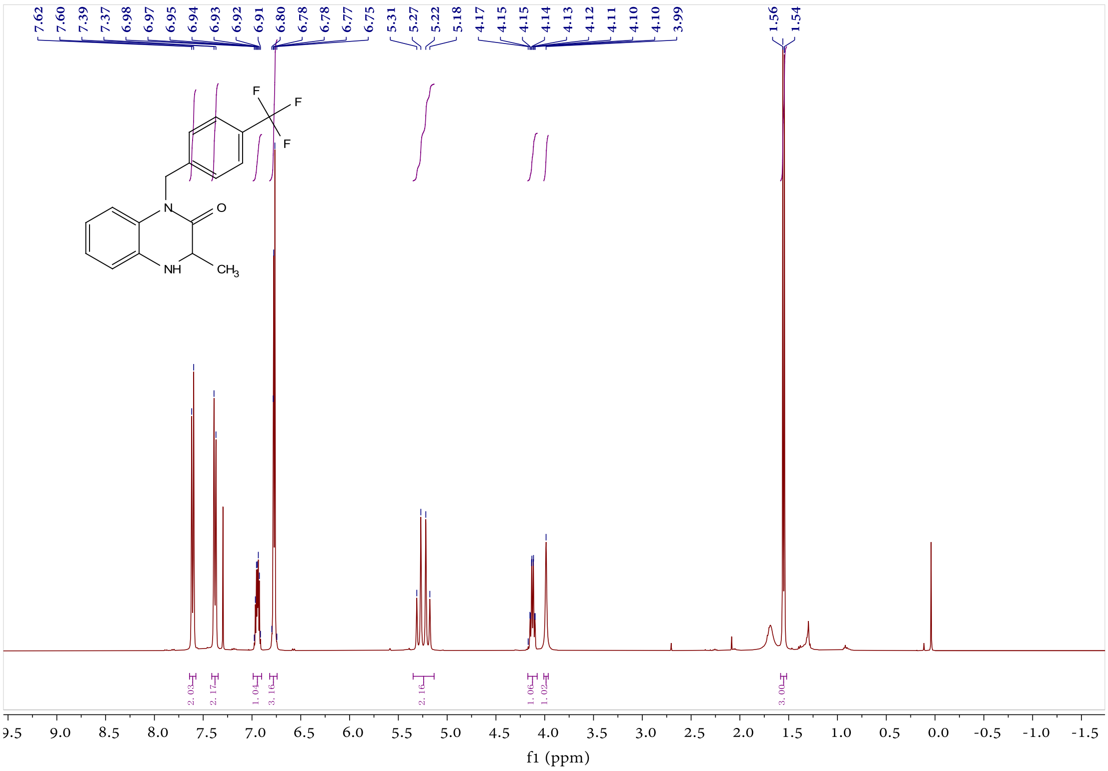

^{13}C NMR spectra for 4m

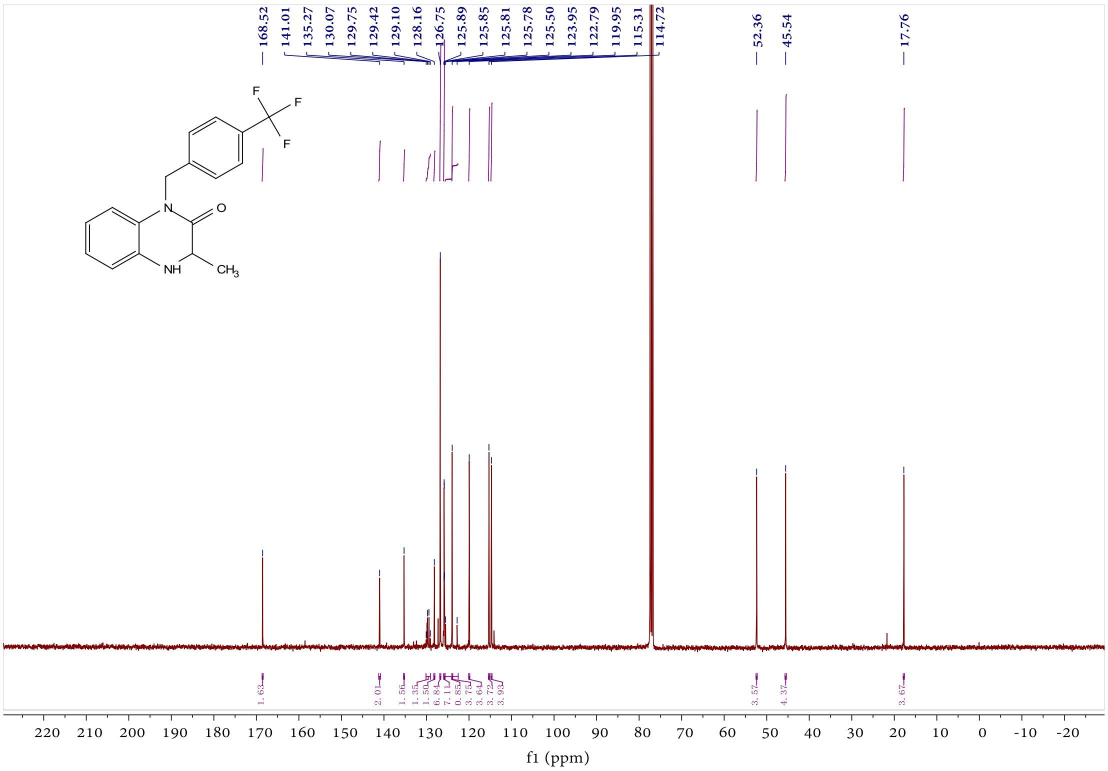

Supplement: SC-014-D3SC00803G-s001 [file SC-014-D3SC00803G-s001.pdf]
